# Brain aging in Type II GM1 gangliosidosis

**DOI:** 10.1038/s43856-026-01745-w

**Published:** 2026-07-06

**Authors:** Connor J. Lewis, Selby I. Chipman, Precilla D’Souza, Jean M. Johnston, Muhammad H. Yousef, William A. Gahl, Cynthia J. Tifft, Maria T. Acosta

**Affiliations:** 1 Office of the Clinical Director, National Human Genome Research Institute, Bethesda, MD USA; 2 Medical Genetics Branch, National Human Genome Research Institute, Bethesda, MD USA; 3 Department of Perioperative Medicine, National Institutes of Health Clinical Center, Bethesda, MD USA

**Keywords:** Neurological disorders, Biomarkers, Neurodegeneration, Paediatric neurological disorders

## Abstract

**Background:**

GM1 gangliosidosis is an inherited, progressive, and fatal neurodegenerative lysosomal storage disorder with no approved treatment. In this study, we present the first use of MRI-based predicted brain age in GM1 gangliosidosis aimed at assessing the neuronal degeneration in this cohort.

**Methods:**

We calculated a predicted brain age and Brain Structures Age Gap Estimation (BSAGE) for 81 MRI scans from 41 Type II (26 juvenile and 15 late-infantile) GM1 gangliosidosis patients and 897 MRI scans from 556 neurotypical controls (NC) utilizing *BrainStructuresAges*, a machine learning MRI analysis pipeline.

**Results:**

NC shows whole-brain aging at a rate of 0.83 per chronological year compared with 1.57 in juvenile GM1 patients and 12.25 in late-infantile GM1 patients. Accelerated and distinct brain aging is observed throughout midbrain structures, including the thalamus and caudate nucleus, hindbrain structures, including the cerebellum and brainstem, and the ventricles in juvenile and late-infantile GM1 patients compared to NC, reflecting neurodegeneration from disease progression. Cross-sectional evaluations of BSAGE for the whole brain show an average BSAGE = 35.50 years in the late-infantile cohort, 21.19 years in the juvenile cohort, and 0.03 years in NC. Predicted brain age and BSAGE both correlate with cross-sectional and longitudinal clinical outcome assessments.

**Conclusions:**

Predicted brain age accurately reflects the neurodegenerative clinical trajectories of the Type II GM1 disease subtypes. Correlations with clinical outcome assessments indicate evaluations of predicted brain aging may be an important candidate neuroimaging outcome measure for clinical trials in GM1 gangliosidosis.

## Plain language summary

GM1 gangliosidosis is an ultrarare genetic disease that causes progressive degeneration to the brain in affected patients. The aim of the present study was to evaluate the neuroimaging marker, predicted brain age in GM1 patients, compared with neurotypical controls from brain magnetic resonance imaging data. Predicted brain ages were calculated using a machine learning analysis pipeline. We found that predicted brain ages accurately reflected the clinical disease progression of GM1. Evaluations of predicted brain aging may be useful for evaluating diseases affecting the brain and for evaluating treatments for these diseases.

## Abstract (web summary)

Lewis et al. calculate predicted brain ages in Type II GM1 gangliosidosis patients and neurotypical controls from brain magnetic resonance imaging. Accelerated brain aging is observed in GM1 patients compared to controls and correlates with clinical outcomes.

## Introduction

GM1 gangliosidosis is a fatal, ultra-rare, neurodegenerative lysosomal storage disorder caused by biallelic mutations in *GLB1* encoding β-galactosidase^1^, which cleaves the terminal β−1,4 galactose of GM1 gangliosides, producing GM2 gangliosides for further catabolism^2^.

Dysfunction of β-galactosidase causes the accumulation of toxic GM1 gangliosides primarily in the central nervous system (CNS), where the rate of synthesis is the highest^3^.

GM1 gangliosidosis affects one in every 100,000 to 200,000 live births^4^. Three clinical sub-types of GM1 gangliosidosis exist, based on the age of symptom onset, which is inversely correlated with the amount of residual enzyme activity^5,6^. Type I (Infantile) GM1 gangliosidosis is the most severe, with death occurring by the age of three. Type II GM1 gangliosidosis patients can be further divided into late-infantile and juvenile classifications based on the age of symptom onset^7^. Late-infantile GM1 patients have symptom onset after 1 year of age; juvenile GM1 gangliosidosis patients have onset between 3 and 5 years^7^. The adult or chronic form of GM1 gangliosidosis is the least severe, with symptom onset typically in the second or third decade and characterized by prominent Parkinsonian features^7,8^.

Type II GM1 gangliosidosis patients typically present with gait disturbances, falling, and slurring of speech^9^. Ataxia, dystonia, dysarthria, skeletal changes, elevated liver function tests (especially aspartate aminotransferase), strabismus, decreased mobility, abnormal electroencephalography (EEG), and swallowing difficulties are all common in the Type II form of the disease. Late-infantile GM1 gangliosidosis patients typically survive into the second decade; juvenile patients can survive into the fourth decade^7,9^.

Numerous neuroimaging findings are associated with Type II GM1 gangliosidosis. Volumetric studies have shown decreased volumes of the whole brain, cerebellum, lentiform nucleus, thalamus, corpus callosum, and caudate nucleus, with consequent enlargement of the lateral ventricles^10–13^. T1-weighted signal abnormalities of the bilateral globus pallidum, increased signal attenuation in the thalamus, and decreased signal attenuation of the basal ganglia have been documented, as well as decreased T2-weighted signal intensity in the thalamus, globus pallidus, and substantia nigra, and hyperintensities of the putamen^14–17^. Magnetic resonance spectroscopy (MRS) shows diminished *N*-acetyl-aspartate (NAA) in the thalamus and centrum semi-ovale and increased myo-inositol in the centrum semi-ovale^7,17^.

There are no treatments for GM1 gangliosidosis^18^, although gene therapy clinical trials are currently underway^19^. As these trials move to the latter phases, outcome measures demonstrating the efficacy of the therapeutic intervention will be required. Due to the CNS pathology of GM1, analysis of anatomical magnetic resonance imaging could serve as an objective outcome measure^20,21^. In this study, we present the first use of MRI-based predicted brain age in GM1 gangliosidosis aimed at assessing the neuronal degeneration in this cohort to the best of our knowledge. These findings could serve as imaging outcome parameters for demonstrating therapeutic efficacy attendant to interventions such as gene therapy.

We show predicted brain aging is accelerated in type II GM1 patients compared to similarly aged neurotypical controls. We also show that predicted brain aging is accelerated in the more severe late-infantile GM1 clinical subtype compared to the juvenile subtype. We find in region-specific analysis, accelerated brain aging in structures known to be affected by GM1 in previous imaging and pathologic studies describing gliosis and neuronal loss. Measures of predicted brain aging correlated with GM1 clinical outcome assessments, which suggests importance as a neuroimaging marker of disease progression.

## Methods

### Type 2 GM1 Gangliosidosis participants

Forty-one GM1 patients from the “Natural History of Glycosphingolipid and Glycoprotein Disorders” protocol (ClinicalTrials.gov ID: NCT00029965) were included in this study^22^. The NIH Institutional Review Board approved this protocol (02-HG-0107), registered on January 28, 2002. Informed consent was completed with the parents or legal guardians of the patients. All participants were assessed for their ability to provide assent; none were deemed capable. As described in D’Souza et al^7^, a GM1 diagnosis was based upon β-galactosidase enzyme deficiency and biallelic pathogenic variants in *GLB1*. Participants were classified into juvenile or late-infantile forms of GM1 based on the age of symptom onset. 61 MRI scans from 26 juvenile (baseline age: 9.6 ± 4.7 years) patients were included in this study alongside 20 MRI scans from 15 late-infantile patients (baseline scan age: 4.6 ± 1.8 years, supplementary Fig. 1, supplementary Table 1).

### Neurotypical control participants

To account for the nearly 25-year age range of GM1 gangliosidosis patients. Neurotypical control MRI scans were gathered from three open-source data sets. First, 97 participants (279 scans) from the Calgary Preschool MRI dataset were included, consisting of participants between the ages of 2 and 8 years (baseline scan age: 3.84 ± 0.88 years old)^23,24^. Second, 159 participants (318 scans) from the Queensland Twin Adolescent Brain (QTAB) were included in this study, consisting of participants between the ages of 9 and 16 years (baseline scan age: 10.93 ± 1.36 years old)^25,26^. Lastly, 300 participants (300 scans) from the Queensland Twin Imaging (QTIM) were included in this study, consisting of participants between the ages of 18 and 30 years (baseline scan age: 21.72 ± 3.71 years old, supplementary Fig. 2, and supplementary Table 2)^27,28^.

### T1-weighted MRI acquisition – GM1 patients

All GM1 patients’ MRI scans were performed under propofol sedation and were performed on a Philips 3T system (Achieva, Philips Healthcare, Best, The Netherlands) with an 8-channel SENSE head coil. Images were acquired using a 3D T1-weighted protocol with the following parameters: 1 mm isotropic resolution, TR of 11 ms, TE of 7 ms, flip angle = 6 degrees, FOV = 220 mm^11^. Unprocessed digital imaging and communications in medicine (DICOM) images were converted to NIfTI using *dcm2niix*^29^.

### T1-weighted MRI acquisition – Calgary preschool MRI

As described in Reynolds et al.^23,24^, Calgary preschool neurotypical controls were scanned without sedation on a General Electric 3 T system (MR750w, GE Healthcare, Chicago, IL, USA) using a 32-channel head coil. T1-weighted imaging from preschool neurotypical controls was acquired with TR/TE = 8.23/3.76 ms, flip angle = 12 degrees, and resolution of 0.9 mm × 0.9 mm × 0.9 mm (resampled to 0.45 mm × 0.45 mm × 0.9 mm), and 210 slices.

### T1-weighted MRI acquisition – Queensland Twin Adolescent Brain (QTAB) MRI

As described in Strike et al.^25,26^, participants from QTAB were scanned on a Siemens 3 T Prisma system with a 64-channel head coil at the Centre for Advanced Imaging at the University of Queensland, Australia. Images were acquired using a 3D T1-weighted Magnetization Prepared with 2 Rapid Gradient Echoes (MP2RAGE) imaging protocol with the following parameters: 0.8 mm isotropic voxels, TR/TE  =  4000/2.99 ms, flip angle of 6 or 7 degrees, FOV  =  256 × 240 mm, and 192 slices.

### T1-weighted MRI acquisition – Queensland Twin IMaging (QTIM) MRI

As described in Strike et al.^27,28^, Participants from QTIM were scanned on a 4 T Bruker Medspec (Bruker, Germany) with a transverse electromagnetic (TEM) head coil. Images were acquired using a 3D T1-weighted protocol with the following parameters: 0.9 mm isotropic voxels, TR/TE = 1500/3.35 ms, FOV = 230 mm, and 256 or 240 slices.

### Ethics declaration

With regard to the GM1 patients, the NIH Institutional Review Board approved this protocol (02-HG-0107). Informed consent was completed with the parents or legal guardians of the patients. All participants were assessed for their ability to provide assent; none were deemed capable. All three NC datasets received ethics approval from their respective institutions for data collection and sharing. With regards to the Calgary study as described in Reynolds et al.^23^, the University of Calgary Conjoint Health Research Ethics Board (CHREB) approved this research project (REB13-0020)^23^. The QTAB study, as described in Strike et al.^25^, ethics approval for this study was originally obtained through the Children’s Health Queensland Human Research Ethics Committee (HREC) (reference HREC/16/QRCH/270) and the University of Queensland HREC (reference 2016001784)^25^. The QTIM study, as described by Strike et al.^27^, the study was approved by the Human Research Ethics Committees of the University of Queensland, QIMR Berghofer Medical Research Institute, and UnitingCare Health.

### MRI Processing

All MRI scans from GM1 patients and NC were sent through volBrain’s *BrainStructuresAges* pipeline^30–32^. There were no excluded MRI scans or scans that failed to process. *BrainStructuresAges* is a deep learning MRI analysis pipeline trained with 2887 MRI scans from various sources to predict brain age^30^. The pipeline was then shown to be more accurate in terms of mean absolute error and R^2^ than other state-of-the-art models in analyzing more than 30,000 MRI scans. We used *BrainStructuresAges* to predict ages for the whole brain, white matter, 3rd ventricle, 4th ventricle, lateral ventricles, inferior lateral ventricles, cerebellar gray matter, cerebellar white matter, lobules I-V of the cerebellum, lobules VI and VII of the cerebellum, lobules VIII-X of the cerebellum, the caudate nucleus, thalamus, brainstem, hippocampus, nucleus accumbens, amygdala, ventral diencephalon (DC), external cerebrospinal fluid (CSF), basal forebrain, putamen, and globus pallidus in all participants. BSAGE was calculated as the predicted brain age minus the patient’s chronological age, as described in Nguyen et al^30^.

### Clinical global impression (CGI) scores

To determine the clinical relevance of predicted brain age in GM1 gangliosidosis participants, we correlated these metrics with clinical global impression (CGI) scores. CGI scores a clinician-rated outcome measure assessing the clinical severity of a patient^33,34^. Clinical Global Impressions were scored by consensus after a retrospective chart review as described in Lewis et al.^35^, baseline CGI Severity (CGI-S) scores were assigned 1 = ‘normal’ to 7 = “among the most extremely” at the patients’ baseline evaluation.

### Vineland Adaptive Behavior Composite (ABC)

The Vineland Adaptive Behavioral Composite (ABC) Standard Scores are a semi-structured clinical interview with the patient and their caregiver^36,37^. The ABC domain of the Vineland is a functional impairment measure with a standard score that has a population mean of 100 and a standard deviation of 15. Vineland-3 scores were used when available, and Vineland-II scores were used when the third edition was not available (neurodevelopmental assessment was conducted before the 3rd edition’s release)^35^. As described in D’Souza et al.^7^, GM1 gangliosidosis patients demonstrated functional impairments assessed by the Vineland scale, including the ABC and fine motor, gross motor, socialization, daily living, and communication subdomains in both late-infantile and juvenile GM1 patients.

### Statistics and Reproducibility

Linear mixed effects modeling (LMEM) in this study was performed in R (The R Foundation, v4.3.1), with the lme4 package^38^. The effect of participant sex (fixed effect) was modeled as a covariate of no interest, and a subject-level random intercept was used to account for repeated T1 scans conducted for each participant^39,40^. Males were assigned a value of 1, and females were assigned a value of 0 to remove the effect of sex from LMEM. Between-group effects were assessed as the interaction between participants’ chronological age and their group on predicted brain age. For comparisons between GM1 gangliosidosis patients and neurotypical controls, NC were assigned a value of 0, and GM1 gangliosidosis patients were assigned a value of 1 to test the effects of GM1 gangliosidosis on the age predictions. For comparisons between late-infantile GM1 patients and neurotypical controls, NC were assigned a value of 0, and late-infantile GM1 patients were assigned a value of 1 to test the effects of the late-infantile disease on the age predictions. For comparisons between juvenile GM1 patients and neurotypical controls, NC were assigned a value of 0, and juvenile GM1 patients were assigned a value of 1 to test the effects of the juvenile disease on the age predictions. For comparisons between juvenile GM1 patients and late-infantile GM1 patients, juvenile patients were assigned a value of 0, and late-infantile GM1 patients were assigned a value of 1 to test the effects of the disease subtype on the age predictions. The slope from the line between predicted brain age and chronological age was quantified for each of the four cohorts analyzed (NC, juvenile GM1, late-infantile GM1, and the entire GM1 cohort) to quantify brain aging in each cohort. The interaction between participant age and the cohort comparison variable was evaluated using a likelihood-ratio test, as shown in section C of the supplementary materials. *P*-values < 0.05 were considered significant after a Bonferroni correction for multiple comparisons (88 comparisons). An ordinary one-way analysis of variation (ANOVA) was performed using Graphpad Prism to compare differences in BSAGE between groups with a post-hoc Tukey test, and was not corrected for multiple comparisons based on the number of brain structures analyzed (version 10.1.0, GraphPad Software, Boston, MA). Spearman correlations were performed between predicted brain age and cross-sectional clinical metrics, and were performed in GraphPad Prism and were not corrected for multiple comparisons.

## Results

### GM1 vs NC

**Fig. 1:**
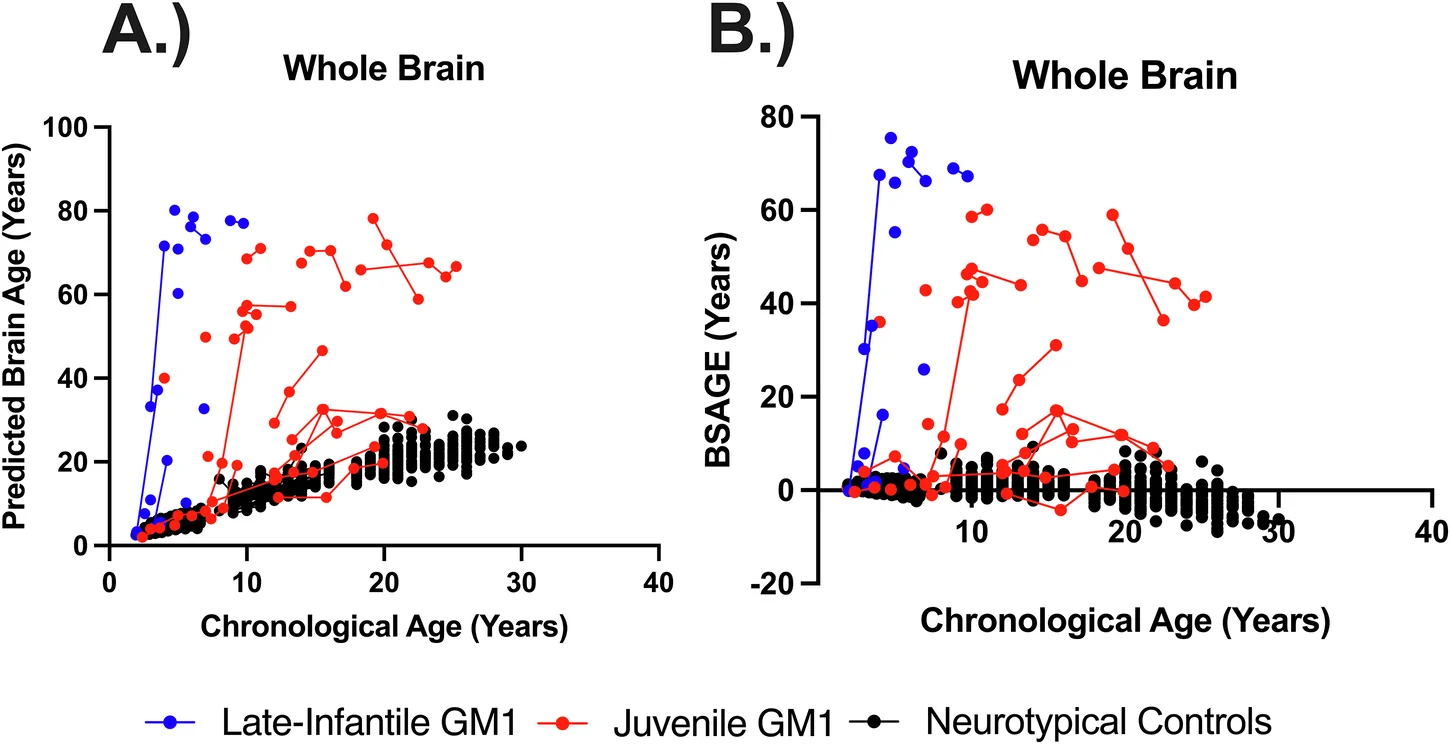
The longitudinal relationship between predicted whole brain age and whole brain, brain structures age gap estimation (BSAGE) with chronological age. Late-infantile (20 MRI scans from *n* = 15 patients) GM1 gangliosidosis patients are shown in blue, juvenile (61 MRI scans from *n* = 26 patients) GM1 gangliosidosis patients are shown in red, and neurotypical controls (897 MRI scans from *n* = 556 participants) are shown in black. Connecting lines indicate repeated scans on the same participant. Statistical analysis was performed using a linear mixed effects model to test the interactions between chronological age and cohort on predicted brain age, and was corrected for multiple comparisons using a Bonferroni correction. **A** The whole GM1 cohort (^2^(1) = 27.78, *p* < 0.0001), juvenile GM1 patients (^2^(1) = 67.32, *p* < 0.0001), and late-infantile GM1 patients (^2^(1) = 383.80, *p* < 0.0001) were found to have a statistically significant increasing effect on predicted whole brain age compared to neurotypical controls. Neurotypical controls showed a rate of brain aging at 0.83 years per chronological year compared to 1.57 years per chronological year in the GM1 cohort, 1.79 years per chronological year in the juvenile GM1 cohort, and 12.20 years per chronological year in the late-infantile cohort. There was also a significant interaction effect of the late-infantile disease (^2^(1) = 15.91, *p* < 0.0001) compared to the juvenile disease, where juvenile patients showed a rate of whole brain aging of 1.65 years per chronological year compared to 12.35 years per chronological year in late-infantile patients. The estimates and standard errors for the linear mixed effects modeling can be found in Supplementary Table 4. Longitudinal relationships between BSAGE and chronological age can be found in Section H of the Supplementary Materials (supplementary Fig. 28–48). **B** The relationship between BSAGE and chronological age in the three cohorts.

**Fig. 2:**
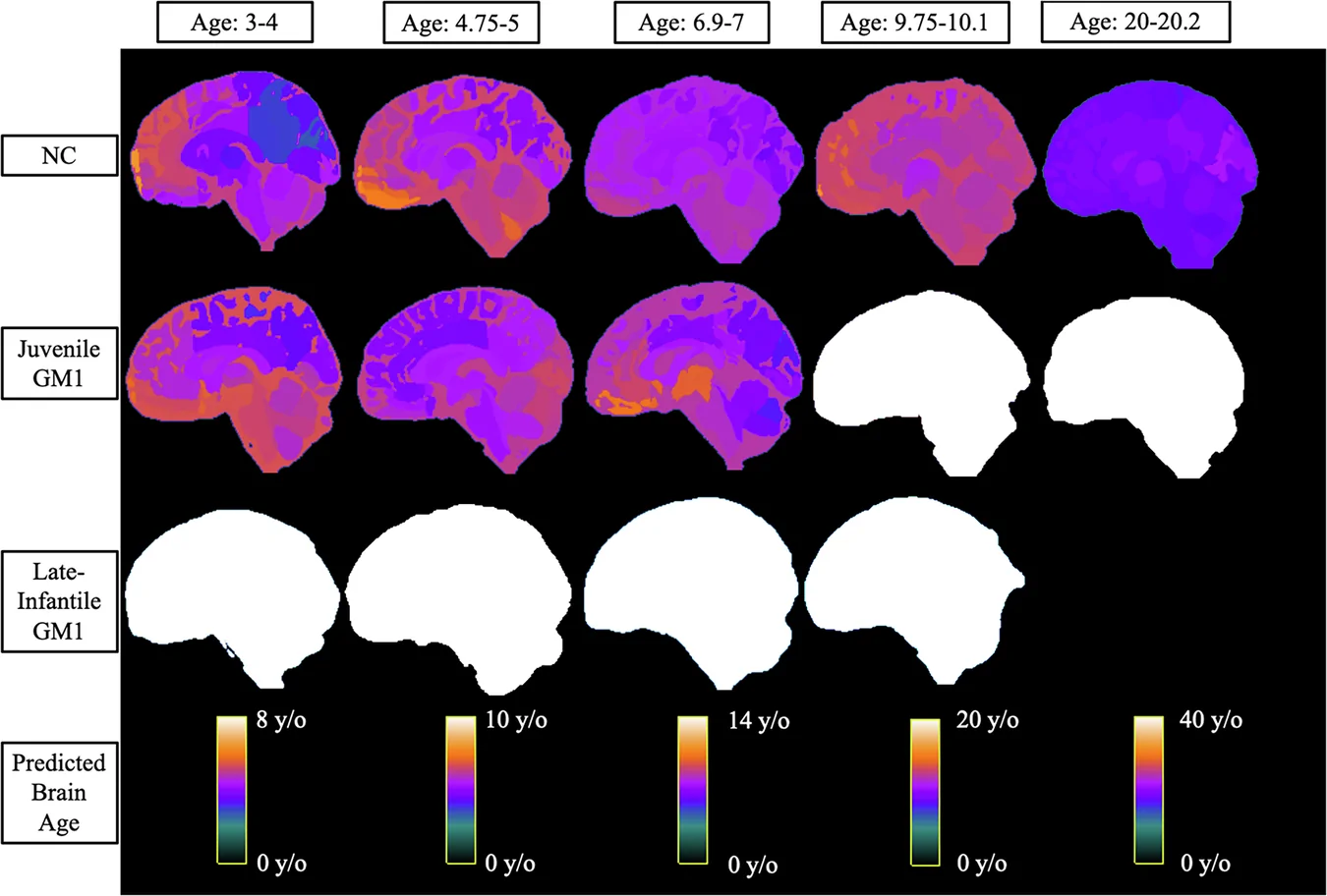
Sagittal view of brain structures, ages of the three cohorts at varying ages. Neurotypical controls (NC) are shown on the top row, juvenile GM1 gangliosidosis patients are shown in the middle row, and late-infantile GM1 gangliosidosis patients are shown in the bottom row. Colors were assigned according to the predicted age of that brain structure and were assigned between black (lowest predicted age) and white (highest predicted age) as shown in the scalar bar in the bottom right portion. The scalar ranges were adjusted for each column with min/max values of 0-8 years for the first column (3.0–4.0 years old), 0–10 years for the second column (4.75–5 years old), 0–14 years for the third column (6.9–7 years old), 0–20 years for the fourth column (9.75–10.1 years old), and 0–40 years for the fifth column (20–20.2 years old) as shown in the bottom row. No 20-year-old MRI scan was available for late-infantile patients, as the oldest late-infantile patient in this study was 9.75 years old at the time of the MRI evaluation. All MRI images were registered to the MNI coordinate space, and slices were taken at x = 1.0 (±1) mm for each participant. Axial and coronal views are available in the supplementary Material (supplementary Figs. 3, 4). A version with a full dynamic range are also available in the Supplementary Material (supplementary Figs. 5–7). Matching anatomical versions are also available in the Supplementary Material (supplementary Figs. 8–10).

In the comparisons between NC and the entire GM1 cohort, neurotypical controls showed an average slope of 0.83 years of predicted whole brain age per year of chronological age, as evaluated from the LMEM (Fig. 1). Predicted whole brain age was affected in two ways in GM1 gangliosidosis patients. First, there was a positive vertical shift in the y-intercept, with GM1 patients having a 18.57 ± 1.73-year higher predicted whole brain age starting at age 0 compared to controls (supplementary Fig. 16 and supplementary Table 4). Second, the interaction between chronological age and GM1 diagnosis showed further divergence with an average increase in predicted whole brain age of 0.74 ± 0.14 per year, resulting in an average predicted whole brain increase of 1.57 years per one year increase in chronological age compared to 0.83 in the neurotypical control cohort *X*^2^(1) = 27.78, *p*_*corrected*_ < 0.0001, Table 1, supplementary Fig. 16). Figure 2 illustrates accelerated brain aging in GM1 gangliosidosis patients; at age 10 both subtypes of GM1 demonstrate significant aging outside of the scalar range.

**Table 1:** Brain age predictions result from linear mixed effects modeling

Structure	*GM1 v NC*	*Juvenile v NC*	*Late-Infantile v NC*	*Juvenile v Late-Infantile*
*χ*^*2*^(1) | *p-value | p-value*_*corrected*_				
Whole Brain	27.78 | **1.4e−7 | 1.2e-5**	67.32 | **2.3e−16 | 2.0e-14**	383.80 | **1.8e−85 | 1.6e-83**	15.91 | **6.6e−5 | 0.0058**
White Matter	40.18 | **2.3e−10 | 2.0e-8**	73.69 | **9.1e−18 | 8.0e-16**	322.4 | **4.4e−72 | 3.9e-70**	12.14 | **0.00049 | 0.043**
3^rd^ Ventricle	22.29 | **2.3e−6 | 0.00020**	55.83 | **7.9e−14 | 7.0e-12**	236.90 | **1.9e−53 | 1.7e-51**	10.58 | 0.0011 | 0.1005
4^th^ Ventricle	13.70 | **0.00021 | 0.018**	33.56 | **6.9e−9 | 6.1e-7**	424.0 | **2.7e−94 | 2.4e-92**	27.84 | **6.5e−6 | 5.7e-4**
Inferior Lateral Ventricles	13.38 | **0.00026 | 0.023**	25.42 | **4.6e−7 | 4.0e-5**	311.1 | **1.3e−69 | 1.1e-67**	13.06 | **0.00030 | 0.026**
Lateral Ventricles	41.46 | **1.2e−10 | 1.1e-8**	85.71 | **2.1e−20 | 1.8e-18**	307.0 | **1.0e−68 | 8.8e-67**	11.25 | 0.00080 | 0.070
Ventral Diencephalon	19.85 | **8.38e−6 | 0.00074**	47.44 | **5.7e−12 | 5.0e−10**	414.1 | **4.6e−92 | 4.0e−90**	20.33 | **6.5e−6 | 5.7e−4**
External CSF	53.83 | **2.2e−13 | 1.9e-11**	88.25 | **6.0e−21 | 5.3e-19**	294.2 | **6.1e−66 | 5.4e-64**	10.98 | **0.00092 | 0.081**
Thalamus	22.03 | **2.7e−06 | 0.00024**	52.80 | **3.7e−13 | 3.3e-11**	335.5 | **6.1e−75 | 5.4e-73**	14.02 | **0.00018 | 0.016**
Caudate Nucleus	47.75 | **4.8e−12 | 4.2e-10**	79.53 | **4.7e−19 | 4.1e-17**	211.6 | **6.1e−48 |** < **0.0001**	8.15 | 0.0043 | 0.38
Putamen	35.30 | **2.8e−9 | 2.5e-7**	49.73 | **1.8e−12 | 1.6e-10**	139.6 | **3.3e−32 | 2.9e-30**	4.90 | 0.027 | 1.000
Hippocampus	17.31 | **3.2e−5 | 0.0028**	31.71 | **1.8e−8 | 1.6e−6**	392.3 | **2.6e−87 | 2.3e-85**	18.88 | **1.4e−5 | 0.0012**
Basal Forebrain	31.50 | **2.0e−8 1.8e-6**	62.40 | **2.8e-15 | 2.5e-13**	378.7 | **2.4e−84 | 2.1e-82**	16.55 | **4.7e−5 | 0.0041**
Cerebellar Gray Matter	11.17 | 0.00083 | 0.073	27.67 | **1.4e−7 | 1.2e−5**	375.8 | **1.0e−83 | 8.8e-82**	25.14 | **5.3e−7 | 4.7e-5**
Cerebellar White Matter	13.25 | **0.00027 | 0.024**	31.81 | **1.7e−8 | 1.5e−6**	395.8 | **4.5e−88 | 4.0e−86**	25.79 | **3.8e−7 | 3.3e−5**
Cerebellar Lobule I-V	19.80 | **8.6e−6 | 0.00076**	40.37 | **2.1e−10 | 1.8e-8**	412.6 | **1.7e−90 | 1.5e-88**	19.28 | **1.1e−5 | 0.00088**
Cerebellar Lobule VI-VII	15.58 | **7.9e−5 | 0.0070**	40.80 | **1.7e−10 | 1.5e-8**	358.9 | **4.8e−80 | 4.2e-78**	22.93 | **1.7e−6 | 0.00015**
Cerebellar Lobule VIII-X	12.25 | **0.00046 | 0.041**	29.7 | **5.0e−8 | 4.4e−6**	340.3 | **5.4e−76 | 4.8e−74**	20.30 | **6.6e−6 | 0.00058**

*p-*values < 0.05 after a Bonferroni correction (*p-value*_*corrected*_) for multiple comparisons were considered significant and bolded.

Estimates and Standard Errors from the LMEM for the interaction between participant age and the cohort comparison can be found in supplementary Table G1.

**Fig. 3:**
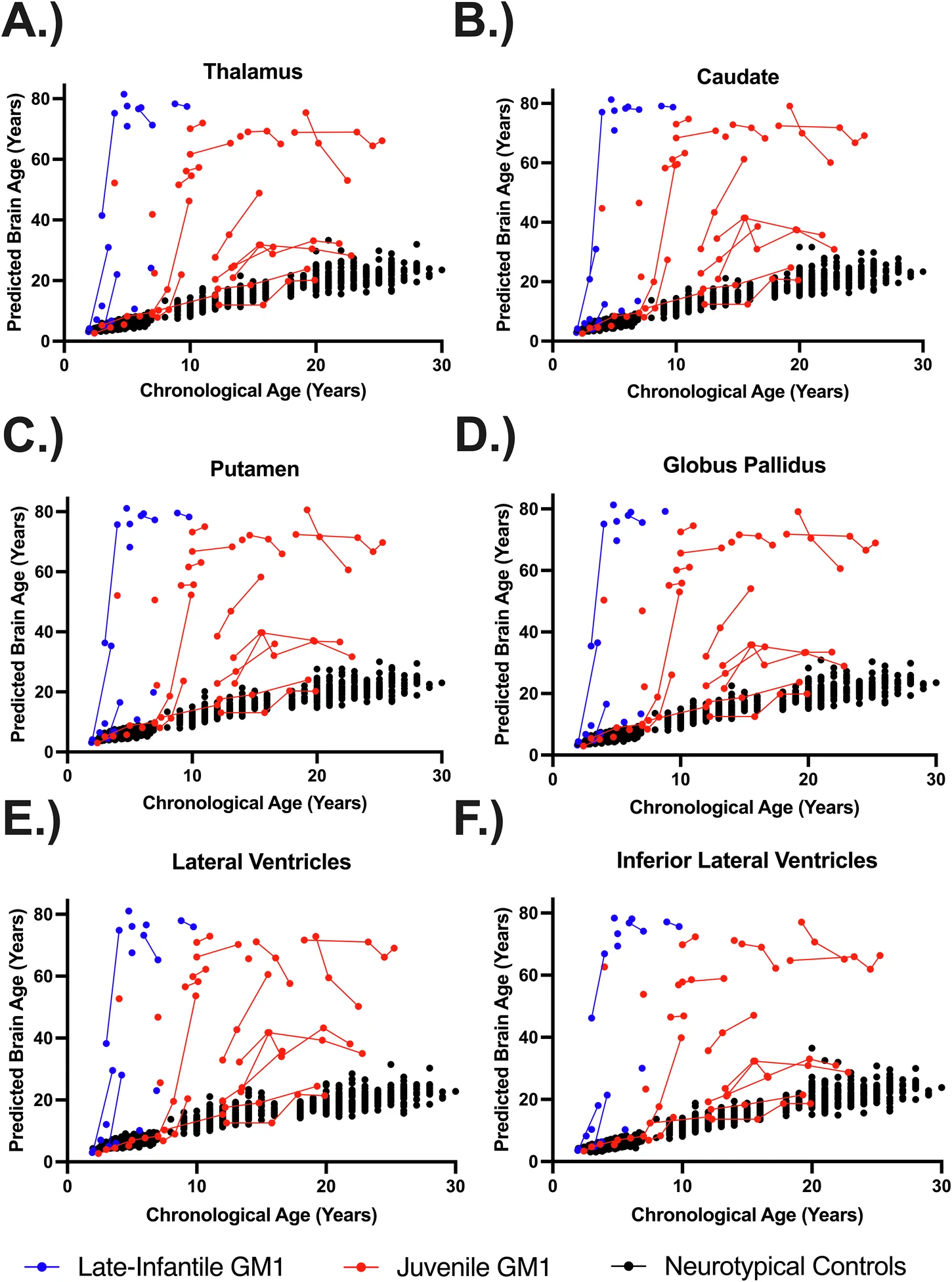
The relationship between predicted brain age and chronological age in midbrain and ventricle structures. Predicted brain ages for **A** thalamus, **B** caudate nucleus, **C** putamen, **D** globus pallidus, **E** lateral ventricles, and **F** inferior lateral ventricles are plotted against chronological age. Late-infantile (20 MRI scans from *n* = 15 patients) GM1 gangliosidosis patients are shown in blue, juvenile (61 MRI scans from *n* = 26 patients GM1 gangliosidosis patients are shown in red, and neurotypical controls (897 MRI scans from *n* = 556 participants) are shown in black. Connecting lines indicate repeated scans on the same participant. Statistical analysis was performed using a linear mixed effects model to test the interactions between chronological age and cohort on predicted brain age, and the results are summarized in Table I (complete results included in supplementary Table 3), with the estimated and standard errors shown in supplementary Table 4.

**Fig. 4:**
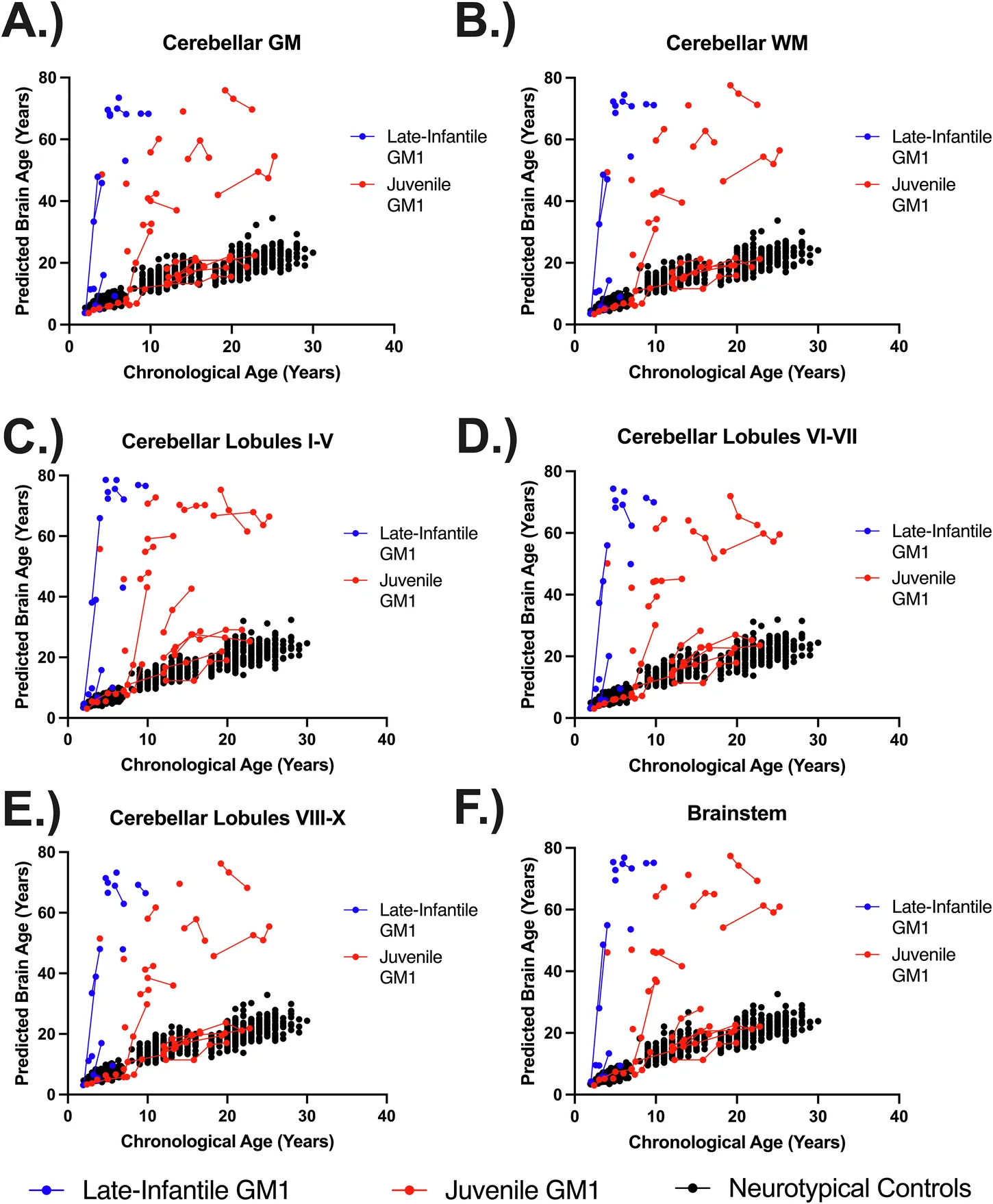
The relationship between predicted brain age and chronological age in hindbrain structures. Predicted brain ages for **A** cerebellar gray matter (GM), **B** cerebellar white matter (WM), **C** cerebellar lobules I-V, **D** cerebellar lobules VI-VII, **E** cerebellar lobules VIII-X, and **F** the brainstem are plotted against chronological age. Late-infantile (20 MRI scans from *n* = 15 patients) GM1 gangliosidosis patients are shown in blue, juvenile (61 MRI scans from *n* = 26 patients) GM1 gangliosidosis patients are shown in red, and neurotypical controls (897 MRI scans from *n* = 556 participants) are shown in black. Connecting lines indicate repeated scans on the same participant. Statistical analysis was performed using a linear mixed effects model to test the interactions between chronological age and cohort on predicted brain age and the results are summarized in Table I (complete results included in supplementary Table 3), with the estimated and standard errors shown in supplementary Table 4.

Similar differences between NC and GM1 patients in predicted whole brain age were observed for the predicted ages of the white matter, 3^rd^ and 4^th^ ventricles, inferior lateral ventricles, lateral ventricles (Fig. 3), external CSF, ventral diencephalon, thalamus, caudate nucleus, nucleus accumbens, putamen, globus pallidus, hippocampus, basal forebrain, amygdala, cerebellar gray and white matter (Fig. 4, supplementary Figs. 20–27), lobules I-V of the cerebellum, lobules VI and VII of the cerebellum, lobules VIII-X of the cerebellum, and the brainstem, as summarized in Table 1.

### Juvenile GM1 vs NC

In the comparisons between NC and the juvenile GM1 cohorts, predicted whole-brain age was also affected in two ways. First, there was a positive vertical shift in the y-intercept by which juvenile GM1 patients had a 10.31 ± 1.61-year higher predicted whole brain age starting at age 0 years on average compared to controls (supplementary Fig. 17 and supplementary Table 4). Second, the interaction between chronological age and juvenile GM1 diagnosis showed further divergence with an average increase in the slope between predicted whole brain age and chronological age of 0.96 ± 0.11 per year resulting in an average predicted whole brain increase of 1.79 years per one year increase in chronological age compared to 0.83 in the neurotypical control cohort (*X*^2^(1) = 67.32, *p*_*corrected*_ < 0.0001, Table 1, supplementary Fig. 17). Figure 2 illustrates the progressive accelerated brain aging in the juvenile GM1 gangliosidosis patients compared to NC.

Similar relationships between NC and juvenile GM1 patients in predicted whole brain age were observed for the predicted ages of the white matter, 3rd and 4th ventricles, inferior lateral ventricles, lateral ventricles, external CSF, ventral diencephalon, thalamus, caudate nucleus (Fig. 3), nucleus accumbens, putamen, globus pallidus, hippocampus, basal forebrain, amygdala, cerebellar gray and white matter, lobules I-V of the cerebellum, lobules VI and VII of the cerebellum (Fig. 4), lobules VIII-X of the cerebellum, and the brainstem, as summarized in Table 1.

### Late-infantile GM1 vs NC

In the comparisons between NC and late-infantile GM1 cohorts, the interaction between chronological age and late-infantile GM1 diagnosis showed substantial divergence with an average increase in the slope between predicted whole brain age and chronological age of 11.37 ± 0.52 per year resulting in an average predicted whole brain increase of 12.20 per one year increase in chronological age compared to 0.83 in the neurotypical control cohort (*X*^2^(1) = 383.30, *p*_*corrected*_ < 0.0001, Table 1, supplementary Fig. 18). There was a negative vertical shift in the y-intercept whereby late-infantile GM1 patients had a -21.29 ± 2.76-year lower predicted brain age starting at age 0 on average compared to controls. However, this effect was diminished due to the high slope (12.20) in the relationship between predicted brain age and chronological age in the late-infantile cohort. Figure 2 illustrates significantly accelerated brain aging in the late-infantile cohort compared to NC and juvenile patients at all ages. Similar relationships between NC and late-infantile GM1 patients in predicted whole brain age were observed for the predicted ages of the white matter, 3rd and 4th ventricles, inferior lateral ventricles, lateral ventricles, external CSF, ventral diencephalon, thalamus, caudate nucleus, nucleus accumbens, putamen, globus pallidus, hippocampus (Fig. 3), basal forebrain, amygdala, cerebellar gray and white matter, lobules I-V of the cerebellum, lobules VI and VII of the cerebellum, lobules VIII-X of the cerebellum (Fig. 4), and the brainstem, as summarized in Table 1 (and further summarized in supplementary Data 2 and 3).

### Juvenile GM1 vs late-infantile GM1

In the comparisons between juvenile GM1 and late-infantile GM1 cohorts, the interaction between chronological age and late-infantile GM1 diagnosis (compared to juvenile GM1) showed substantial divergence with an average increase in the slope between predicted whole brain age and chronological age of 10.70 ± 2.63 per year resulting in an average predicted whole brain increase of 12.35 per one year increase in chronological age compared to 1.65 in the juvenile GM1 cohort (*X*^2^(1) = 15.91, *p*_*corrected*_ < 0.0001, Table 1, supplementary Fig. 19). There was a negative vertical shift in the y-intercept whereby late-infantile GM1 patients had a −34.12 ± 15.04-year lower predicted brain age starting at age 0 on average compared to juvenile GM1 patients. However, this effect was diminished due to the high slope (12.20) in the relationship between predicted brain age and chronological age in the late-infantile cohort. Similar relationships between juvenile and late-infantile GM1 patients in predicted whole brain age were observed for the predicted ages of the white matter, 3rd and 4th ventricles, inferior lateral ventricles, lateral ventricles, external CSF, ventral diencephalon, thalamus, caudate nucleus, nucleus accumbens, putamen, globus pallidus, hippocampus, basal forebrain, amygdala, cerebellar gray and white matter, lobules I-V of the cerebellum, lobules VI and VII of the cerebellum, lobules VIII-X of the cerebellum (Figs. 3, 4), and the brainstem, as summarized in Table 1. There was also a descriptive increase in the proportion of late-infantile GM1 participants’ baseline and longitudinal scans, whole brain predicted age above normative, compared to juvenile participants (supplementary Tables 5 and 6).

### Brain structures age gap estimation (BSAGE)

**Fig. 5:**
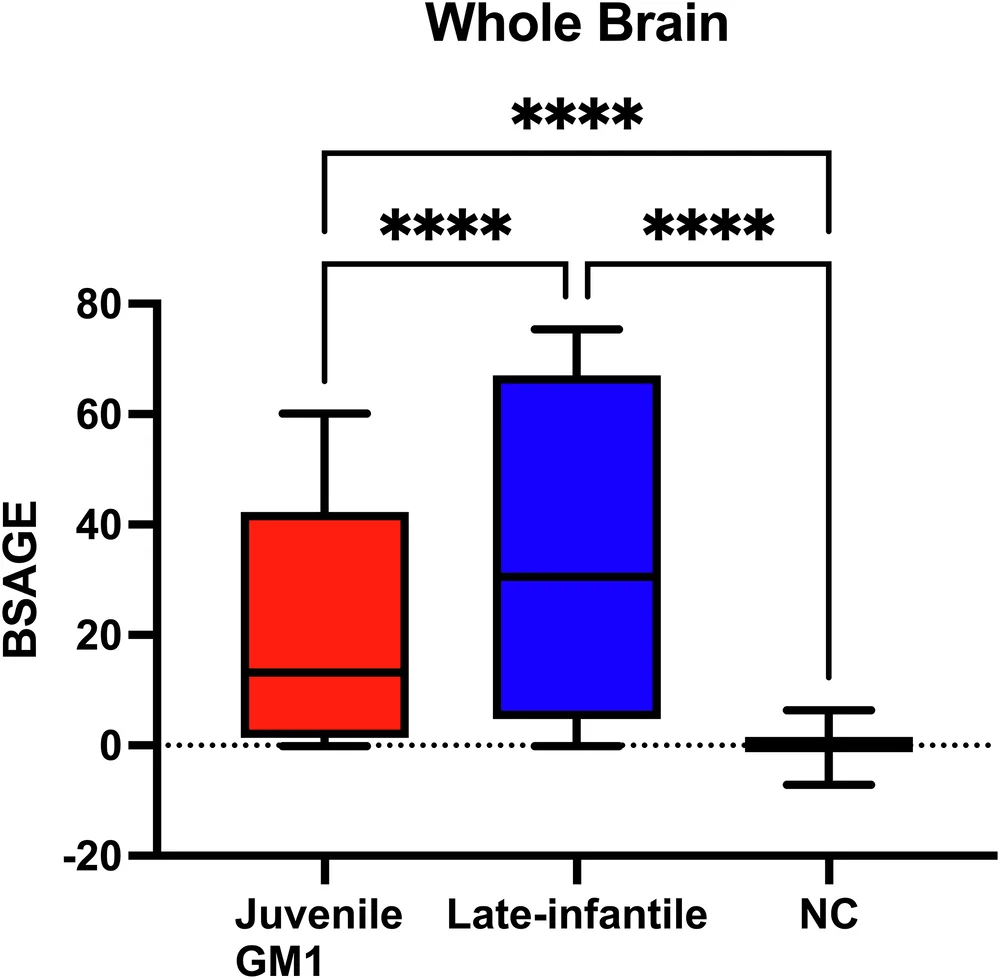
Comparison of Whole Brain BSAGE between Juvenile GM1 patients (*n* = 26), lateinfantile GM1 patients (*n* = 15), and neurotypical controls (*n* = 556). In all box and whisker plots, the center line represents the median, box limits represent quartiles, and error bars extend to the 1st and 99th percentiles. Late-infantile GM1 patients had an average BSAGE = 35.50, juvenile GM1 patients had an average BSAGE = 21.19, and NC participants had an average BSAGE = 0.03. An ordinary one-way analysis of variance (ANOVA) showed statistically significant (F(2, 595) = 671.5, *p* = 2.7e−153) differences in BSAGE between the cohorts. A post hoc Tukey test showed the late-infantile cohort had a statistically significantly higher BSAGE compared to both the neurotypical controls (*p* = 4.79e−10) and juvenile GM1 patients (*p* = 6.81e−10). The juvenile GM1 cohort also had a significantly higher BSAGE compared to the neurotypical controls (*p* = 4.79e−10). Cohort comparisons between late-infantile GM1, juvenile GM1, and NC for BSAGE for the other brain structures can be found in section I of the Supplementary Materials. There were no adjustments made for multiple comparisons of BSAGE based on the number of brain structures analyzed.

Late-infantile GM1 patients had an average cross-sectional whole brain BSAGE = 35.50, juvenile GM1 patients had an average BSAGE = 21.19, and NC participants had an average BSAGE = 0.03 (Fig. 5) at the time of most recent MRI scan. An analysis of variance (ANOVA) showed statistically significant (F(2,594) = 671.5, *p* < 0.0001) differences in BSAGE between the cohorts. A post-hoc Tukey test showed that the late-infantile cohort had a statistically significantly higher BSAGE compared to both the neurotypical controls (*p* < 0.0001) and juvenile GM1 patients (*p* < 0.0001). The juvenile GM1 cohort also had a significantly higher BSAGE compared to the neurotypical controls (*p* < 0.0001).

Similar relationships between NC, juvenile GM1 patients, and late-infantile patients in whole brain BSAGE were observed for the BSAGE of the white matter, 3^rd^, 4^th^ ventricle, inferior lateral, and lateral ventricles, external CSF, ventral diencephalon, thalamus, caudate nucleus, nucleus accumbens, putamen, globus pallidus, hippocampus, basal forebrain, amygdala, cerebellar gray and white matter, lobules I-V of the cerebellum, lobules VI and VII of the cerebellum, lobules VIII-X of the cerebellum, and the brainstem, as described in Section I of the supplementary Material (supplementary Fig. 49–69).

Longitudinal evaluations of BSAGE (Fig. 1B) showed that NC control BSAGE remained close to the x-axis or a 0 value for BSAGE despite increases in chronological age. Juvenile GM1 patients, however, demonstrated BSAGE increases commensurate with an increase in chronological age. Furthermore, late-infantile GM1 patients demonstrated an increased slope in the relationship between BSAGE and chronological age compared to both NC and juvenile GM1 gangliosidosis patients (Fig. 1B, supplementary Figs. 28–48).

Comparison of Whole Brain BSAGE among the three NC participants (supplementary Fig. 15), showed that the Calgary participants had an average BSAGE = 0.63, the QTAB participants had an average BSAGE = 1.48, and the QTIM participants had an average BSAGE = −0.93. An analysis of variance (ANOVA) showed statistically significant (F(2553) = 43.53, *p* < 0.0001) differences in BSAGE between the three NC cohorts. A post-hoc Tukey test showed that the QTAB cohort had a statistically significantly higher BSAGE compared to both Calgary (*p* = 0.009) and QTIM (*p* < 0.0001). The Calgary cohort also had a significantly higher BSAGE compared to QTIM (*p* < 0.0001). Comparison of Whole Brain predicted brain age and chronological age by the NC cohort is shown in Supplementary Fig. 14.

### Clinical correlations

**Fig. 6:**
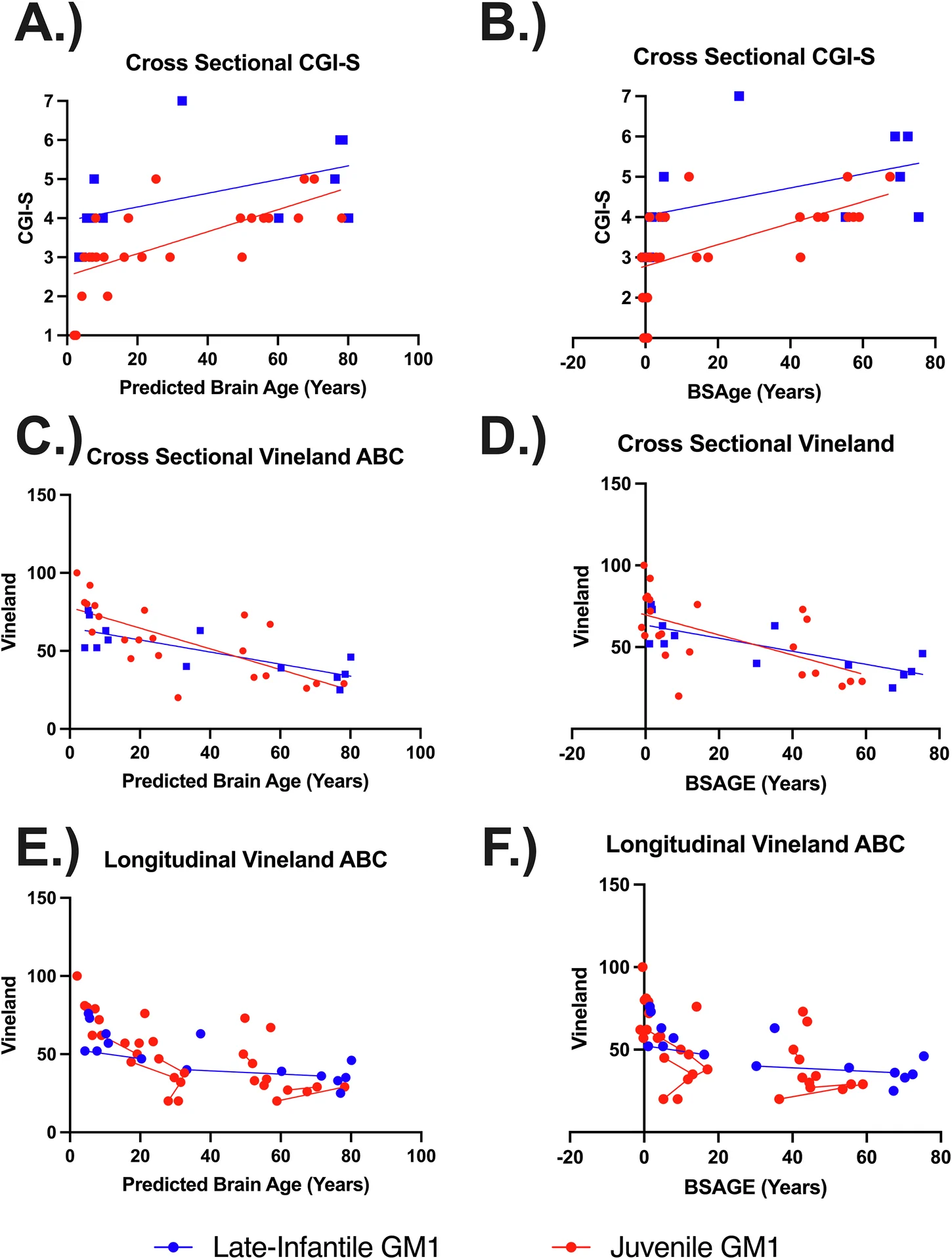
The relationship between clinical outcome assessments and both predicted whole brain age and whole brain BSAGE for late-infantile (blue) and juvenile (red) GM1 patients. BSAGE is equivalent to the patient’s predicted brain age minus their chronological age. Spearman correlations were performed between predicted brain age and cross-sectional clinical metrics (**A**–**D**). Linear mixed effects modeling were performed between predicted brain age and longitudinal clinical metrics (**E**, **F**). Relationships between clinical outcome assessments and brain ages were exploratory and were not corrected for multiple comparisons. **A** Predicted brain age correlated with CGI-S in both the late-infantile (ρ(10) = 0.63; 95% CI [0.06, 0.89], *p* = 0.03) and juvenile (ρ(23) = 0.76; 95% CI [0.51, 0.89], *p* < 0.01) GM1 patients. **B** Brain structures age gap estimation (BSAGE) also correlated with CGI-S in both the late-infantile (ρ(10) = 0.64, *p* = 0.03) and juvenile (ρ(23) = 0.74, *p* < 0.01) GM1 patients. **C** Predicted brain age correlated with cross-sectional Vineland scores in both the late-infantile (ρ(11) = -0.72 95% CI [-0.91, -0.27], *p* < 0.01) and juvenile (ρ(20) = -0.78; 95% CI [-0.90, -0.52], *p* < 0.01) GM1 patients. **D** BSAGE also correlated with cross-sectional Vineland scores in both the late-infantile (ρ(11) = −0.73; 95% CI [−0.92, −0.29], *p* < 0.01) and juvenile (ρ(20) = −0.66; 95% CI [−0.85, −0.31], *p* < 0.01) GM1 patients. **E** Predicted brain age correlated with longitudinal Vineland scores in both the late-infantile (*X*^2^(1) = 12.71, R^2^ = 0.57, *p* < 0.01) and juvenile (*X*^2^(1) = 12.11, R^2^ = 0.39, *p* < 0.01) GM1 patients. **F** BSAGE also correlated with longitudinal Vineland scores in both the late-infantile (*X*^2^(1) = 12.15, R^2^ = 0.55, *p* < 0.01) and juvenile (*X*^2^(1) = 4.22, R^2^ = 0.16, *p* = 0.04) GM1 patients.

Both the predicted whole brain (ρ(10) = 0.63, *p* = 0.03) and brain structures age gap estimation (BSAGE, ρ(10) = 0.64, *p* = 0.03) positively correlated with baseline CGI-S in late-infantile GM1 gangliosidosis patients (Fig. 6A, 6B). Predicted whole brain (ρ(23) = 0.76, *p* < 0.01) and brain structures age gap estimation (BSAGE, ρ(23) = 0.74, *p* < 0.01) also both positively correlated with baseline CGI-S in juvenile GM1 gangliosidosis patients (Fig. 6A, 6B).

Predicted whole brain age (ρ(11) = -0.72, *p* < 0.01) and BSAGE (ρ(11) = -0.73, *p* < 0.01) both negatively correlated with cross-sectional Vineland adaptive behavioral composite standard scores in late-infantile GM1 patients. Predicted whole brain age (ρ(20) = -0.78, *p* < 0.01) and BSAGE (ρ(20) = -0.66, *p* < 0.01) also negatively correlated with cross-sectional Vineland adaptive behavioral composite standard scores in juvenile GM1 gangliosidosis patients (Fig. 6C, D).

Predicted brain age correlated with longitudinal Vineland scores in both the late-infantile

(*X*^2^(1)=12.71, R^2^ = 0.57, *p* < 0.01) and juvenile (*X*^2^(1) = 12.11, R^2^ = 0.39, *p* < 0.01) GM1 patients. BSAGE also correlated with longitudinal Vineland scores in both the lateinfantile (*X*^2^(1) = 12.15, R^2^ = 0.55, *p* < 0.01) and juvenile (*X*^2^(1) = 4.22, R^2^ = 0.16, *p* = 0.04) GM1 patients (Fig. 6E, 6F).

### Correlations with Brain Volumetrics

Predicted whole brain age was correlated with whole brain volume (cm^3^) in both juvenile (R^2^ = 0.57) and late-infantile (R^2^ = 0.46) GM1 gangliosidosis patients (supplementary Fig. 70).

Predicted whole brain age was weakly correlated with whole brain volume (cm^3^) in the NC (R^2^ = 0.02, supplementary Fig. 74). Predicted whole brain age was also correlated with whole brain volume controlled for ICV in both the juvenile (R^2^ = 0.77) and late-infantile (R^2^ = 0.51) GM1 gangliosidosis patients (supplementary Fig. 71). Predicted whole brain age was also weakly correlated with whole brain volume controlled for ICV in the NC (R^2^ = 0.07, supplementary Fig. 75). Correlations between predicted whole brain age and both total brain volume and total brain volume controlled for ICV by NC cohort are shown in supplementary Fig. 78, 79, 82, 83, 86, and 87.

Whole brain BSAGE was correlated with whole brain volume (cm^3^) in both the juvenile (R^2^ = 0.49) and late-infantile (R^2^ = 0.43) GM1 gangliosidosis patients (supplementary Fig. 72). Whole brain BSAGE was not correlated with whole brain volume (cm^3^) in NC participants (supplementary Fig. 76). Whole brain BSAGE was correlated with whole brain volume controlled for ICV in both the juvenile (R^2^ = 0.69) and late-infantile (R^2^ = 0.47) GM1 gangliosidosis patients (supplementary Fig. 73). Whole brain BSAGE was weakly correlated with whole brain volume controlled for ICV in the NC (R^2^ = 0.08, supplementary Fig. 77). Correlations between predicted whole BSAGE and both total brain volume and total brain volume controlled for ICV by NC cohort are shown in supplementary Fig. 80, 81, 84, 85, 88, and 89.

## Discussion

In this study, we quantified brain aging in the largest cohort of type II GM1 gangliosidosis patients (81 total T1-weighted scans from 41 patients) alongside 556 neurotypical controls (897 total T1-weighted scans) of the same age range. We found that GM1 patients and NC have distinct brain aging trajectories; GM1 patients, on average, showed whole brain aging at approximately twice the rate of their unaffected peers (1.57 compared to 0.83), reflecting neurodegenerative changes from disease progression. Furthermore, when examining the type II GM1 gangliosidosis subtypes, we found that the juvenile GM1 patients’ whole brain aged at a rate similar to that of the whole GM1 cohort, but the late-infantile patients aged at nearly eight times that rate (12.35, Fig. 1), indicating a divergence in neurodegeneration between the disease subtypes. Furthermore, qualitative evaluations demonstrated a similar trend

(supplementary Tables 5, 6). As shown in supplementary Fig. 11–13, increases in both whole brain predicted brain age and BSAGE are reflective of volumetric changes known to be associated with GM1 progression, including cerebral atrophy, lateral ventricle enlargement, corpus callosum atrophy, cerebellar atrophy, and demylinetation^10–14^.

This relationship accurately depicts the neurologic clinical picture of the two subtypes of GM1 gangliosidosis; late-infantile patients have seizures, speech difficulties, and mobility difficulties earlier and more frequently than their juvenile GM1 counterparts. Late-infantile patients also die significantly before their juvenile counterparts, with survival typically until early in the second decade of life, compared to juvenile patients who can survive into the fourth decade of life^9^. Similar relationships to the accelerated aging indicating neurodegeneration was observed for the whole brain were observed for localized structures including cerebellar structures including cerebellar gray and white matter, cerebellar lobules I-V, cerebellar lobules VI and VII, cerebellar lobules VIII-X, and the brainstem (Fig. 4), midbrain structures including the thalamus, caudate nucleus, putamen, and globus pallidus (Fig. 3), and cerebrospinal fluid (CSF) structures including the 3^rd^ ventricle, 4^th^ ventricle, lateral ventricle, inferior lateral ventricle, and external CSF, reflecting the widespread neurodegeneration in Type II GM1 patients. This is consistent with previous volumetric and MRI studies in Type II GM1 gangliosidosis patients that showed atrophy of the cerebrum, cerebellum, corpus callosum, thalamus, caudate nucleus, lentiform nucleus (including the globus pallidus and putamen), associated ventricle enlargement, as well as white matter effects^10–13,18^. Furthermore, in a neuropathological investigation of a postmortem juvenile GM1 gangliosidosis patient, neuronal loss was found throughout the basal ganglia (including the caudate, putamen, and globus pallidus), gray matter of the cerebellum, amygdala, thalamus, neocortex, limbic system, pons, and spinal cord^41^. Gliosis was also observed in these structures and the white matter of the cerebellum^41^. In total, these findings are consistent with the regions identified with accelerated brain aging, suggesting that accelerated brain aging may be associated with gliosis, neuronal loss, or some combination.

Previous investigations into brain aging through brain age predications have demonstrated various neurotypical development findings, including increased brain development during puberty and the capability of predicting mortality^42,43^. Regarding evaluations of the brain age gap or BSAGE as referred to in this study, previous studies have shown that a positive brain age gap is common in neurodegenerative diseases, indicating diagnostic potential^42^. Furthermore, Nguyen et al^30^. utilizing the same *BrainStructuresAges* pipeline used in the present study, found the hippocampus to be the most accelerated aging region in Alzheimer’s patients, the temporal and frontal lobes to have accelerated aging in frontotemporal dementia patients, the thalamus and cerebral gray matter in multiple sclerosis, the prefrontal and medial temporal lobe in schizophrenia patients, and a mostly neurotypical brain in Parkinson’s patients. These results highlight the capability and specificity of the model.

Deviations from neurotypical development in either direction (either underdeveloped or premature development) have been related to worse outcomes. For instance, in toddlers, increased BSAGE has been shown to be associated with worse self-regulation, and decreases in BSAGE have been associated with worse cognition^44^. Furthermore, in a study leveraging data from the adolescent brain cognitive development (ABCD) study, they found increases in T1-weighted derived BSAGE to be associated with socioeconomic disadvantage and neighborhood safety^45^. Decreased BSAGE has also been connected to attention-deficit/hyperactivity disorder (ADHD) and depression in adolescents^46^. Cross-sectional BSAGE evaluations from the most recent MRI scan also revealed significant differences between late-infantile and juvenile GM1 gangliosidosis patients. Despite the late-infantile patients being younger than the juvenile patients on average, they showed significantly more aging (Fig. 5). Cross-sectional BSAGE evaluations also demonstrated whole brain aging differences among the three NC cohorts (supplementary Fig. 15). However, all three NC cohorts were within two years of true chronological age which was minor compared to either the juvenile (21.19) or late-infantile (35.50) GM1 patients (Fig. 5) which showed significant deviations from neurotypical development. Furthermore, in a few juvenile patients who were scanned either early in their disease course or did not have significant GM1 progression, BSAGE was reflectively low, further substantiating the accuracy and utility of the model (supplementary Fig. 13).

Correlations between whole-brain volumetrics and predicted whole-brain age were noteworthy (Section J of the supplementary Materials). First, the predicted whole brain age and BSAGE in NC were at most weakly correlated with whole brain volume. This finding suggests that *BrainStructuresAges* is not solely a univariate function of volume and is potentially a more robust neuroimaging-derived aging parameter. Consideration of localized volumes, white and gray matter density, or cortical thickness and surface area in combination with brain volume may be more indicative of brain aging due to the voxel-level approach^47–49^. Second, in both juvenile and late-infantile patients, predicted whole brain age and BSAGE were correlated with brain volume. This finding indicates that accelerated brain aging is a product of volumetric loss or atrophy in GM1 patients. However, since these findings were not perfectly correlated, brain aging in GM1 gangliosidosis was also not observed to be a univariate function of volume, further suggesting that brain age predictions are more robust than one-dimensional volumetric measurements.

Correlations with clinical outcome assessments (Fig. 6), including the Vineland Adaptive Behavioral Composite (ABC) and clinical global impression (CGI) scale, further indicate the importance of brain aging in this cohort. Increased brain aging was associated with worsening scores in both categories, reflecting both patients’ global clinical presentation and adaptive behavior. This evaluation of brain aging in GM1 suggests it may be an important neuroimaging marker of disease progression. Additionally, improvements or reversal of the brain aging metrics or decreased BSAGE in this cohort resulting from therapeutic intervention, including gene therapy, may be reflective of morphological and therapeutic benefit^50,51^. Future studies are needed to validate the clinical significance of brain aging and the role of brain age prediction in the evaluation of therapies in this cohort.

Limitations of this methodology need to be considered before this model is used in clinical practice, clinical trials, and other research projects assessing neurodegeneration. First, there were variations in scanning protocols between GM1 patients and each of the neurotypical control datasets. GM1 patients, Calgary, and QTAB participants were all scanned on a 3 T system, however, of different manufacturers (Phillips, General Electric, and Siemens, respectively). QTIM participants were scanned on a 4T Bruker system. Furthermore, the GM1 patients were sedated during their lengthy MRI acquisition protocol, including magnetic resonance spectroscopy (MRS), diffusion tensor imaging (DTI), and functional magnetic resonance imaging (fMRI) collection. Since the present study was focused solely on structural MRI, propofol-related effects were likely negligible, but there was likely increased motion in the NC cohorts comparatively. Ultimately, the resolution for GM1 patients (1 mm^3^), Calgary NC (resampled to 0.182 mm^3^), QTAB NC (0.512 mm^3^), and QTIM NC (0.729 mm^3^) were all 1 mm^3^ or better, which is sufficient for volumetric analysis^52^. Second, while the whole GM1 cohort was of sufficient sample size (*n* = 41), there was not an even distribution of late-infantile (*n* = 15) and juvenile (*n* = 26) patients. Moreover, longitudinal MRI evaluations showed an even further skew, as there were a total of 20 MRI scans in the late-infantile cohort and 61 in the juvenile cohort. This ultimately reflects both the rarity of this disease and the short window between diagnosis and death, which occurs early in the second decade of life in late-infantile patients^7,9^. Despite this challenge, future studies on Type II GM1 gangliosidosis should focus on gathering data from more patients or employ alternative techniques of boosting the sample size to achieve results that increasingly reflect the disease. Third, the present study is dependent on a single deep learning U-Net convolution neural network, to which future studies should replicate the present results with alternative models and training data. Similarly, the accuracy of these models in analyzing data from pediatric neurodegenerative diseases requires further investigation. Future studies investigating brain aging in GM1 gangliosidosis should explore multimodal MRI data, including fMRI and DTI, and potentially MRS in evaluations of brain aging^49^.

In summary, we quantified brain aging in Type II GM1 gangliosidosis patients in relation to neurotypical controls of the same age range. Brain aging was quantified using a machine learning analysis pipeline capable of predicting brain age in numerous structures throughout the brain. We found widespread accelerated brain aging in GM1 patients compared to neurotypical controls in structures throughout the brain, reflecting neurodegenerative disease progression. Late-infantile patients were also found to have accelerated aging compared to juvenile GM1 patients, accurately depicting the clinical phenotypes of these patients. We also correlated brain aging with clinical outcome assessments to bolster predicted age as a candidate neuroimaging surrogate outcome assessment.

## Supplementary information

Transparent Peer Review file

Supplemental Material

Description of Additional Supplementary Files

Supplementary Data 1

Supplemental Data 2

Supplemental Data 3

## Data Availability

The source data for Figs. 1, 3–6 are in Supplementary Data 1. The source data for Fig. 2 is included in section D of the supplementary material. Neuroimaging data for the Calgary Preschool neurotypical control group are publicly available here: https://osf.io/axz5r/.^34,35^. Neuroimaging data for the Queensland Twin Adolescent Brain (QTAB) is publicly available here: https://openneuro.org/datasets/ds004146/versions/1.0.4.^36,37^. Neuroimaging data for the Queensland Twin IMaging (QTIM) dataset is publicly available here: https://openneuro.org/datasets/ds004169/versions/1.0.6.^38,39^.
