# Supplementary material for: Brain aging in Type II GM1 gangliosidosis
Source: Commun Med (Lond). 2026 Jul 6;6:568. doi: 10.1038/s43856-026-01745-w (PMC13720018; doi:10.1038/s43856-026-01745-w)
Supplement: Supplementary file 1 [file 43856_2026_1745_MOESM1_ESM.pdf]

nature portfolio

Peer Review File

Brain Aging in Type II GM1 Gangliosidosis

Corresponding Author: Dr Maria Acosta

This file contains all reviewer reports in order by version, followed by all author rebuttals in order by version.

Version 0:

Reviewer comments:

Reviewer #1

(Remarks to the Author)
The authors calculated a predicted brain age and Brain Structures Age Gap Estimation from 41 Type II GM1 gangliosidosis patients and 556 healthy controls utilizing a machine learning analysis pipeline.
They demonstrated in GM1 patients accelerated and distinct brain aging compared to controls throughout midbrain, hindbrain structures, brainstem, and the ventricles thus suggesting both the predicted brain age and BSAGE as a surrogate neuroimaging outcome measure in GM1 gangliosidosis.
The manuscript is clearly written and shows original results deriving from the use of an already known machine learning analysis approach in a relatively huge number of patients and controls.
Regarding the specific brain areas differently involved in the disease, it would be useful to add neuropathological data supporting the manuscript findings.

Reviewer #2

(Remarks to the Author)
This manuscript presents a well-executed and novel analysis of brain aging in patients with Type II GM1 gangliosidosis using a machine learning pipeline for MRI-based brain age prediction (BrainStructuresAges). However, the writing is poor. It is hard to follow, I might doubt its impact. Therefore, I suggest reject unless the author provide a meticulous revision.
Main comments:
1. The logic of writing of introduction need to be improved. First, this study focus on type II GM1, I think it is unnecessary to mention a lot about type I GM1. Second, it is unclear why we want to use MRI-based predicted brain age in assessing the neuronal degeneration, why not gray matter area, thickness. What is advantage or motivation using brain age. You claim that "These findings could serve as imaging outcome parameters for demonstrating therapeutic efficacy attendant to interventions such as gene therapy". Is the cohort from random placebo control trails with treatment? How the finding can serve for demonstrating therapeutic efficacy.
2. For the first sentence in result 'In the comparisons between NC and the entire GM1 cohort, neurotypical controls showed an average increase in predicted whole brain age of 0.83 years per one year increase in biological age with no statistical effect of sex ($\chi 2(1) = 1.76$, $p = 0.19$).', you should simply mention what is the aim, and what you did. It is hard to understand how you get the result "predicted whole brain age of 0.83 years per one year increase in biological age". What model you used to get predicted brain age, linear mixed effect model (LME)? Additionally, another question is that how you train the LME model. Do you only fit the model with NC subjects, otherwise, it is hard for me to understand why the patient predict age deviated so larger compared to the NC, if you use patient and NC to fit the model. If you train using NC, you should mention this simply in one sentence before the description of result. Second thinking, even you apply NC-trained model to the patient, typically the test scatter should not have so huge deviation. Do you normalize the MRI data for the patient using mean, std from NC? Are patients and NC from same scanner location in same parameters?
3. Based on some papers, they used GAMLSS (https://www.nature.com/articles/s41586-022-04554-y#Sec9), CNN (https://www.nature.com/articles/s43587-022-00219-7) to predict brain age, do you think the results can be varying if using different models. If so are your results stable and reliable?
4. For the figure 2, it is confused. First, what the color represented? What this sentence "Colors were assigned according to the predicted age of that brain structure" mean? Do you visualize the beta weight for the LME in predicting age? If you only used the NC to train LME, how to obtain the patients' brain mapping? I do not know how you visualize the brain map, so the normalization of each column might not make sense. What is the range value in specific number? The number can help me understand why the late infantile GM1 are totally white.
5 For all the statistical analysis, do you implement multiple test correction?

Reviewer #3

(Remarks to the Author)
This study presents a novel application of machine learning-derived brain age prediction methods to a rare neurodegenerative disorder, Type II GM1 gangliosidosis. The authors analyzed 81 T1-weighted MRI scans from 41 patients and 897 scans from 556 neurotypical controls using the BrainStructuresAges pipeline. The work attempts to provide neuroimaging biomarkers for future clinical trials. The manuscript is generally well-written, and the findings are of potential value. However, several major conceptual and methodological issues must be addressed.
1. Conceptual Challenges in Interpreting Brain Age in Severe Neurodevelopmental Disorders like Type II GM1 Gangliosidosis
The most fundamental issue concerns the conceptual appropriateness of applying brain age prediction models to disorders such as Type II GM1 gangliosidosis, which are characterized by severe neurodevelopmental disruption and not merely a deviation along a typical trajectory of brain maturation or aging. Previous studies applying brain age prediction to pediatric conditions such as autism spectrum disorder (ASD), attention-deficit/hyperactivity disorder (ADHD), or childhood epilepsy have generally highlighted modest delays in brain development. These patients, although developmentally atypical, still follow a broadly preserved trajectory of age-related neurodevelopment. This typically results in negative predicted age differences (PAD), suggesting developmental delay rather than deviation from the underlying structure of neurodevelopmental processes.
Similarly, in studies of adult neuropsychiatric or neurodegenerative conditions (e.g., schizophrenia, Alzheimer's disease), accelerated brain aging has been inferred from a positive PAD. These conditions are interpreted as reflecting premature aging along a continuum of normal age-related decline, and therefore are meaningfully situated within models of normative aging trajectories.
In contrast, patients with Type II GM1 gangliosidosis, particularly those with the late-infantile subtype, exhibit brain structures that yield predicted ages of 60 to 80 years in children as young as three. This profound discrepancy challenges the interpretability of PAD as a simple biomarker of either delayed development or accelerated aging. These brain morphologies do not reflect a mere shift along the typical developmental or degenerative trajectory but rather a fundamental deviation from it. The structural and maturational abnormalities in these patients are likely outside the range that the model was trained to recognize as age-appropriate at any point in the lifespan.
Nevertheless, I acknowledge the potential value of brain age models in identifying individuals whose neurodevelopment is drastically abnormal. We therefore recommend reframing the application of brain age prediction in this context—from a focus on quantitative estimates of age acceleration or delay, to a categorical identification of trajectory deviation. For example, the model could be leveraged to detect patients whose predicted whole-brain age or BSAGE values fall outside the 95% prediction interval (confidence band) derived from the neurotypical control group at matched chronological ages. These patients may be labeled as "off-trajectory," in contrast to "on-trajectory" patients whose predicted brain age remains within normative expectations.
This stratification into on-trajectory and off-trajectory groups has multiple potential benefits. First, it aligns better with the biological reality of GM1 gangliosidosis, in which developmental pathways are fundamentally derailed. Second, it could facilitate more clinically meaningful subgroup analyses. For example, as the authors suggest that LI patients may be more severely affected than juvenile (Juv) patients, one could hypothesize that LI patients are more likely to fall into the off-trajectory group. This hypothesis can be formally tested using a chi-square test.
Moreover, after stratification based on brain trajectory deviation, traditional statistical approaches can be applied to examine group differences in clinical severity scores, cognitive-behavioral outcomes, or longitudinal progression. Two-sample t-tests or nonparametric equivalents could be employed to compare Vineland scores, Clinical Global Impression (CGI), or other relevant variables between off- and on-trajectory patients. This approach could preserve the valuable biological insights derived from brain age modeling, while avoiding overinterpretation of uninterpretable continuous outputs (e.g., assigning 70-year-old brain ages to toddlers).
This approach may offer more clinically meaningful information. For instance, identifying whether a patient is on or off the normative developmental trajectory could inform treatment prioritization or therapeutic expectations. Patients who remain within normative bounds may benefit from aggressive rehabilitation and supportive therapies aimed at preserving functional development. In contrast, those who fall significantly outside the trajectory may require alternative clinical strategies, potentially including palliative or highly individualized interventions. This stratification, grounded in deviation from normative developmental expectations rather than presumed "aging," may offer more accurate and clinically actionable insights for this patient population. The authors are experts in this area and could have done a better job in this discussion.

2 Clarification of Brain Age Prediction Averaging and Regions Used
The manuscript references use of the BrainStructuresAges method (PMID: 38224546), which produces predictions for over 100 brain structures. Yet, the main text and methods list only a subset. It is unclear whether the whole-brain predicted age was computed from the average of all available structures or just from a selected few. Figure 2 suggests the authors used a more comprehensive set. Clarification is needed regarding exactly which regions were included in the averaging to derive whole-brain age, and whether the weights were uniform.

3 Model Calibration and Use of Support Vector Regression
The cited method (PMID: 38224546) used support vector regression to model the relationship between chronological and predicted age. In this manuscript, the authors compute the average predicted age directly from structural outputs without such a correction. This results in a control group slope of 0.83 (i.e., predicted age increases less than 1 year per biological

year), which may suggest suboptimal calibration. If the authors aim to use quantitative values of predicted age and BSAGE (e.g., 12.2 years/year in LI patients), then calibrating the model for slope ~1 in neurotypical controls might be necessary. Alternatively, if the focus is only on trajectory deviation (see point 1), recalibration may not be necessary.

4 Colormap Saturation in Brain Renderings (Figure 2 and Supplement D)
The authors appear to have deliberately limited the colormap scale, resulting in highly saturated ("white") renderings in LI and older Juv patients. This visually supports the claim of extreme predicted age deviation but prevents readers from forming independent impressions of gradient and severity. I recommend relaxing the colormap bounds to allow more nuanced visual comparison across cohorts and brain structures, or alternatively, explicitly provide a supplemental version with a full dynamic range.

5 Redundancy in Displayed Results Across Brain Structures
Figures summarizing BSAGE or predicted age across dozens of structures, though comprehensive, yield little novel insight beyond what is seen in whole-brain summaries. Most regional findings closely mirror whole-brain results and are not separately discussed in detail. I suggest reducing these to highlight only the most discriminative or clinically relevant regions (e.g., thalamus, caudate, cerebellum), with full results moved to supplementary material.

6 Confusing and Inconsistent Language in Reporting Predicted Age Differences
The manuscript contains several instances in which the terminology used to describe predicted brain age differences is confusing or internally inconsistent. For example, in the comparison between juvenile and late-infantile (LI) GM1 patients, the authors report that “LI GM1 patients had a -34.12 ± 15.04-year lower predicted brain age on average compared to Juv GM1 patients” (line 149), while also noting that Juv patients had a “10.31 ± 1.61-year higher predicted whole brain age on average compared to controls” (line 112). Elsewhere, they describe GM1 patients having “18.57 ± 1.73-year higher predicted brain age” than controls (line 95).
This language becomes problematic because in all cases the observed effect is essentially the same: a higher predicted brain age in GM1 patients relative to controls or subtypes. Yet the inconsistent use of negative versus positive descriptors (e.g., “lower predicted age” vs. “higher predicted age”) and ambiguous sentence structure makes interpretation unnecessarily difficult.

7 Clarification of Sample Sizes Across Datasets
Authors need to state the number of subject number of each dataset in the method. The authors state in Supplement Figure E1: "n = 556, 897 total MRI scans". Easily misleading, when I first read it I thought you had 6 figures of subjects.

8. Please define BSAGE at its first occurrence in the abstract and main text (currently only defined in Methods).

Reviewer #4

(Remarks to the Author)
General Comment
The authors conducted brain age estimation using BrainStructuresAges on MRI scans of patients with GM1 gangliosidosis. Compared to healthy controls, the predicted brain age of the patients was estimated to be older than their chronological age, and the gap between predicted and chronological age was associated with disease severity. This suggests that brain age estimation could serve as a quantitative evaluation method for the brains of patients with GM1 gangliosidosis.

Major Comments
1. It would be helpful to include a description of how the annual brain aging rate was calculated. Some patients underwent multiple MRI scans: it is unclear whether this was taken into account.
2. The Results section contains noticeable redundancies. Similar sentences are repeated with only slight changes in numerical values. In particular, the repetition of content around lines 103–108 stands out. Using tables to summarize such data could improve readability and conciseness.
3. The manuscript states that the average predicted brain age in late-infantile GM1 (LI GM1) patients is −21.29 ± 2.76 years compared to controls (lines 132–133). However, the predicted brain age in LI GM1 patients should be higher than that of controls, suggesting this may be an error. A similar issue appears in line 150.
4. Since the estimated age and BASAGE are similar metrics, their content seems redundant. Given that BASAGE, which is corrected for the age at the time of imaging, is likely the more important indicator, it might be preferable to reduce the emphasis on the results for the estimated age itself.
5. There is considerable variability in BASAGE among GM1 patients—some show very high values, while others are close to zero. Please discuss possible reasons for this variability. For example, are there any distinctive imaging features that might explain these differences?
6. What kinds of brain changes are reflected by a high BASAGE value? Although there is some discussion of the relationship between brain volume and predicted age, it may not be sufficient. As your study also provides region-wise results, further discussion from that perspective would be valuable.
7. Given the high variability in predicted brain age among patients, it may be difficult to use this as an individual clinical marker despite group-level differences. Furthermore, due to the limited longitudinal data, there is insufficient evidence to demonstrate that predicted age correlates with disease progression. Therefore, referring to it as a “neuroimaging surrogate clinical outcome assessment” may be an overstatement.

Minor Comments

1. Please standardize the terminology used for disease names. For example, expressions like “late-infantile GM1 gangliosidosis,” “L1 GM1,” “late-infantile GM1,” and “late-infantile” are used inconsistently. The same applies to “juvenile GM1.”
2. Unify the usage of either “biological” or “chronological” when referring to participants' actual ages.
3. In line 97, “the interaction between biological age and GM1” may be intended as “the interaction between biological and estimated age in GM1.” Please check similar phrases repeated later as well.
4. “Figure 7” (line 200) should be corrected to “Figure 7A and 7B.”
5. “Figure 2B” in lines 181 and 186 should be corrected to “Figure 2.”
6. “Figure 3” in line 709 should be corrected to “Figure 5.”

Version 1:

Reviewer comments:

Reviewer #1

(Remarks to the Author)
The authors have made improvements to their manuscript based on the questions previously raised.

Reviewer #2

(Remarks to the Author)
I read the response letter for me part. But I still hold some questions:
1. In response to my previous comment 1, you stated: “This advanced aging correlated with clinical outcomes (Vineland and CGI scores), so that, if the gene therapy were to slow the rapid brain aging, it would suggest therapeutic benefit.” However, the notion that gene therapy may slow brain aging appears to be a hypothesis, and your current results do not provide direct evidence to support it. Nor do you cite any external references substantiating this assumption. Therefore, I still believe the statement claiming that your findings “demonstrate therapeutic efficacy attendant to interventions such as gene therapy” is too strong. At least, you should elaborate on this logic in the discussion section and cite relevant literature that supports this proposed link.
Additionally, you stated: “Since the brain age prediction model was trained using deep learning it is plausible that this type of analysis is more robust than a simple thickness, surface area, or volumetric measurement.”, I also hold different opinions. I have engineering background in computer vision and medical imaging processing. In general, with sample size less than 10 thousand, deep learning sometimes can not outperform machine learning. I know there are lots of studies using deep learning as well as machine learning to estimate brain aging. About the model choosing, this is a disputed topic. But, coming back to your case, at least, you should add one more sentence with referring paper to support the advantage of deep-learning model in section of MRI Processing.

2. In response to my previous comment 3, I know they extracted low-level image features like cortical features. However, GAM can be used on raw MRI image, you can just vectorize all voxels in 2d image array as vector to train a model. Since you only mentioned you used deep learning model, I have no idea what type of deep learning you are using, transformer, BERT, CNN. Even for CNN, there are many of different typical models including ResNet, DenseNet, Unet. Ideally, you should make a basic control analysis to see whether using other deep learning model, the statistical analyses between brain aging and some cognitive/clinical test index was same. Otherwise, adding short discussion in the limitation to admit we did not try other models, it is interesting to see how the results of other models look like in future. Tbh, since I have one or two projects which also focus on brain aging (more than thousands of subjects), if the downstream statistical analyses to clinical measurements did not produce very smaller p values ($p<0.0001$), the results using different model might change. This is why I think at least, you should say something about this into limitations.
If all those questions have be further addressed, I agree this paper can be accepted.

Reviewer #3

(Remarks to the Author)
The authors have substantially improved the manuscript in this revision. The writing is clearer, and the study design and results are now more logically presented. The proposed MRI-derived brain age metrics offer potentially important insights into Type II GM1 gangliosidosis. However, several key conceptual issues remain unresolved, particularly regarding the interpretation and applicability of “brain age gap” in the context of severe pediatric neurodevelopmental disorders.
1. Interpretability of Brain Age in Severe Neurodevelopmental Disorders Remains Problematic
The authors have not yet adequately addressed the core conceptual concern raised in the first round: whether assigning predicted brain ages of 60–80 years to 3–5-year-old patients with late-infantile GM1 gangliosidosis is biologically meaningful. Such extreme positive Brain Structure Age Gaps (BSAGE) suggest a fundamental deviation from normative brain development—not a simple acceleration along a typical aging trajectory.
While the authors argue that nearly all GM1 patients fall outside the 95% confidence interval of the normative model, making an “on/off-trajectory” classification impractical, this does not fully resolve the concern. I recommend that the authors present the proportion of patients whose predicted brain ages exceed 1, 2, or 3 standard deviations above age-matched neurotypical controls. This could provide clinically meaningful gradation of disease severity, especially in early-stage patients.
More importantly, the manuscript should be revised to clarify the interpretive limits of the brain age concept in this context. For example, rather than stating that late-infantile GM1 patients have “brains of a 70-year-old,” it would be more accurate to

emphasize that their MRI features deviate substantially from normal pediatric development and lie outside the model's trained age range. In such cases, predicted age should be interpreted as an index of disease burden or neurodegenerative change, not as literal evidence of premature aging.
The authors implicitly acknowledge this in noting that most GM1 patients lie "outside the scalar range" of the model. They now need to make this explicit in the text. This clarification is crucial to prevent misuse or misinterpretation of the brain age framework. It will also aid clinicians and readers in understanding that, for instance, a +35-year BSAGE in a 5-year-old child signals severely atypical brain structure, not that the child is biologically elderly. Such reframing would both improve conceptual rigor and enhance clinical utility.

2. Terminology Related to "Surrogate Outcome" Should Be More Cautious
The authors present additional analyses showing correlations between brain age metrics and clinical measures (CGI, Vineland), supporting their clinical relevance. However, the evidence remains insufficient to justify calling these metrics "surrogate neuroimaging outcomes." True surrogate outcomes must reliably reflect clinical endpoints in the context of treatment. At present, the study has not demonstrated whether therapeutic interventions (e.g., gene therapy) can alter the trajectory of predicted brain aging. The authors themselves acknowledge that the clinical significance of these findings remains to be validated.
Accordingly, I recommend revising such terminology throughout the manuscript. Referring to predicted brain age as a "candidate imaging biomarker" or "potential outcome measure" would better reflect the current state of evidence and highlight the need for future validation.

Reviewer #4

(Remarks to the Author)
The authors responded sincerely and accurately to all of my comments. In particular, their handling of my major comments 3, 5, and 6 has made the paper clearer and more insightful. I have no other concerns.

Version 3:

Reviewer comments:

Reviewer #2

(Remarks to the Author)
I think all of my comments have been solved. Now the paper is more clearer and insightful. I am ok for their publication in ComMed journal.

Reviewer #3

(Remarks to the Author)
The authors have made improvements to their manuscript based on the questions previously raised.

Open Access This Peer Review File is licensed under a Creative Commons Attribution 4.0 International License, which permits use, sharing, adaptation, distribution and reproduction in any medium or format, as long as you give appropriate credit to the original author(s) and the source, provide a link to the Creative Commons license, and indicate if changes were made.
In cases where reviewers are anonymous, credit should be given to 'Anonymous Referee' and the source.
The images or other third party material in this Peer Review File are included in the article's Creative Commons license, unless indicated otherwise in a credit line to the material. If material is not included in the article's Creative Commons license and your intended use is not permitted by statutory regulation or exceeds the permitted use, you will need to obtain permission directly from the copyright holder.
To view a copy of this license, visit https://creativecommons.org/licenses/by/4.0/

Reviewer #1 (Remarks to the Author):

The authors calculated a predicted brain age and Brain Structures Age Gap Estimation from 41 Type II GM1 gangliosidosis patients and 556 healthy controls utilizing a machine learning analysis pipeline.
They demonstrated in GM1 patients accelerated and distinct brain aging compared to controls throughout midbrain, hindbrain structures, brainstem, and the ventricles thus suggesting both the predicted brain age and BSAGE as a surrogate neuroimaging outcome measure in GM1 gangliosidosis.
The manuscript is clearly written and shows original results deriving from the use of an already known machine learning analysis approach in a relatively huge number of patients and controls. Regarding the specific brain areas differently involved in the disease, it would be useful to add neuropathological data supporting the manuscript findings.

We appreciate Reviewer 1's time and consideration of our work. With reference to the neuropathology comment, we did not obtain any postmortem brain from the patients described in this cohort. However, PMID: 32219895 describes the neuropathology of a juvenile Type II GM1 gangliosidosis patient. Hence, we have incorporated the following language in the discussion of our manuscript (lines 382-388). "Furthermore, in a neuropathological investigation of a postmortem juvenile GM1 gangliosidosis patient, neuronal loss was found throughout the basal ganglia (including the caudate, putamen, and globus pallidus), gray matter of the cerebellum, amygdala, thalamus, neocortex, limbic system, pons, and spinal cord. Gliosis was also observed in these structures and in the white matter of the cerebellum. In total, these findings are consistent with the regions identified with accelerated brain aging, suggesting that accelerated brain aging may be associated with gliosis, neuronal loss, or some combination."

Reviewer #2 (Remarks to the Author):

This manuscript presents a well-executed and novel analysis of brain aging in patients with Type II GM1 gangliosidosis using a machine learning pipeline for MRI-based brain age prediction (BrainStructuresAges). However, the writing is poor. It is hard to follow, I might doubt its impact. Therefore, I suggest reject unless the author provide a meticulous revision.

Main comments:

1. The logic of writing of introduction need to be improved. First, this study focus on type II GM1, I think it is unnecessary to mention a lot about type I GM1. Second, it is unclear why we want to use MRI-based predicted brain age in assessing the neuronal degeneration, why not gray matter area, thickness. What is advantage or motivation using brain age. You claim that "These findings could serve as imaging outcome parameters for demonstrating therapeutic efficacy attendant to interventions such as gene therapy". Is the cohort from random placebo control trails with treatment? How the finding can serve for demonstrating therapeutic efficacy.

We agree there was too much discussion of infantile GM1 in the introduction and have rephrased this portion of the introduction to include only the following language (lines 53-54): "Type I (Infantile) GM1 gangliosidosis is the most severe, with death occurring by the age of three."

Regarding whether the cohort was from a randomized placebo-controlled trial, it was not; the dataset was from a natural history study of GM1 gangliosidosis with no intervention. The referenced gene therapy clinical trial is also not a randomized placebo control trial. Ethical considerations have rejected the use of these types of trials due to the rapid deterioration of GM1 patients; this trial design is common in rare disease research. For example, Lenmeldy (Metachromatic Leukodystrophy: PMID: 27289174) and Zolgensma (Spinal Cerebellar Ataxia: PMID: 34536405), both received FDA approval utilizing data from historical controls due to the same ethical concerns of withholding a viable treatment in a degenerative disease.

As for demonstrating therapeutic efficacy, the natural history of brain aging in GM1 gangliosidosis, as described in this manuscript shows significantly accelerated brain aging, particularly in the late-infantile patients. This advanced aging correlated with clinical outcomes (Vineland and CGI scores) so that, if the gene therapy were to slow the rapid brain aging, it would suggest therapeutic benefit. Furthermore, the motivation for utilizing this type of analysis in GM1 is that it is a novel marker of brain deterioration in this cohort. Since the brain age prediction model was trained using deep learning it is plausible that this type of analysis is more robust than a simple thickness, surface area, or volumetric measurement.

2. For the first sentence in result ‘In the comparisons between NC and the entire GM1 cohort, neurotypical controls showed an average increase in predicted whole brain age of 0.83 years per one year increase in biological age with no statistical effect of sex ($\chi 2(1) = 1.76$, $p = 0.19$).’, you should simply mention what is the aim, and what you did. It is hard to understand how you get the result “predicted whole brain age of 0.83 years per one year increase in biological age”. What model you used to get predicted brain age, linear mixed effect model (LME)? Additionally, another question is that how you train the LME model. Do you only fit the model with NC subjects, otherwise, it is hard for me to understand why the patient predict age deviated so larger compared to the NC, if you use patient and NC to fit the model. If you train using NC, you should mention this simply in one sentence before the description of result. Second thinking, even you apply NC-trained model to the patient, typically the test scatter should not have so huge deviation. Do you normalize the MRI data for the patient using mean, std from NC? Are patients and NC from same scanner location in same parameters?

We appreciate reviewer 2’s comments towards improving the clarity of the manuscript. We have removed the latter part of the designated sentence (referring to no statistical effect of sex) to improve clarity. We also added the following language to the end of that sentence to clarify how that metric was calculated: “as evaluated from the linear mixed effects model” (line 202-203).

Predicted brain age was calculated using Volbrain’s BrainStructuresAges pipeline which outputs predicted brain ages for the structures analyzed in this work. Predicted brain ages were then statistically modeled using linear mixed effects modeling with the outputted data from Volbrain’s BrainStructuresAges. The linear mixed effects modeling quantified the average change in predicted brain age based on the values from BrainStructuresAges for each of the cohorts analyzed in this study. The model was fit with both NC and patients (Model 1 shown below, Supplement C). However, included in the model is the interaction between Age and Cohort (NC versus patient), where NC were assigned a value of 0 and GM1 patients were assigned a value of 1. So, removing the effect of GM1 gives the averages for the NC cohort and vise-versa. The result of an increase of 0.83 years per one year increase in biological year was derived from the estimate of the effect of Age in the LMEM as shown in Supplement Table G1.

Model 1:

model1 <- lmer(Predicted Brain Age ~ Age * Cohort + Sex + (1|Participant), dataset)

summary(lmer)

Predicted Brain Age – Corresponds to the predicted brain age determined by *BrainStructuresAges* for the structure being analyzed
Cohort – Corresponds to the comparison between cohorts as presented in Table 1.
Sex – Corresponds to the participants’ biological sex

Age – Corresponds to the participants' chronological age
Participant – Each participant was given a distinct number to account for repeated measures (subject level random intercept).

The plots shown in Figures 1, 3, 4, and 5 have huge scatter as a result of the GM1 disease. This phenomenon has been shown in neurologic and neurodegenerative diseases that are less severe than GM1 so the increased deviation from normal is to be expected and consistent with the clinical presentation of GM1.

The goal of this study was to quantify brain aging in GM1 patients in relation to NC, so there was no normalization between GM1 and NC data.

Data from patients are not from the same scanner or session as data from NC. This was mentioned as a limitation of this study, but we believe that the variance attributable to inter-scanner variability is largely mitigated by the high resolution (1 mm^3 or better) scans utilized for each of the cohorts.

3. Based on some papers, they used GAMLSS (https://www.nature.com/articles/s41586-022-04554-y#Sec9), CNN (https://www.nature.com/articles/s43587-022-00219-7) to predict brain age, do you think the results can be varying if using different models. If so are your results stable and reliable?

The stability and reliability of the methods utilized in this manuscript are outlined in PMID: 38224546, which compared the brain age prediction model utilized in this study with other models, demonstrating improved mean absolute error and R^2 compared to other methods. The first study mentioned calculated percentiles from normative volumetric and cortical thickness measurements, which while related, is a fundamentally different approach to the methods utilized in our study. The second study mentioned utilized a CNN to calculate brain aging which is the same as the CNN utilized in the creation of the model we used (PMID: 38224546). Both studies showed increases in BSAGE for both Alzheimer's disease and frontotemporal dementia which suggest our results would be similar if we utilized a different model. It is worth mentioning, their study did not include any pediatric data which suggests the model utilized in our study would be both more stable and reliable for the primarily pediatric normative and GM1 data of this study.

4. For the figure 2, it is confused. First, what the color represented? What this sentence "Colors were assigned according to the predicted age of that brain structure" mean? Do you visualize the beta weight for the LME in predicting age? If you only used the NC to train LME, how to obtain the patients' brain mapping? I do not know how you visualize the brain map, so the normalization of each column might not make sense. What is the range value in specific number? The number can help me understand why the late infantile GM1 are totally white.

The color is a representative map of the predicted brain age on the individualized structure. The *BrainStructuresAges* (https://volbrain.net/services/BrainStructureAges) pipeline utilized in this manuscript outputs the predicted brain ages overlaid on their specific structure. For example, the predicted brain age for the cerebellum (and cerebellar lobules) is shown as a color corresponding to the color bar in all images.

The specific number for the scalar range varied depending on the column, which is why we did not supply a specific number in the figure itself. However, the scalar ranges were included in the figure caption, "The scalar ranges were adjusted for each column with min/max values of 0-8 years for the first column (3.0-4.0 years old), 0-10 years for the second column (4.75-5 years old), 0-14 years for the third column (6.9-7 years old), 0-20 years for the fourth column (9.75-10.1 years old), and 0-40 years for the fifth column (20-20.2 years old)." The late-infantile GM1 patients' age map was above and outside of the range at all time points and resulted in the white color highlighting the advanced aging in that cohort. In the juvenile cohort, there was some evidence of advanced aging early on as evident by the orange color, and more significant aging later in the disease course as evident by the color in the older two age maps. We have updated figure 2 to incorporate labeled scalar bars for each column for clarity.

5 For all the statistical analysis, do you implement multiple test correction?

We implemented a Bonferroni correction for multiple comparisons and have updated the statistical analysis section of the methods to improve clarity (lines 193). "*P*-values < 0.05 were considered significant after a Bonferroni correction for multiple comparisons (88 comparisons)."

Reviewer #3 (Remarks to the Author):

This study presents a novel application of machine learning-derived brain age prediction methods to a rare neurodegenerative disorder, Type II GM1 gangliosidosis. The authors analyzed 81 T1-weighted MRI scans from 41 patients and 897 scans from 556 neurotypical controls using the BrainStructuresAges pipeline. The work attempts to provide neuroimaging biomarkers for future clinical trials. The manuscript is generally well-written, and the findings are of potential value. However, several major conceptual and methodological issues must be addressed.
1. Conceptual Challenges in Interpreting Brain Age in Severe Neurodevelopmental Disorders like Type II GM1 Gangliosidosis
The most fundamental issue concerns the conceptual appropriateness of applying brain age prediction models to disorders such as Type II GM1 gangliosidosis, which are characterized by severe neurodevelopmental disruption and not merely a deviation along a typical trajectory of brain maturation or aging. Previous studies applying brain age prediction to pediatric conditions

such as autism spectrum disorder (ASD), attention-deficit/hyperactivity disorder (ADHD), or childhood epilepsy have generally highlighted modest delays in brain development. These patients, although developmentally atypical, still follow a broadly preserved trajectory of age-related neurodevelopment. This typically results in negative predicted age differences (PAD), suggesting developmental delay rather than deviation from the underlying structure of neurodevelopmental processes.

Similarly, in studies of adult neuropsychiatric or neurodegenerative conditions (e.g., schizophrenia, Alzheimer's disease), accelerated brain aging has been inferred from a positive PAD. These conditions are interpreted as reflecting premature aging along a continuum of normal age-related decline, and therefore are meaningfully situated within models of normative aging trajectories.

In contrast, patients with Type II GM1 gangliosidosis, particularly those with the late-infantile subtype, exhibit brain structures that yield predicted ages of 60 to 80 years in children as young as three. This profound discrepancy challenges the interpretability of PAD as a simple biomarker of either delayed development or accelerated aging. These brain morphologies do not reflect a mere shift along the typical developmental or degenerative trajectory but rather a fundamental deviation from it. The structural and maturational abnormalities in these patients are likely outside the range that the model was trained to recognize as age-appropriate at any point in the lifespan.

Nevertheless, I acknowledge the potential value of brain age models in identifying individuals whose neurodevelopment is drastically abnormal. We therefore recommend reframing the application of brain age prediction in this context—from a focus on quantitative estimates of age acceleration or delay, to a categorical identification of trajectory deviation. For example, the model could be leveraged to detect patients whose predicted whole-brain age or BSAGE values fall outside the 95% prediction interval (confidence band) derived from the neurotypical control group at matched chronological ages. These patients may be labeled as "off-trajectory," in contrast to "on-trajectory" patients whose predicted brain age remains within normative expectations.

This stratification into on-trajectory and off-trajectory groups has multiple potential benefits. First, it aligns better with the biological reality of GM1 gangliosidosis, in which developmental pathways are fundamentally derailed. Second, it could facilitate more clinically meaningful subgroup analyses. For example, as the authors suggest that LI patients may be more severely affected than juvenile (Juv) patients, one could hypothesize that LI patients are more likely to fall into the off-trajectory group. This hypothesis can be formally tested using a chi-square test. Moreover, after stratification based on brain trajectory deviation, traditional statistical approaches can be applied to examine group differences in clinical severity scores, cognitive-behavioral outcomes, or longitudinal progression. Two-sample t-tests or nonparametric equivalents could be employed to compare Vineland scores, Clinical Global Impression (CGI), or other relevant variables between off- and on-trajectory patients. This approach could preserve the valuable biological insights derived from brain age modeling, while avoiding

overinterpretation of uninterpretable continuous outputs (e.g., assigning 70-year-old brain ages to toddlers).

This approach may offer more clinically meaningful information. For instance, identifying whether a patient is on or off the normative developmental trajectory could inform treatment prioritization or therapeutic expectations. Patients who remain within normative bounds may benefit from aggressive rehabilitation and supportive therapies aimed at preserving functional development. In contrast, those who fall significantly outside the trajectory may require alternative clinical strategies, potentially including palliative or highly individualized interventions. This stratification, grounded in deviation from normative developmental expectations rather than presumed "aging," may offer more accurate and clinically actionable insights for this patient population. The authors are experts in this area and could have done a better job in this discussion.

Thank you for your careful consideration and review of our manuscript. We understand the desire to stratify these patients into on and off trajectory in terms of brain aging for an individualized approach. However, in terms of putting this into practice, the 95% confidence intervals in Supplement Figure F1 show that virtually all GM1 patients (apart from a couple) would be categorized into the off trajectory. This would hinder the comparisons between clinical outcome assessments and predicted brain age as there would only be a couple in the on-trajectory category. Furthermore, categorizing them this way would remove the degree in which they are off trajectory. For instance, whether or not a patient is early in their disease course and just moved into the off trajectory versus a patient who is very severe in their disease course. This is captured in the current analysis.

2 Clarification of Brain Age Prediction Averaging and Regions Used

The manuscript references use of the BrainStructuresAges method (PMID: 38224546), which produces predictions for over 100 brain structures. Yet, the main text and methods list only a subset. It is unclear whether the whole-brain predicted age was computed from the average of all available structures or just from a selected few. Figure 2 suggests the authors used a more comprehensive set. Clarification is needed regarding exactly which regions were included in the averaging to derive whole-brain age, and whether the weights were uniform.

There was no average computed for the various structures upon which we could base a predicted brain age for the whole brain. Whole brain age was calculated from the Volbrain's BrainStructuresAges pipeline as its own value. As for the weighting, since we did not train the original model and no salience maps were described in the original article (PMID: 38224546), it is unclear which structures or regions were unequally contributing to the predicted whole brain age. However, when comparing the results for the whole brain to specified regions, they appear to be well correlated, indicating that the disease process is captured by the whole brain predicted age.

3 Model Calibration and Use of Support Vector Regression
The cited method (PMID: 38224546) used support vector regression to model the relationship between chronological and predicted age. In this manuscript, the authors compute the average predicted age directly from structural outputs without such a correction. This results in a control group slope of 0.83 (i.e., predicted age increases less than 1 year per biological year), which may suggest suboptimal calibration. If the authors aim to use quantitative values of predicted age and BSAGE (e.g., 12.2 years/year in LI patients), then calibrating the model for slope ~1 in neurotypical controls might be necessary. Alternatively, if the focus is only on trajectory deviation (see point 1), recalibration may not be necessary.

We appreciate this comment. We elected not to recalibrate the model since our NC cohort did not span the entire human lifespan and only included participants between the ages of 2 and 30 years to overlap with our GM1 cohort. Furthermore, since we did not train the original model, and the original model included both a larger and more distributed normative dataset in terms of age, we believe this would move our analysis further from the ground truth.

4 Colormap Saturation in Brain Renderings (Figure 2 and Supplement D)
The authors appear to have deliberately limited the colormap scale, resulting in highly saturated ("white") renderings in LI and older Juv patients. This visually supports the claim of extreme predicted age deviation but prevents readers from forming independent impressions of gradient and severity. I recommend relaxing the colormap bounds to allow more nuanced visual comparison across cohorts and brain structures, or alternatively, explicitly provide a supplemental version with a full dynamic range.

In figure 2, the gradient was assigned from age 0 to twice the patient/participant age for each column. However, we see the value of a corresponding image with a full dynamic range. We have included this in Supplement D for axial, coronal, and sagittal versions of the images.

5 Redundancy in Displayed Results Across Brain Structures
Figures summarizing BSAGE or predicted age across dozens of structures, though comprehensive, yield little novel insight beyond what is seen in whole-brain summaries. Most regional findings closely mirror whole-brain results and are not separately discussed in detail. I suggest reducing these to highlight only the most discriminative or clinically relevant regions (e.g., thalamus, caudate, cerebellum), with full results moved to supplementary material.

We agree that the results for predicted age and BSAGE across the various structures are repetitive. Hence, we have consolidated figures 3 and 4 into a single figure and moved 7

graphs to supplement H. We believe the remaining structures including the whole brain, thalamus, caudate nucleus, globus pallidus, putamen, and both the lateral and inferior lateral ventricles are important due to previous reports documenting pathogenic atrophy/enlargement of those regions in this cohort (PMID 29352662, 39874851). Furthermore, the dissection of the cerebellum into specific lobules could be valuable since it was included in our previous report of late-onset GM2 (PMID: 40297453) and since dysarthria, clumsiness, and cerebellar atrophy are well-documented in Type II GM1 patients.

6 Confusing and Inconsistent Language in Reporting Predicted Age Differences
The manuscript contains several instances in which the terminology used to describe predicted brain age differences is confusing or internally inconsistent. For example, in the comparison between juvenile and late-infantile (LI) GM1 patients, the authors report that "LI GM1 patients had a -34.12 ± 15.04-year lower predicted brain age on average compared to Juv GM1 patients" (line 149), while also noting that Juv patients had a "10.31 ± 1.61-year higher predicted whole brain age on average compared to controls" (line 112). Elsewhere, they describe GM1 patients having "18.57 ± 1.73-year higher predicted brain age" than controls (line 95).
This language becomes problematic because in all cases the observed effect is essentially the same: a higher predicted brain age in GM1 patients relative to controls or subtypes. Yet the inconsistent use of negative versus positive descriptors (e.g., "lower predicted age" vs. "higher predicted age") and ambiguous sentence structure makes interpretation unnecessarily difficult.

The language refers to the y-intercept of the lines from the linear mixed effects model (constant effect). For the late-infantile patients the y-intercept of the best fit line was -34.12 years of predicted brain age. In the late-infantile patients, due to the high slope of this line (more than 12 year predicted brain age increase per chronological year) this effect is not present, as the x-intercept (2.8) of this line is close to the age of youngest late-infantile patient scanned (2.0 years old). The late-infantile patients' predicted brain age likely has an exponential relationship with chronological age, such that patients under age 2 (when they have more minor symptoms or are asymptomatic) have more normative brain changes, before the disease progression becomes more rapid. However, since we do not have any patients with scans under the age of 2, we do not have sufficient data to prove this hypothesis.

In the juvenile patients and in the entire GM1 cohort, the interpretation is more straightforward as there is a positive vertical shift in the line from normative development and further divergence in the slope of the relationship between predicted brain and chronological age from there.

We understand how this caused confusion and have updated the results section to clarify this confusion. We provided specifics when referencing the slope versus the y-intercept.

7 Clarification of Sample Sizes Across Datasets
Authors need to state the number of subject number of each dataset in the method. The authors state in Supplement Figure E1: "n = 556, 897 total MRI scans". Easily misleading, when I first read it I thought you had 6 figures of subjects.

We agree with Reviewer 3 that the above referenced language is misleading. Hence, we have clarified throughout the manuscript and supplement with the following change "897 MRI scans from 556 participants" and similar changes to phrasing regarding late-infantile and juvenile GM1.

8. Please define BSAGE at its first occurrence in the abstract and main text (currently only defined in Methods).

We have made this correction to the manuscript.

Reviewer #4 (Remarks to the Author):

General Comment
The authors conducted brain age estimation using BrainStructuresAges on MRI scans of patients with GM1 gangliosidosis. Compared to healthy controls, the predicted brain age of the patients was estimated to be older than their chronological age, and the gap between predicted and chronological age was associated with disease severity. This suggests that brain age estimation could serve as a quantitative evaluation method for the brains of patients with GM1 gangliosidosis.

Major Comments
1. It would be helpful to include a description of how the annual brain aging rate was calculated. Some patients underwent multiple MRI scans: it is unclear whether this was taken into account.

The annual brain aging rate was calculated using the linear mixed effects model (the estimate) with a subject-level random intercept to account for participants with multiple MRI scans. Since the model was fit with both NC and patients (Model 1 shown below, Supplement C), the aging rate was calculated solely as the estimate of Age for NC (since NC were assigned a value of 0 in all three models). For the whole GM1 cohort (late-infantile and juvenile combined), the aging rate was calculated from the LMEM with NC assigned a value of 0, and all GM1 patients assigned a value of 1, where the aging rate was calculated as the estimate of Age plus the estimate of the interaction between Age and

Cohort (since Cohort was equal to one for GM1 patients). The same method was used to calculate aging for the late-infantile GM1 patients with the juvenile patients removed from the analysis. Further, the same method was used to calculate aging for the juvenile cohort, with the late-infantile patients removed from the analysis. We added the following language to the statistical analysis section of the methods "the slope from the line between predicted brain age and chronological age was quantified for each of the four cohorts analyzed (NC, juvenile GM1, late-infantile GM1, and entire GM1 cohort) to quantify brain aging in each cohort" (lines 188-190).

Model 1:

model1 <- lmer(Predicted Brain Age ~ Age * Cohort + Sex + (1|Participant), dataset)

summary(lmer)

Predicted Brain Age – Corresponds to the predicted brain age determined by *BrainStructuresAges* for the structure being analyzed
Cohort – Corresponds to the comparison between cohorts as presented in Table 1.
Sex – Corresponds to the participants' biological sex
Age – Corresponds to the participants' chronological age
Participant – Each participant was given a distinct number to account for repeated measures (subject level random intercept).

2. The Results section contains noticeable redundancies. Similar sentences are repeated with only slight changes in numerical values. In particular, the repetition of content around lines 103–108 stands out. Using tables to summarize such data could improve readability and conciseness.

We agree with this comment from Reviewer 4 and have consolidated much of the Results section including the specified lines.

3. The manuscript states that the average predicted brain age in late-infantile GM1 (LI GM1) patients is −21.29 ± 2.76 years compared to controls (lines 132–133). However, the predicted brain age in LI GM1 patients should be higher than that of controls, suggesting this may be an error. A similar issue appears in line 150.

We appreciate this comment from reviewer 4 towards improving the clarity of our manuscript. Reviewer 3 had the same concern which we have addressed and updated in the results section of the manuscript.

The language refers to the y-intercept of the lines from the linear mixed effects model (constant effect). For the late-infantile patients the y-intercept of the best fit line was -34.12 years of predicted brain age. In the late-infantile patients, due to the high slope of this line (more than 12 year predicted brain age increase per chronological year) this effect is not

present as the x-intercept (2.8) of this line is close to the age of youngest late-infantile patient scanned (2.0 years old). The true relationship for the late-infantile patients' predicted brain age likely has an exponential relationship with chronological age, such that patients under age 2 (when they have more minor symptoms or are asymptomatic) have more normative brain changes, before the disease progression becomes more rapid. However, since we do not have any patients with scans under the age of 2, we do not have sufficient data to prove this hypothesis.

In the juvenile patients and in the entire GM1 cohort, the interpretation is more straightforward as there is a positive vertical shift in the line from normative development and further divergence in the slope of the relationship between predicted brain and chronological age from there.

We understand how this caused confusion and have updated the results section to clarify this confusion. We provided specifics when referencing the slope versus the y-intercept.

4. Since the estimated age and BASAGE are similar metrics, their content seems redundant. Given that BASAGE, which is corrected for the age at the time of imaging, is likely the more important indicator, it might be preferable to reduce the emphasis on the results for the estimated age itself.

We agree that BSAGE and estimated age are quite similar metrics. We also agree that BSAGE is the more important metric. However, due to the novelty of predicted brain age in a pediatric neurodegenerative condition, we deemed it important to show both metrics in detail.

5. There is considerable variability in BASAGE among GM1 patients—some show very high values, while others are close to zero. Please discuss possible reasons for this variability. For example, are there any distinctive imaging features that might explain these differences?

We agree there was variability in both BSAGE and predicted age in the juvenile GM1 cohort. We have added supplemental figure D11 to address this concern. There were a few juvenile patients who were scanned early in their disease course who did not show substantial divergence from normal in terms of brain development. There were also a couple of juvenile patients who had slower progression in their disease; this was accurately reflected in low BSAGE values as shown in Figure D11. We have added the following language to the discussion to further address this concern: "Furthermore, in a few juvenile patients who were scanned either early in their disease course or did not have significant GM1 progression, BSAGE was reflectively low, further substantiating the accuracy and utility of the model (Supplement Figure D11) (lines 416-418)."

6. What kinds of brain changes are reflected by a high BASAGE value? Although there is some discussion of the relationship between brain volume and predicted age, it may not be sufficient. As your study also provides region-wise results, further discussion from that perspective would be valuable.

We appreciate the reviewers comment towards providing clarity to the underlying changes in BSAGE. To address this concern, we have added supplemental figures D9 and D10 showing characteristic brain aberrations associated with GM1. We also added the following language to the discussion (lines 361-365): "as shown in supplemental figures D9 and D10 increases in both whole brain predicted brain age and BSAGE are reflective of volumetric changes known to be associated with GM1 progression including cerebral atrophy, lateral ventricle enlargement, corpus callosum atrophy, cerebellar atrophy, and demyelination."

7. Given the high variability in predicted brain age among patients, it may be difficult to use this as an individual clinical marker despite group-level differences. Furthermore, due to the limited longitudinal data, there is insufficient evidence to demonstrate that predicted age correlates with disease progression. Therefore, referring to it as a "neuroimaging surrogate clinical outcome assessment" may be an overstatement.

We tempered the language in the referenced paragraph to a degree. However, the correlations between predicted brain age and both the Clinical Global Impressions (CGI) Scale and Vineland adaptive behavioral scales (both validated clinical outcome assessments) do indicate the significance as a surrogate clinical outcome assessment. Patients who were further in their disease process had higher CGI scores (worse), lower vineland scores, and higher predicted brain age.

Minor Comments

1. Please standardize the terminology used for disease names. For example, expressions like "late-infantile GM1 gangliosidosis," "L1 GM1," "late-infantile GM1," and "late-infantile" are used inconsistently. The same applies to "juvenile GM1."

We agree and have updated the manuscript and supplement to include 'juvenile' and 'late-infantile' terminology uniformly.

2. Unify the usage of either "biological" or "chronological" when referring to participants' actual ages.

We have replaced all references to "biological age" with "chronological age".

3. In line 97, "the interaction between biological age and GM1" may be intended as "the

interaction between biological and estimated age in GM1." Please check similar phrases repeated later as well.

We agree that the interaction language was unclear. Hence, in the Results section, we have updated the language to say (lines 206-207) "the interaction between chronological age and GM1 diagnosis" as it is the interaction between participants' biological age and their cohort designation (NC, GM1, late-infantile GM1, or juvenile GM1) that was tested.

4. "Figure 7" (line 200) should be corrected to "Figure 7A and 7B."

We agree and have updated the manuscript to say "Figure 7A and 7B" accordingly.

5. "Figure 2B" in lines 181 and 186 should be corrected to "Figure 2."

We have corrected Figure 2B in lines 181 and 186 to read Figure 1B in both locations as originally intended.

6. "Figure 3" in line 709 should be corrected to "Figure 5."

We agree Figure 5 (line 709) was mislabeled as Figure 3. We have updated the manuscript to address this concern.

Reviewer #1 (Remarks to the Author):

The authors have made improvements to their manuscript based on the questions previously raised.

We appreciate Reviewer 1's time and consideration of our work.

Reviewer #2 (Remarks to the Author):

I read the response letter for me part. But I still hold some questions:
1. In response to my previous comment 1, you stated: "This advanced aging correlated with clinical outcomes (Vineland and CGI scores), so that, if the gene therapy were to slow the rapid brain aging, it would suggest therapeutic benefit." However, the notion that gene therapy may slow brain aging appears to be a hypothesis, and your current results do not provide direct evidence to support it. Nor do you cite any external references substantiating this assumption. Therefore, I still believe the statement claiming that your findings "demonstrate therapeutic efficacy attendant to interventions such as gene therapy" is too strong. At least, you should elaborate on this logic in the discussion section and cite relevant literature that supports this proposed link.

We have toned down the strength of our language in the indicated paragraph to reflect the speculative nature of this claim. "This evaluation of brain aging in GM1 suggests it may be an important neuroimaging marker of disease progression. Additionally, improvements or reversal of the brain aging metrics or decreased BSAGE in this cohort resulting from therapeutic intervention including gene therapy may be reflective of morphological and therapeutic benefit. Future studies are needed to validate the clinical significance of brain aging and the role of brain age prediction in the evaluation of therapies in this cohort."

Additionally, you stated: "Since the brain age prediction model was trained using deep learning it is plausible that this type of analysis is more robust than a simple thickness, surface area, or volumetric measurement.", I also hold different opinions. I have engineering background in computer vision and medical imaging processing. In general, with sample size less than 10 thousand, deep learning sometimes cannot outperform machine learning. I know there are lots of studies using deep learning as well as machine learning to estimate brain aging. About the model choosing, this is a disputed topic. But, coming back to your case, at least, you should add one more sentence with referring paper to support the advantage of deep-learning model in section of MRI Processing.

We agree with the assessment by Reviewer 2 that the literature is divided on the abilities of deep learning brain age prediction models compared to machine learning with some reports indicating deep learning models are superior and other reports suggesting machine learning models are comparable. However, our argument is that the predicted brain age as a voxel derived measure is likely more robust than just taking the volume, cortical thickness, or cortical surface area of a region in isolation. We have updated the manuscript with the following clarification, "due to the voxel level approach".

2. In response to my previous comment 3, I know they extracted low-level image features like cortical features. However, GAM can be used on raw MRI image, you can just vectorize all voxels in 2d image array as vector to train a model. Since you only mentioned you used deep learning model, I have no idea what type of deep learning you are using, transformer, BERT, CNN. Even for CNN, there are many of different typical models including ResNet, DenseNet, Unet. Ideally, you should make a basic control analysis to see whether using other deep learning model, the statistical analyses between brain aging and some cognitive/clinical test index was same. Otherwise, adding short discussion in the limitation to admit we did not try other models, it is interesting to see how the results of other models look like in future. Tbh, since I have one or two projects which also focus on brain aging (more than thousands of subjects), if the downstream statistical analyses to clinical measurements did not produce very smaller p values ($p<0.0001$), the results using different model might change. This is why I think at least, you should say something about this into limitations.

The original model trained by Nguyen el al. (PMID: 38224546) utilized a 3-D U-Net CNN which demonstrated comparable results to other models. However, we do acknowledge the limitations of not attempting replicate analysis with alternative models in this cohort. We have added the following language to the limitations section to address this concern. "Third, the present study is dependent on a single deep learning U-Net convolution neural network, to which future studies should replicate the present results with alternative models and training data."

If all those questions have be further addressed, I agree this paper can be accepted.

Reviewer #3 (Remarks to the Author):

The authors have substantially improved the manuscript in this revision. The writing is clearer, and the study design and results are now more logically presented. The proposed MRI-derived

brain age metrics offer potentially important insights into Type II GM1 gangliosidosis. However, several key conceptual issues remain unresolved, particularly regarding the interpretation and applicability of "brain age gap" in the context of severe pediatric neurodevelopmental disorders.

1. Interpretability of Brain Age in Severe Neurodevelopmental Disorders Remains Problematic
The authors have not yet adequately addressed the core conceptual concern raised in the first round: whether assigning predicted brain ages of 60–80 years to 3–5-year-old patients with late-infantile GM1 gangliosidosis is biologically meaningful. Such extreme positive Brain Structure Age Gaps (BSAGE) suggest a fundamental deviation from normative brain development—not a simple acceleration along a typical aging trajectory.

We understand the conceptual concern with assigning significantly high predicted brain ages to pediatric patients. To this end, we have taken significant strides in the manuscript to orient the parameters away from aging and towards markers of neurodegeneration. We also have provided evidence (supplement D) to the significant deterioration of the brain as observed on MRI.

While the authors argue that nearly all GM1 patients fall outside the 95% confidence interval of the normative model, making an "on/off-trajectory" classification impractical, this does not fully resolve the concern. I recommend that the authors present the proportion of patients whose predicted brain ages exceed 1, 2, or 3 standard deviations above age-matched neurotypical controls. This could provide clinically meaningful gradation of disease severity, especially in early-stage patients.

We acknowledge your request and have added supplement L with the requested analysis.

More importantly, the manuscript should be revised to clarify the interpretive limits of the brain age concept in this context. For example, rather than stating that late-infantile GM1 patients have "brains of a 70-year-old," it would be more accurate to emphasize that their MRI features deviate substantially from normal pediatric development and lie outside the model's trained age range. In such cases, predicted age should be interpreted as an index of disease burden or neurodegenerative change, not as literal evidence of premature aging.

We acknowledge your concern. We do not believe we have made any remaining comments towards the notion our analysis indicates premature aging. We agree GM1 patients fall outside of normal development and have mentioned this in the limitations portion of the discussion with the following statement "Similarly, the accuracy of these models in analyzing data from pediatric neurodegenerative diseases requires further investigation". However, the model (PMID: 38224546 did have pediatric scans included during the training, so the age range of our patient cohort is reasonable.

The authors implicitly acknowledge this in noting that most GM1 patients lie “outside the scalar range” of the model. They now need to make this explicit in the text. This clarification is crucial to prevent misuse or misinterpretation of the brain age framework. It will also aid clinicians and readers in understanding that, for instance, a +35-year BSAGE in a 5-year-old child signals severely atypical brain structure, not that the child is biologically elderly. Such reframing would both improve conceptual rigor and enhance clinical utility.

We agree with the above-mentioned claim to improve clinical utility. We have addressed this concern in several portions of the discussion and abstract.

2. Terminology Related to “Surrogate Outcome” Should Be More Cautious
The authors present additional analyses showing correlations between brain age metrics and clinical measures (CGI, Vineland), supporting their clinical relevance. However, the evidence remains insufficient to justify calling these metrics “surrogate neuroimaging outcomes.” True surrogate outcomes must reliably reflect clinical endpoints in the context of treatment. At present, the study has not demonstrated whether therapeutic interventions (e.g., gene therapy) can alter the trajectory of predicted brain aging. The authors themselves acknowledge that the clinical significance of these findings remains to be validated. Accordingly, I recommend revising such terminology throughout the manuscript. Referring to predicted brain age as a “candidate imaging biomarker” or “potential outcome measure” would better reflect the current state of evidence and highlight the need for future validation.

We agree we have not documented improvements in brain aging because of therapeutic intervention, however brain aging accurately describes disease progression in this cohort. We have addressed this concern to reflect brain aging as a candidate neuroimaging biomarker in this cohort.

Reviewer #4 (Remarks to the Author):

The authors responded sincerely and accurately to all of my comments. In particular, their handling of my major comments 3, 5, and 6 has made the paper clearer and more insightful. I have no other concerns.

We appreciate Reviewer 4’s time and consideration of our work.
