# Supplementary material for: Brain aging in Type II GM1 gangliosidosis
Source: Commun Med (Lond). 2026 Jul 6;6:568. doi: 10.1038/s43856-026-01745-w (PMC13720018; doi:10.1038/s43856-026-01745-w)
Supplement: Supplementary file 2 [file 43856_2026_1745_MOESM2_ESM.pdf]

Brain Aging in Type II GM1 Gangliosidosis

Supplementary Material

Table of Contents

Methods……………………………………………………………………………………….…..2

Supplement A: Natural History Study Participant Characteristics……………..………...………..2

Supplement B: Neurotypical Controls Participants………………………………………..……5

Supplement C: Linear Mixed Effects Modeling (LMEM)……...……………………….....……..6

Results…………………………………………………………………………………...……...7

Supplement D: Axial, Coronal, and Anatomical Slices of Brain Aging…………………...………..7

Supplement E: Comparisons between Neurotypical Control Datasets…………………..………..17

Supplement F: Linear Mixed Effects Modeling (LMEM) Outputs…………………….…..……..19

Supplement G: Midbrain Stuctures Predicted Brain Age……………..…………………………..21

Supplement H: Longitudinal Brain structures age gap estimation (BSAGE) ………………..…..29

Supplement I: Cross-Sectional Brain structures age gap estimation (BSAGE)……….…………40

Supplement J: Comparisons Between Predicted Brain Age and Brain Volumetrics……………..61

Supplement K: Descriptive evaluation of accelerated brain aging in GM1 cohort….…………...73

Supplementary References………………………………………………………………………72

Supplementary Methods
Supplement A: Natural History Study Participant Characteristics

Patients were enrolled in the National Human Genome Research Institute (NHGRI) study of the "Natural History of Glycosphingolipid Storage Disorders and Glycoprotein Disorders" (ClinicalTrials.gov ID: NCT00029965).[1] Demographic information for each of the 41 GM1 patients is listed in Supplement Table 1, and the age of the entire cohort is shown in Supplement Figure 1.

Supplement Table 1. Natural History Study T1-Weighted MRI Scan Age (n = 41). N/A are designated when a participant did not have the above referenced MRI scan.

Participant	Sex	GM1 Sub-type	Baseline Age (years old)	Scan #2 Age (years old)	Scan #3 Age (years old)	Scan #4 Age (years old)	Scan #5 Age (years old)	Number of T1 Scans
1	M	Late-infantile	0-5	N/A	N/A	N/A	N/A	1
2	M	Late-infantile	0-5	N/A	N/A	N/A	N/A	1
3	F	Late-infantile	0-5	N/A	N/A	N/A	N/A	1
4	F	Late-infantile	0-5	N/A	N/A	N/A	N/A	1
5	F	Late-infantile	0-5	N/A	N/A	N/A	N/A	1
6	F	Late-infantile	6-10	N/A	N/A	N/A	N/A	1
7	F	Late-infantile	0-5	N/A	N/A	N/A	N/A	1
8	M	Late-infantile	0-5	0-5	N/A	N/A	N/A	2
9	M	Late-infantile	6-10	6-10	N/A	N/A	N/A	2
10	M	Late-infantile	0-5	6-10	N/A	N/A	N/A	2
11	F	Late-infantile	0-5	0-5	N/A	N/A	N/A	2
12	F	Late-infantile	6-10	N/A	N/A	N/A	N/A	1
13	M	Late-infantile	0-5	N/A	N/A	N/A	N/A	1
14	F	Late-infantile	0-5	N/A	N/A	N/A	N/A	1
15	F	Late-infantile	0-5	0-5	N/A	N/A	N/A	2
16	M	Juvenile	0-5	0-5	N/A	N/A	N/A	2
17	F	Juvenile	0-5	0-5	6-10	6-10	6-10	5
18	F	Juvenile	6-10	N/A	N/A	N/A	N/A	1
19	F	Juvenile	0-5	N/A	N/A	N/A	N/A	1
20	M	Juvenile	6-10	N/A	N/A	N/A	N/A	1
21	M	Juvenile	6-10	N/A	N/A	N/A	N/A	1
22	F	Juvenile	6-10	N/A	N/A	N/A	N/A	1
23	M	Juvenile	0-5	N/A	N/A	N/A	N/A	1
24	M	Juvenile	0-5	N/A	N/A	N/A	N/A	1
25	M	Juvenile	16-20	21-25	21-25	21-25	N/A	4
26	M	Juvenile	11-15	11-15	16-20	16-20	21-25	5
27	F	Juvenile	11-15	11-15	16-20	21-25	N/A	4
28	M	Juvenile	11-15	11-15	16-20	16-20	N/A	4
29	F	Juvenile	11-15	11-15	11-15	N/A	N/A	3
30	F	Juvenile	11-15	11-15	16-20	N/A	N/A	3
31	M	Juvenile	11-15	11-15	16-20	N/A	N/A	3
32	M	Juvenile	6-10	11-15	N/A	N/A	N/A	2
33	F	Juvenile	16-20	16-20	21-25	N/A	N/A	3
34	F	Juvenile	6-10	6-10	6-10	N/A	N/A	3
35	F	Juvenile	11-15	11-15	16-20	N/A	N/A	3
36	F	Juvenile	6-10	6-10	N/A	N/A	N/A	2
37	M	Juvenile	6-10	6-10	N/A	N/A	N/A	2
38	F	Juvenile	6-10	11-15	N/A	N/A	N/A	2
39	F	Juvenile	11-15	N/A	N/A	N/A	N/A	1
40	F	Juvenile	6-10	11-15	N/A	N/A	N/A	2
41	F	Juvenile	0-5	N/A	N/A	N/A	N/A	1

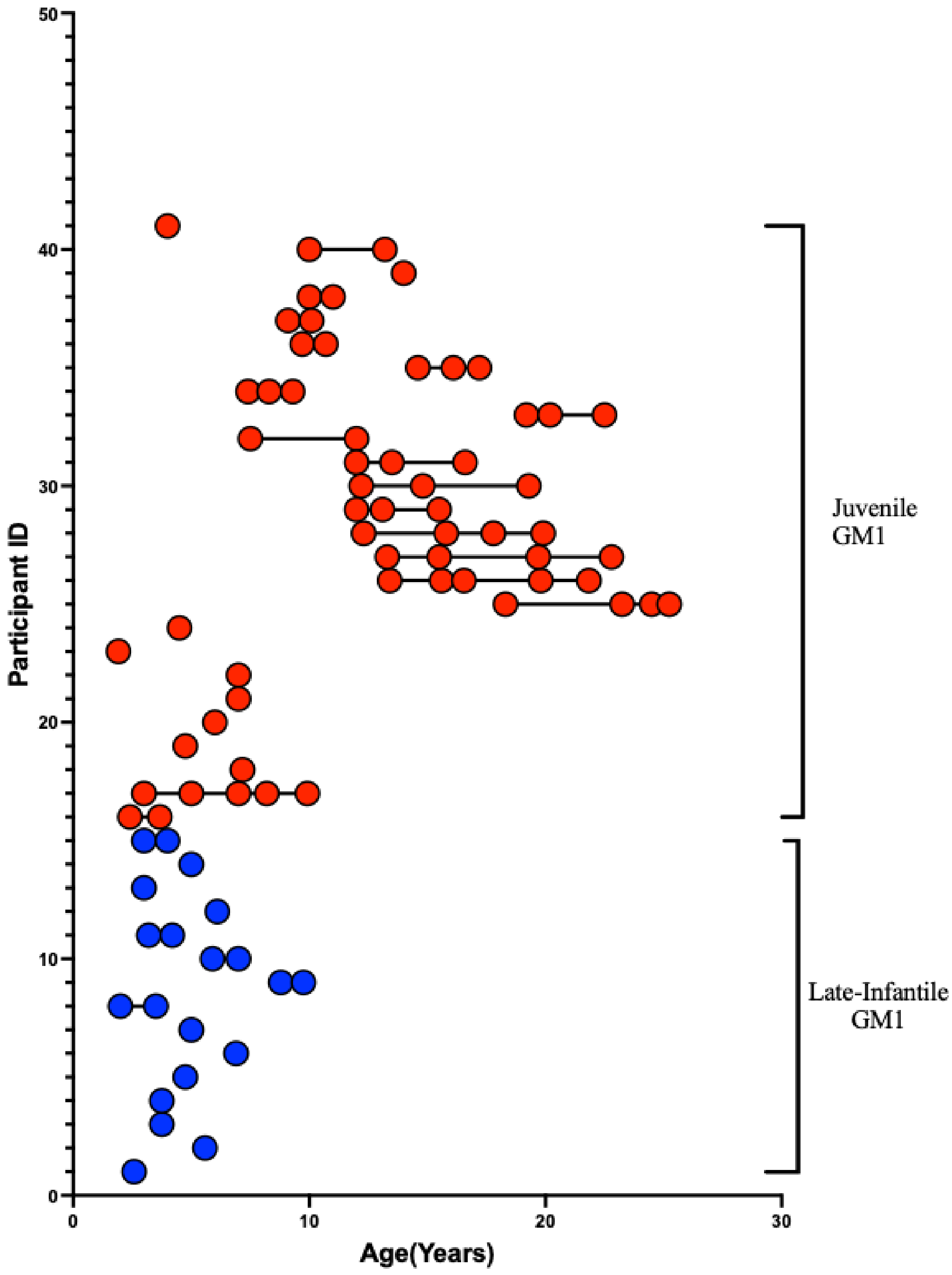

Supplement Figure 1. GM1 patient age at each T1-weighted MRI Scan. Late-Infantile (20 MRI scans from 15 patients) GM1 patients are shown in blue and juvenile (61 MRI scans from 26 patients) GM1 patients are red. Each T1-weighted scan is represented as a circle for all 81 scans; connecting lines indicate that a repeated scan was collected on the same patient. Each patient is shown on a separate row.

Supplement B: Neurotypical Control Participants

Supplement Table 2. Summary of Neurotypical Control Data Sets

	Calgary[2,3]	QTAB[4,5]	QTIM[6,7]
n	97	159	300
**Baseline Age (x̄ ± SD)**	3.84 ± 0.88	10.93 ± 1.36	21.72 ± 3.71
**Age (min, max)**	(1.97, 6.9)	(8, 16)	(12, 30)
**Males (*n*)**	51	82	123
**Females (*n*)**	46	77	177

First, 97 participants (279 scans) from the Calgary Preschool MRI dataset were included consisting of participants between the ages of 2 and 7 years old.[2,3] Second, 159 participants (318 scans) from the Queensland Twin Adolescent Brain (QTAB) were included in this study, consisting of participants between the ages of 8 and 16 years old.[4,5] Lastly, 300 participants (300 scans) from the Queensland Twin IMaging (QTIM) were included in this study, consisting of participants between the ages of 12 and 30.[6,7]

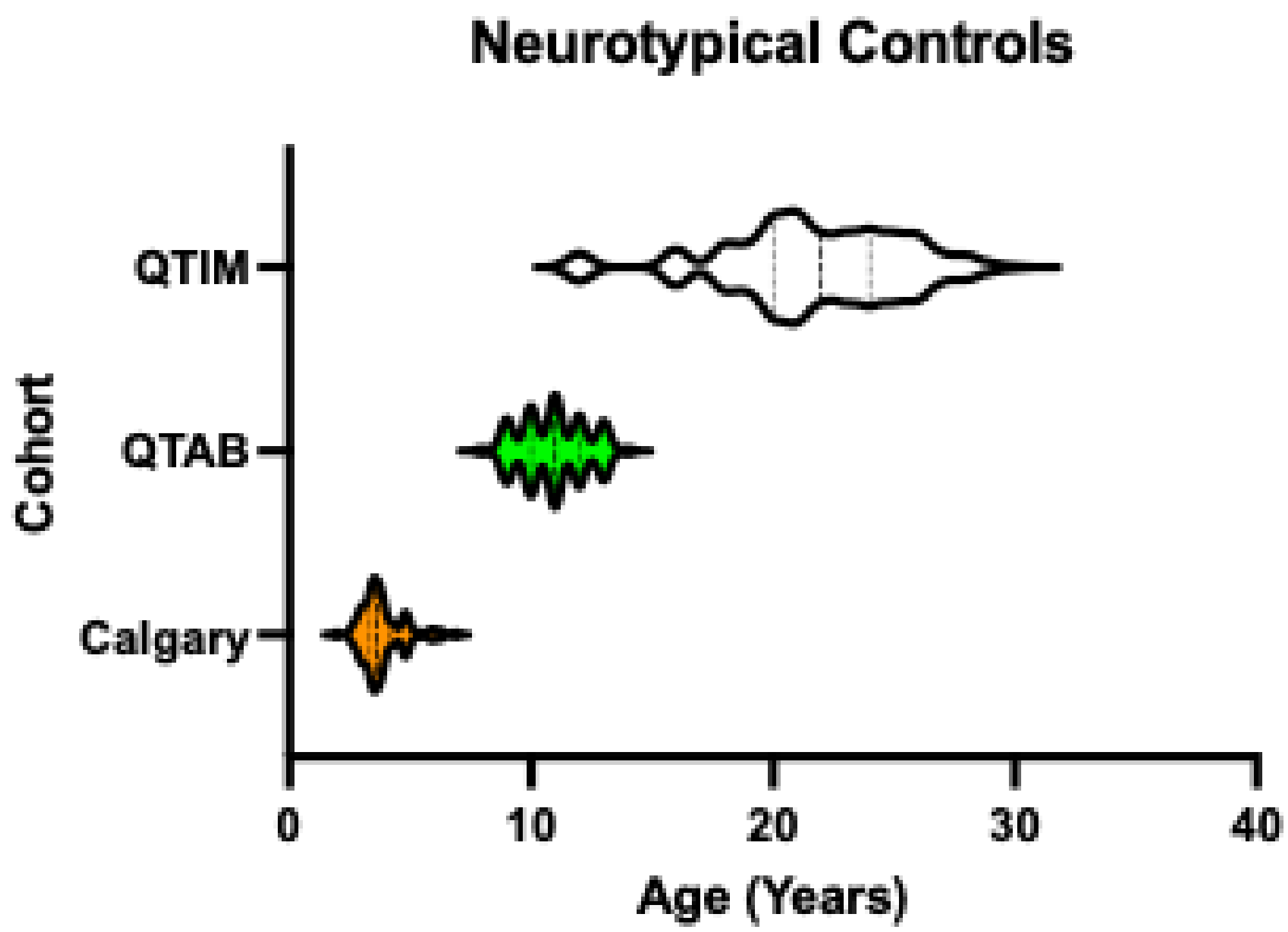

Supplement Figure 2. Violin Plot of Neurotypical Control Age. Baseline Scans were used for the Calgary and QTAB Datasets.

Supplement C: Linear Mixed Effects Modeling

Testing the interaction of Cohort and Age on predicted brain age
Linear mixed effects modeling was built in R[8] with the LME4[9-11] package. The interaction between the cohort and biological age was tested using the sample code below:

Model 1:
```
model1 <- lmer(Predicted Brain Age ~ Age * Cohort + Sex + (1|Participant), dataset)
summary(lmer)
```

Model 2:
```
model2 <- lmer(Predicted Brain Age ~ Age + Cohort + Sex + (1|Participant), dataset)
summary(lmer)
```

Likelihood-ratio test:
```
fm.anova <- anova(model1,model2)
summary(fm.anova)
```

Predicted Brain Age – Corresponds to the predicted brain age determined by *BrainStructuresAges* for the structure being analyzed
Cohort – Corresponds to the comparison between cohorts as presented in Table 1.
Sex – Corresponds to the participants' biological sex
Age – Corresponds to the participants' chronological age
Participant – Each participant was given a distinct number to account for repeated measures (subject level random intercept).

Supplementary Results

Supplement D: Axial, Coronal, and Anatomical Slices of Brain Aging

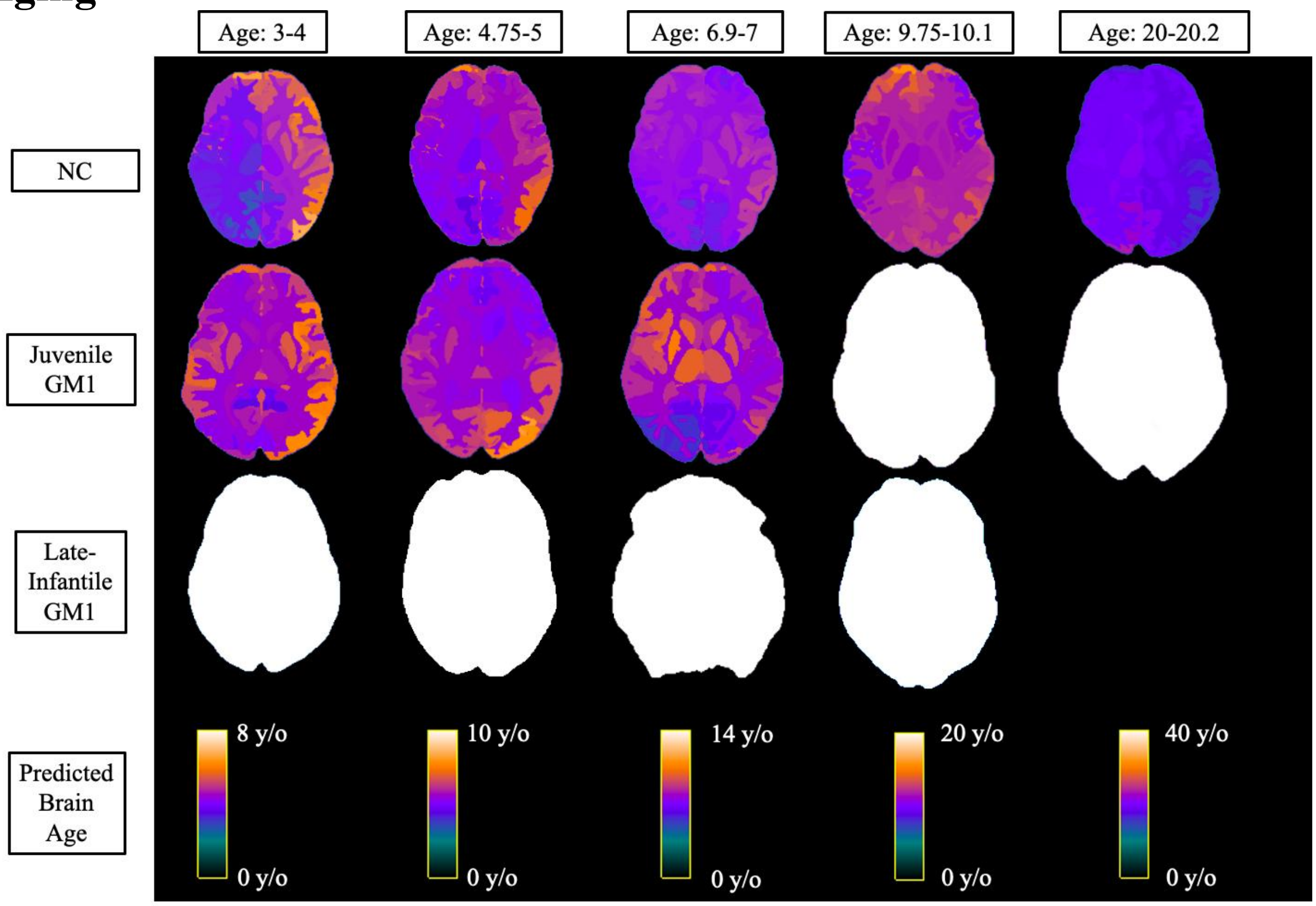

Supplement Figure 3. Axial View of Brain Structures Ages of the Three Cohorts at Varying Ages. Neurotypical controls (NC) are shown on the top row, juvenile GM1 gangliosidosis patients are shown in the middle row, and late-infantile GM1 gangliosidosis patients are shown in the bottom row. Colors were assigned according to the predicted age of that brain structure and were assigned between very black (lowest predicted age) and white (highest predicted age) as shown in the scalar bar in the bottom right portion. The scalar ranges were adjusted for each column with min/max values of 0-8 years for the first column (3.0-4.0 years old), 0-10 years for the second column (4.75-5 years old), 0-14 years for the third column (6.9-7 years old), 0-20 years for the fourth column (9.75-10.1 years old), and 0-40 years for the fifth column (20-20.2 years old). No 20-year-old MRI scan was available for late-infantile patients as the oldest late-infantile patient in this study was 9.75 years old at the time of the MRI evaluation. All MRI images were registered to the MNI coordinate space and slices were taken at z = 6.4 (± 1) mm for each participant. A matching anatomical version of this image is available (Supplement Figure 9).

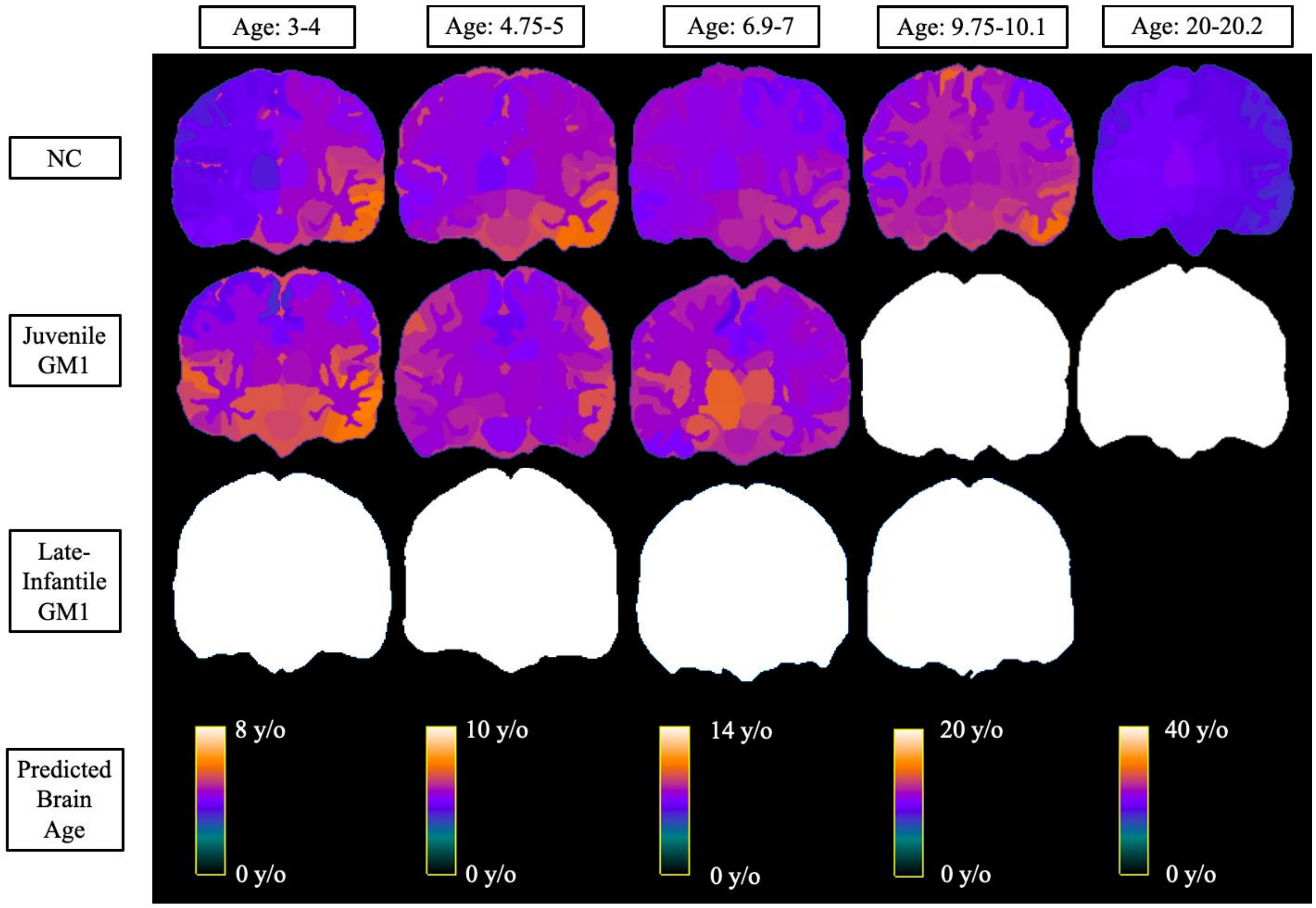

Supplement Figure 4. Coronal View of Brain Structures Ages of the Three Cohorts at Varying Ages. Neurotypical controls (NC) are shown on the top row, juvenile GM1 gangliosidosis patients are shown in the middle row, and late-infantile GM1 gangliosidosis patients are shown in the bottom row. Colors were assigned according to the predicted age of that brain structure and were assigned between very black (lowest predicted age) and white (highest predicted age) as shown in the scalar bar in the bottom right portion. The scalar ranges were adjusted for each column with min/max values of 0-8 years for the first column (3.0-4.0 years old), 0-10 years for the second column (4.75-5 years old), 0-14 years for the third column (6.9-7 years old), 0-20 years for the fourth column (9.75-10.1 years old), and 0-40 years for the fifth column (20-20.2 years old). No 20-year-old MRI scan was available for late-infantile patients as the oldest late-infantile patient in this study was 9.75 years old at the time of the MRI evaluation. All MRI images were registered to the MNI coordinate space and slices were taken at y = -17.6 (± 1) mm for each participant. A matching anatomical version of this image is available (Supplement Figure 10).

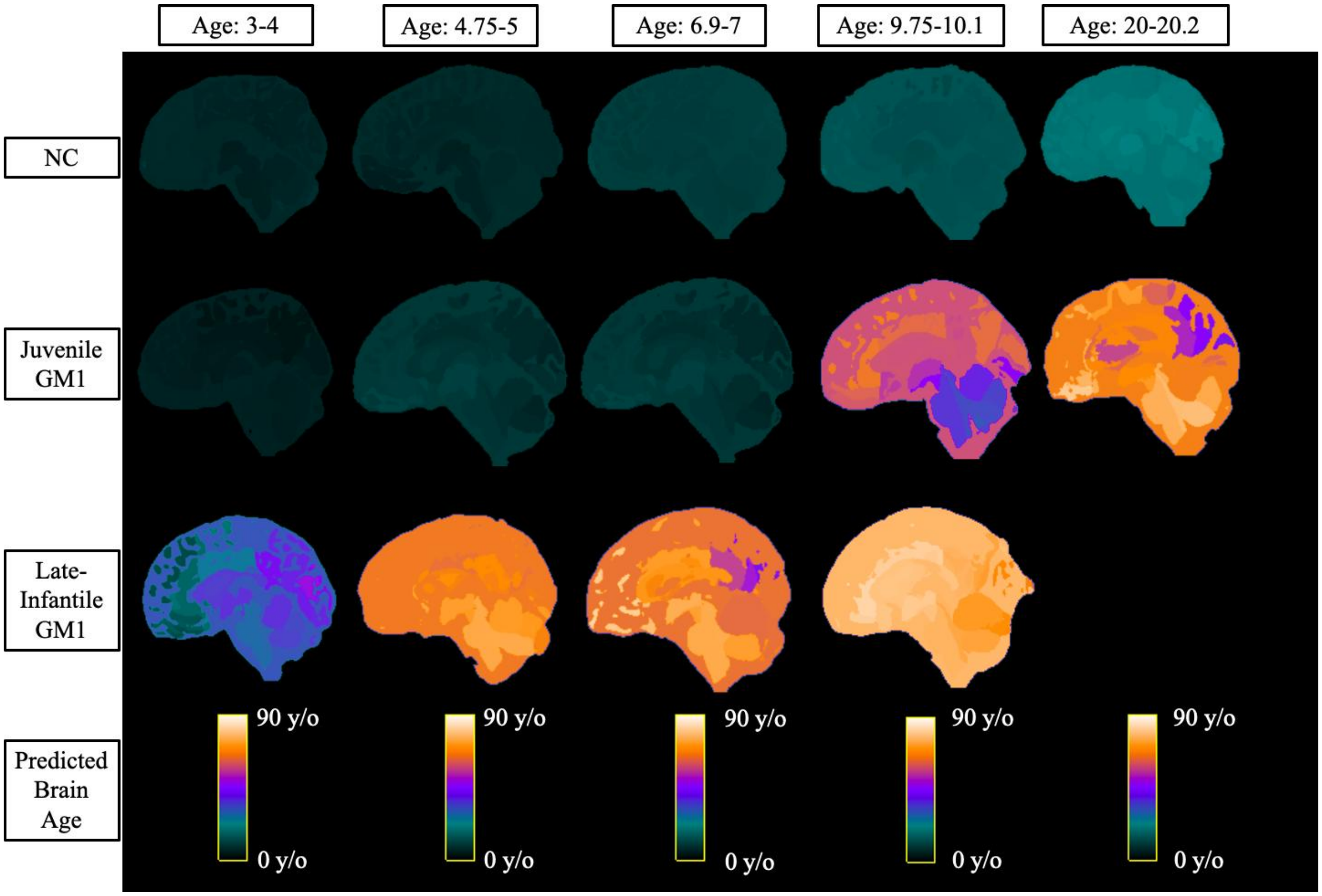

Supplement Figure 5. Saggital View of Brain Structures Ages of the Three Cohorts at Varying Ages with Full Dynamic Range. Neurotypical controls (NC) are shown on the top row, juvenile GM1 gangliosidosis patients are shown in the middle row, and late-infantile GM1 gangliosidosis patients are shown in the bottom row. Colors were assigned according to the predicted age of that brain structure and were assigned between black (lowest predicted age) and white (highest predicted age) as shown in the scalar bar in the bottom row. The scalar ranges included the full dynamic range between 0 and 90 years for predicted brain age in all columns. No 20-year-old MRI scan was available for late-infantile patients as the oldest late-infantile patient in this study was 9.75 years old at the time of the MRI evaluation. All MRI images were registered to the MNI coordinate space and slices were taken at x = 1.0 (± 1) mm for each participant. A matching anatomical version of this image is available (Supplement Figure 8).

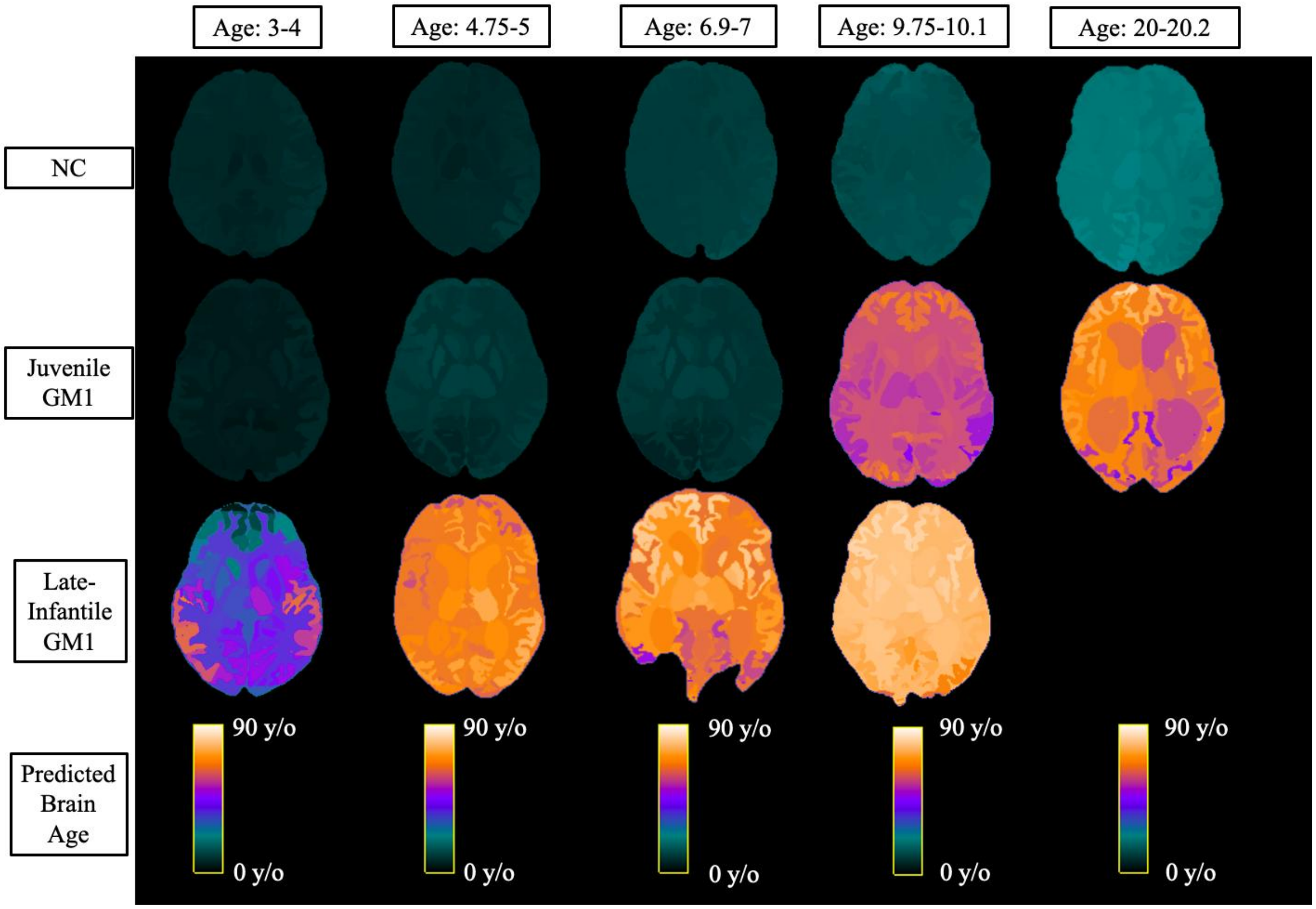

Supplement Figure 6. Axial View of Brain Structures Ages of the Three Cohorts at Varying Ages with Full Dynamic Range. Neurotypical controls (NC) are shown on the top row, juvenile GM1 gangliosidosis patients are shown in the middle row, and late-infantile GM1 gangliosidosis patients are shown in the bottom row. Colors were assigned according to the predicted age of that brain structure and were assigned between black (lowest predicted age) and white (highest predicted age) as shown in the scalar bar in the bottom row. The scalar ranges included the full dynamic range between 0 and 90 years for predicted brain age in all columns. No 20-year-old MRI scan was available for late-infantile patients as the oldest late-infantile patient in this study was 9.75 years old at the time of the MRI evaluation. All MRI images were registered to the MNI coordinate space and slices were taken at z = 6.4 (± 1) mm for each participant. A matching anatomical version of this image is available (Supplement Figure 9).

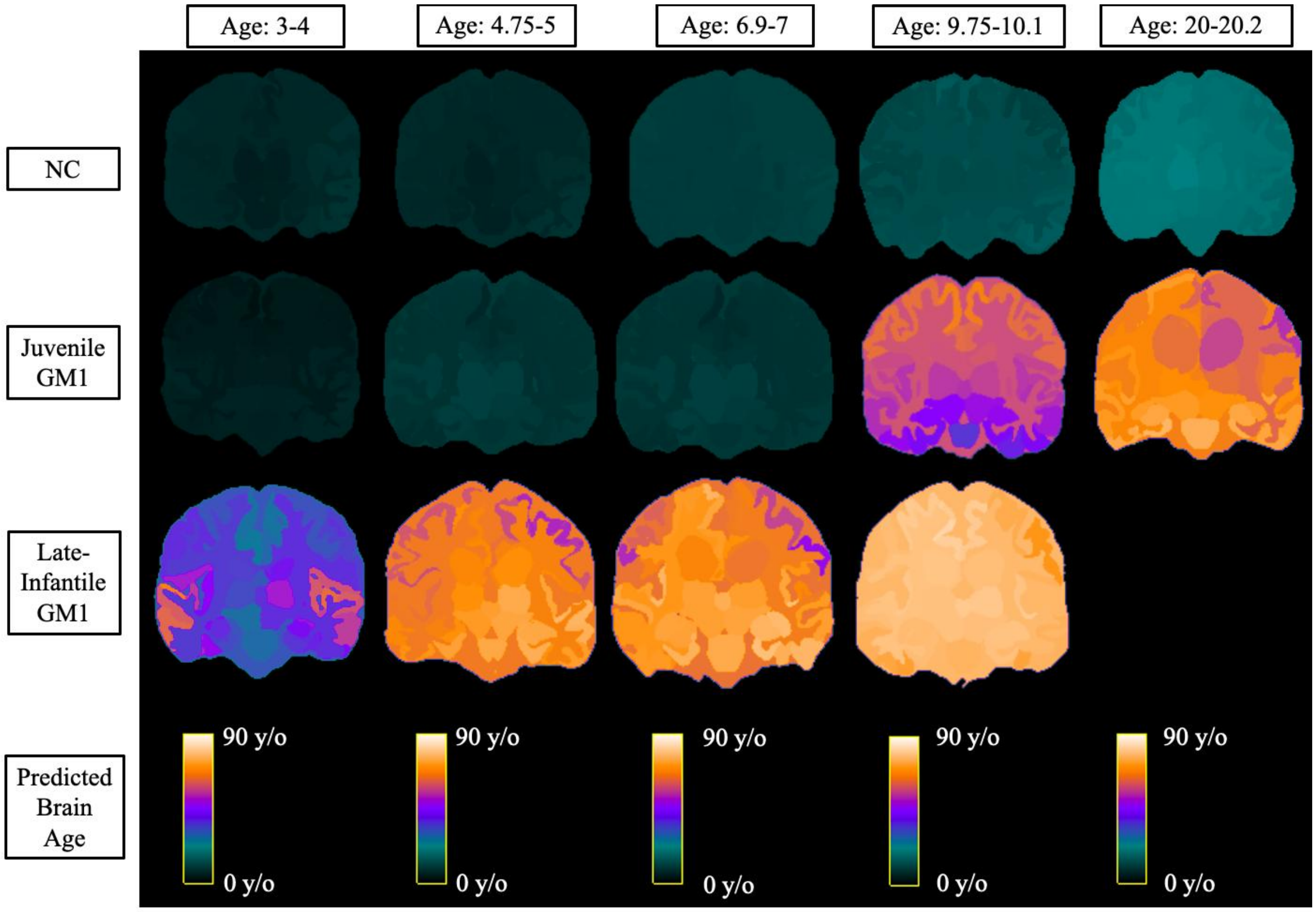

Supplement Figure 7. Coronal View of Brain Structures Ages of the Three Cohorts at Varying Ages with Full Dynamic Range. Neurotypical controls (NC) are shown on the top row, juvenile GM1 gangliosidosis patients are shown in the middle row, and late-infantile GM1 gangliosidosis patients are shown in the bottom row. Colors were assigned according to the predicted age of that brain structure and were assigned between black (lowest predicted age) and white (highest predicted age) as shown in the scalar bar in the bottom row. The scalar ranges included the full dynamic range between 0 and 90 years for predicted brain age in all columns. No 20-year-old MRI scan was available for late-infantile patients as the oldest late-infantile patient in this study was 9.75 years old at the time of the MRI evaluation. All MRI images were registered to the MNI coordinate space and slices were taken at y = -17.6 (± 1) mm for each participant. A matching anatomical version of this image is available (Supplement Figure 10).

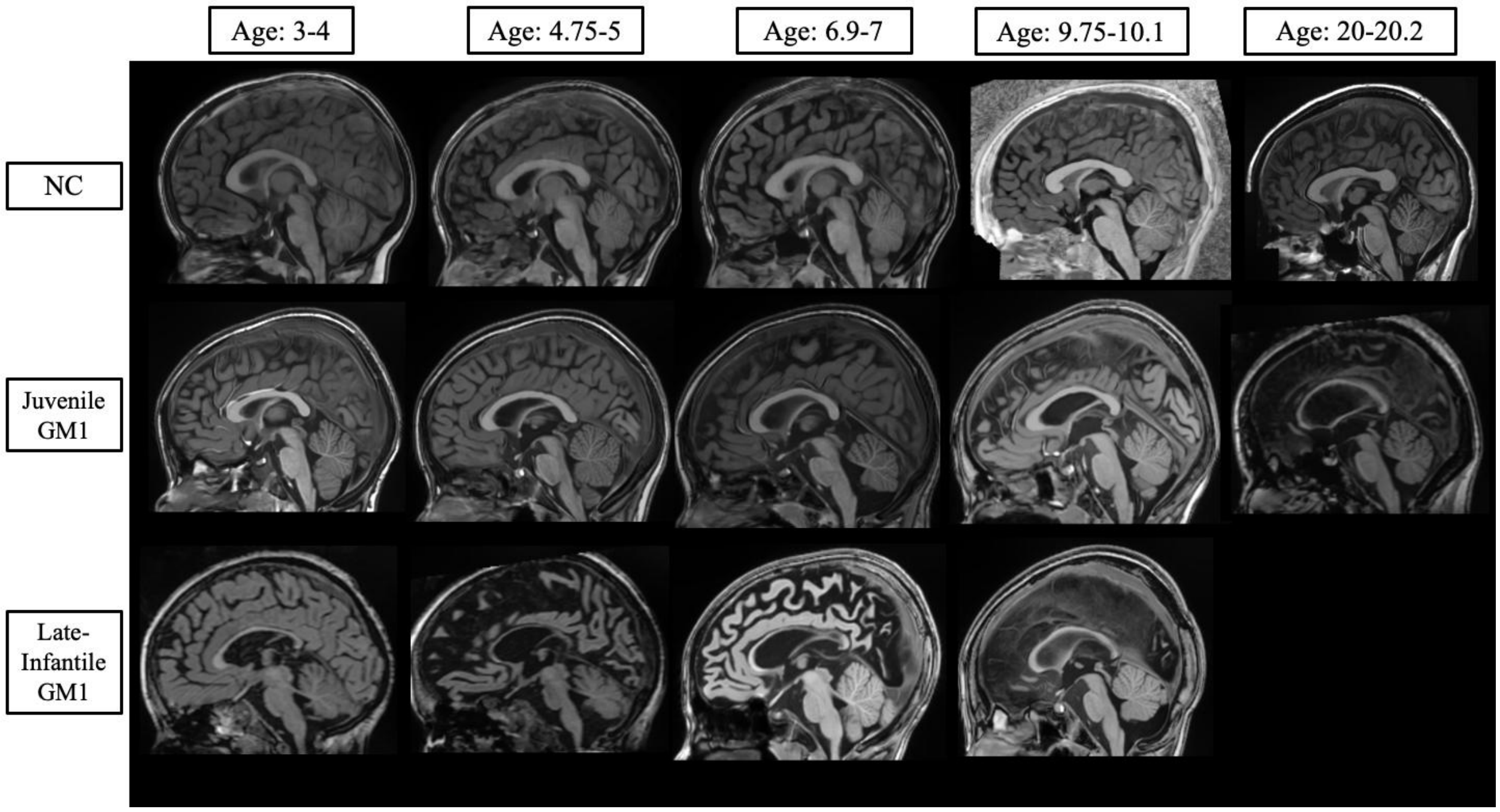

Supplement Figure 8. T1-Weighted Sagittal Anatomical Slice of Figure 2. Neurotypical controls (NC) are shown on the top row, juvenile GM1 gangliosidosis patients are shown in the middle row, and late-infantile GM1 gangliosidosis patients are shown in the bottom row. All MRI images were registered to the MNI coordinate space and slices were taken at x = 1.0 (± 1) mm (identical to Figure 2) for each participant.

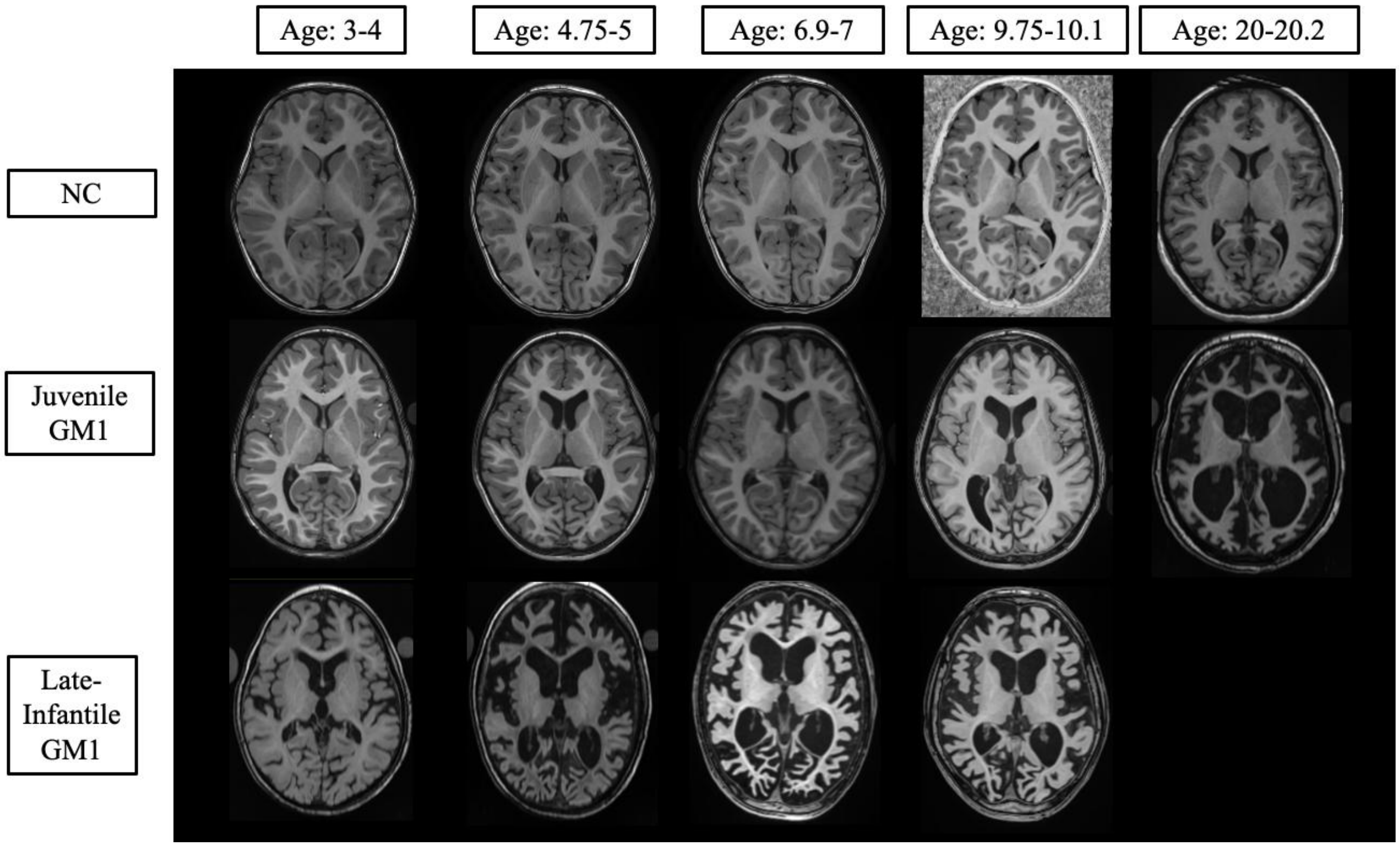

Supplement Figure 9. T1-Weighted Axial Anatomical Slice of Supplement Figure 3. Neurotypical controls (NC) are shown on the top row, juvenile GM1 gangliosidosis patients are shown in the middle row, and late-infantile GM1 gangliosidosis patients are shown in the bottom row. All MRI images were registered to the MNI coordinate space and slices were taken at z = 6.4 (± 1) mm (identical to Supplement Figure 3) for each participant.

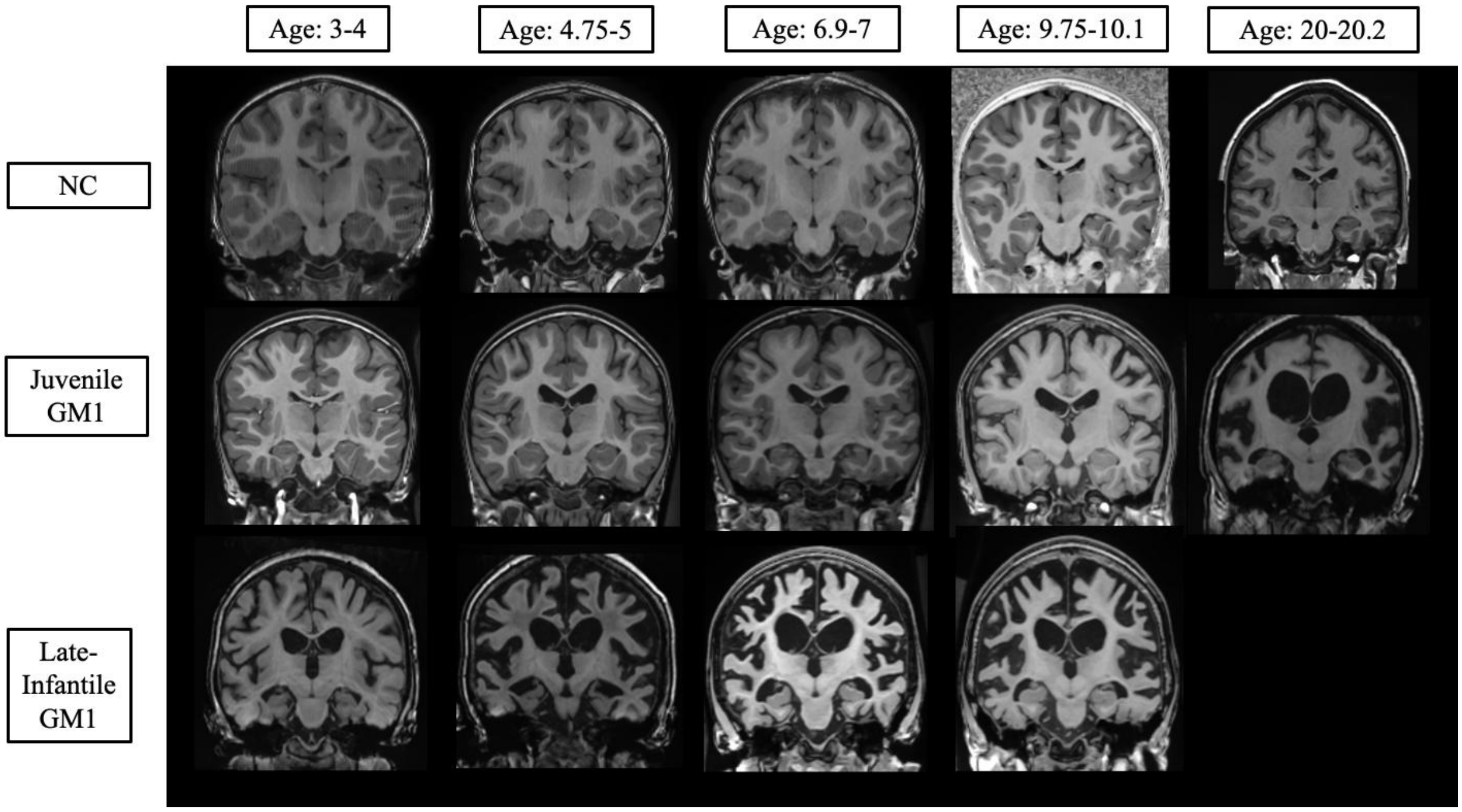

Supplement Figure 10. T1-Weighted Coronal Anatomical Slice of Supplement Figure 4. Neurotypical controls (NC) are shown on the top row, juvenile GM1 gangliosidosis patients are shown in the middle row, and late-infantile GM1 gangliosidosis patients are shown in the bottom row. All MRI images were registered to the MNI coordinate space and slices were taken at y = -17.6 (± 1) mm (identical to Supplement Figure 4) for each participant.

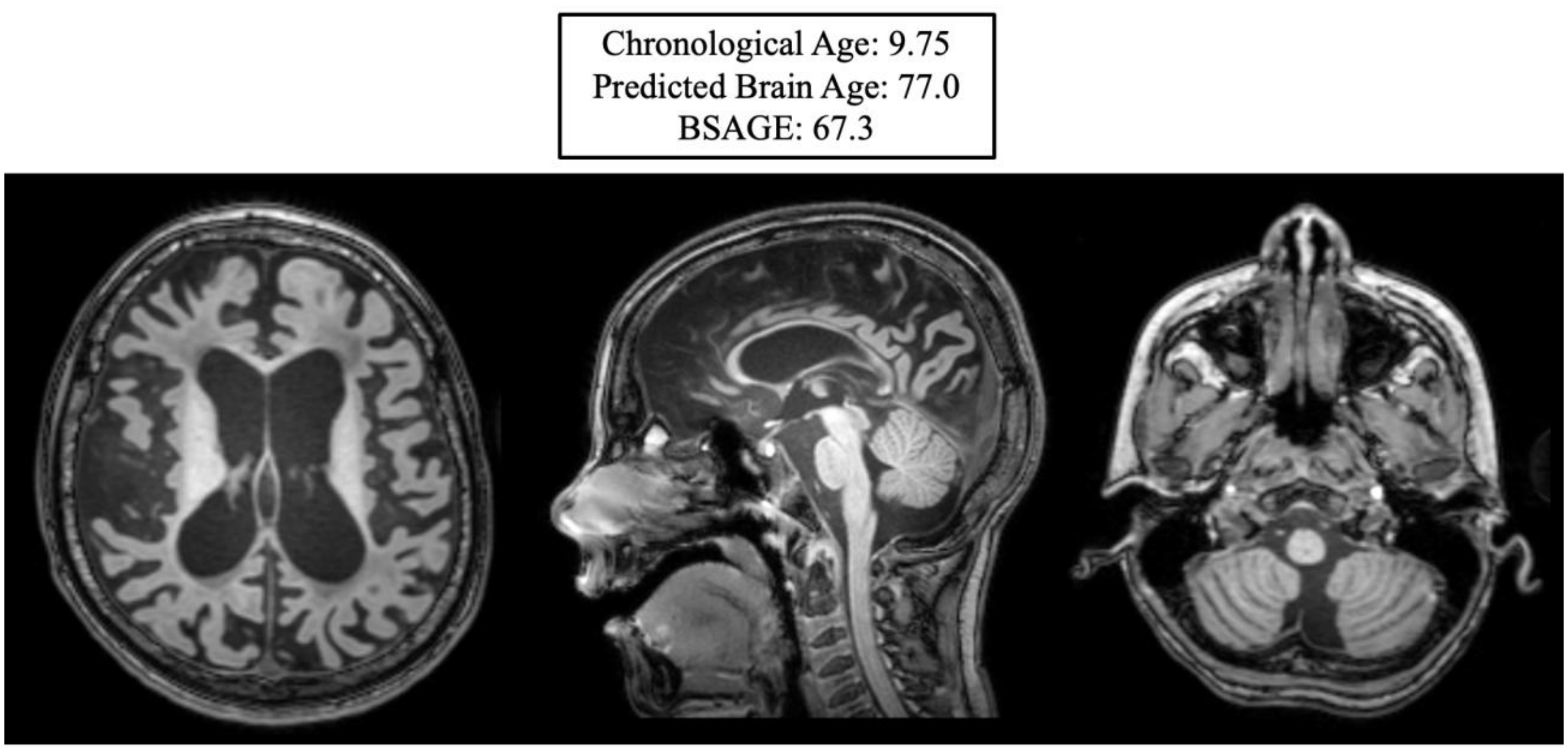

Supplement Figure 11. Late-infantile GM1 patient with advanced brain aging. Patient has characteristic findings of late-infantile GM1 including: enlarged lateral ventrciles indicative of cerebral atrophy, demylination, corupus callosum atrophy, and cerebellar atrophy.

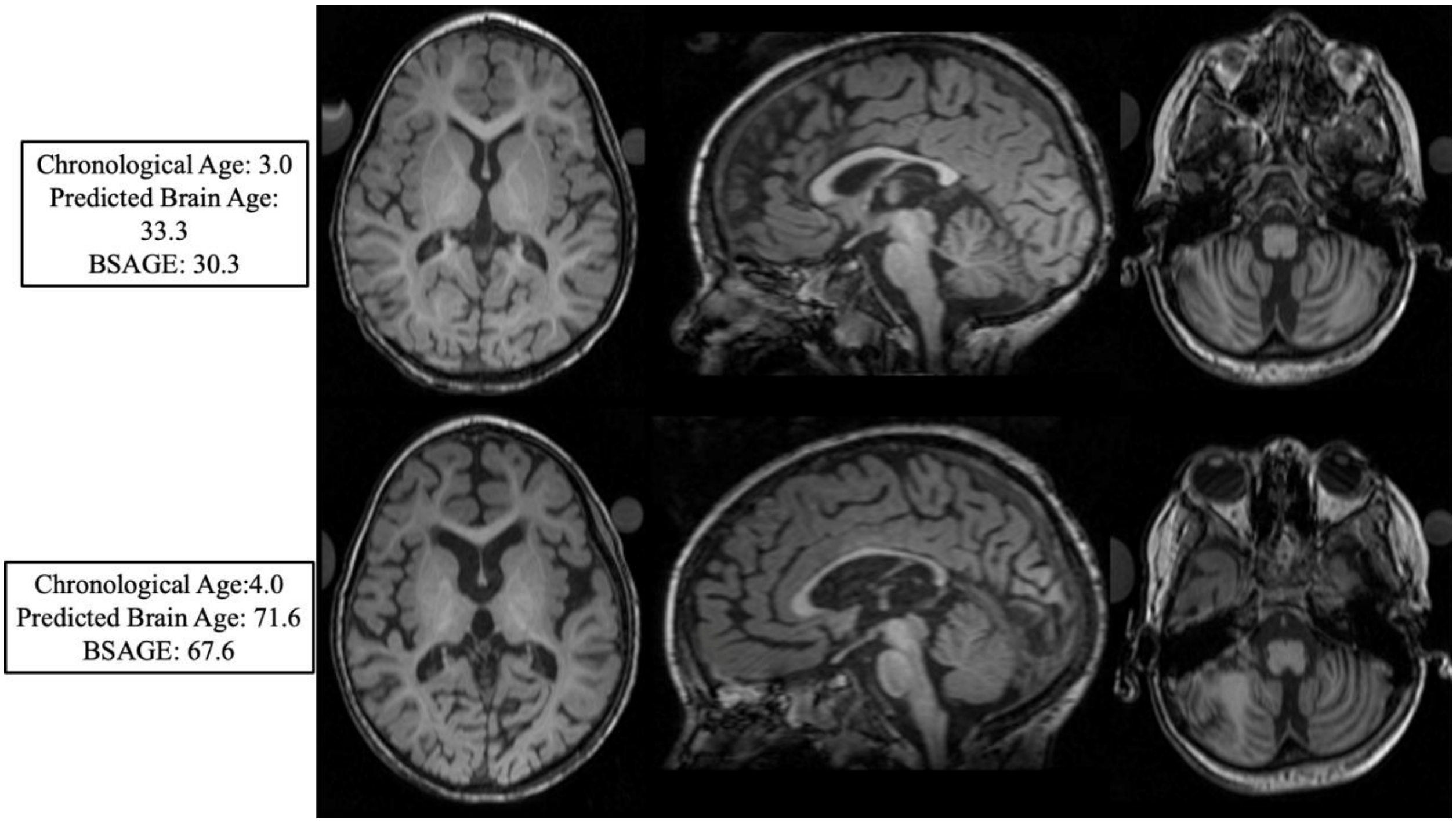

Supplement Figure 12. Longitudinal MRI scans of a late-infantile GM1 patient with advanced brain aging. Longitudinally, the patient shows characteristic findings of late-infantile GM1 progression including: enlarged lateral ventrciles indicative of cerebral atrophy, demylination, corupus callosum atrophy, and cerebellar atrophy.

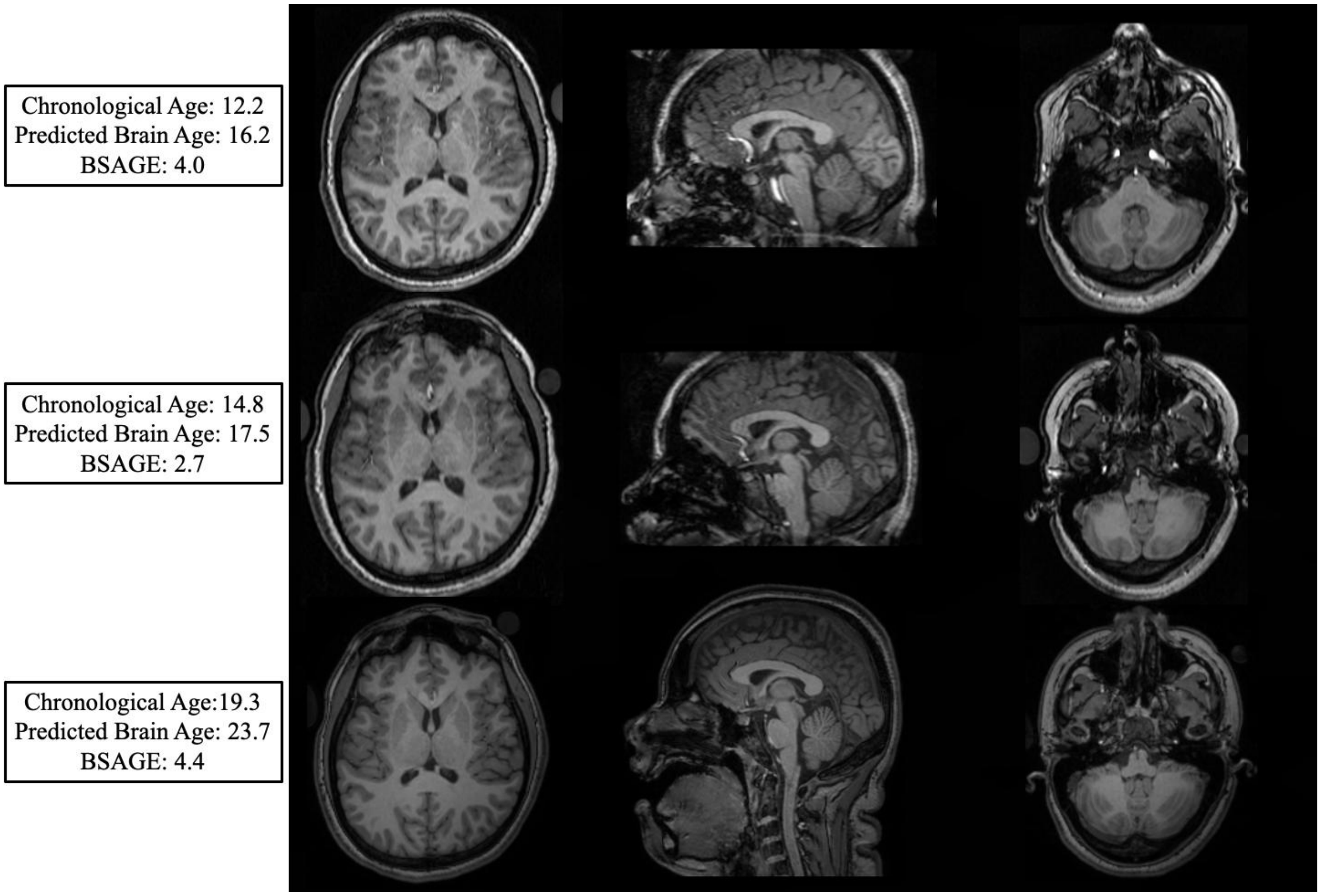

Supplement Figure 13. Longitudinal MRI scans of a juvenile GM1 patient with low BSAGE. Patient does not have the significant atrophy present in other patients in this cohort and has a much lower BSAGE. GM1 progression is also stable in this patient over more than 7 years.

Supplement E: Comparisons between Neurotypical Control Datasets

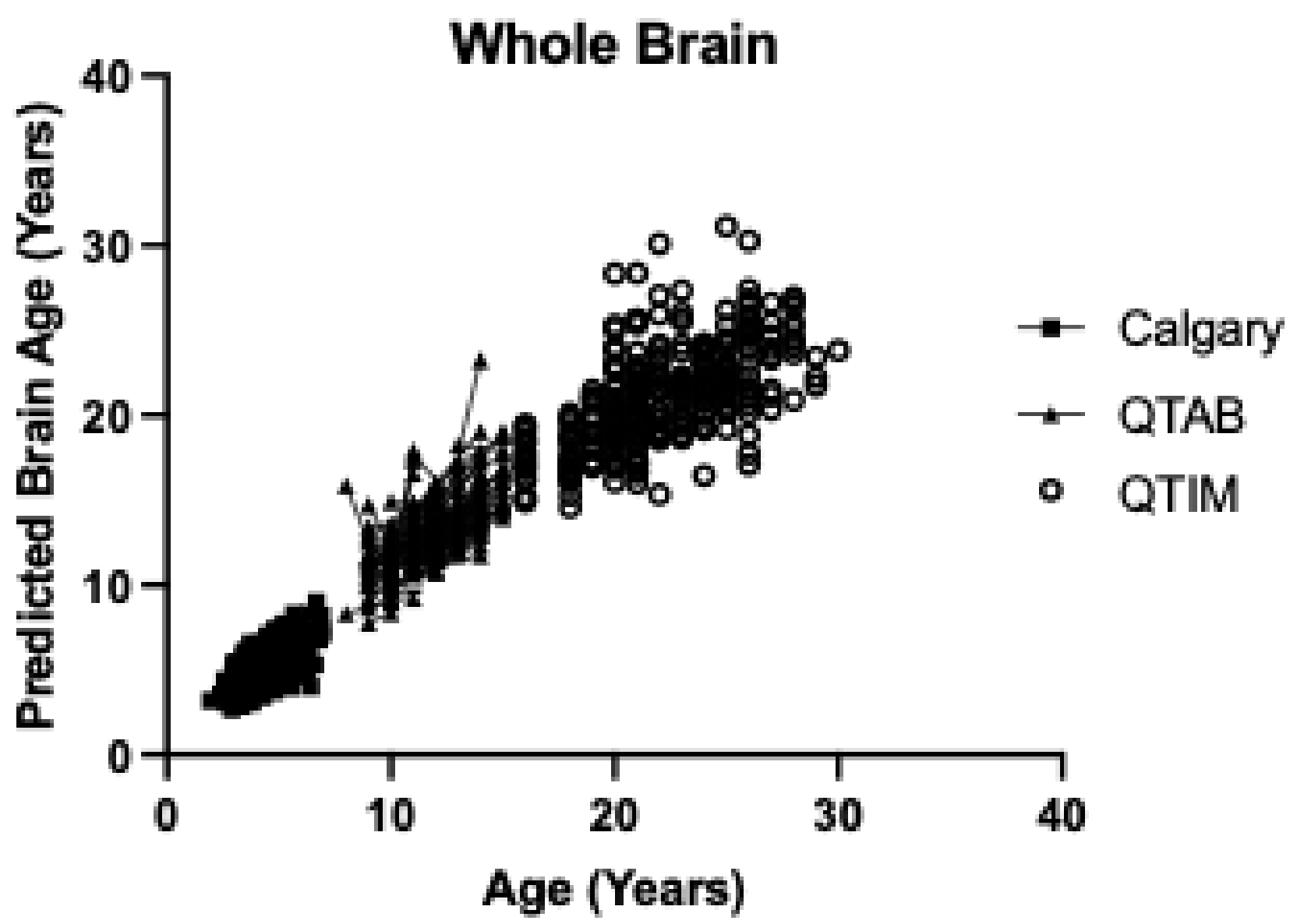

Supplement Figure 14. The relationship between predicted brain age and biological age in NC participants (897 MRI scans from 556 participants). Calgary participants are shown as squares, QTAB participants are shown as triangles, and QTIM participants are shown as open circles. Connecting lines indicate repeated scans on the same participant. Calgary participants showed an average increase of 0.77 (0.03) in predicted brain age per biological year, QTAB participants showed an average increase of 0.81 (0.05) in predicted brain age per biological year, and QTIM participants showed an average increase of 0.65 (0.03) in predicted brain age per biological year

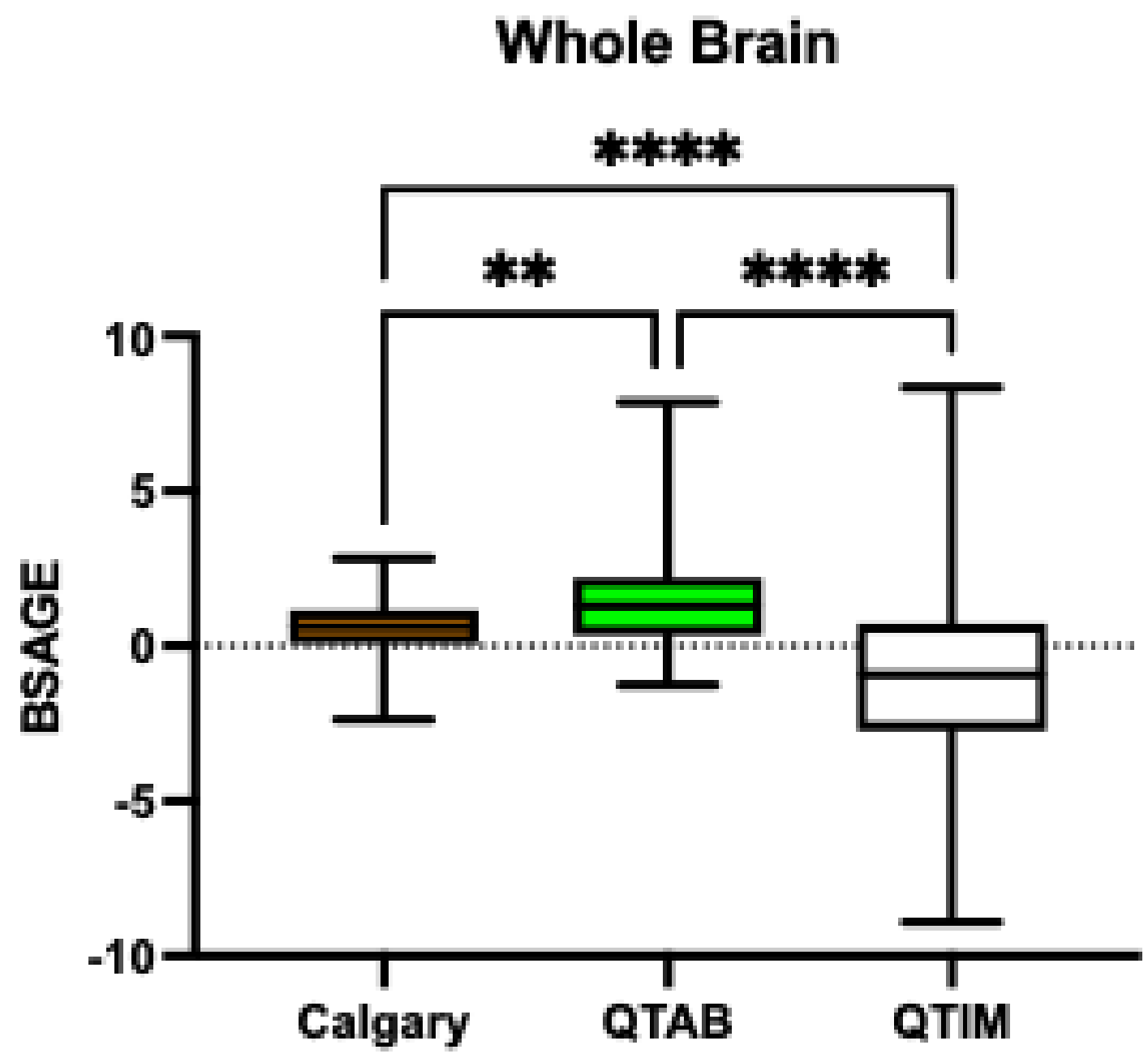

Supplement Figure 15. Comparison of Whole Brain BSAGE between NC participants (n = 556). Calgary participants had an average BSAGE = 0.63, QTAB participants had an average BSAGE = 1.48, and QTIM participants had an average BSAGE = -0.93. An analysis of variance (ANOVA) showed statistically significant ($F(2, 554) = 43.53$, $p < 0.0001$) differences in BSAGE between the three NC cohorts. A post-hoc Tukey test showed the QTAB cohort had a statistically significantly higher BSAGE compared to both Calgary ($p = 0.009$) and QTIM ($p < 0.0001$). The Calgary cohort also had a significantly higher BSAGE compared to QTIM ($p < 0.0001$).

Supplement F: Linear Mixed Effects Modeling (LMEM) Outputs

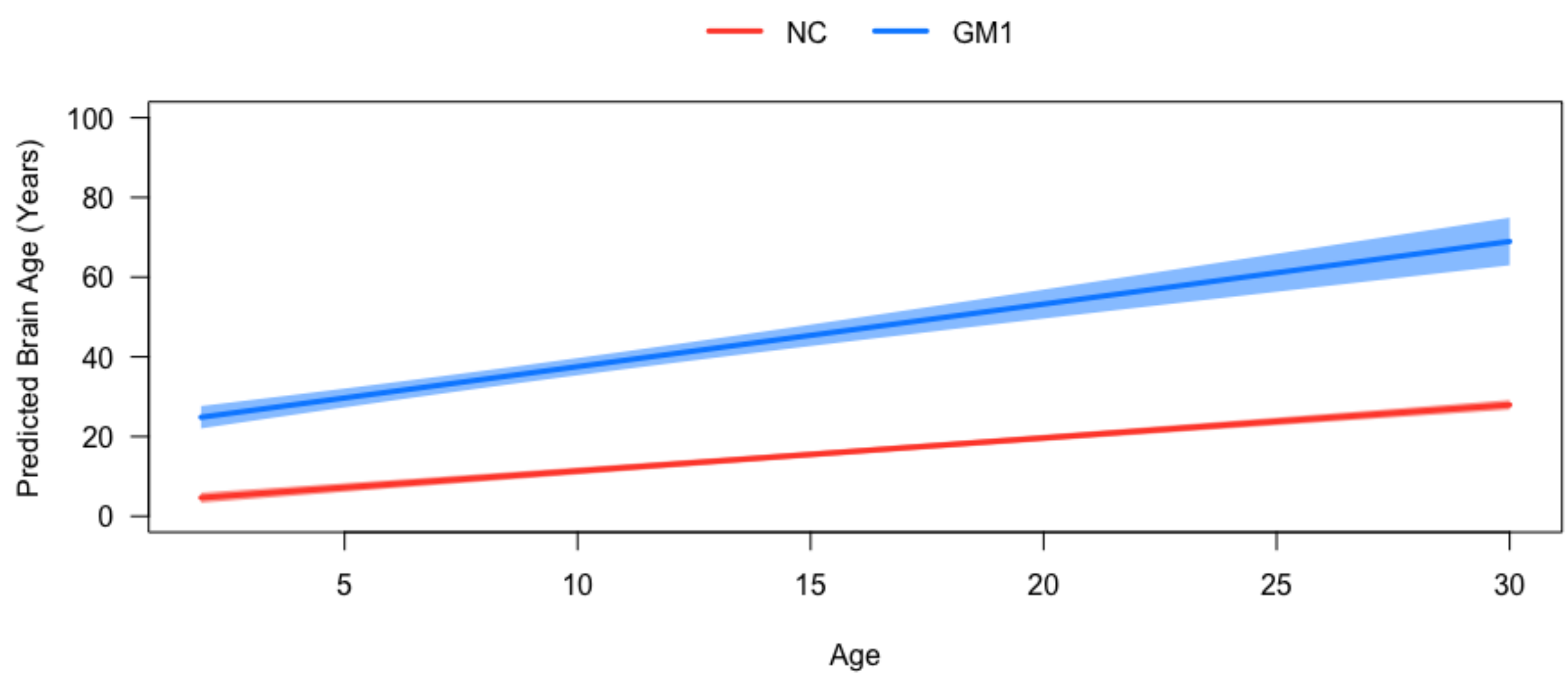

Supplement Figure 16. Comparisons of GM1 patients and neurotypical controls (NC) for predicted whole brain age as assessed by LMEM. The GM1 cohort is shown in blue, and the neurotypical controls are shown in red. The shaded regions represent the 95% confidence intervals calculated from the estimates and standards errors defined in Supplement Table 4.

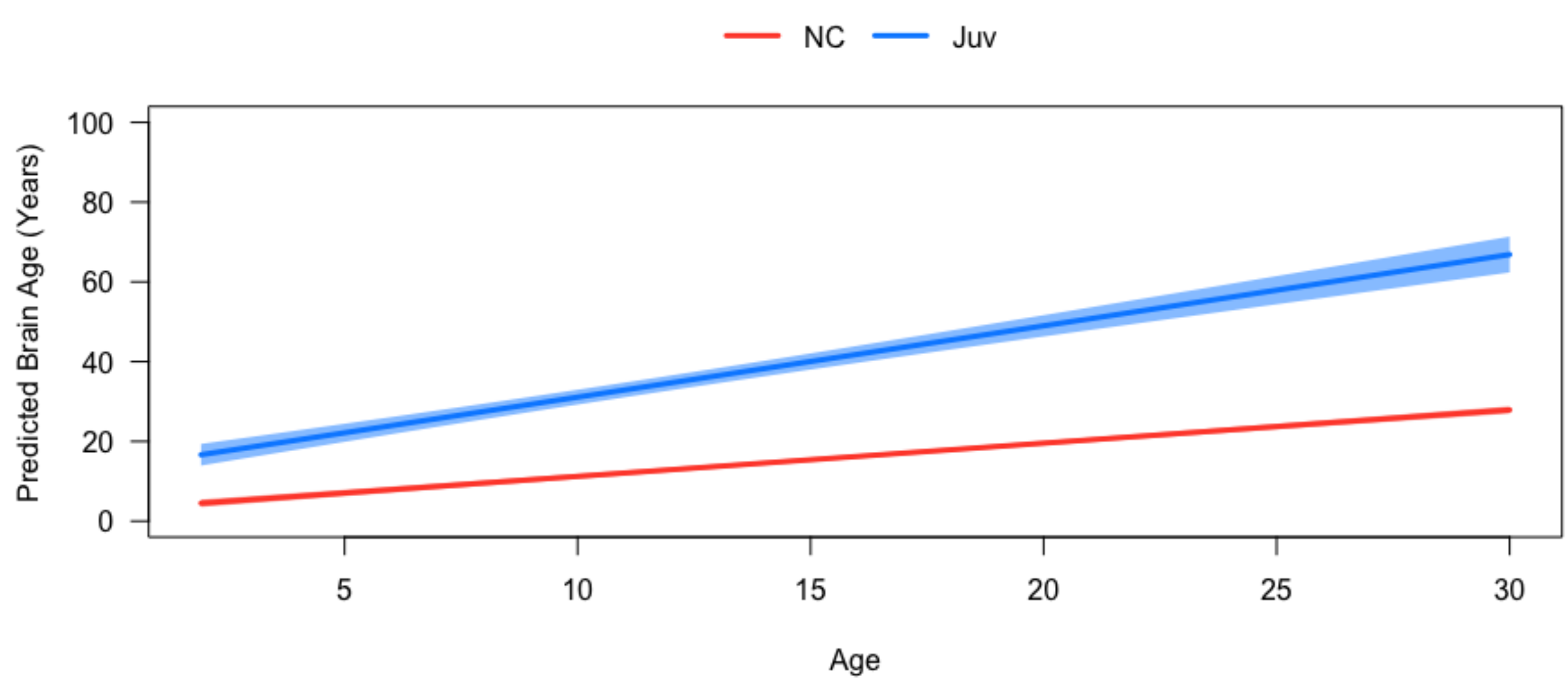

Supplement Figure 17. Comparisons of juvenile (Juv) GM1 patients and neurotypical controls (NC) for predicted whole brain age as assessed by LMEM. The juvenile GM1 cohort is shown in blue, and the NC are shown in red. The shaded regions represent the 95% confidence intervals calculated from the estimates and standards errors defined in Supplement Table 4.

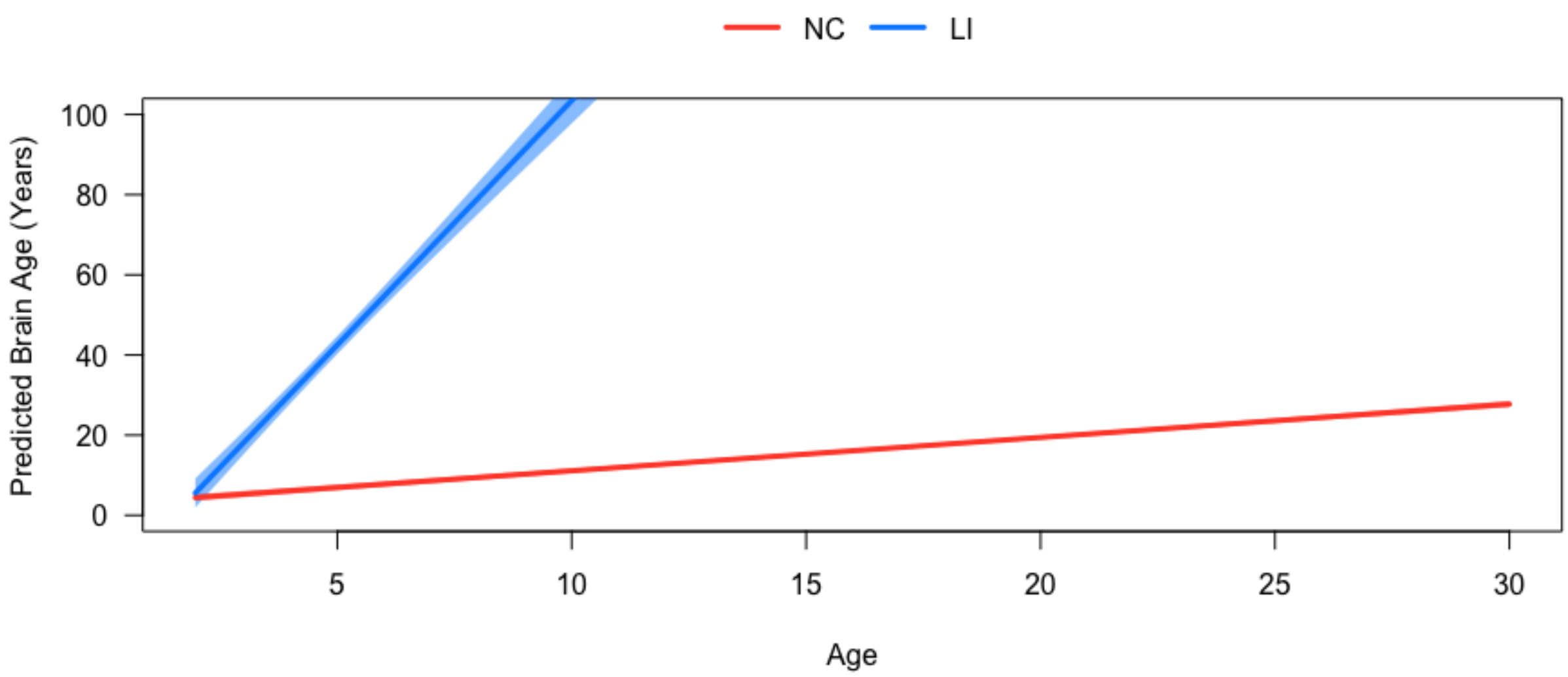

Supplement Figure 18. Comparisons of late-infantile GM1 patients and neurotypical controls for predicted whole brain age as assessed by LMEM. The late-infantile GM1 cohort is shown in blue, and the neurotypical controls are shown in red. The shaded regions represent the 95% confidence intervals calculated from the estimates and standards errors defined in Supplement Table 4.

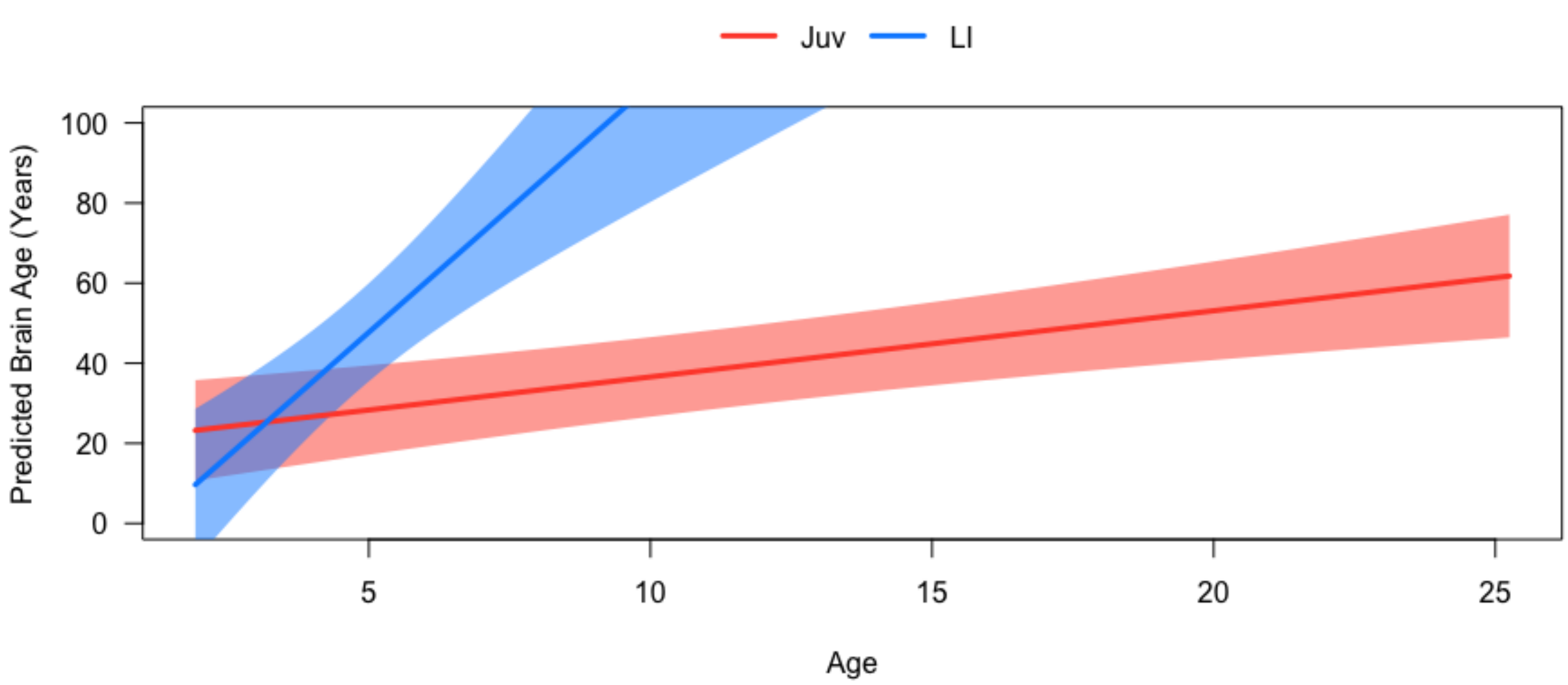

Supplement Figure 19. Comparisons of late-infantile GM1 patients and Juvenile (Juv) GM1 patients for predicted whole brain age as assessed by LMEM. The late-infantile GM1 cohort is shown in blue, and the juvenile GM1 patients are shown in red. The shaded regions represent the 95% confidence intervals calculated from the estimates and standards errors defined in Supplement Table 4.

Supplement G: Midbrain Stuctures Predicted Brain Age

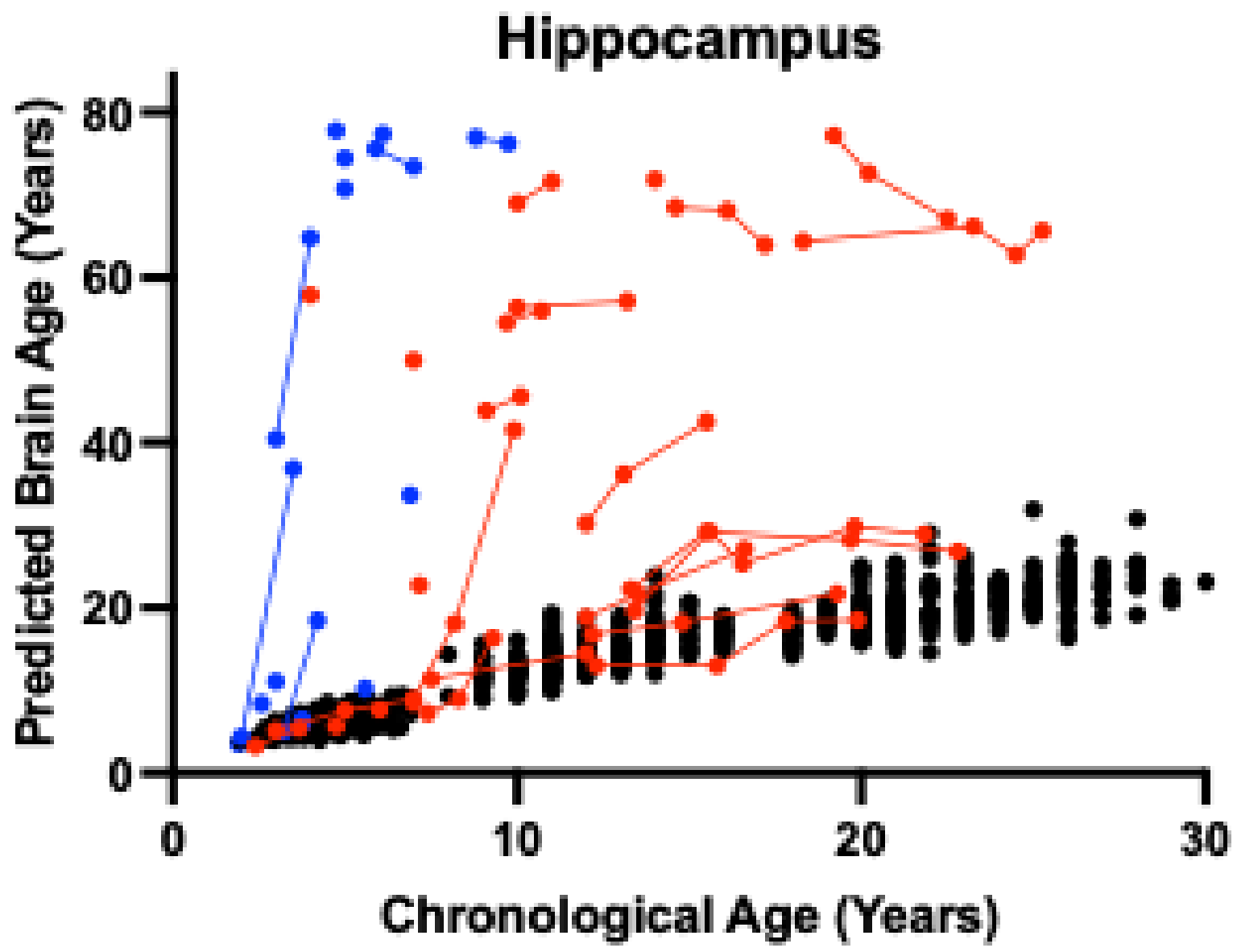

Supplement Figure 20. The relationship between predicted brain age and biological age in the hippocampus plotted against chronological age. Late-infantile (20 MRI scans from 15 patients) GM1 gangliosidosis patients are shown in blue, juvenile (61 MRI scans from 26 patients) GM1 gangliosidosis patients are shown in red, and neurotypical controls (897 MRI scans from 556 participants) participants are shown in black. Connecting lines indicate repeated scans on the same participant. Statistical analysis was performed using a linear mixed effects model to test the interactions between chronological age and cohort on predicted brain age and the results are summarized in Table I, with the estimated and standard errors shown in Supplement Table 4.

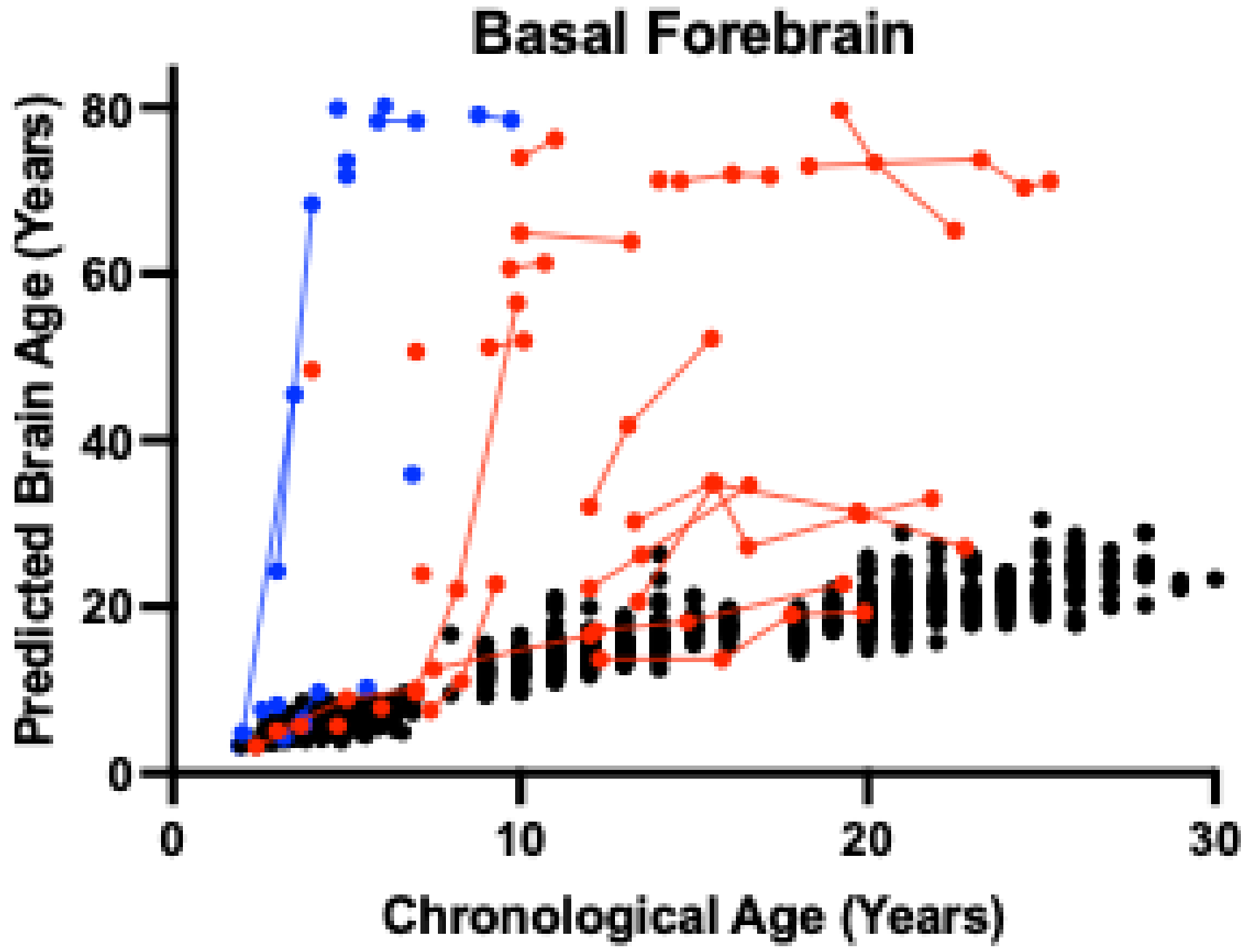

Supplement Figure 21. The relationship between predicted brain age and biological age in the basal forebrain plotted against chronological age. Late-infantile (20 MRI scans from 15 patients) GM1 gangliosidosis patients are shown in blue, juvenile (61 MRI scans from 26 patients) GM1 gangliosidosis patients are shown in red, and neurotypical controls (897 MRI scans from 556 participants) participants are shown in black. Connecting lines indicate repeated scans on the same participant. Statistical analysis was performed using a linear mixed effects model to test the interactions between chronological age and cohort on predicted brain age and the results are summarized in Supplement Table 3, with the estimated and standard errors shown in Supplement Table 4.

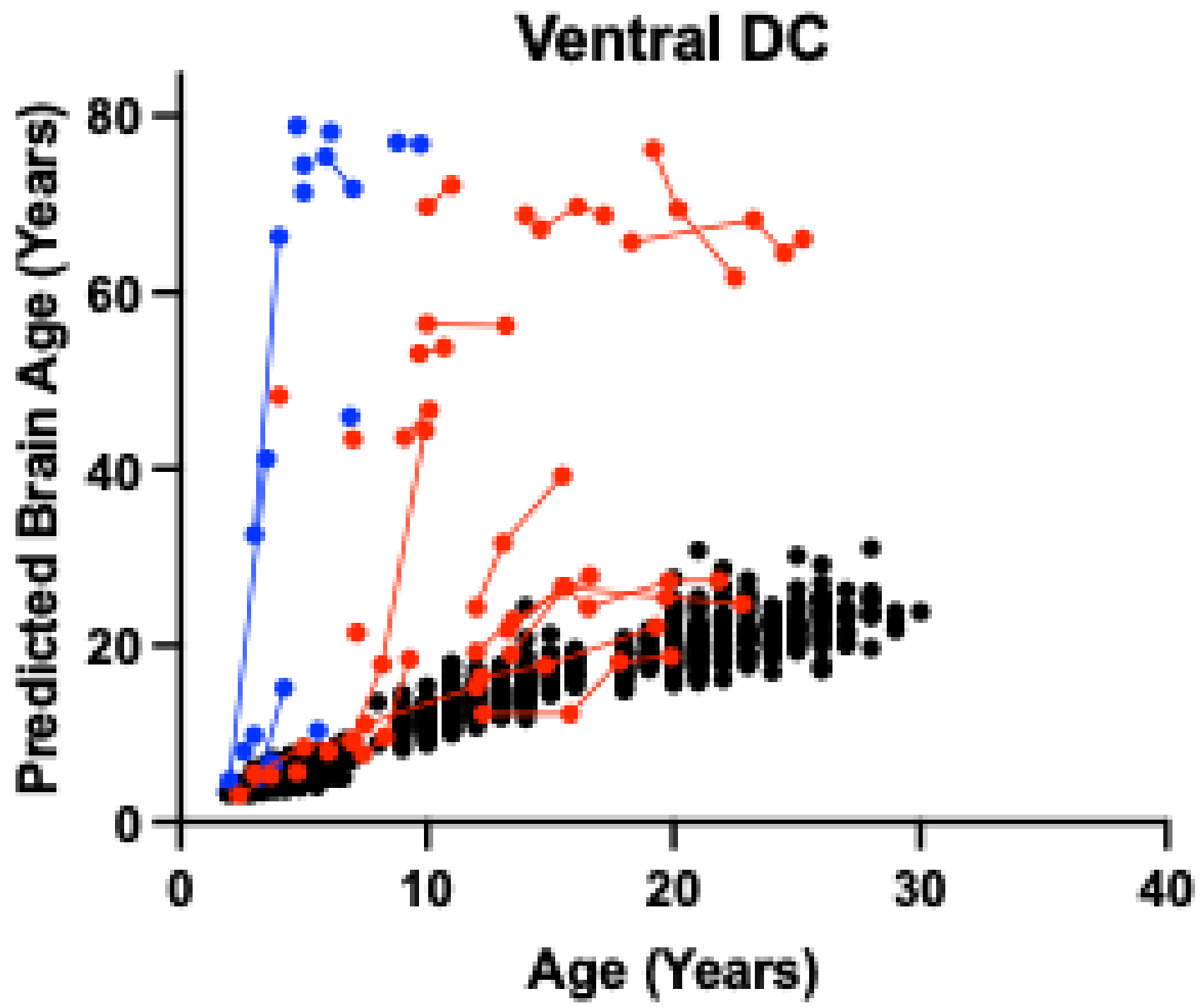

Supplement Figure 22. The relationship between predicted brain age and biological age in the ventral diencephalon (DC) plotted against chronological age. Late-infantile (20 MRI scans from 15 patients) GM1 gangliosidosis patients are shown in blue, juvenile (61 MRI scans from 26 patients) GM1 gangliosidosis patients are shown in red, and neurotypical controls (897 MRI scans from 556 participants) participants are shown in black. Connecting lines indicate repeated scans on the same participant. Statistical analysis was performed using a linear mixed effects model to test the interactions between chronological age and cohort on predicted brain age and the results are summarized in Table I, with the estimated and standard errors shown in Supplement Table 4.

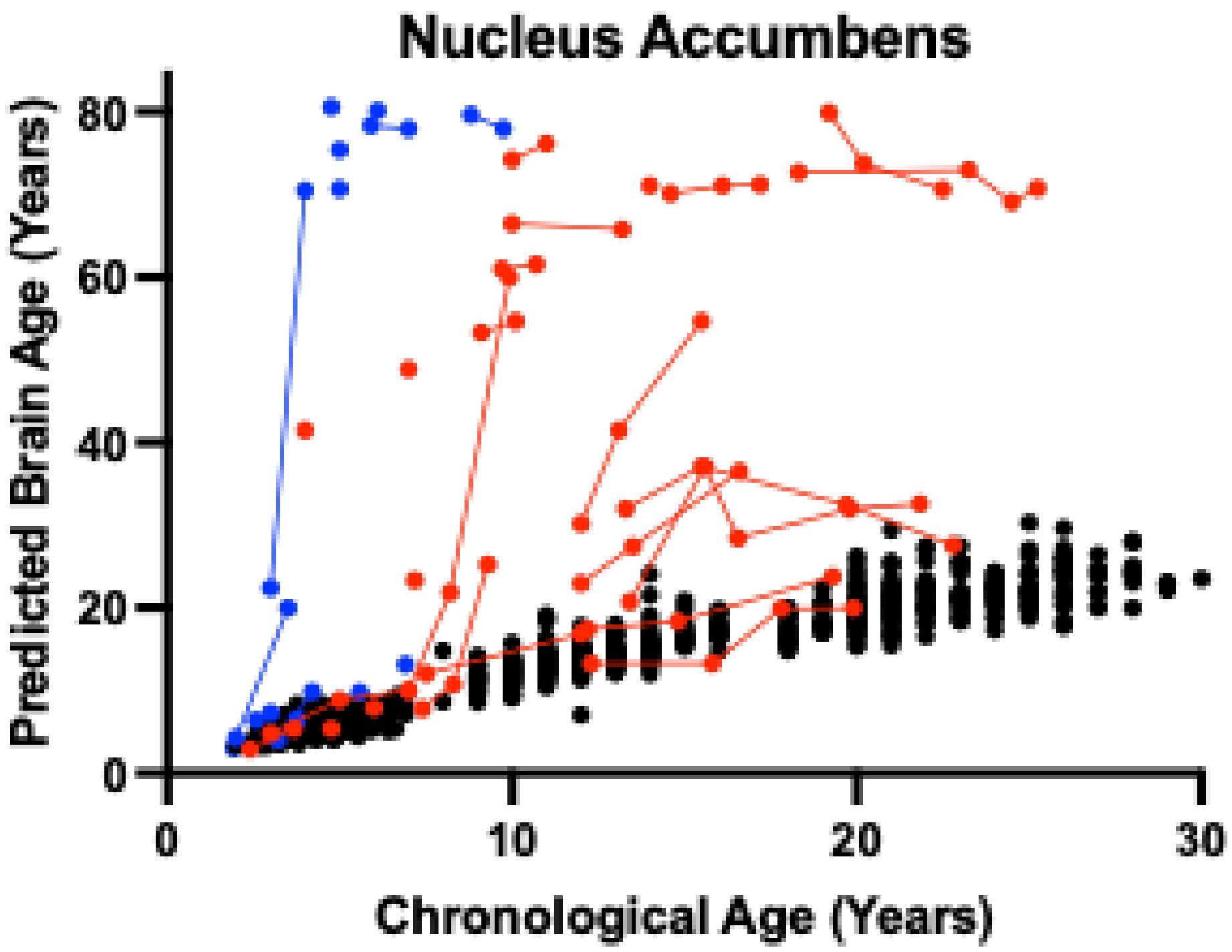

Supplement Figure 23. The relationship between predicted brain age and biological age in the nucleus accumbens plotted against chronological age. Late-infantile (20 MRI scans from 15 patients) GM1 gangliosidosis patients are shown in blue, juvenile (61 MRI scans from 26 patients) GM1 gangliosidosis patients are shown in red, and neurotypical controls (897 MRI scans from 556 participants) participants are shown in black. Connecting lines indicate repeated scans on the same participant. Statistical analysis was performed using a linear mixed effects model to test the interactions between chronological age and cohort on predicted brain age and the results are summarized in Supplement Table 3, with the estimated and standard errors shown in Supplement Table 4.

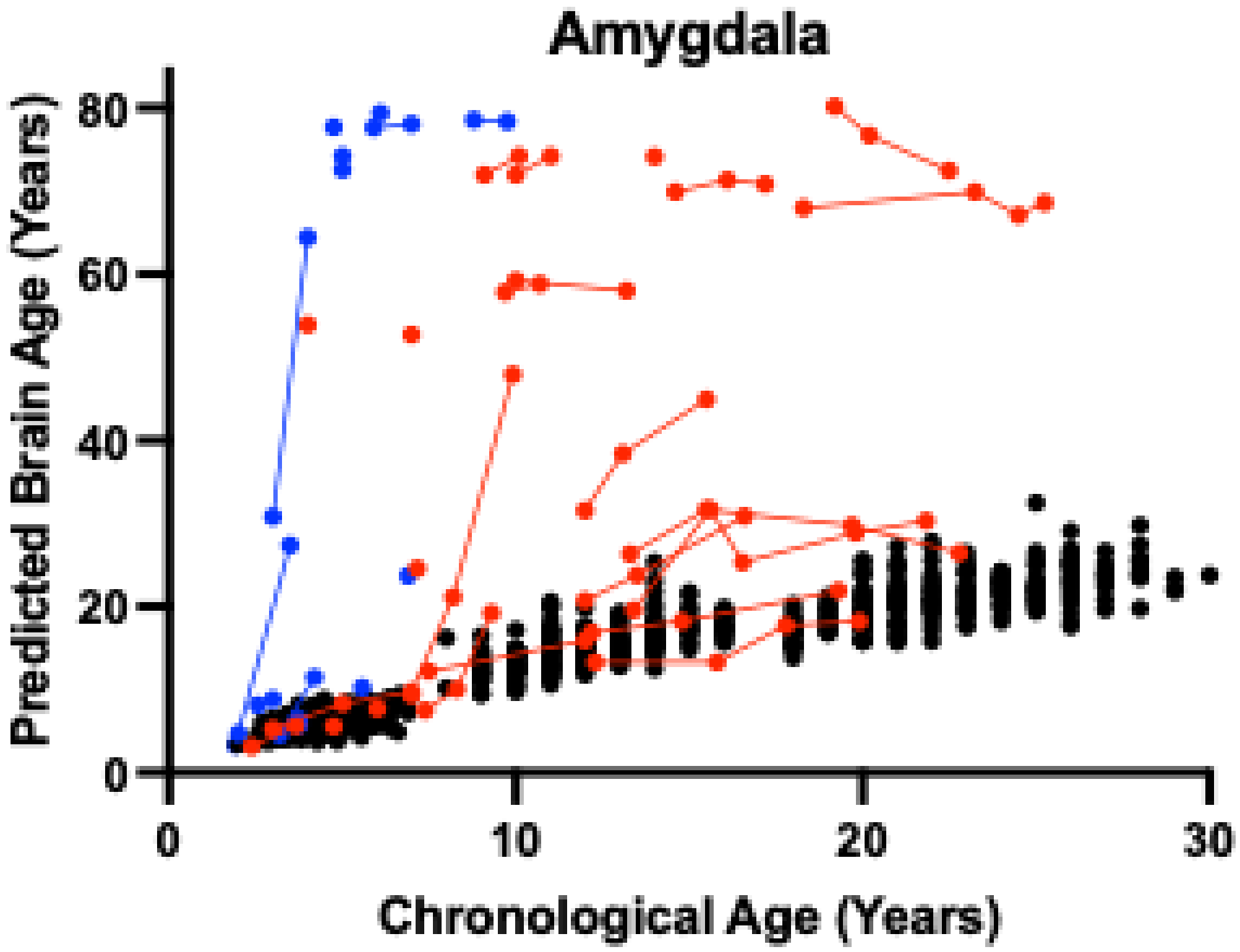

Supplement Figure 24. The relationship between predicted brain age and biological age in the amygdala plotted against chronological age. Late-infantile (20 MRI scans from 15 patients) GM1 gangliosidosis patients are shown in blue, juvenile (61 MRI scans from 26 patients) GM1 gangliosidosis patients are shown in red, and neurotypical controls (897 MRI scans from 556 participants) participants are shown in black. Connecting lines indicate repeated scans on the same participant. Statistical analysis was performed using a linear mixed effects model to test the interactions between chronological age and cohort on predicted brain age and the results are summarized in Supplement Table 3, with the estimated and standard errors shown in Supplement Table 4.

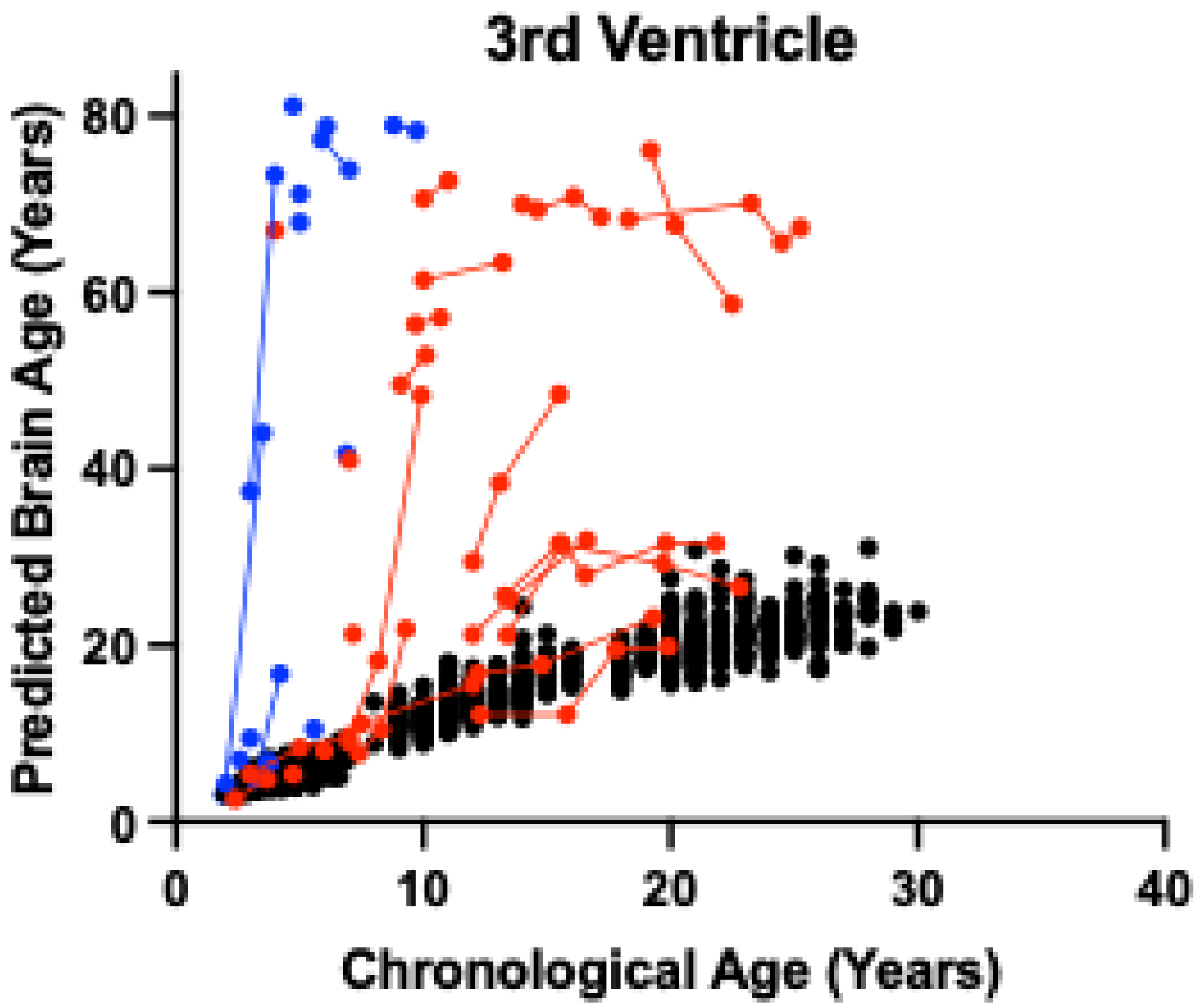

Supplement Figure 25. The relationship between predicted brain age and biological age in the 3rd ventricle plotted against chronological age. Late-infantile (20 MRI scans from 15 patients) GM1 gangliosidosis patients are shown in blue, juvenile (61 MRI scans from 26 patients) GM1 gangliosidosis patients are shown in red, and neurotypical controls (897 MRI scans from 556 participants) participants are shown in black. Connecting lines indicate repeated scans on the same participant. Statistical analysis was performed using a linear mixed effects model to test the interactions between chronological age and cohort on predicted brain age and the results are summarized in Table I, with the estimated and standard errors shown in Supplement Table 4.

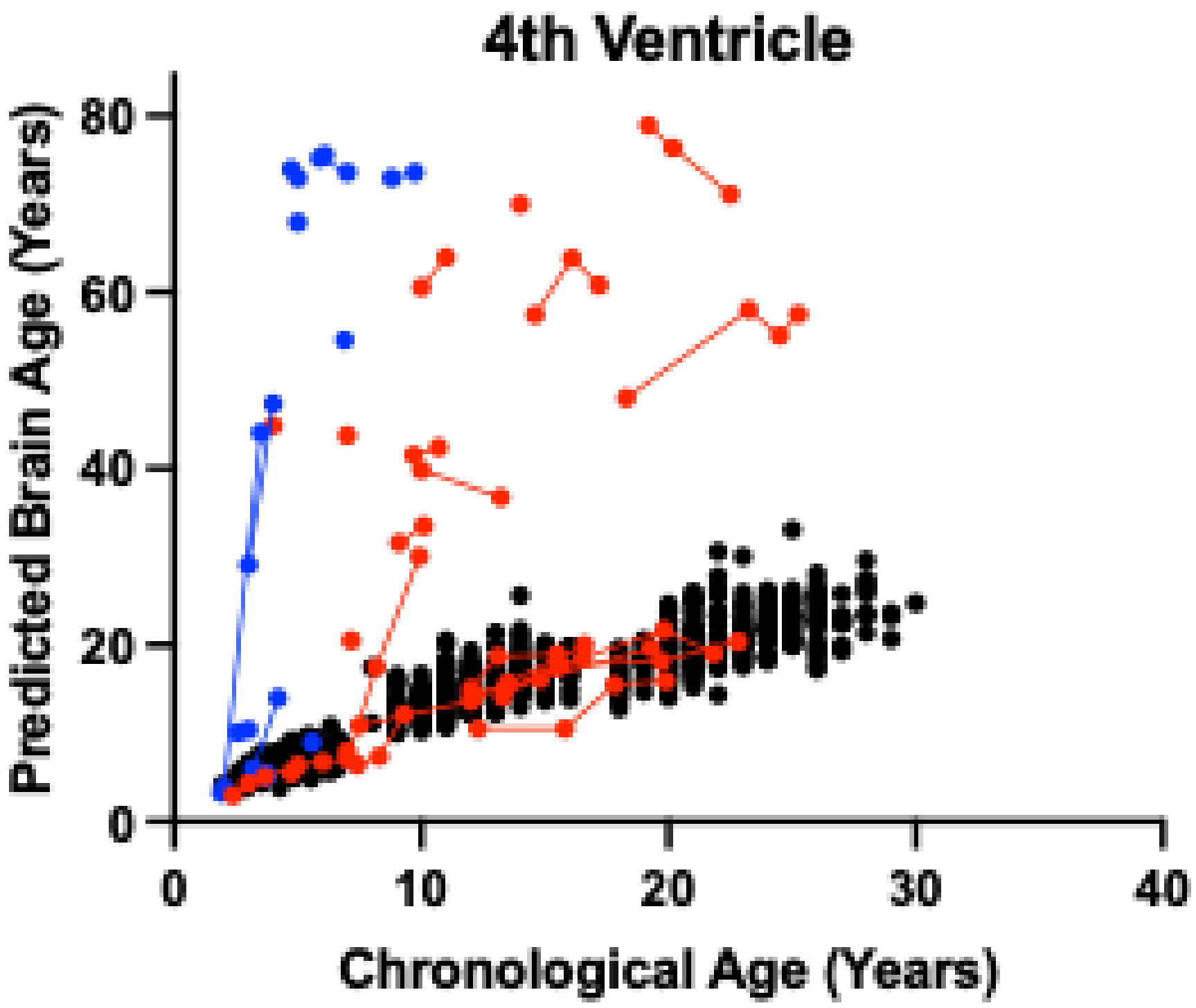

Supplement Figure 26. The relationship between predicted brain age and biological age in the 4th ventricle plotted against chronological age. Late-infantile (20 MRI scans from 15 patients) GM1 gangliosidosis patients are shown in blue, juvenile (61 MRI scans from 26 patients) GM1 gangliosidosis patients are shown in red, and neurotypical controls (897 MRI scans from 556 participants) participants are shown in black. Connecting lines indicate repeated scans on the same participant. Statistical analysis was performed using a linear mixed effects model to test the interactions between chronological age and cohort on predicted brain age and the results are summarized in Table I, with the estimated and standard errors shown in Supplement Table 4.

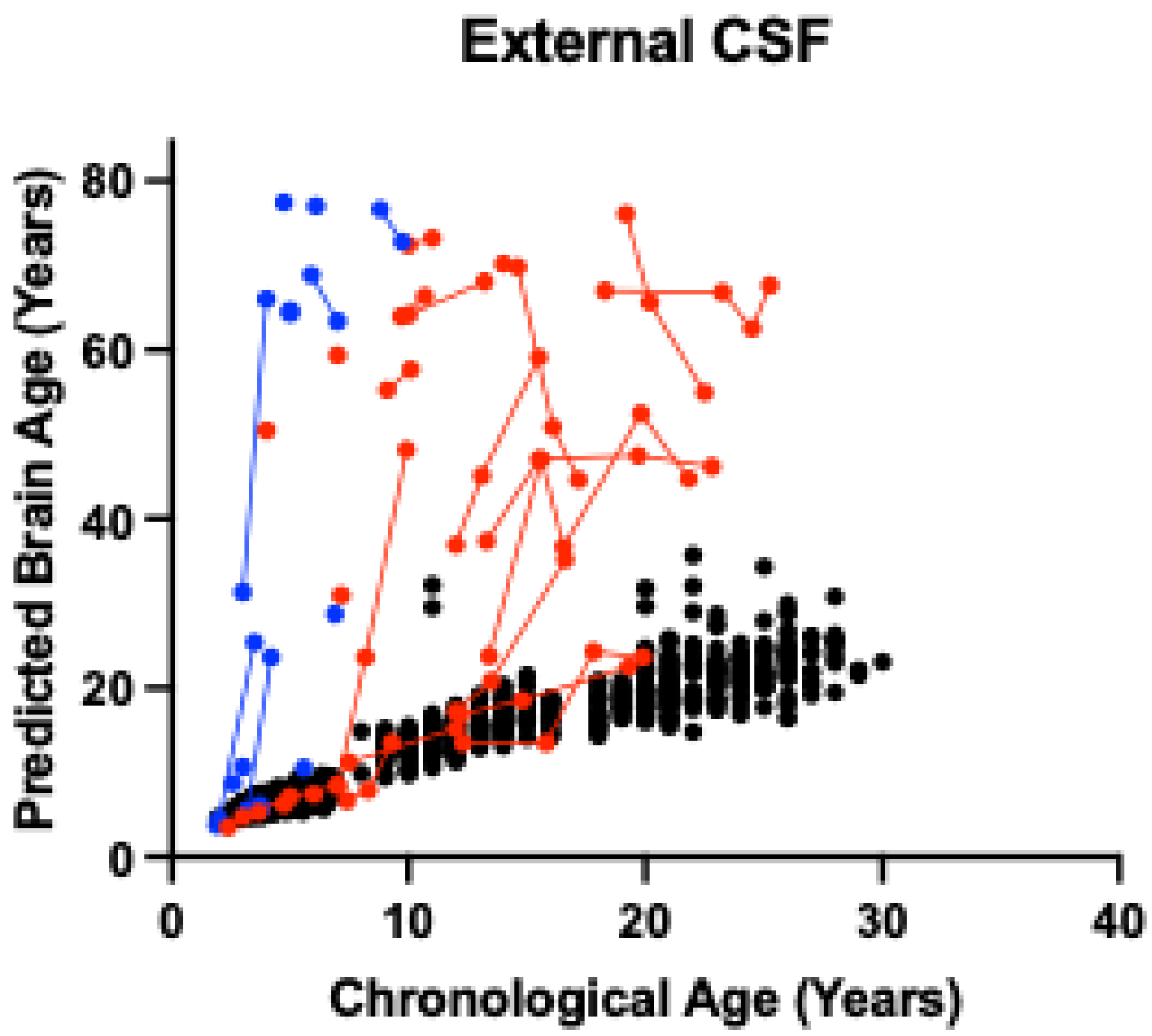

Supplement Figure 27. The relationship between predicted brain age and biological age in the external cerebrospinal fluid plotted against chronological age. Late-infantile (20 MRI scans from 15 patients) GM1 gangliosidosis patients are shown in blue, juvenile (61 MRI scans from 26 patients) GM1 gangliosidosis patients are shown in red, and neurotypical controls (897 MRI scans from 556 participants) participants are shown in black. Connecting lines indicate repeated scans on the same participant. Statistical analysis was performed using a linear mixed effects model to test the interactions between chronological age and cohort on predicted brain age and the results are summarized in Table I, with the estimated and standard errors shown in Supplement Table 4.

Supplement H: Longitudinal Brain structures age gap estimation (BSAGE)

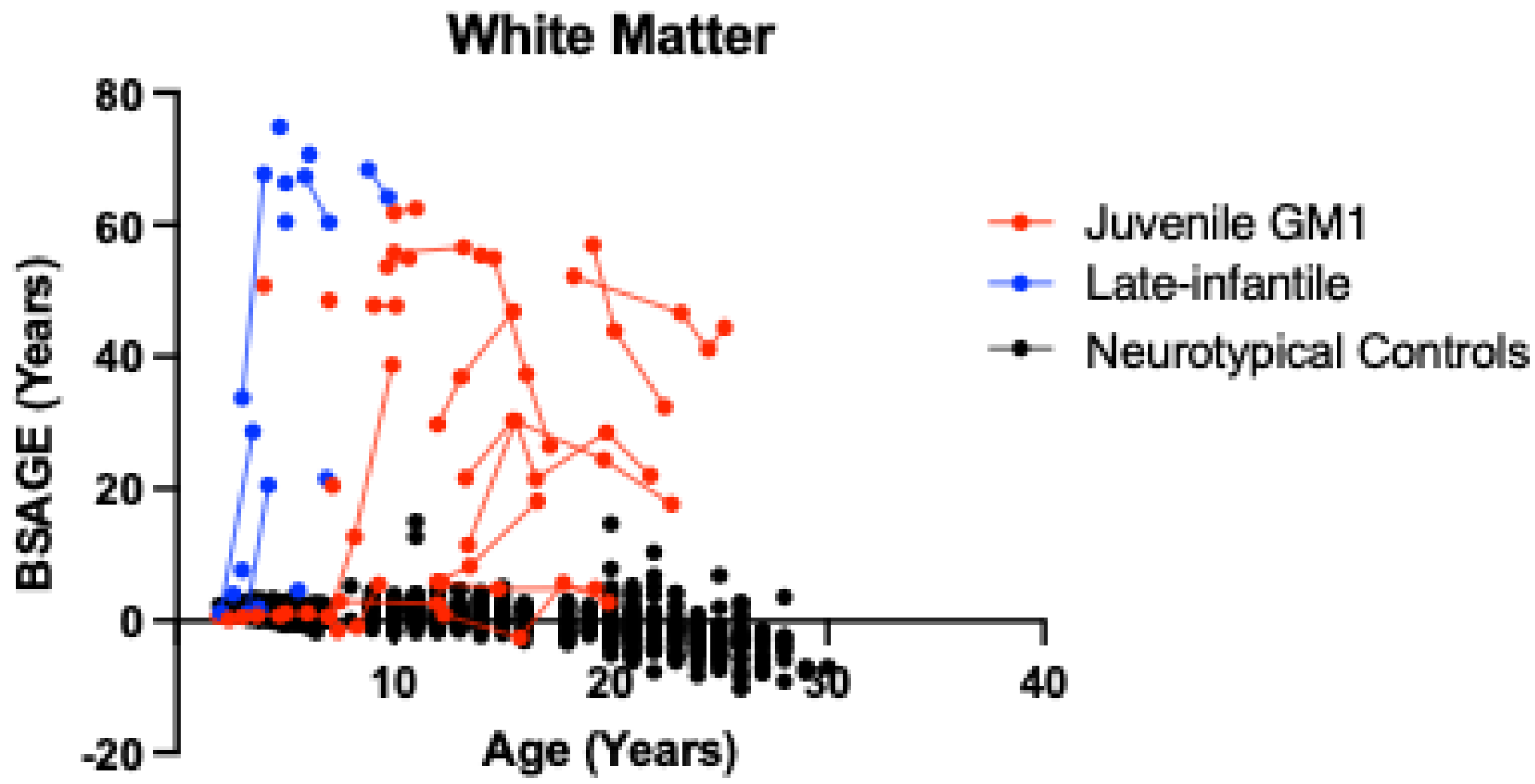

Supplement Figure 28. The longitudinal relationship between white matter BSAGE and chronological age. Late-infantile GM1 gangliosidosis patients are shown in blue, juvenile GM1 gangliosidosis patients are shown in red, and neurotypical controls participants are shown in black. Connecting lines indicate repeated scans on the same participant.

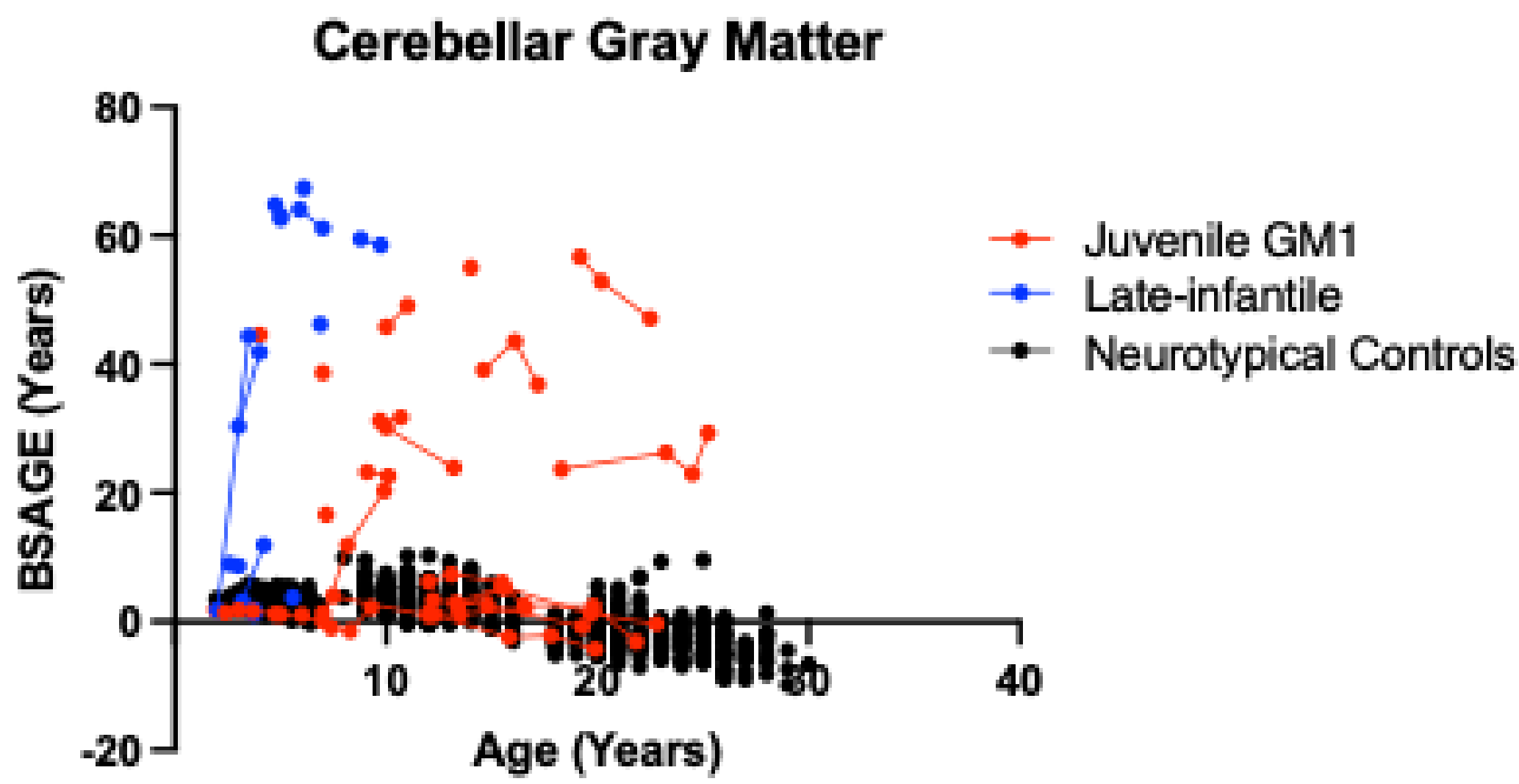

Supplement Figure 29. The longitudinal relationship between cerebellar gray matter BSAGE and chronological age. Late-infantile GM1 gangliosidosis patients are shown in blue, juvenile GM1 gangliosidosis patients are shown in red, and neurotypical controls participants are shown in black. Connecting lines indicate repeated scans on the same participant.

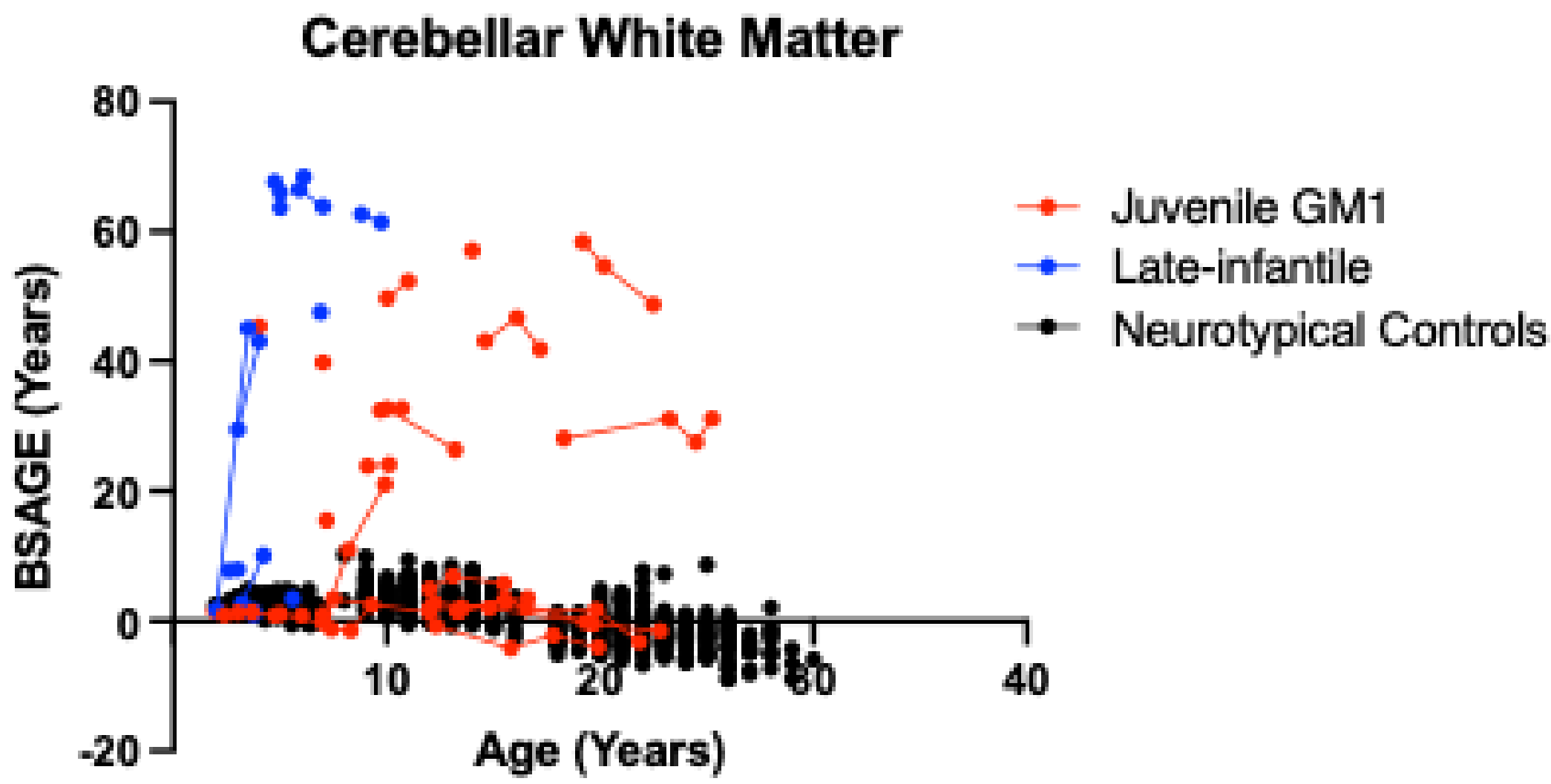

Supplement Figure 30. The longitudinal relationship between cerebellar white matter BSAGE and chronological age. Late-infantile GM1 gangliosidosis patients are shown in blue, juvenile GM1 gangliosidosis patients are shown in red, and neurotypical controls participants are shown in black. Connecting lines indicate repeated scans on the same participant.

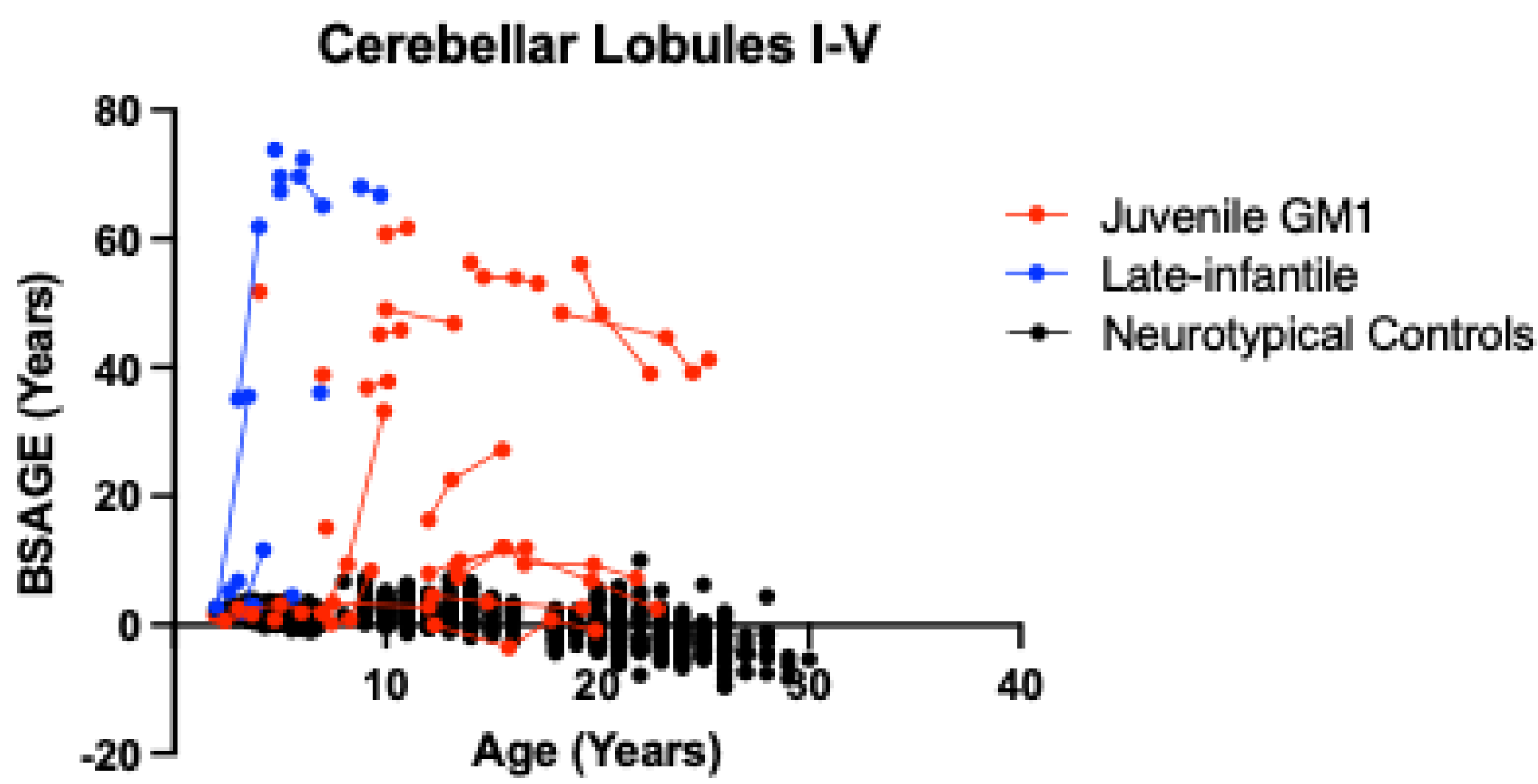

Supplement Figure 31. The longitudinal relationship between cerebellar lobules I-V BSAGE and chronological age. Late-infantile GM1 gangliosidosis patients are shown in blue, juvenile GM1 gangliosidosis patients are shown in red, and neurotypical controls participants are shown in black. Connecting lines indicate repeated scans on the same participant.

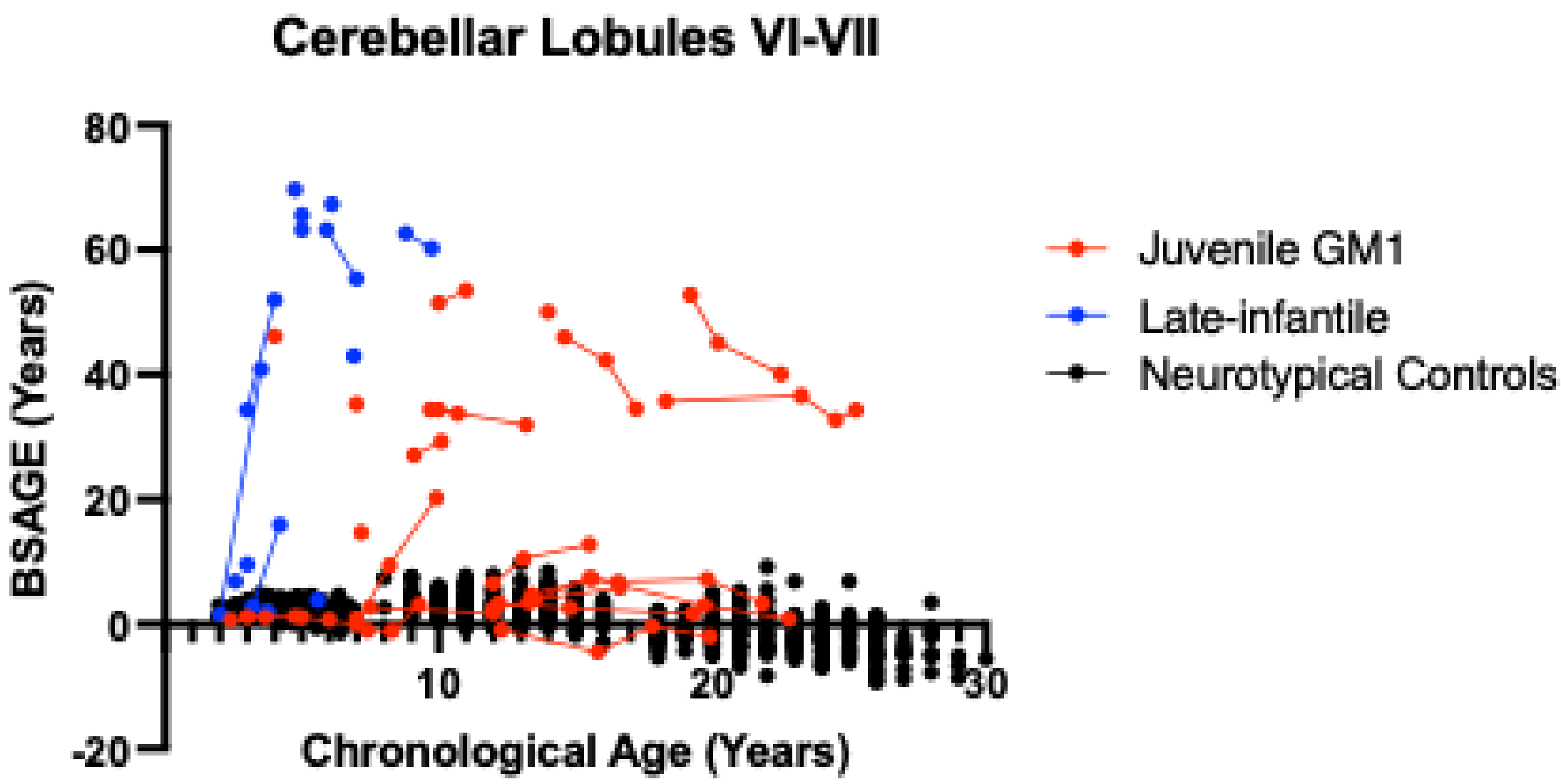

Supplement Figure 32. The longitudinal relationship between cerebellar lobules VI-VII BSAGE and chronological age. Late-infantile GM1 gangliosidosis patients are shown in blue, juvenile GM1 gangliosidosis patients are shown in red, and neurotypical controls participants are shown in black. Connecting lines indicate repeated scans on the same participant.

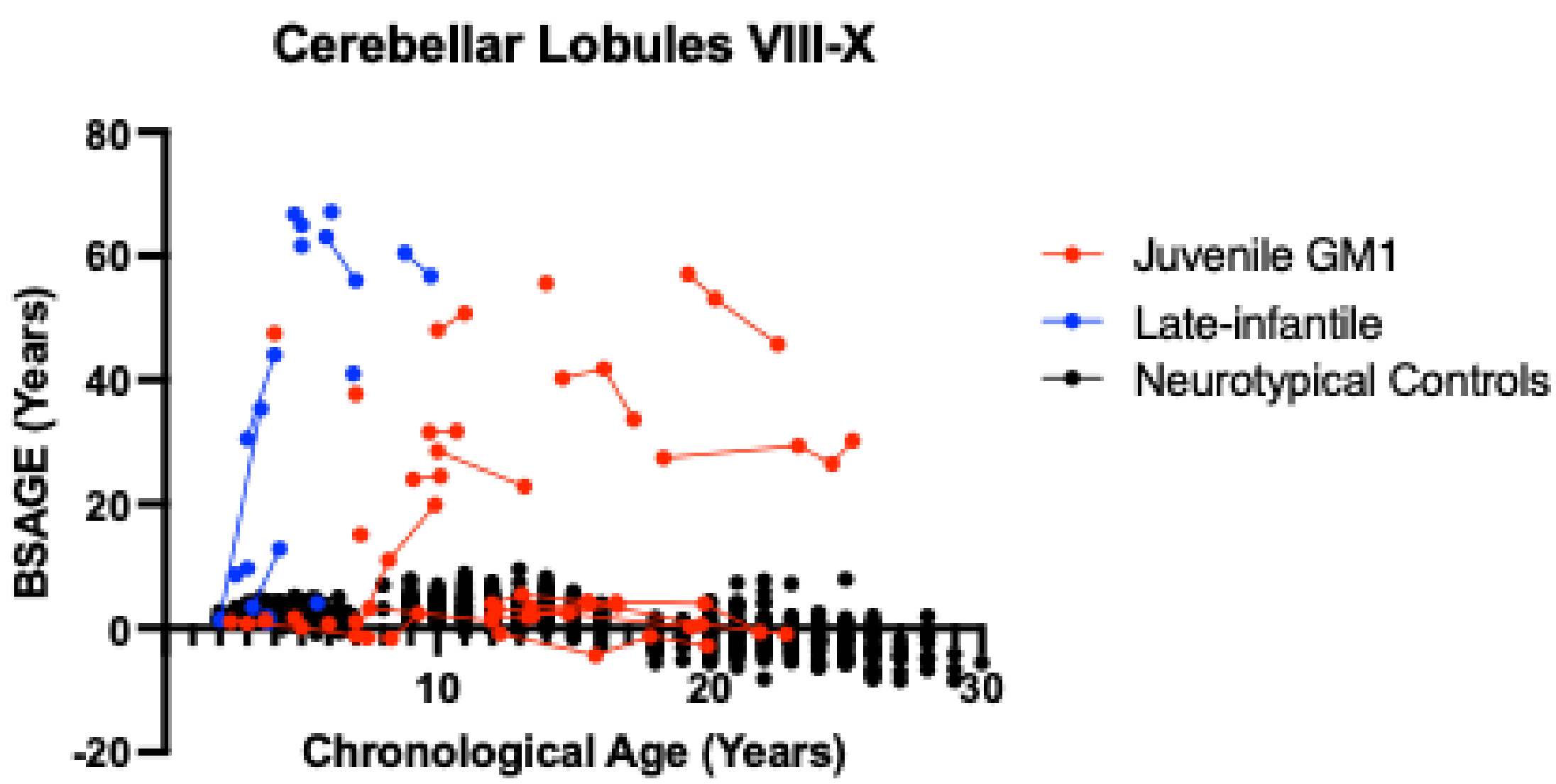

Supplement Figure 33. The longitudinal relationship between cerebellar lobules VIII-X BSAGE and chronological age. Late-infantile GM1 gangliosidosis patients are shown in blue, juvenile GM1 gangliosidosis patients are shown in red, and neurotypical controls participants are shown in black. Connecting lines indicate repeated scans on the same participant.

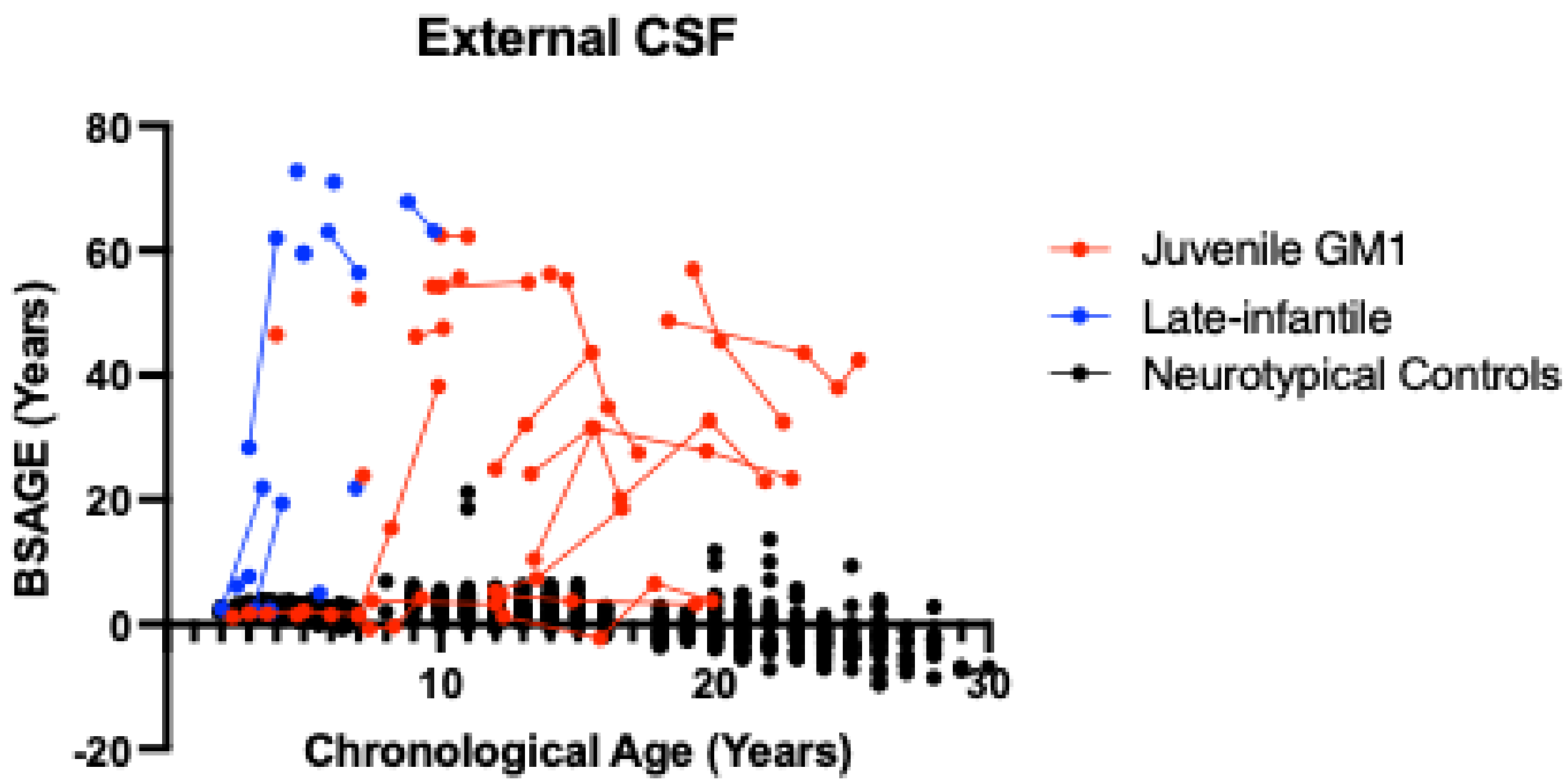

Supplement Figure 34. The longitudinal relationship between external cerebrospinal fluid (CSF) BSAGE and chronological age. Late-infantile GM1 gangliosidosis patients are shown in blue, juvenile GM1 gangliosidosis patients are shown in red, and neurotypical controls participants are shown in black. Connecting lines indicate repeated scans on the same participant.

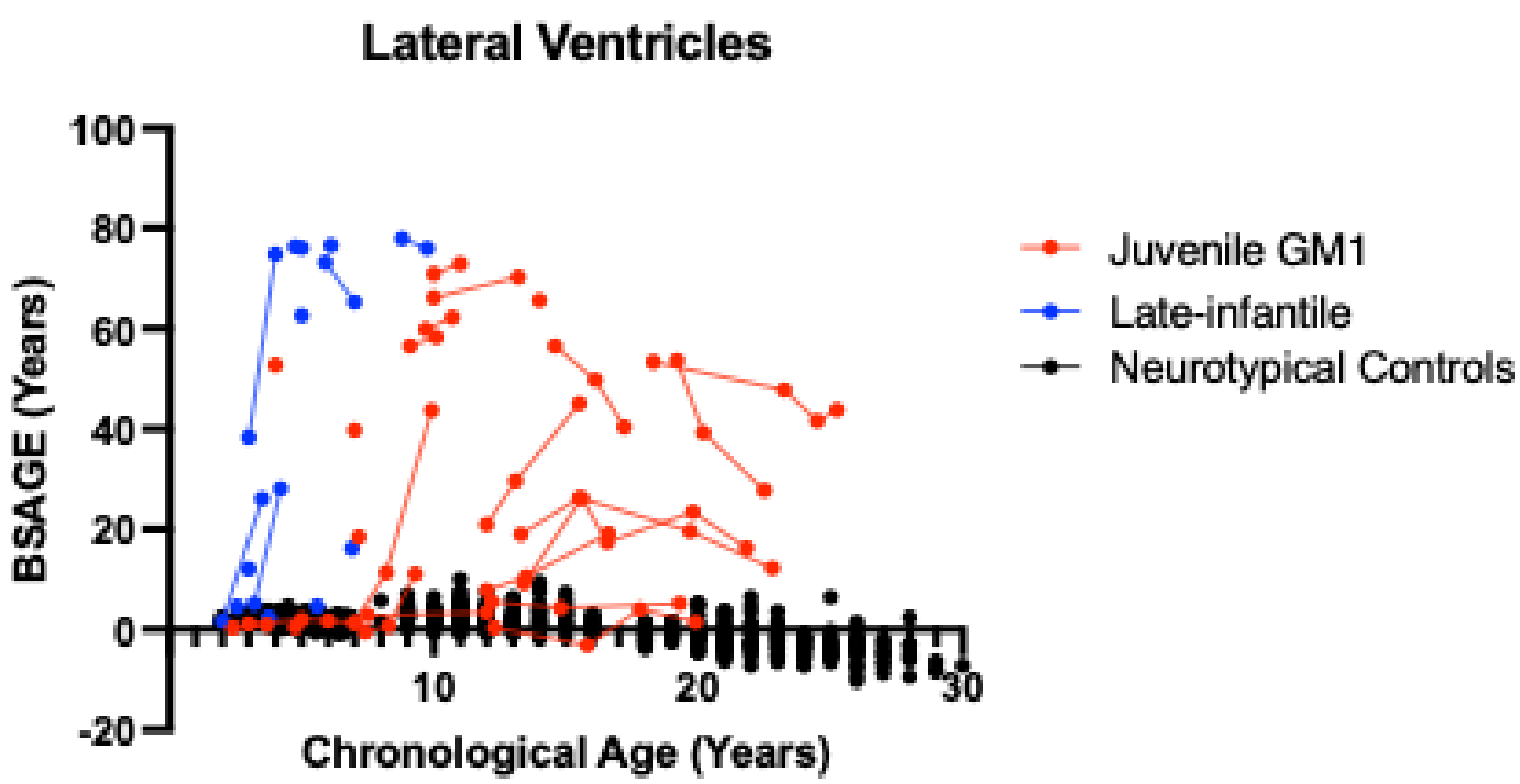

Supplement Figure 35. The longitudinal relationship between lateral ventricles BSAGE and chronological age. Late-infantile GM1 gangliosidosis patients are shown in blue, juvenile GM1 gangliosidosis patients are shown in red, and neurotypical controls participants are shown in black. Connecting lines indicate repeated scans on the same participant.

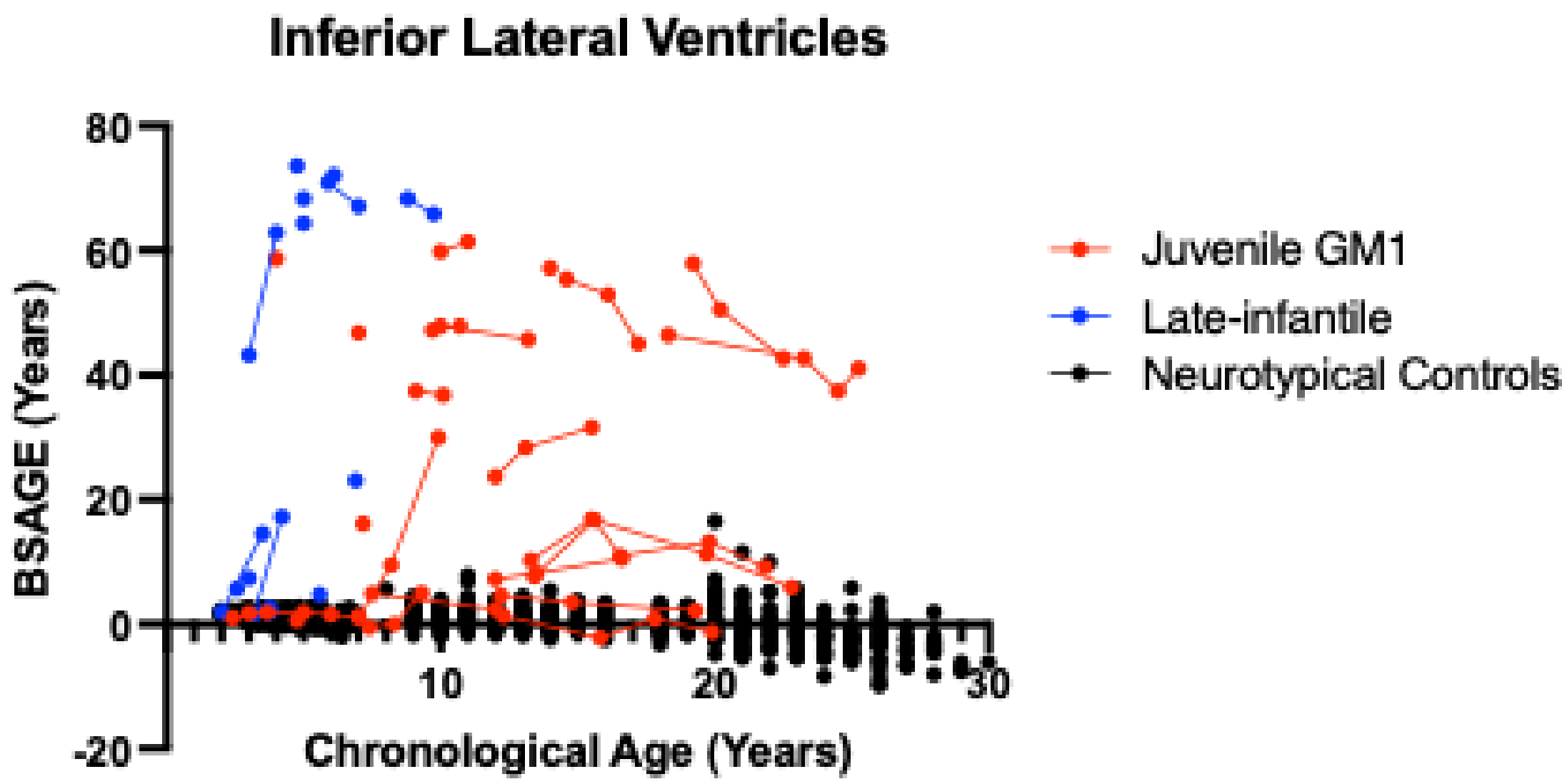

Supplement Figure 36. The longitudinal relationship between inferior lateral ventricles BSAGE and chronological age. Late-infantile GM1 gangliosidosis patients are shown in blue, juvenile GM1 gangliosidosis patients are shown in red, and neurotypical controls participants are shown in black. Connecting lines indicate repeated scans on the same participant.

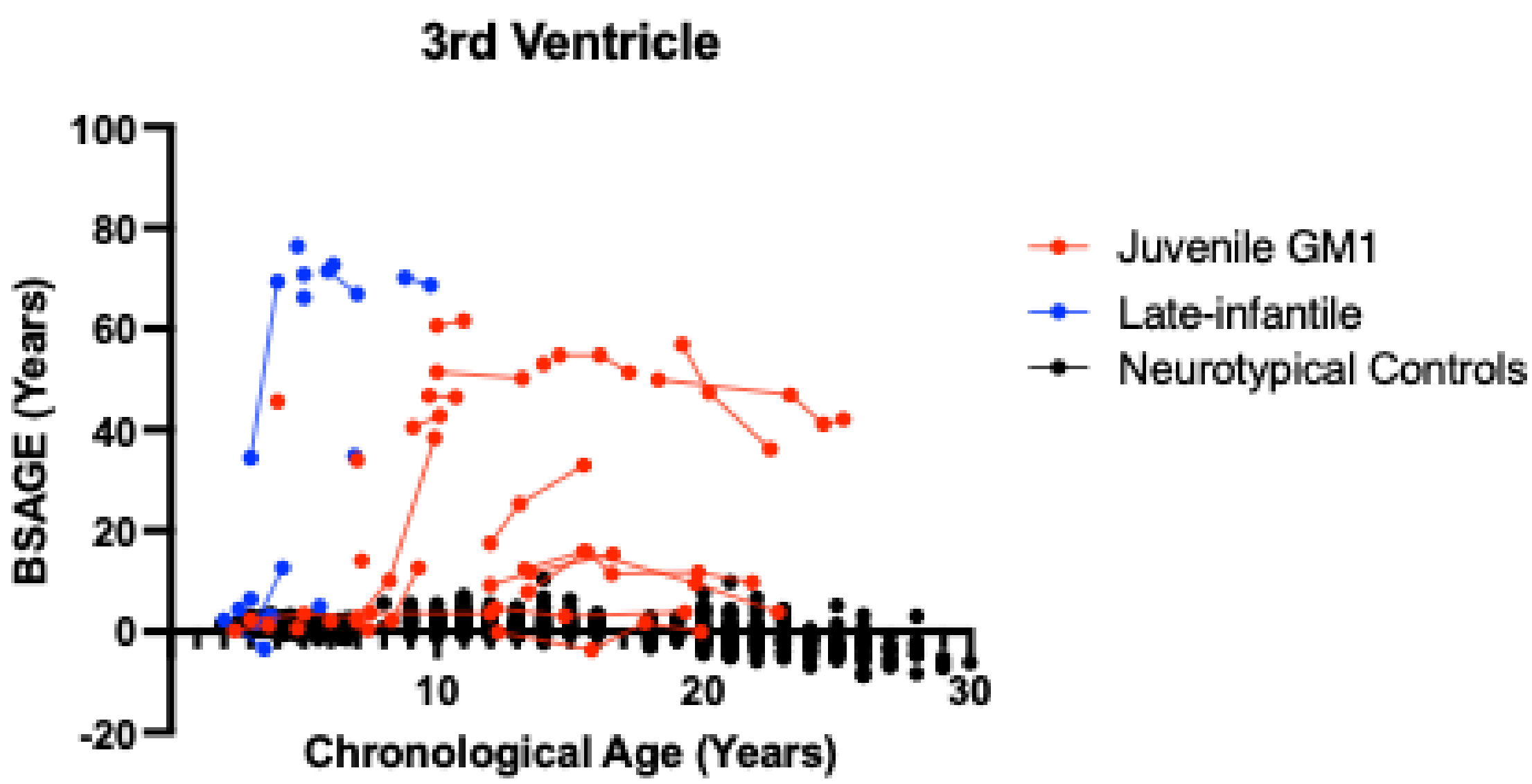

Supplement Figure 37. The longitudinal relationship between 3rd ventricle BSAGE and chronological age. Late-infantile GM1 gangliosidosis patients are shown in blue, juvenile GM1 gangliosidosis patients are shown in red, and neurotypical controls participants are shown in black. Connecting lines indicate repeated scans on the same participant.

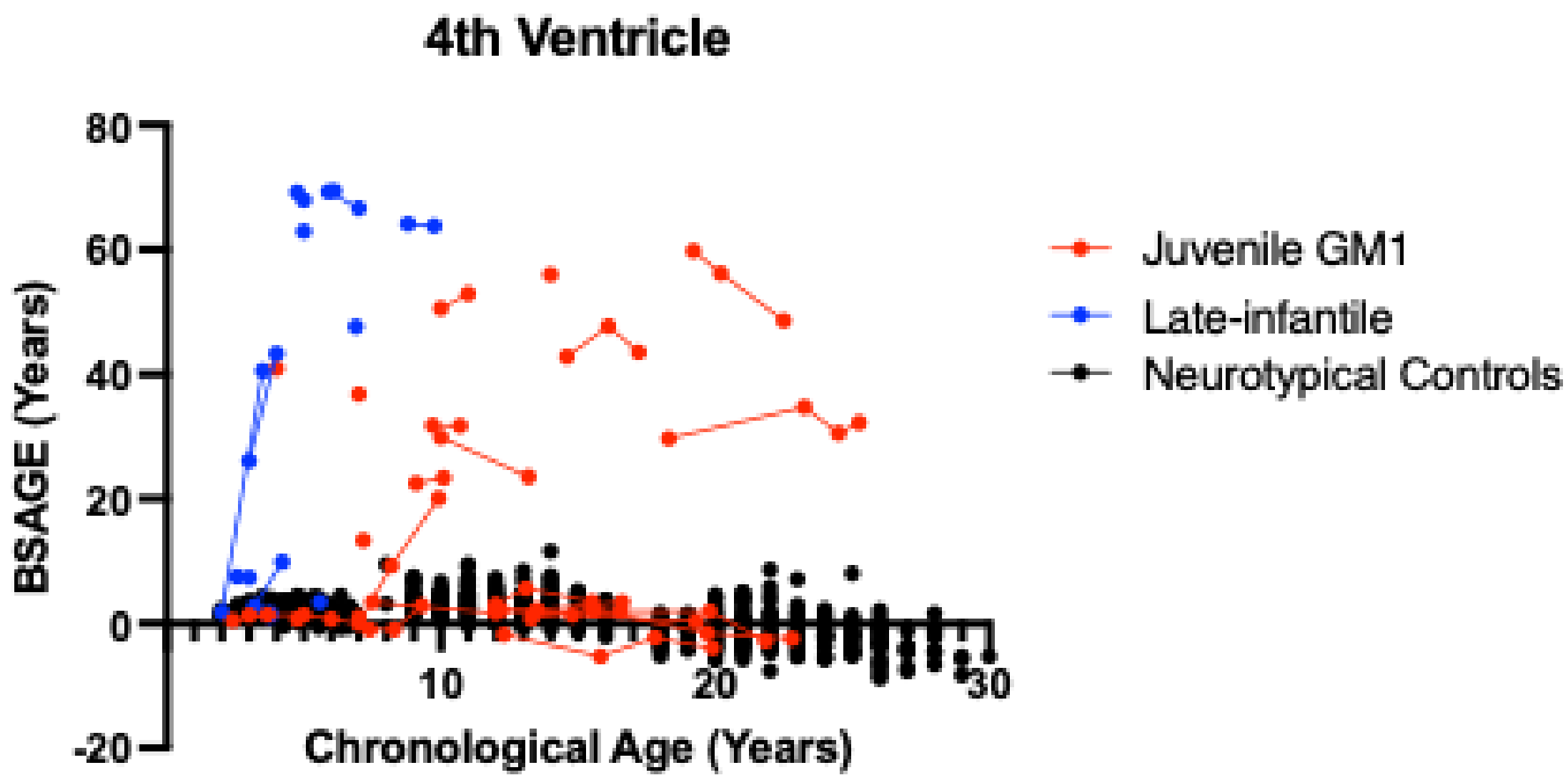

Supplement Figure 38. The longitudinal relationship between 4th ventricle BSAGE and chronological age. Late-infantile GM1 gangliosidosis patients are shown in blue, juvenile GM1 gangliosidosis patients are shown in red, and neurotypical controls participants are shown in black. Connecting lines indicate repeated scans on the same participant.

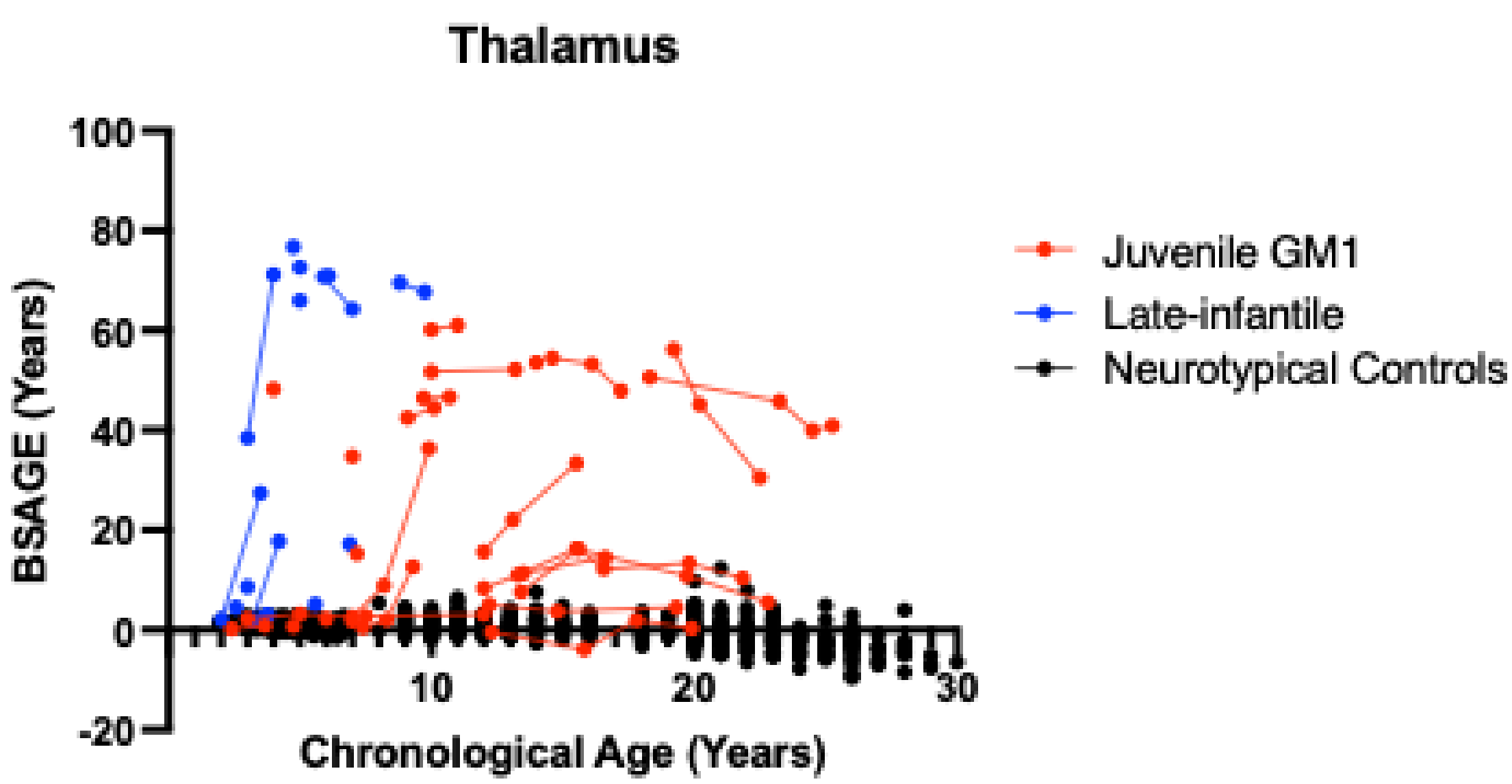

Supplement Figure 39. The longitudinal relationship between thalamus BSAGE and chronological age. Late-infantile GM1 gangliosidosis patients are shown in blue, juvenile GM1 gangliosidosis patients are shown in red, and neurotypical controls participants are shown in black. Connecting lines indicate repeated scans on the same participant.

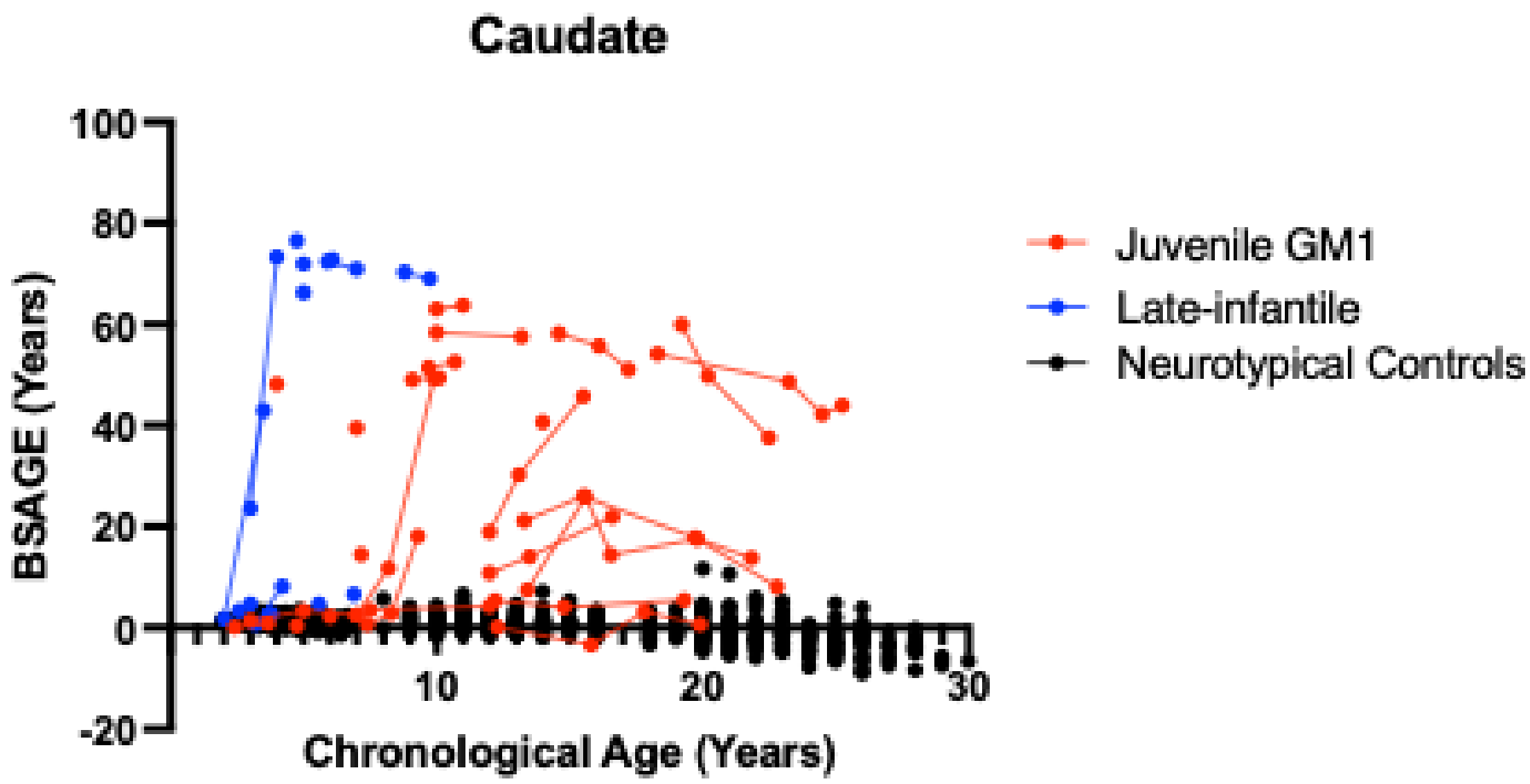

Supplement Figure 40. The longitudinal relationship between caudate nucleus BSAGE and chronological age. Late-infantile GM1 gangliosidosis patients are shown in blue, juvenile GM1 gangliosidosis patients are shown in red, and neurotypical controls participants are shown in black. Connecting lines indicate repeated scans on the same participant.

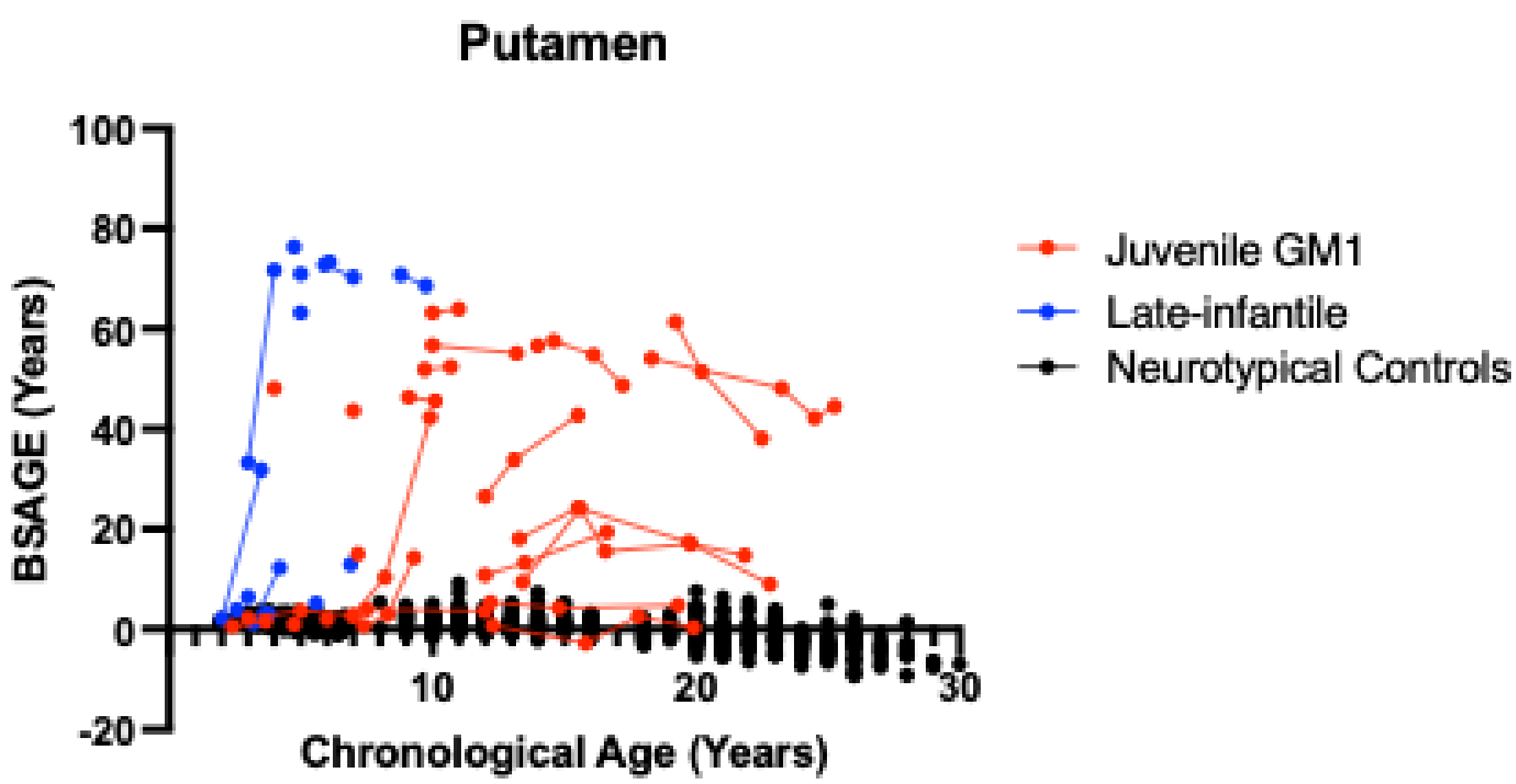

Supplement Figure 41. The longitudinal relationship between putamen BSAGE and chronological age. Late-infantile GM1 gangliosidosis patients are shown in blue, juvenile GM1 gangliosidosis patients are shown in red, and neurotypical controls participants are shown in black. Connecting lines indicate repeated scans on the same participant.

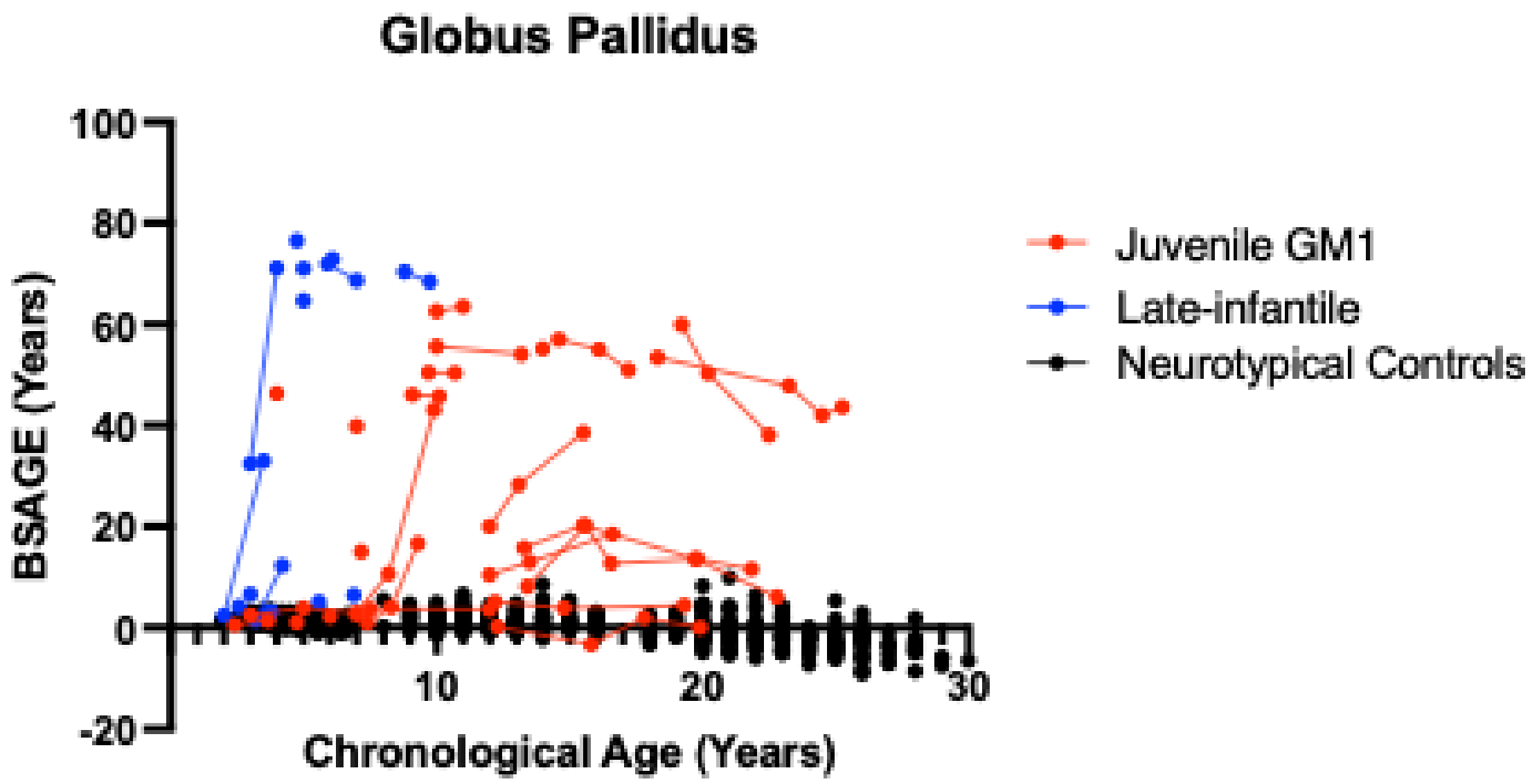

Supplement Figure 42. The longitudinal relationship between globus pallidus BSAGE and chronological age. Late-infantile GM1 gangliosidosis patients are shown in blue, juvenile GM1 gangliosidosis patients are shown in red, and neurotypical controls participants are shown in black. Connecting lines indicate repeated scans on the same participant.

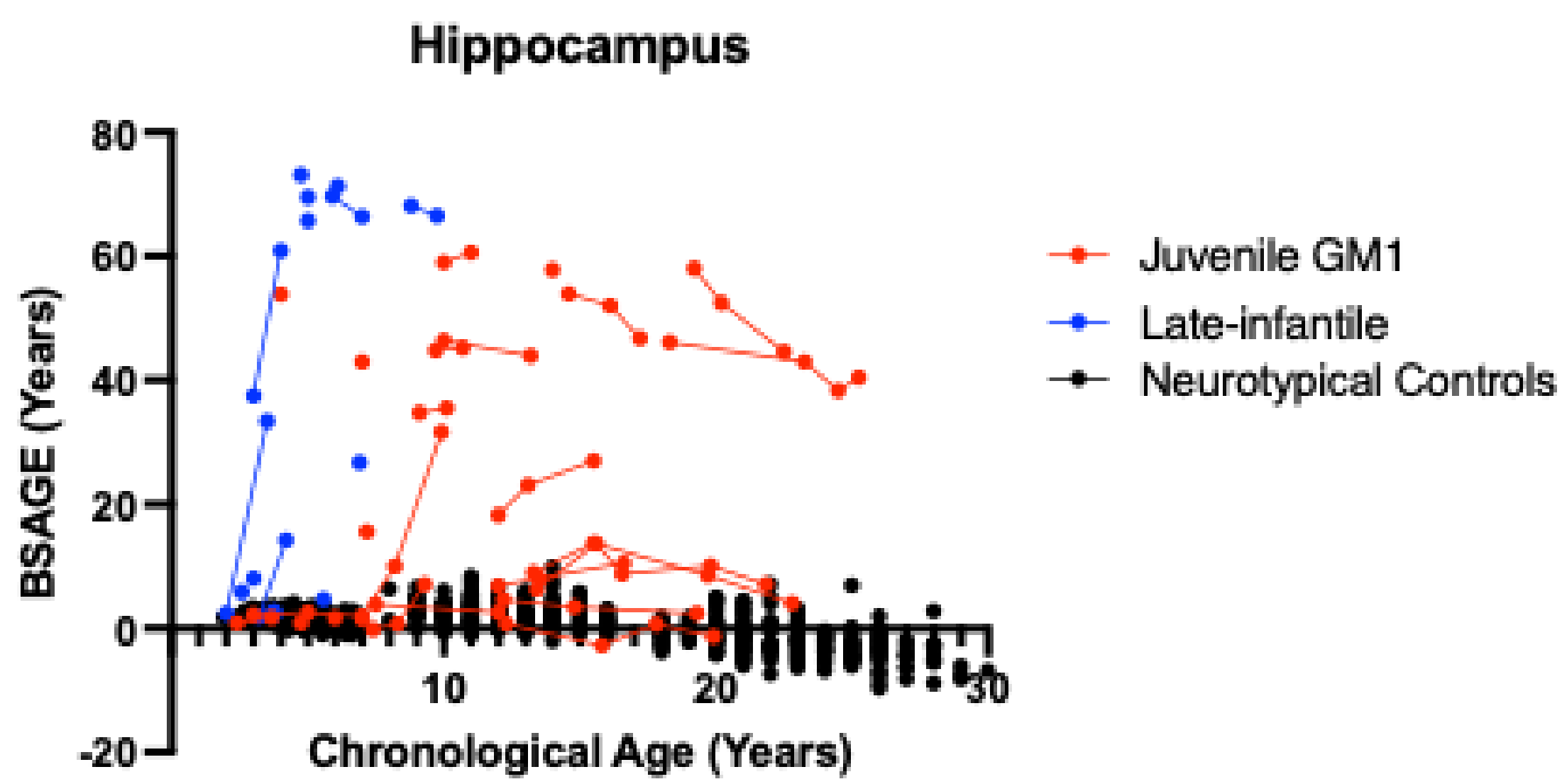

Supplement Figure 43. The longitudinal relationship between hippocampus BSAGE and chronological age. Late-infantile GM1 gangliosidosis patients are shown in blue, juvenile GM1 gangliosidosis patients are shown in red, and neurotypical controls participants are shown in black. Connecting lines indicate repeated scans on the same participant.

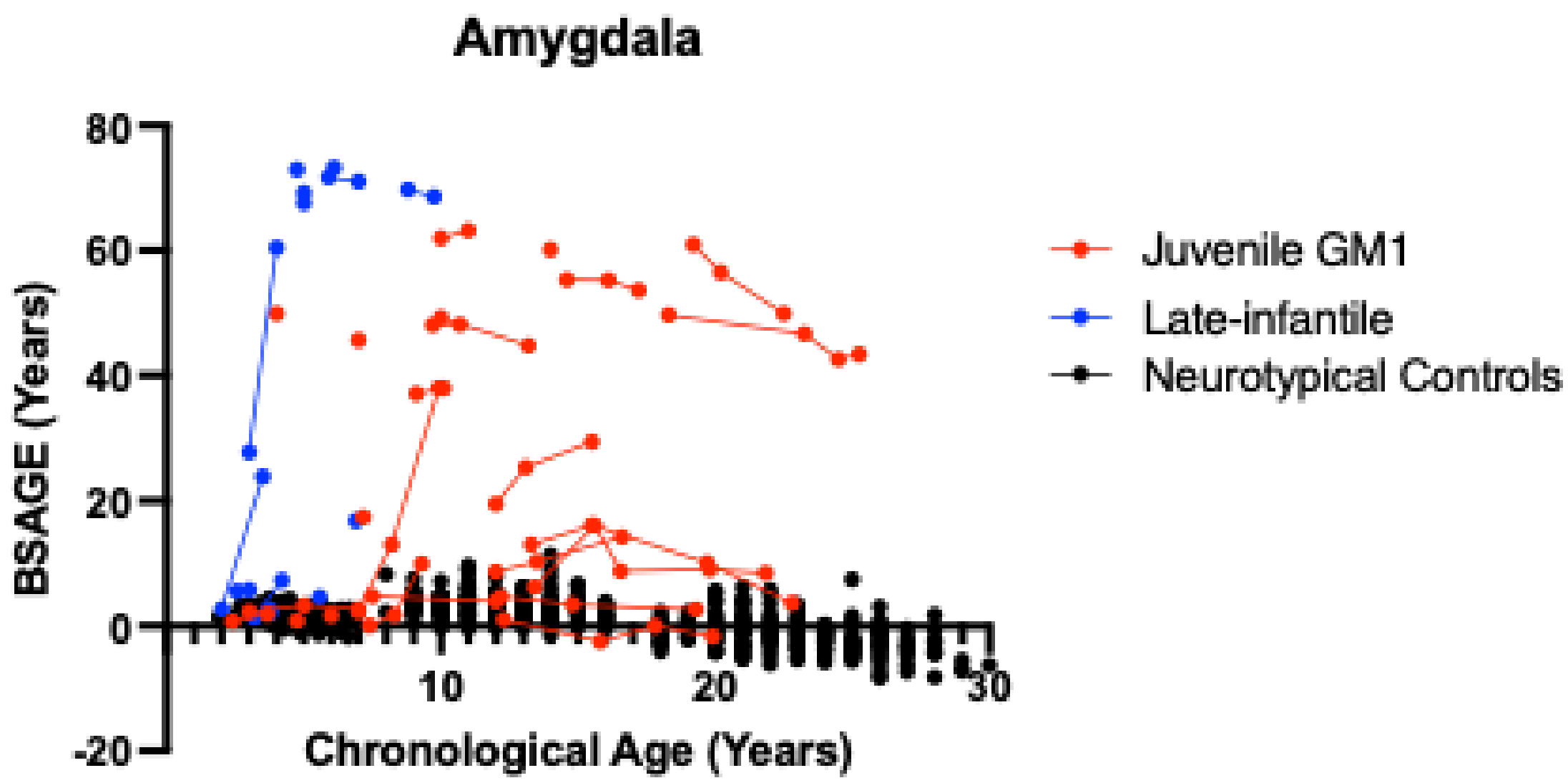

Supplement Figure 44. The longitudinal relationship between amygdala BSAGE and chronological age. Late-infantile GM1 gangliosidosis patients are shown in blue, juvenile GM1 gangliosidosis patients are shown in red, and neurotypical controls participants are shown in black. Connecting lines indicate repeated scans on the same participant.

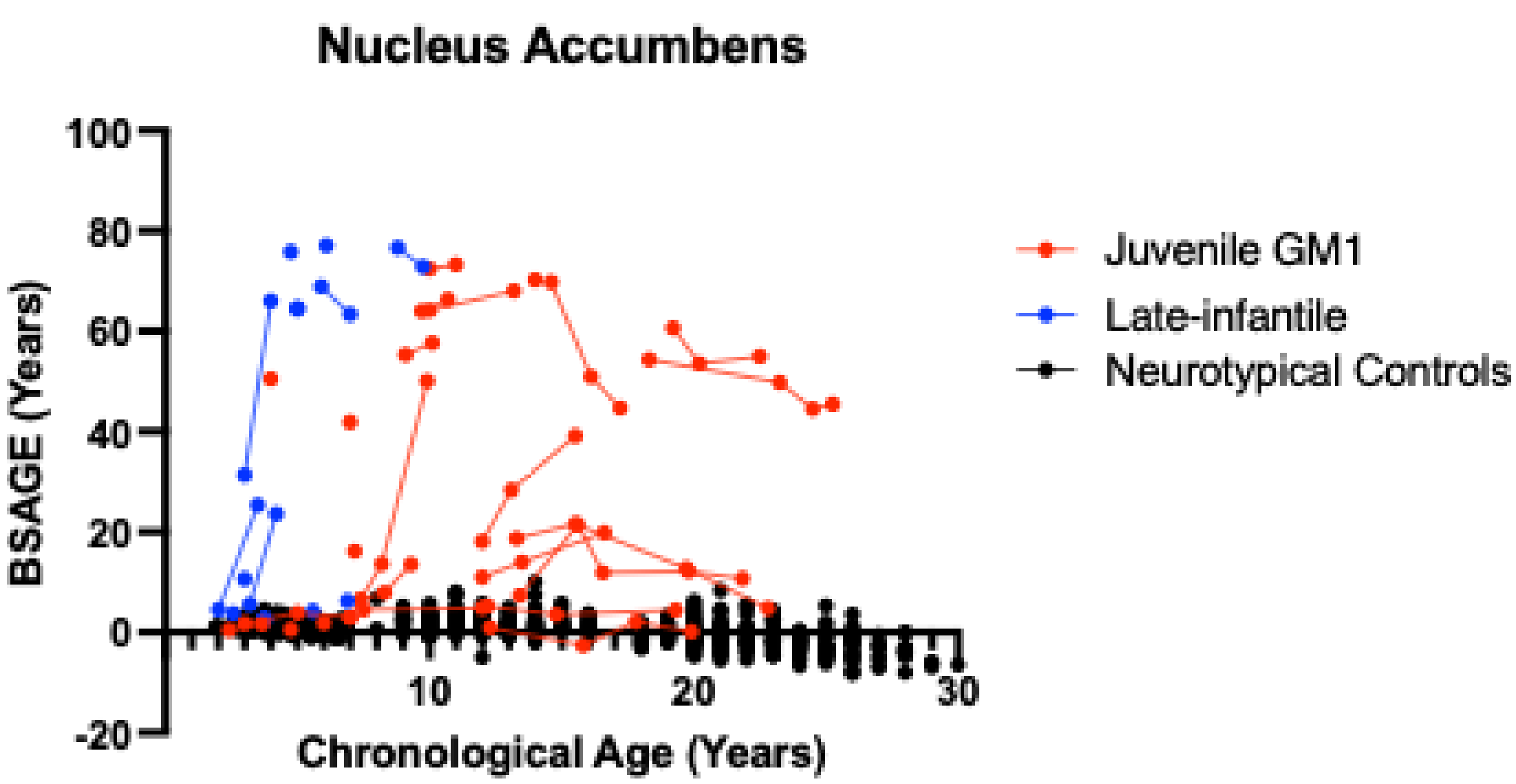

Supplement Figure 45. The longitudinal relationship between nucleus accumbens BSAGE and chronological age. Late-infantile GM1 gangliosidosis patients are shown in blue, juvenile GM1 gangliosidosis patients are shown in red, and neurotypical controls participants are shown in black. Connecting lines indicate repeated scans on the same participant.

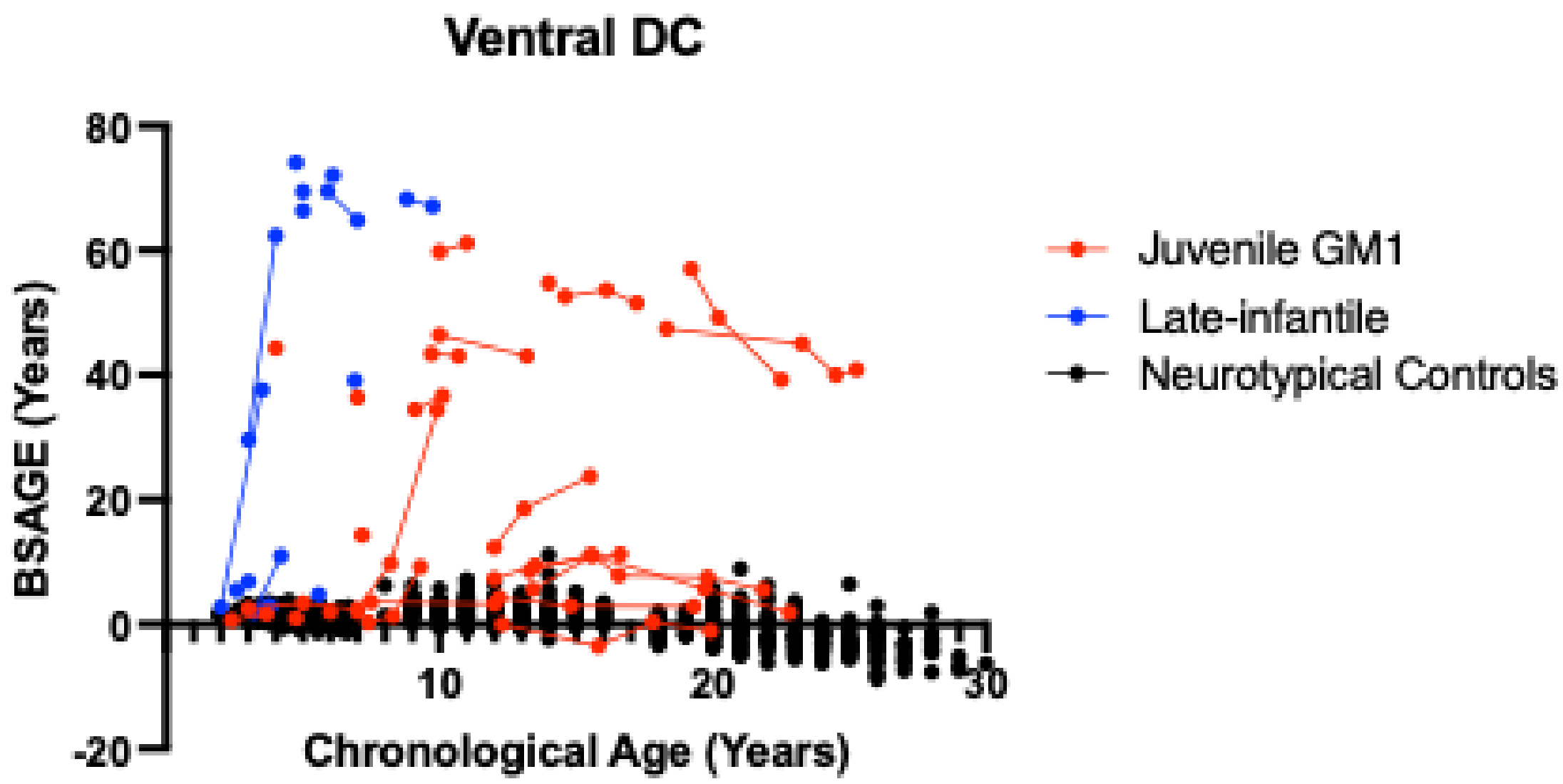

Supplement Figure 46. The longitudinal relationship between ventral diencephalon (DC) BSAGE and chronological age. Late-infantile GM1 gangliosidosis patients are shown in blue, juvenile GM1 gangliosidosis patients are shown in red, and neurotypical controls participants are shown in black. Connecting lines indicate repeated scans on the same participant.

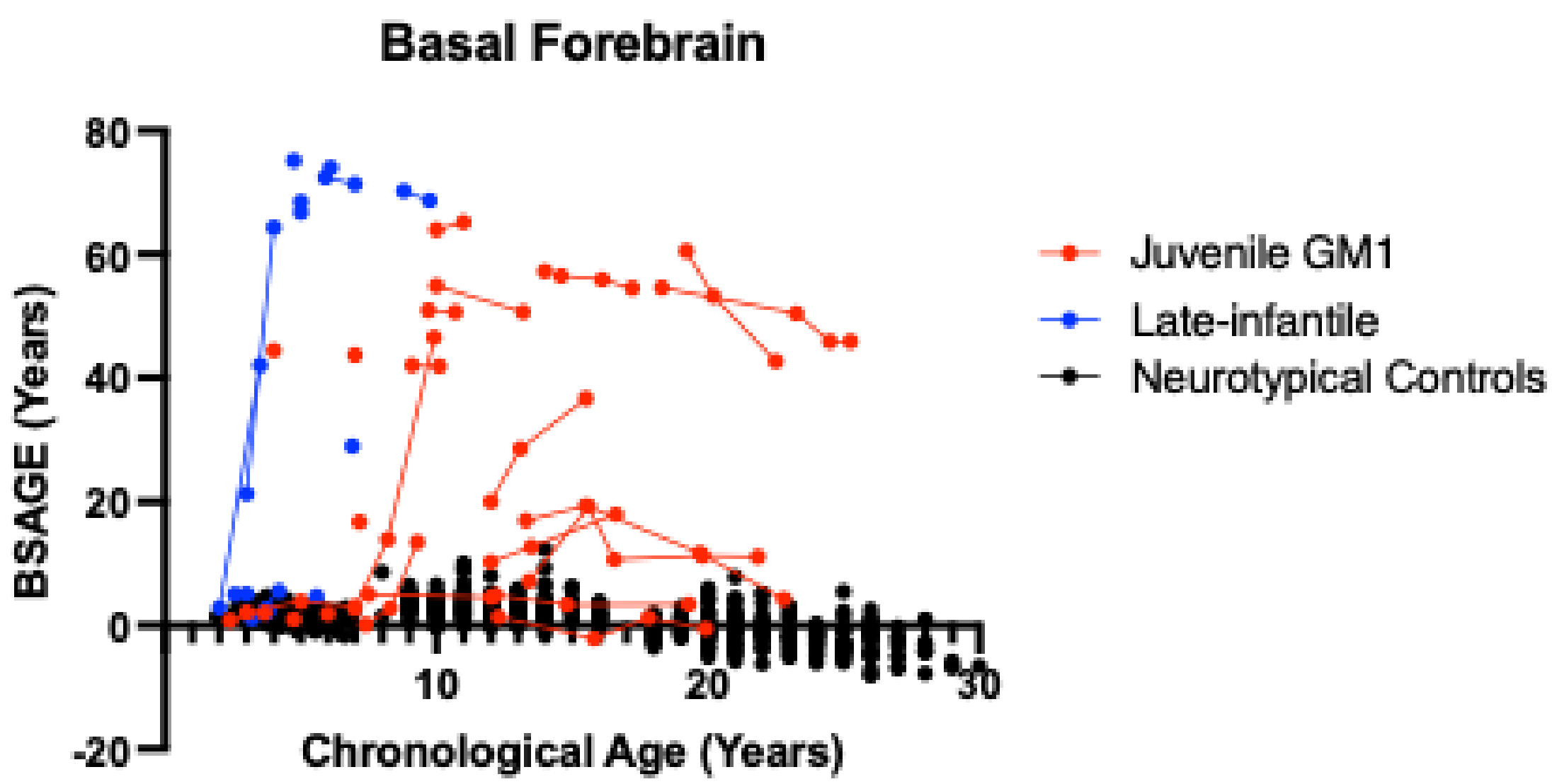

Supplement Figure 47. The longitudinal relationship between basal forebrain BSAGE and chronological age. Late-infantile GM1 gangliosidosis patients are shown in blue, juvenile GM1 gangliosidosis patients are shown in red, and neurotypical controls participants are shown in black. Connecting lines indicate repeated scans on the same participant.

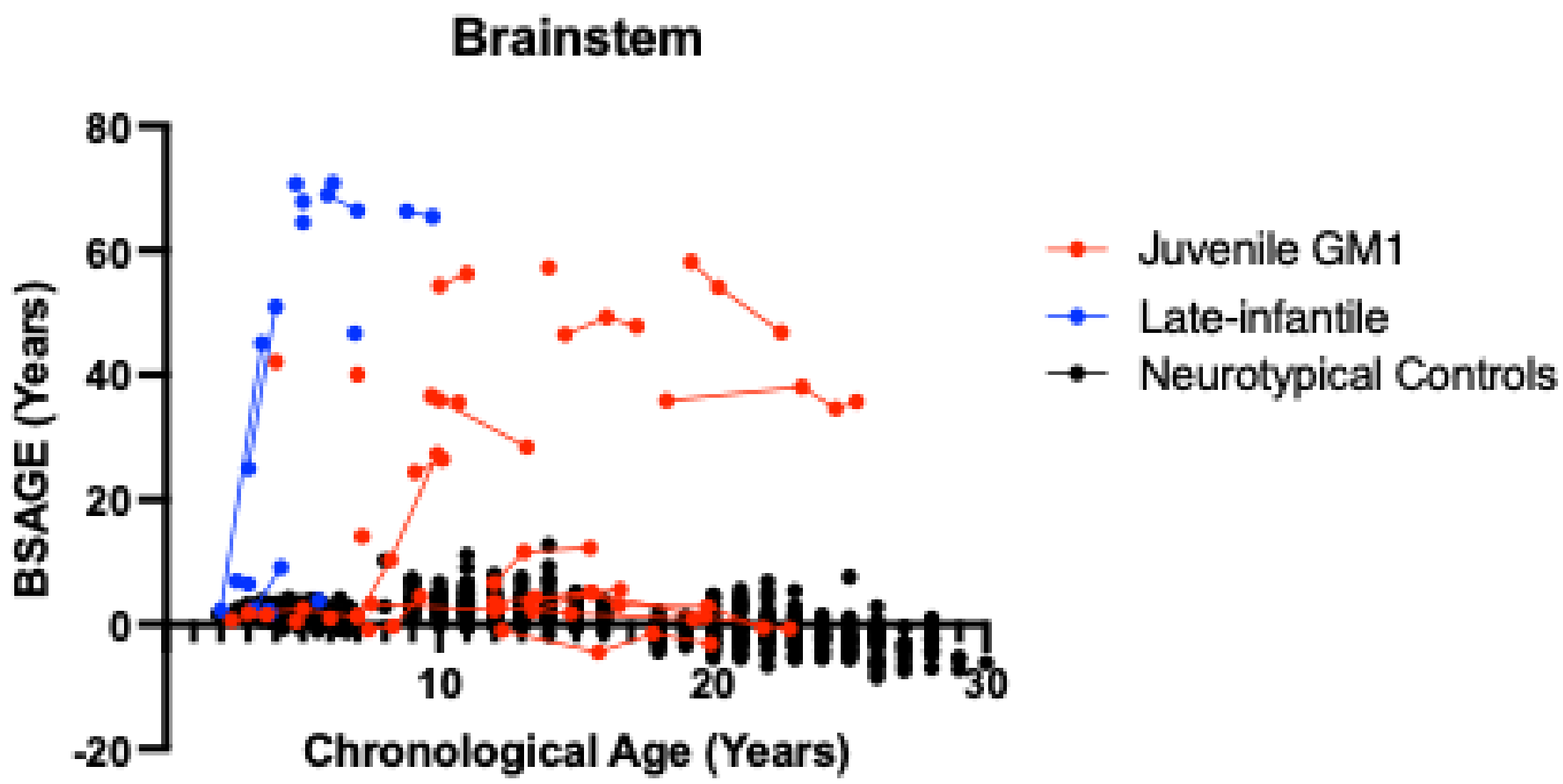

Supplement Figure 48. The longitudinal relationship between brainstem BSAGE and chronological age. Late-infantile GM1 gangliosidosis patients are shown in blue, juvenile GM1 gangliosidosis patients are shown in red, and neurotypical controls participants are shown in black. Connecting lines indicate repeated scans on the same participant.

Supplement I: Cross-Sectional Brain structures age gap estimation (BSAGE)

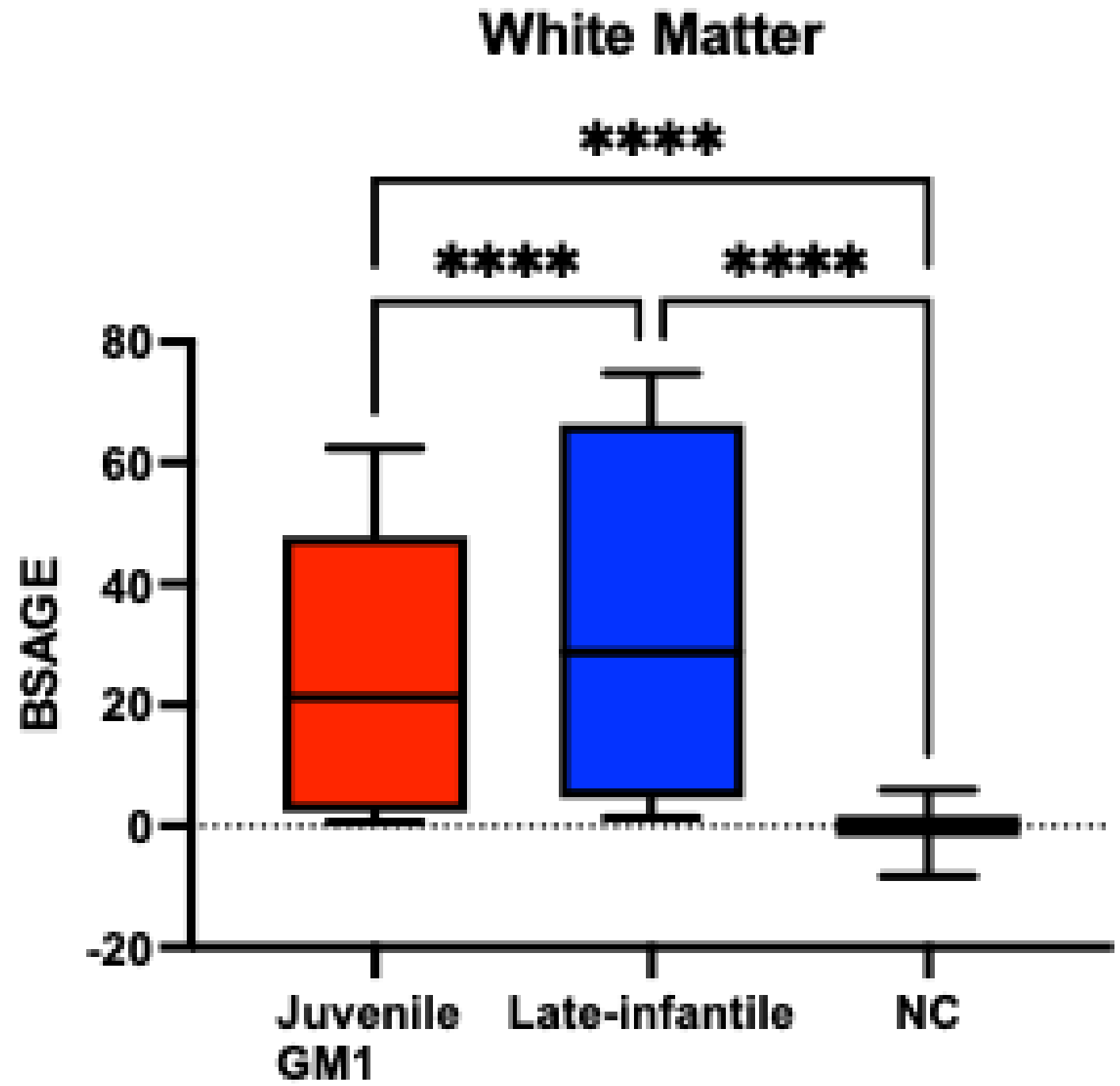

Supplement Figure 49. Comparison of White Matter BSAGE between Juvenile GM1 patients, late-infantile GM1 patients, and neurotypical controls. In all box and whisker plots, the center line represents the median, box limits represent quartiles, and error bars extend to the 1st and 99th percentiles. Late-infantile GM1 patients had an average BSAGE = 36.99, juvenile GM1 patients had an average BSAGE = 25.57, and NC participants had an average BSAGE = -0.21. An ordinary one-way analysis of variance (ANOVA) showed statistically significant ($F(2, 595) = 358.8$, p = 5.949e-103) differences in BSAGE between the cohorts. A post-hoc Tukey test showed the late-infantile cohort had a statistically significantly higher BSAGE compared to both the neurotypical controls (p = 4.79e-10) and juvenile GM1 patients (p = 2.15e-6). The juvenile GM1 cohort also had a significantly higher BSAGE compared to the neurotypical controls (p = 4.79e-10). There were no adjustments made for multiple comparisons of BSAGE based on the number of brain structures analyzed.

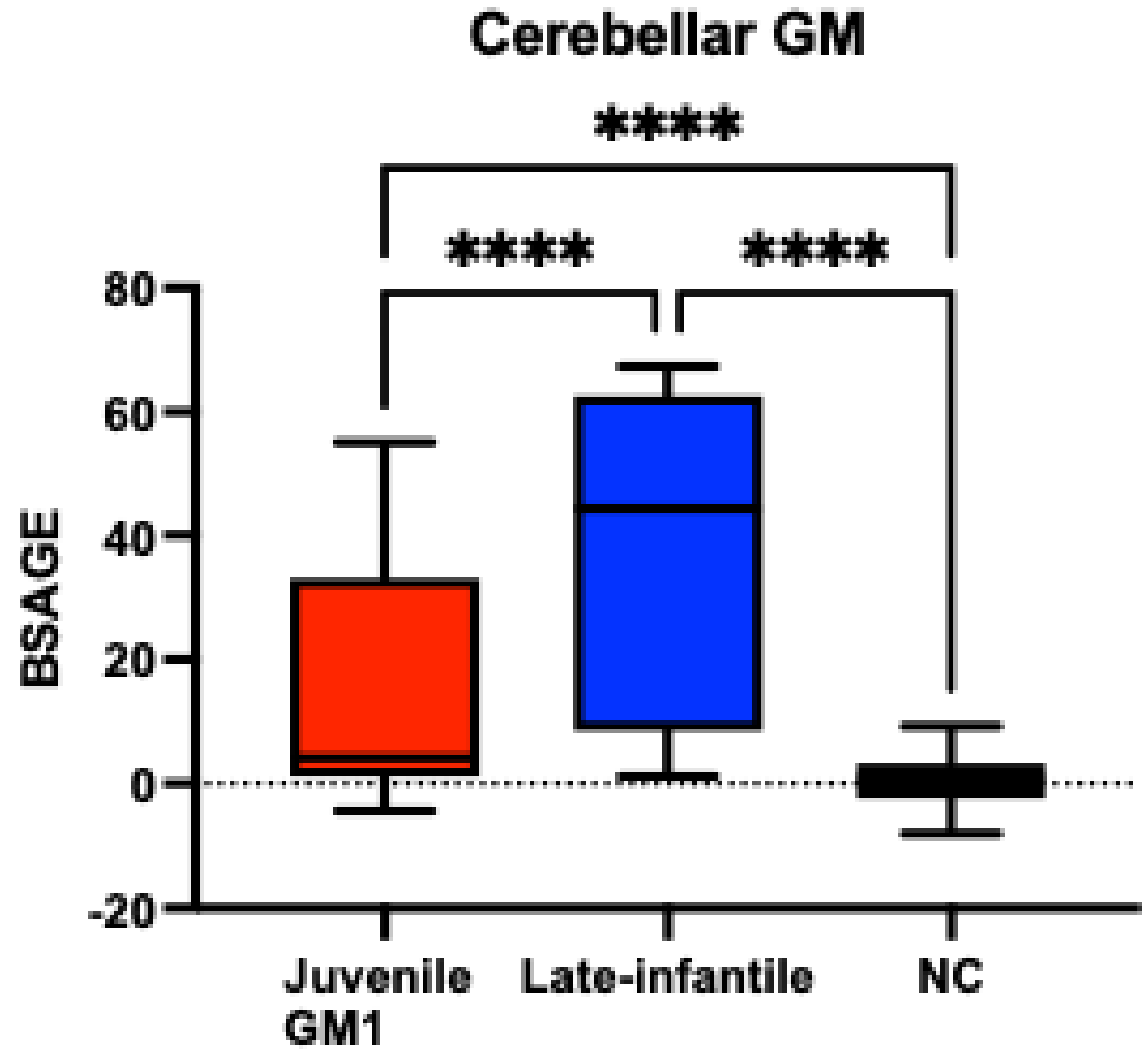

Supplement Figure 50. Comparison of Cerebellar Gray Matter BSAGE between Juvenile GM1 patients, late-infantile GM1 patients, and neurotypical controls. In all box and whisker plots, the center line represents the median, box limits represent quartiles, and error bars extend to the 1st and 99th percentiles. Late-infantile GM1 patients had an average BSAGE = 36.41, juvenile GM1 patients had an average BSAGE = 16.42, and NC participants had an average BSAGE = 0.49. An ordinary one-way analysis of variance (ANOVA) showed statistically significant ($F(2, 595) = 270.8$, $p = 2.374e-84$) differences in BSAGE between the cohorts. A post-hoc Tukey test showed the late-infantile cohort had a statistically significantly higher BSAGE compared to both the neurotypical controls ($p = 4.79e-10$) and juvenile GM1 patients ($p = 4.79e-10$). The juvenile GM1 cohort also had a significantly higher BSAGE compared to the neurotypical controls ($p = 4.79e-10$). There were no adjustments made for multiple comparisons of BSAGE based on the number of brain structures analyzed.

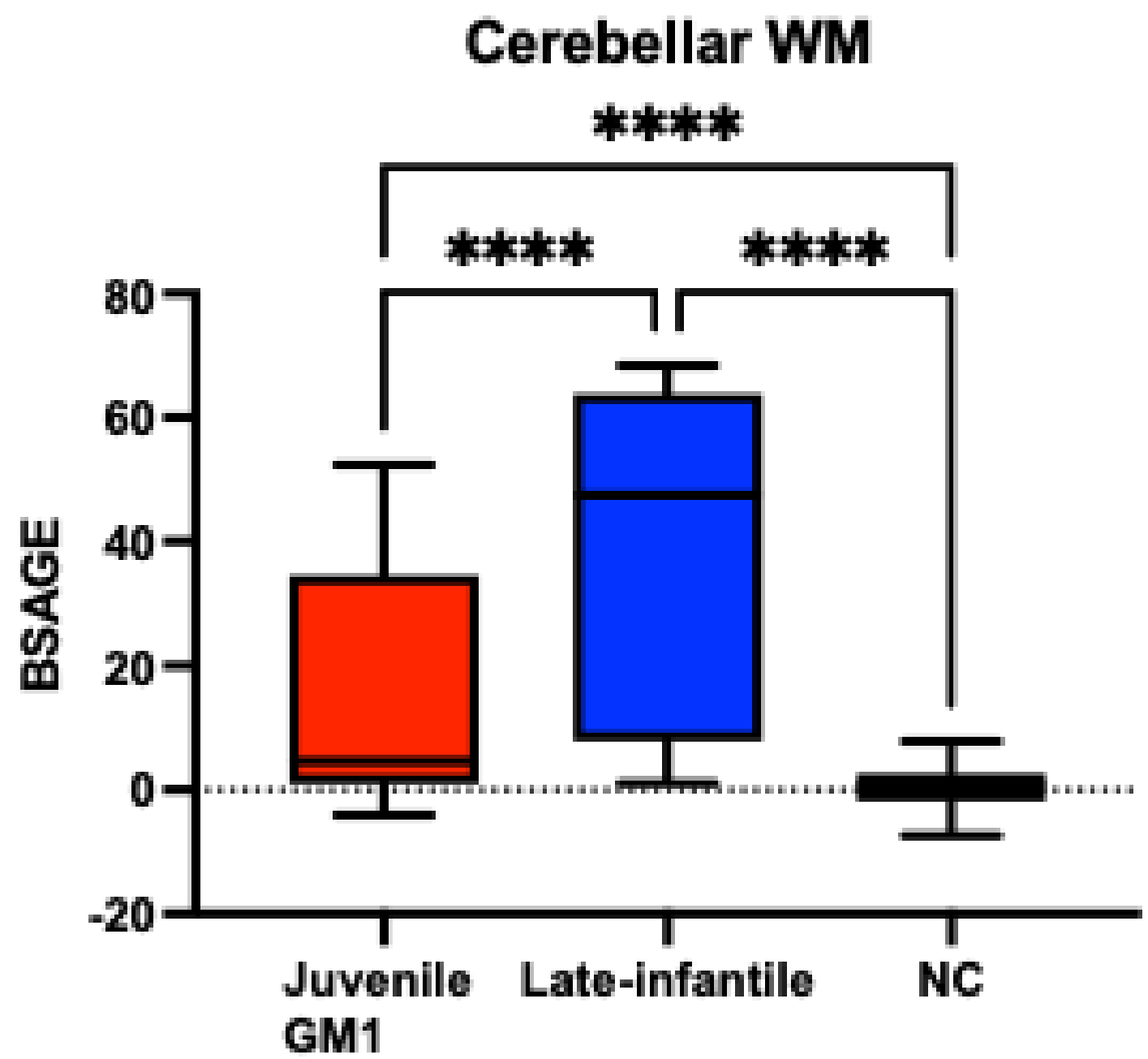

Supplement Figure 51. Comparison of Cerebellar White Matter BSAGE between Juvenile GM1 patients, late-infantile GM1 patients, and neurotypical controls. In all box and whisker plots, the center line represents the median, box limits represent quartiles, and error bars extend to the 1st and 99th percentiles. Late-infantile GM1 patients had an average BSAGE = 38.03, juvenile GM1 patients had an average BSAGE = 16.75, and NC participants had an average BSAGE = 0.36. An analysis of variance (ANOVA) showed statistically significant (F(2, 595) = 285.9, *p* = 9.713e-88) differences in BSAGE between the cohorts. A post-hoc Tukey test showed the late-infantile cohort had a statistically significantly higher BSAGE compared to both the neurotypical controls (*p* = 4.79e-10) and juvenile GM1 patients (*p* = 4.79e-10). The juvenile GM1 cohort also had a significantly higher BSAGE compared to the neurotypical controls (*p* = 4.79e-10). There were no adjustments made for multiple comparisons of BSAGE based on the number of brain structures analyzed.

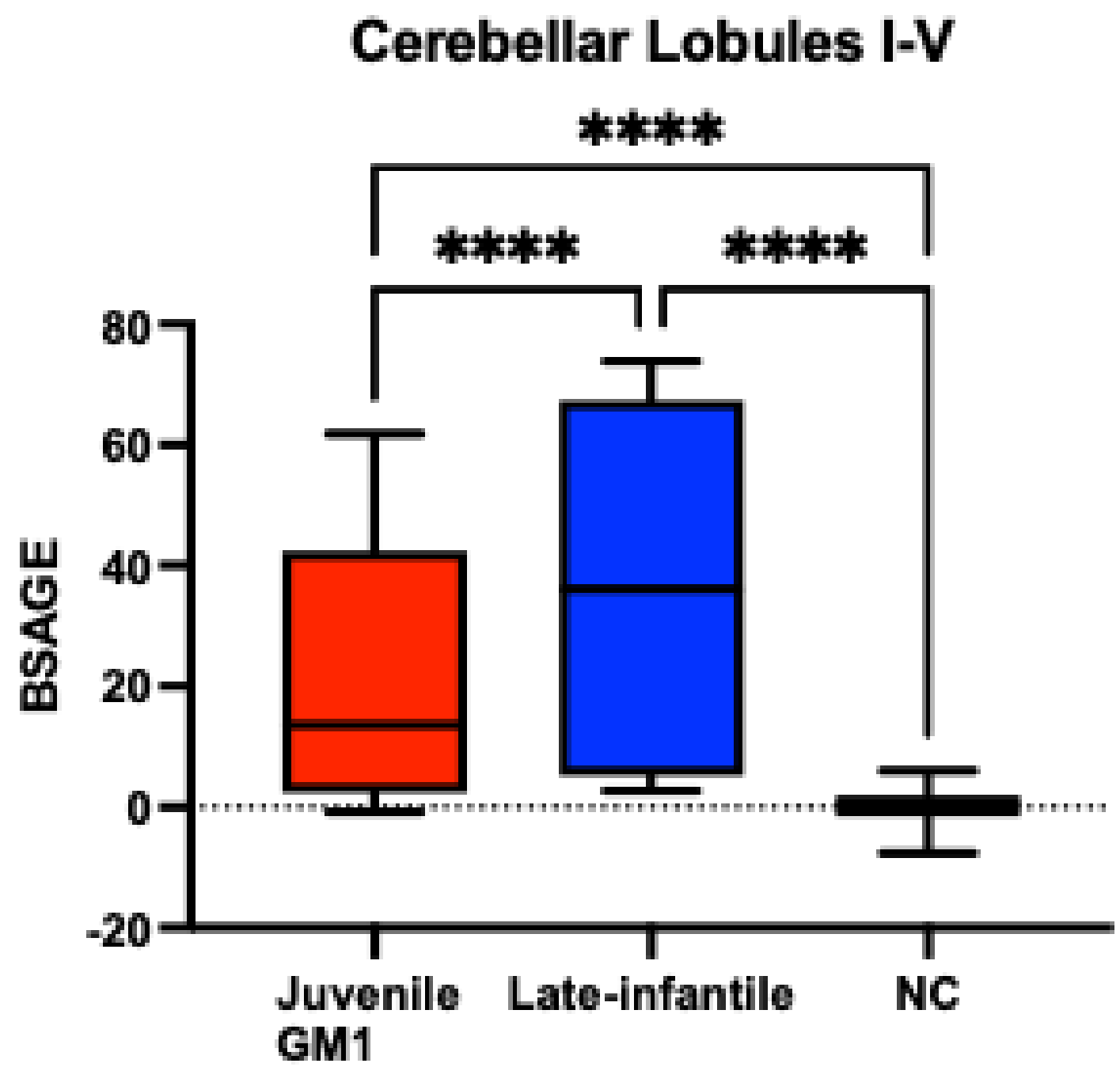

Supplement Figure 52. Comparison of Cerebellar Lobules I-V BSAGE between Juvenile GM1 patients, late-infantile GM1 patients, and neurotypical controls. In all box and whisker plots, the center line represents the median, box limits represent quartiles, and error bars extend to the 1st and 99th percentiles. Late-infantile GM1 patients had an average BSAGE = 38.82, juvenile GM1 patients had an average BSAGE = 22.79, and NC participants had an average BSAGE = 0.16. An analysis of variance (ANOVA) showed statistically significant ($F(2, 595) = 585.6$, p = 2.657e-141) differences in BSAGE between the cohorts. A post-hoc Tukey test showed the late-infantile cohort had a statistically significantly higher BSAGE compared to both the neurotypical controls (p = 4.83e-10) and juvenile GM1 patients (p = 4.98e-10). The juvenile GM1 cohort also had a significantly higher BSAGE compared to the neurotypical controls (p = 4.83e-10). There were no adjustments made for multiple comparisons of BSAGE based on the number of brain structures analyzed.

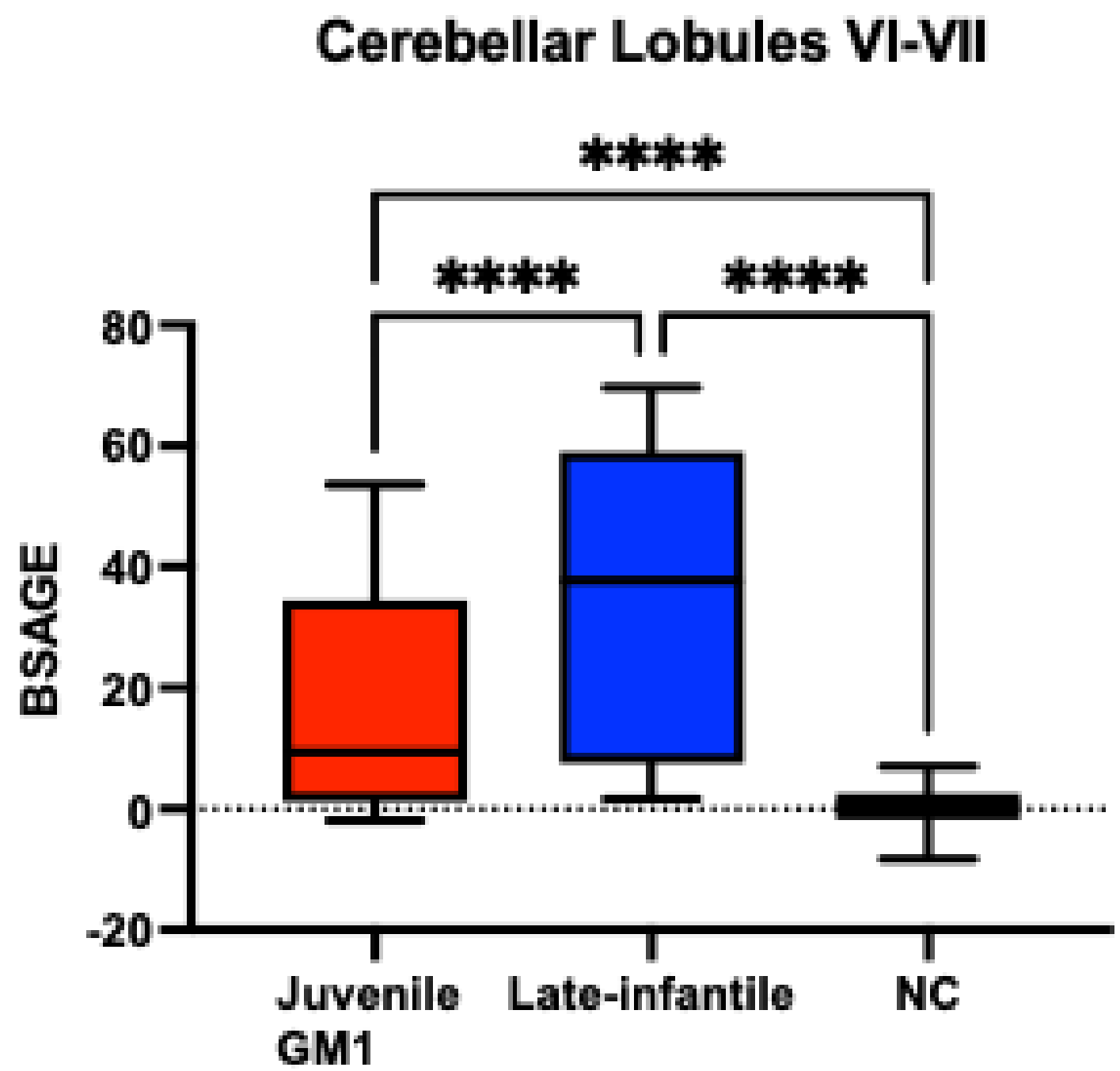

Supplement Figure 53. Comparison of Cerebellar Lobules VI-VII BSAGE between Juvenile GM1 patients, late-infantile GM1 patients, and neurotypical controls. In all box and whisker plots, the center line represents the median, box limits represent quartiles, and error bars extend to the 1st and 99th percentiles. Late-infantile GM1 patients had an average BSAGE = 36.97, juvenile GM1 patients had an average BSAGE = 17.58, and NC participants had an average BSAGE = 0.35. An analysis of variance (ANOVA) showed statistically significant ($F(2, 595) = 358.3$, $p = 4.571e\text{-}108$) differences in BSAGE between the cohorts. A post-hoc Tukey test showed the late-infantile cohort had a statistically significantly higher BSAGE compared to both the neurotypical controls ($p = 4.70e\text{-}10$) and juvenile GM1 patients ($p = 4.70e\text{-}10$). The juvenile GM1 cohort also had a significantly higher BSAGE compared to the neurotypical controls ($p = 4.70e\text{-}10$). There were no adjustments made for multiple comparisons of BSAGE based on the number of brain structures analyzed.

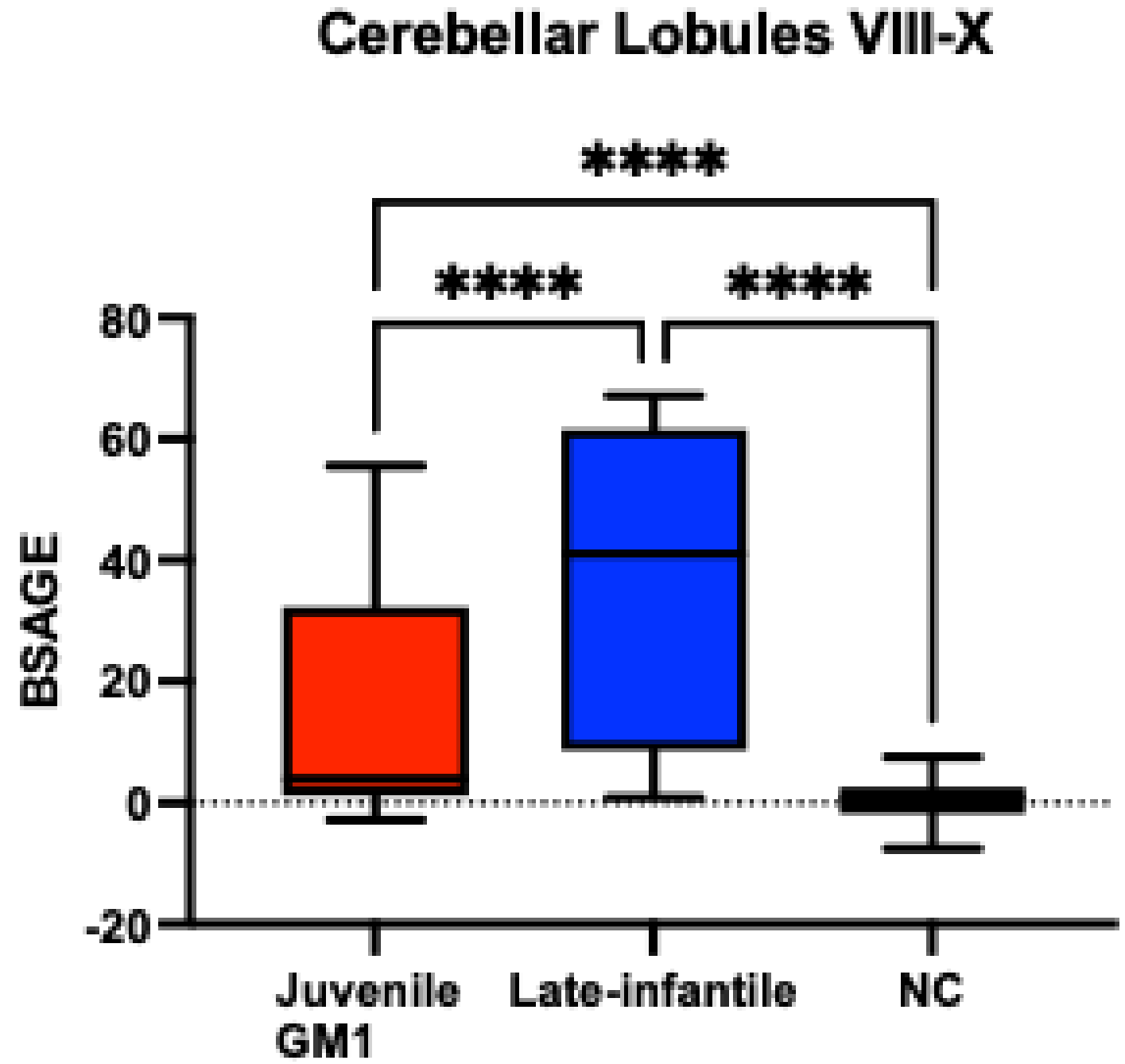

Supplement Figure 54. Comparison of Cerebellar Lobules VIII-X BSAGE between Juvenile GM1 patients, late-infantile GM1 patients, and neurotypical controls. In all box and whisker plots, the center line represents the median, box limits represent quartiles, and error bars extend to the 1st and 99th percentiles. Late-infantile GM1 patients had an average BSAGE = 35.40, juvenile GM1 patients had an average BSAGE = 16.50, and NC participants had an average BSAGE = 0.40. An analysis of variance (ANOVA) showed statistically significant ($F(2, 595) = 284.5$, $p = 1.985e{-}87$) differences in BSAGE between the cohorts. A post-hoc Tukey test showed the late-infantile cohort had a statistically significantly higher BSAGE compared to both the neurotypical controls ($p = 4.75e{-}10$) and juvenile GM1 patients ($p = 4.75e{-}10$). The juvenile GM1 cohort also had a significantly higher BSAGE compared to the neurotypical controls ($p = 4.75e{-}10$). There were no adjustments made for multiple comparisons of BSAGE based on the number of brain structures analyzed.

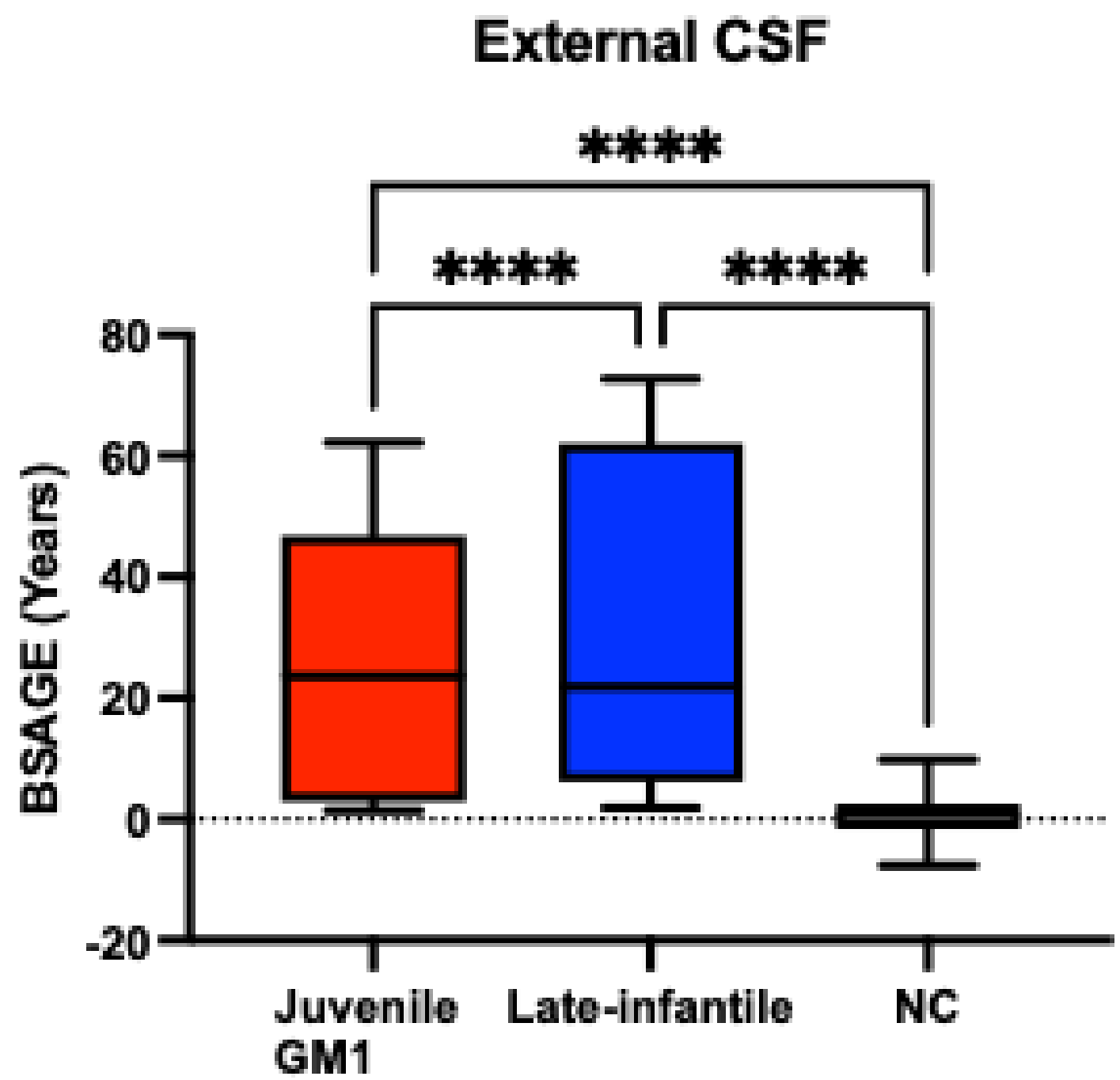

Supplement Figure 55. Comparison of External Cerebrospinal Fluid (CSF) BSAGE between Juvenile GM1 patients, late-infantile GM1 patients, and neurotypical controls (NC). In all box and whisker plots, the center line represents the median, box limits represent quartiles, and error bars extend to the 1st and 99th percentiles. Late-infantile GM1 patients had an average BSAGE = 35.35, juvenile GM1 patients had an average BSAGE = 25.87, and NC participants had an average BSAGE = 0.46. An analysis of variance (ANOVA) showed statistically significant ($F(2, 595) = 428.0$, $p = 6.649e-116$) differences in BSAGE between the cohorts. A post-hoc Tukey test showed the late-infantile cohort had a statistically significantly higher BSAGE compared to both the neurotypical controls ($p = 4.79e-10$) and juvenile GM1 patients ($p = 9.96e-5$). The juvenile GM1 cohort also had a significantly higher BSAGE compared to the neurotypical controls ($p = 4.79e-10$). There were no adjustments made for multiple comparisons of BSAGE based on the number of brain structures analyzed.

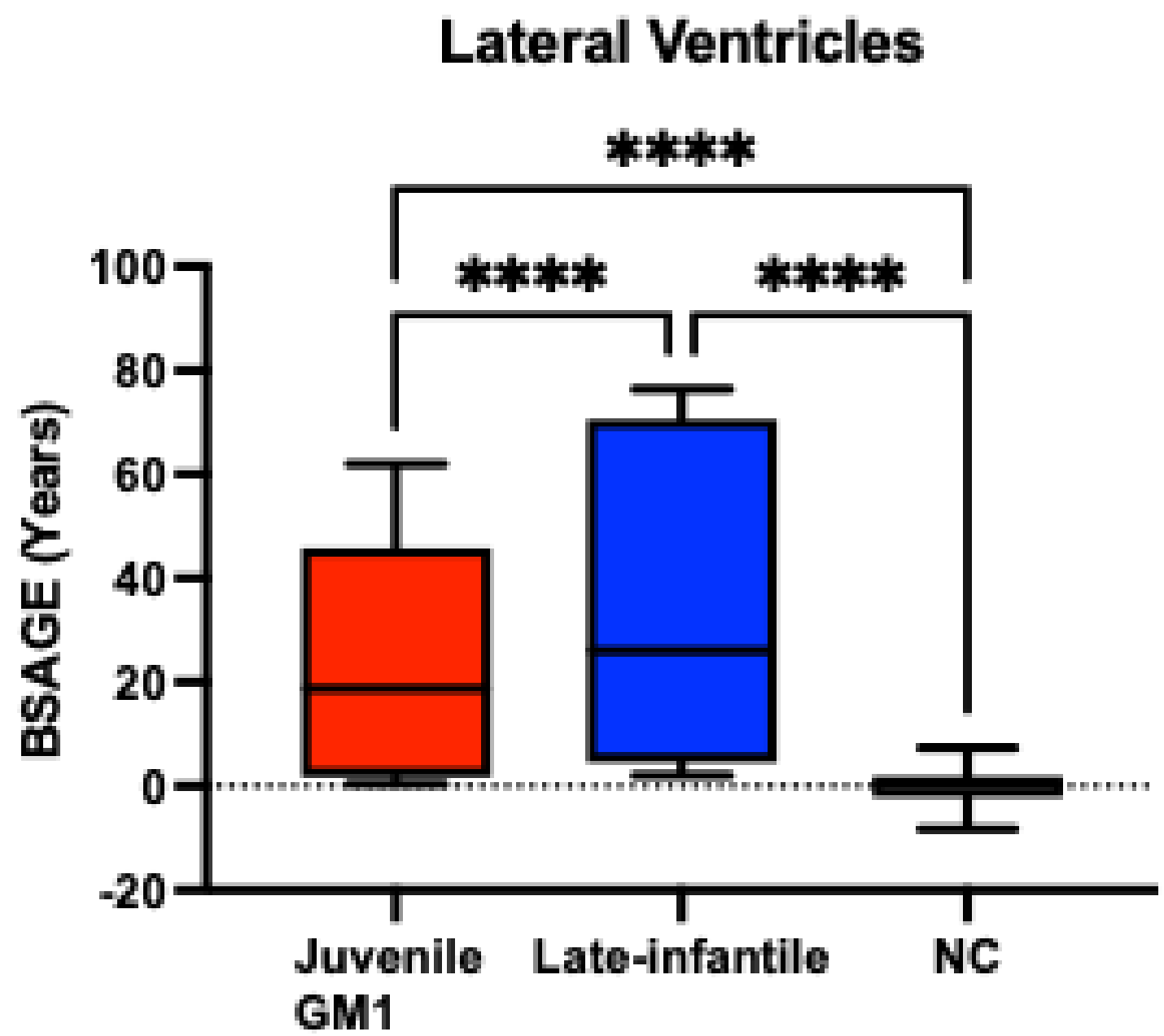

Supplement Figure 56. Comparison of lateral ventricle BSAGE between Juvenile GM1 patients, late-infantile GM1 patients, and neurotypical controls (NC). In all box and whisker plots, the center line represents the median, box limits represent quartiles, and error bars extend to the 1st and 99th percentiles. Late-infantile GM1 patients had an average BSAGE = 37.63, juvenile GM1 patients had an average BSAGE = 25.14, and NC participants had an average BSAGE = -0.06. An analysis of variance (ANOVA) showed statistically significant ($F(2, 595) = 572.6$, p = 2.191e-139) differences in BSAGE between the cohorts. A post-hoc Tukey test showed the late-infantile cohort had a statistically significantly higher BSAGE compared to both the neurotypical controls (p = 4.79e-10) and juvenile GM1 patients (p = 2.95e-7). The juvenile GM1 cohort also had a significantly higher BSAGE compared to the neurotypical controls (p = 4.79e-10). There were no adjustments made for multiple comparisons of BSAGE based on the number of brain structures analyzed.

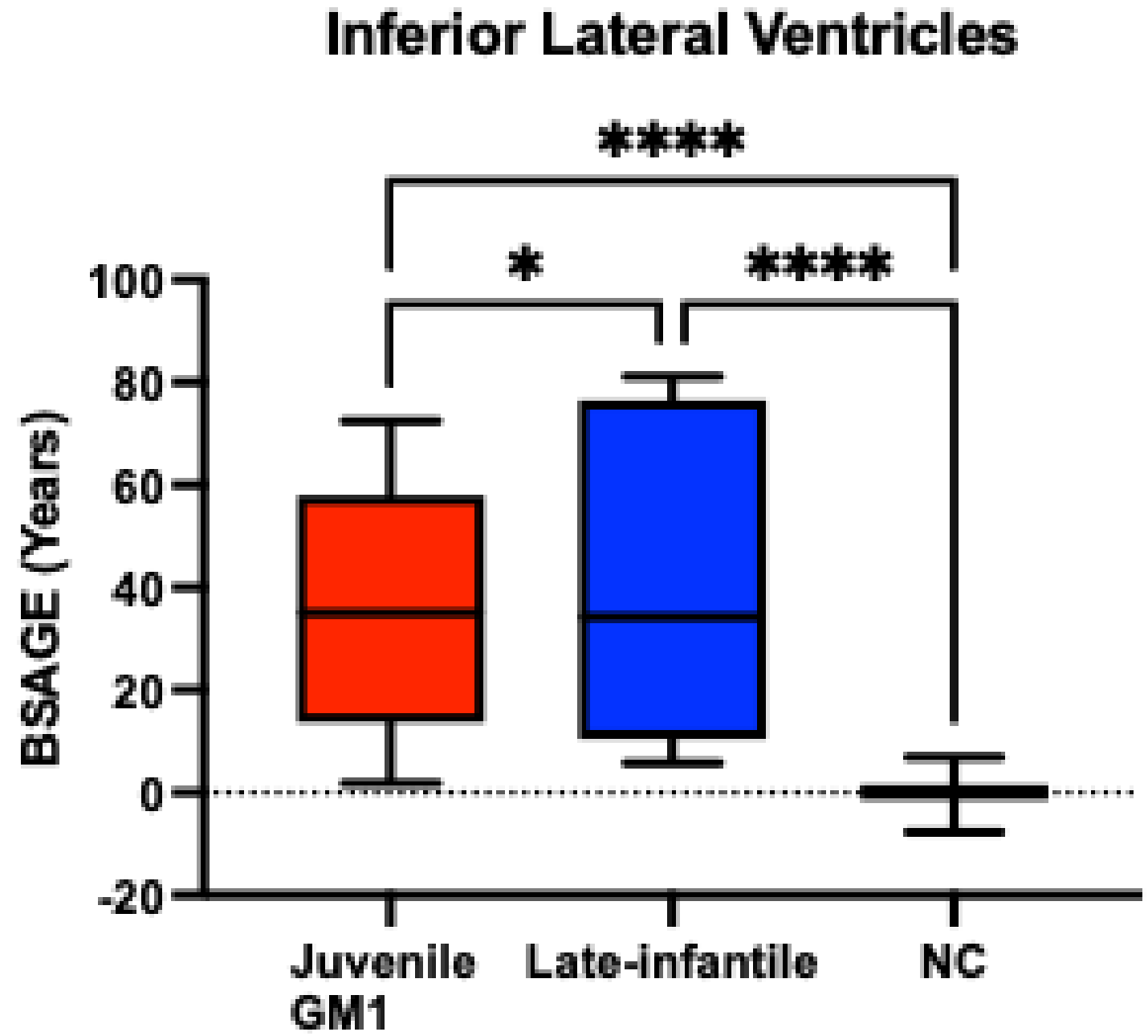

Supplement Figure 57. Comparison of the inferior lateral ventricle BSAGE between Juvenile GM1 patients, late-infantile GM1 patients, and neurotypical controls (NC). In all box and whisker plots, the center line represents the median, box limits represent quartiles, and error bars extend to the 1st and 99th percentiles. Late-infantile GM1 patients had an average BSAGE = 42.49, juvenile GM1 patients had an average BSAGE = 35.77, and NC participants had an average BSAGE = -0.09. An analysis of variance (ANOVA) showed statistically significant ($F(2, 595) = 673.1$, $p = 1.652e-153$) differences in BSAGE between the cohorts. A post-hoc Tukey test showed the late-infantile cohort had a statistically significantly higher BSAGE compared to both the neurotypical controls ($p = 4.79e-10$) and juvenile GM1 patients ($p = 0.0104$). The juvenile GM1 cohort also had a significantly higher BSAGE compared to the neurotypical controls ($p = 4.79e-10$). There were no adjustments made for multiple comparisons of BSAGE based on the number of brain structures analyzed.

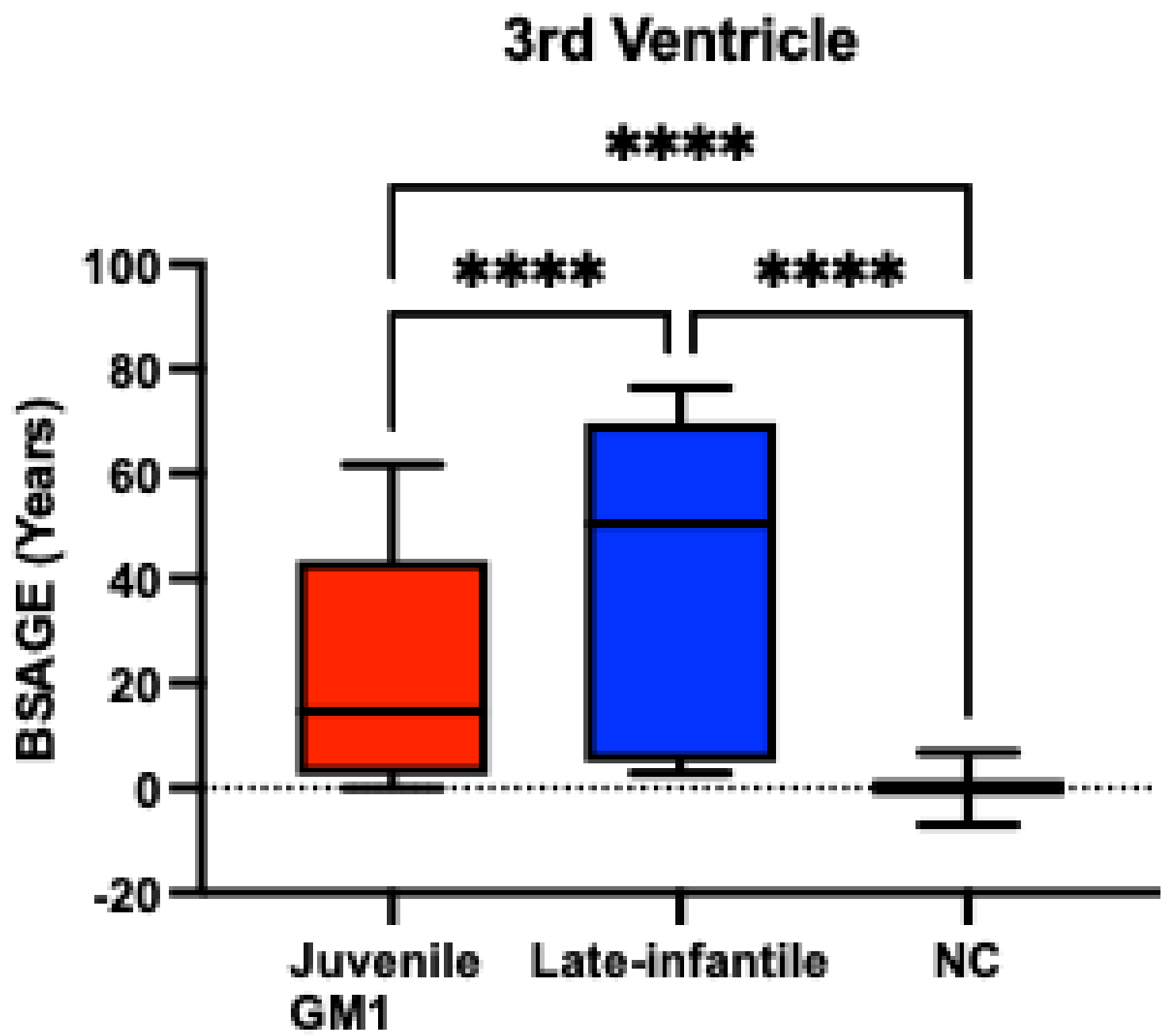

Supplement Figure 58. Comparison of the 3rd ventricle BSAGE between Juvenile GM1 patients, late-infantile GM1 patients, and neurotypical controls (NC). In all box and whisker plots, the center line represents the median, box limits represent quartiles, and error bars extend to the 1st and 99th percentiles. Late-infantile GM1 patients had an average BSAGE = 40.00, juvenile GM1 patients had an average BSAGE = 23.34, and NC participants had an average BSAGE = 0.04. An analysis of variance (ANOVA) showed statistically significant ($F(2,594) = 650.1$, $p = 2.072e\text{-}150$) differences in BSAGE between the cohorts. A post-hoc Tukey test showed the late-infantile cohort had a statistically significantly higher BSAGE compared to both the neurotypical controls ($p = 4.83e\text{-}10$) and juvenile GM1 patients ($p = 4.87e\text{-}10$). The juvenile GM1 cohort also had a significantly higher BSAGE compared to the neurotypical controls ($p = 4.83e\text{-}10$). There were no adjustments made for multiple comparisons of BSAGE based on the number of brain structures analyzed.

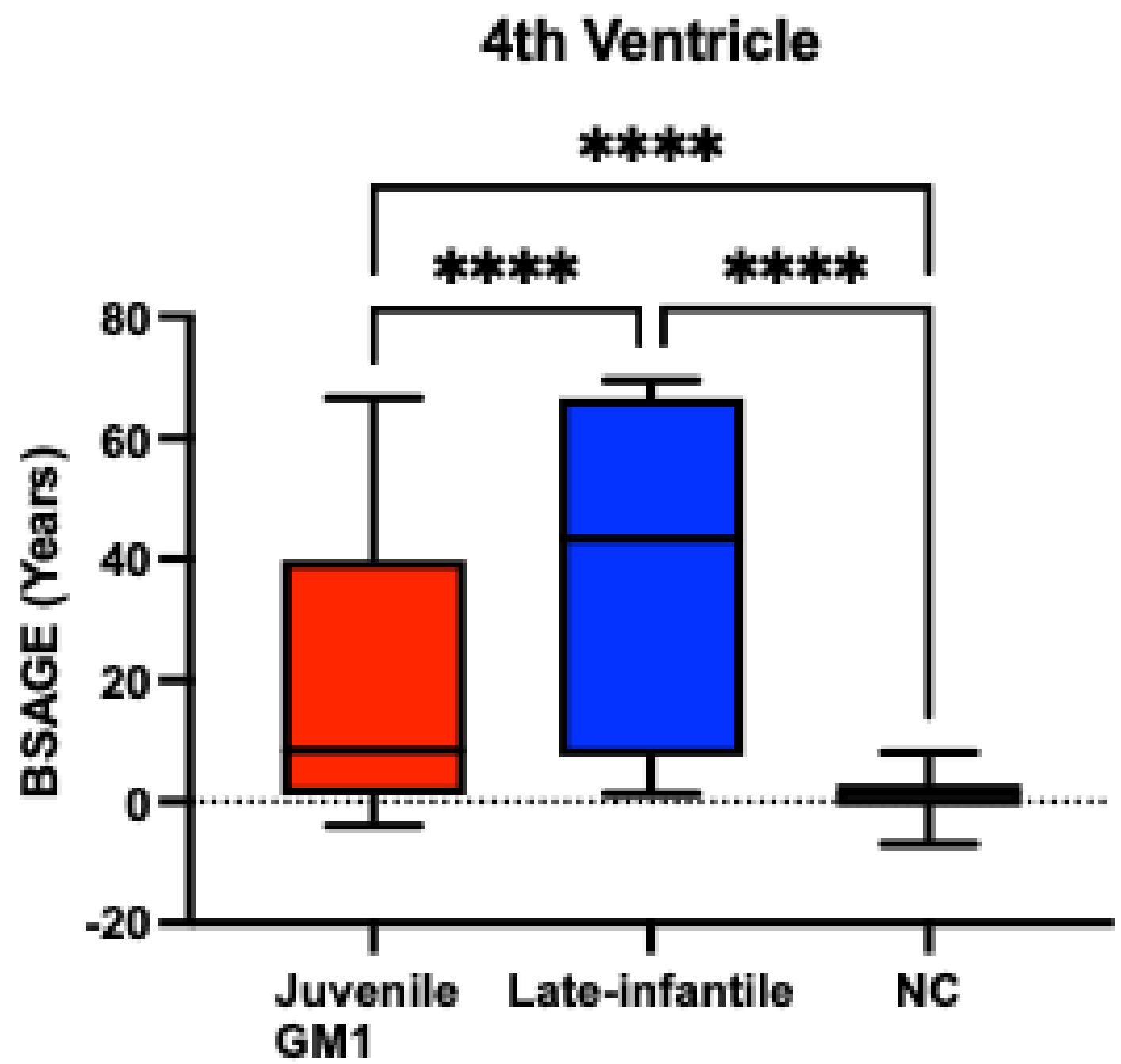

Supplement Figure 59. Comparison of the 4rd ventricle BSAGE between Juvenile GM1 patients, late-infantile GM1 patients, and neurotypical controls (NC). In all box and whisker plots, the center line represents the median, box limits represent quartiles, and error bars extend to the 1st and 99th percentiles. Late-infantile GM1 patients had an average BSAGE = 37.52, juvenile GM1 patients had an average BSAGE = 20.09, and NC participants had an average BSAGE = 0.96. An analysis of variance (ANOVA) showed statistically significant ($F(2, 595) = 438.0$, $p = 1.132e-117$) differences in BSAGE between the cohorts. A post-hoc Tukey test showed the late-infantile cohort had a statistically significantly higher BSAGE compared to both the neurotypical controls ($p < 0.0001$) and juvenile GM1 patients ($p < 0.0001$). The juvenile GM1 cohort also had a significantly higher BSAGE compared to the neurotypical controls ($p < 0.0001$). There were no adjustments made for multiple comparisons of BSAGE based on the number of brain structures analyzed.

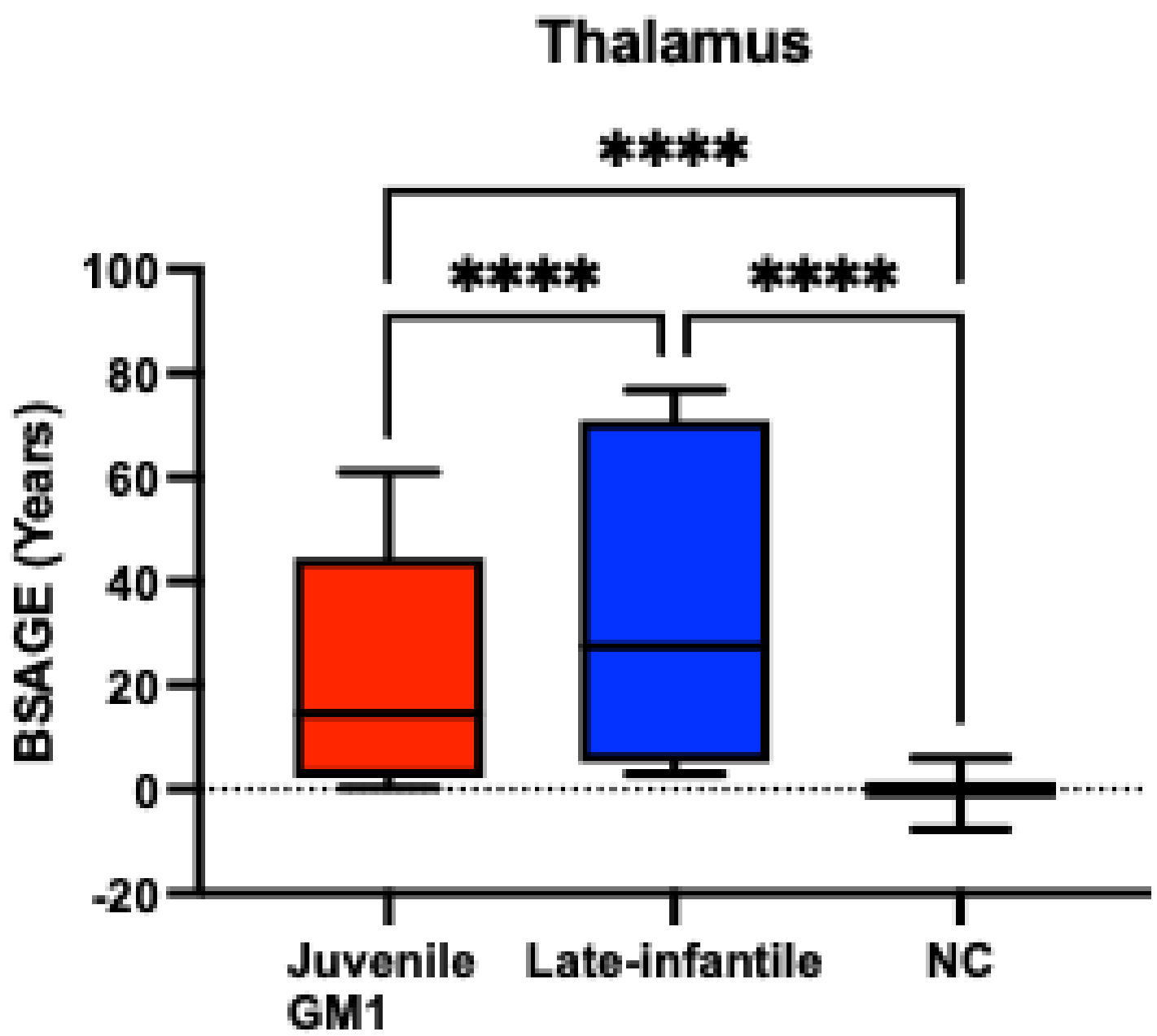

Supplement Figure 60. Comparison of the thalamus BSAGE between Juvenile GM1 patients, late-infantile GM1 patients, and neurotypical controls (NC). In all box and whisker plots, the center line represents the median, box limits represent quartiles, and error bars extend to the 1st and 99th percentiles. Late-infantile GM1 patients had an average BSAGE = 38.41, juvenile GM1 patients had an average BSAGE = 22.49, and NC participants had an average BSAGE = -0.24. An analysis of variance (ANOVA) showed statistically significant ($F(2, 595) = 558.1$, $p = 3.25e\text{-}137$) differences in BSAGE between the cohorts. A post-hoc Tukey test showed the late-infantile cohort had a statistically significantly higher BSAGE compared to both the neurotypical controls ($p = 4.75e\text{-}10$) and juvenile GM1 patients ($p = 4.88e\text{-}10$). The juvenile GM1 cohort also had a significantly higher BSAGE compared to the neurotypical controls ($p = 4.75e\text{-}10$). There were no adjustments made for multiple comparisons of BSAGE based on the number of brain structures analyzed.

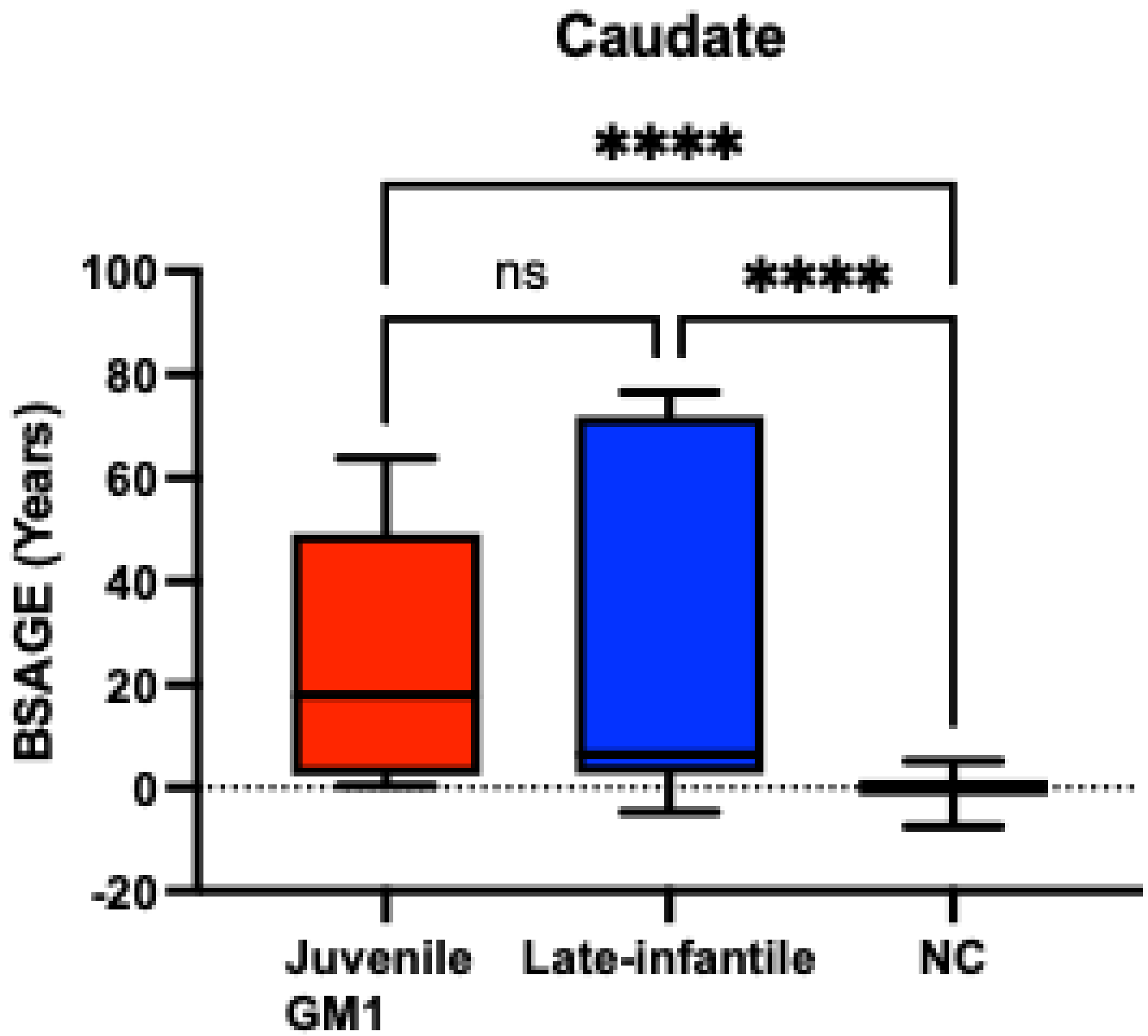

Supplement Figure 61. Comparison of the Caudate Nucleus BSAGE between Juvenile GM1 patients, late-infantile GM1 patients, and neurotypical controls (NC). In all box and whisker plots, the center line represents the median, box limits represent quartiles, and error bars extend to the 1st and 99th percentiles. Late-infantile GM1 patients had an average BSAGE = 30.64, juvenile GM1 patients had an average BSAGE = 25.34, and NC participants had an average BSAGE = -0.11. An analysis of variance (ANOVA) showed statistically significant ($F(2, 595) = 289.0$, $p = 2.007e-88$) differences in BSAGE between the cohorts. A post-hoc Tukey test showed the late-infantile cohort had a statistically significantly higher BSAGE compared to the neurotypical controls ($p = 4.79e-10$). There was no statistical difference between the late-infantile and juvenile cohorts ($p = 0.0806$). The juvenile GM1 cohort also had a significantly higher BSAGE compared to the neurotypical controls ($p = 4.79e-10$). There were no adjustments made for multiple comparisons of BSAGE based on the number of brain structures analyzed.

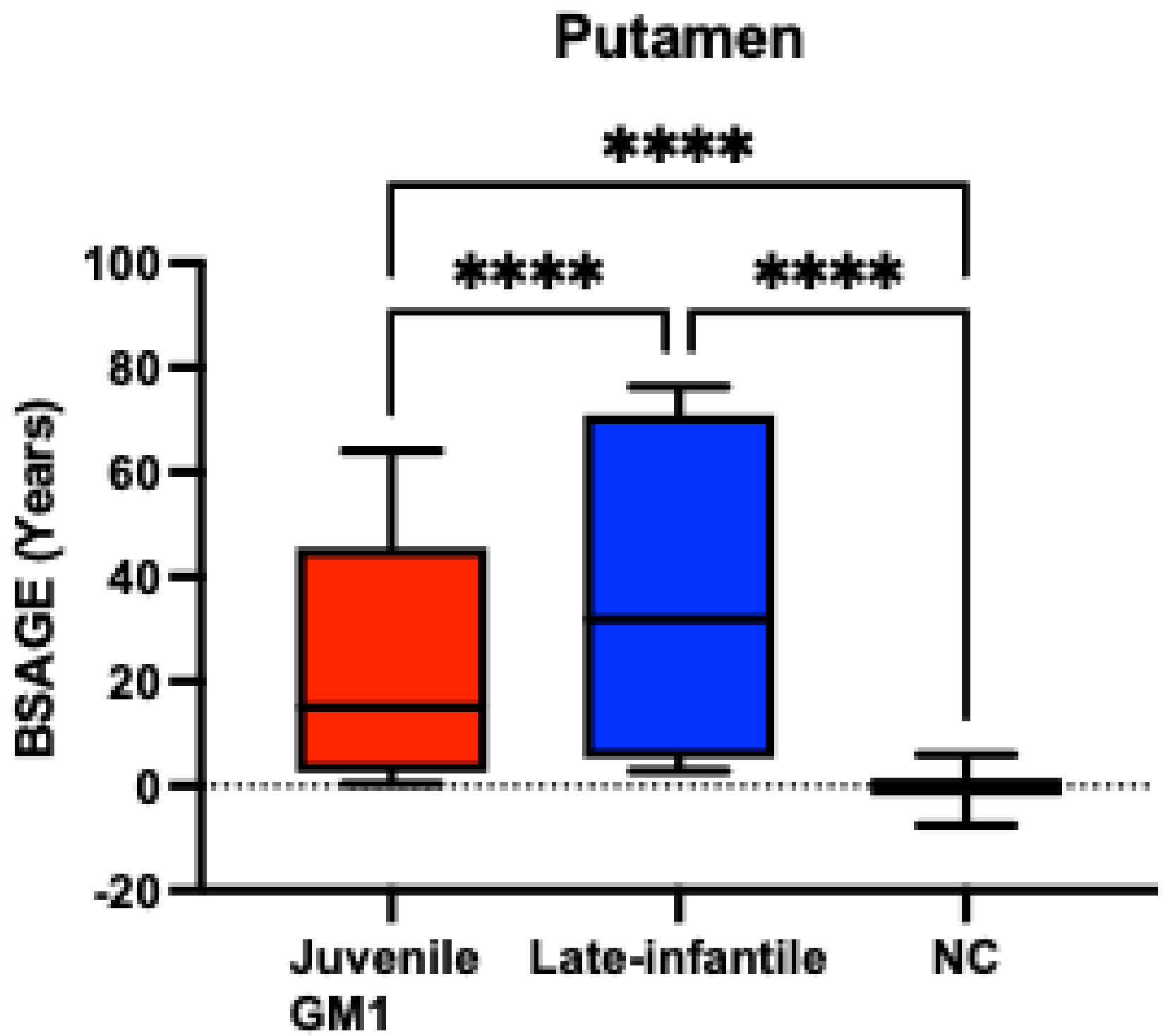

Supplement Figure 62. Comparison of the Putamen BSAGE between Juvenile GM1 patients, late-infantile GM1 patients, and neurotypical controls (NC). In all box and whisker plots, the center line represents the median, box limits represent quartiles, and error bars extend to the 1st and 99th percentiles. Late-infantile GM1 patients had an average BSAGE = 38.20, juvenile GM1 patients had an average BSAGE = 25.05, and NC participants had an average BSAGE = -0.19. An analysis of variance (ANOVA) showed statistically significant ($F(2, 595) = 660.8$, $p = 7.34e\text{-}152$) differences in BSAGE between the cohorts. A post-hoc Tukey test showed the late-infantile cohort had a statistically significantly higher BSAGE compared to both the neurotypical controls ($p = 4.75e\text{-}10$) and juvenile GM1 patients ($p = 9.83e\text{-}8$). The juvenile GM1 cohort also had a significantly higher BSAGE compared to the neurotypical controls ($p = 4.75e\text{-}10$). There were no adjustments made for multiple comparisons of BSAGE based on the number of brain structures analyzed.

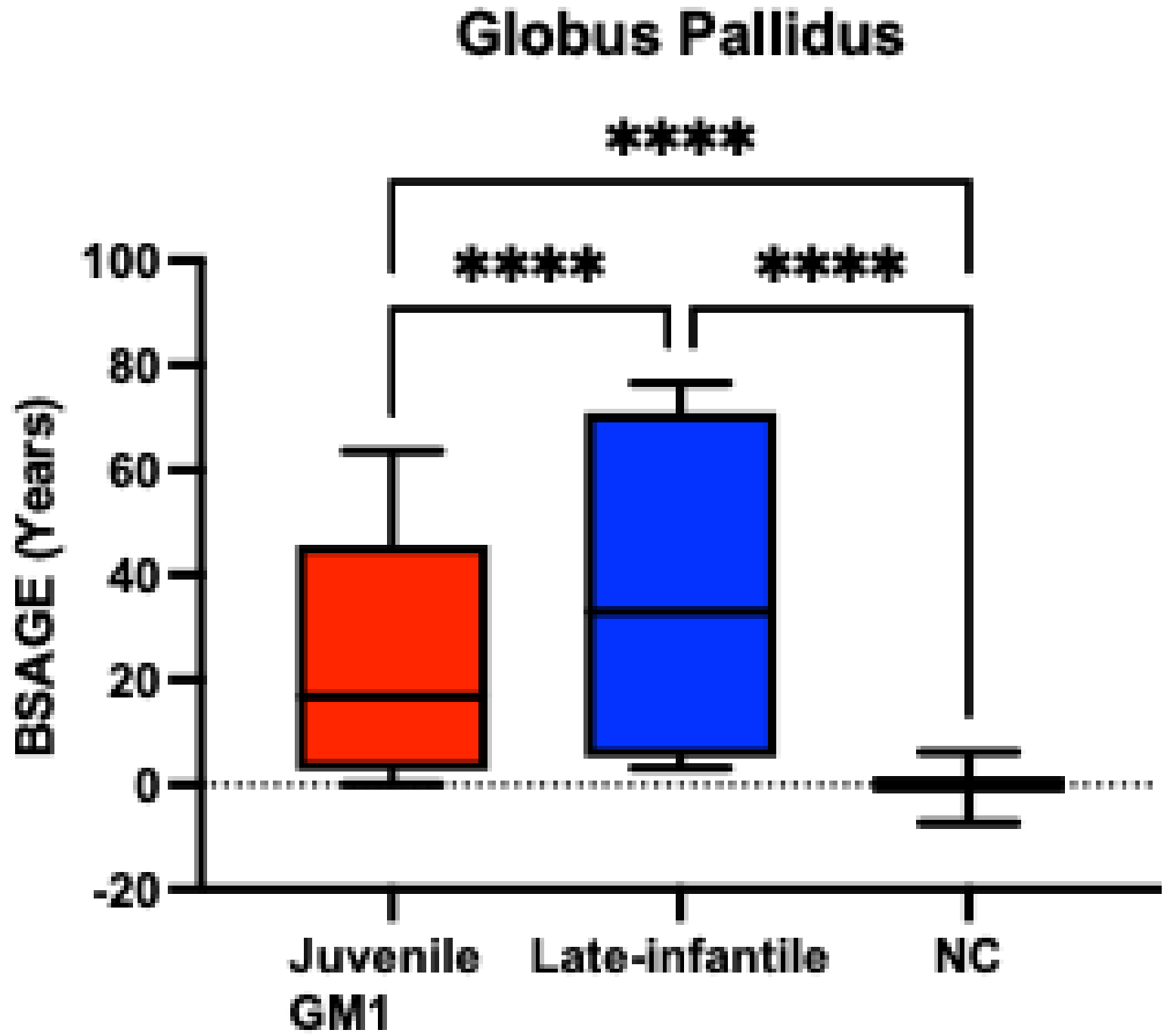

Supplement Figure 63. Comparison of the Globus Pallidus BSAGE between Juvenile GM1 patients, late-infantile GM1 patients, and neurotypical controls (NC). In all box and whisker plots, the center line represents the median, box limits represent quartiles, and error bars extend to the 1st and 99th percentiles. Late-infantile GM1 patients had an average BSAGE = 37.82, juvenile GM1 patients had an average BSAGE = 24.44, and NC participants had an average BSAGE = -0.10. An analysis of variance (ANOVA) showed statistically significant ($F(2, 595) = 438.0$, $p = 1.132e-117$) differences in BSAGE between the cohorts. A post-hoc Tukey test showed the late-infantile cohort had a statistically significantly higher BSAGE compared to both the neurotypical controls ($p = 4.75e-10$) and juvenile GM1 patients ($p = 4.74e-8$). The juvenile GM1 cohort also had a significantly higher BSAGE compared to the neurotypical controls ($p = 4.75e-10$). There were no adjustments made for multiple comparisons of BSAGE based on the number of brain structures analyzed.

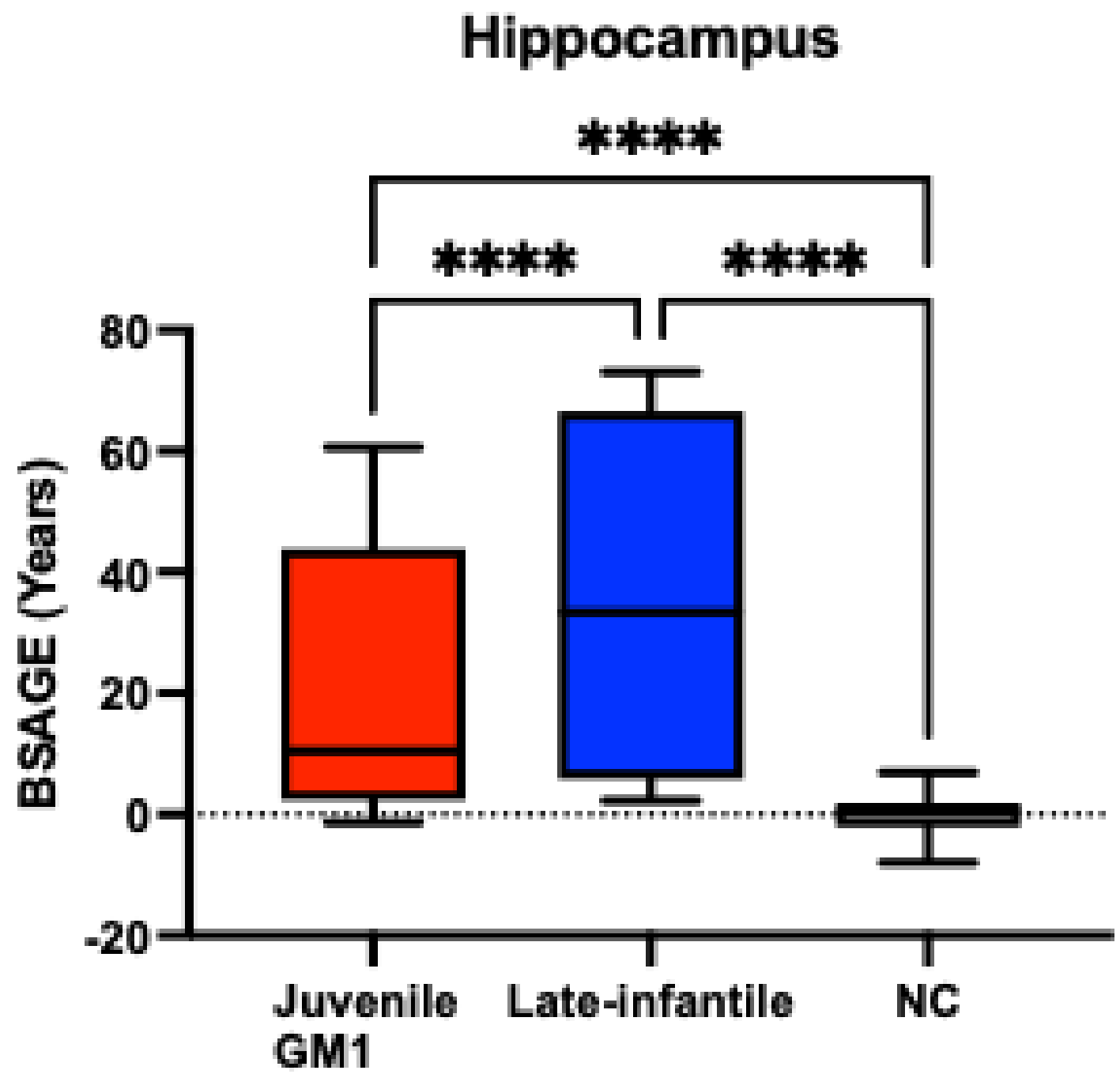

Supplement Figure 64. Comparison of the hippocampus BSAGE between Juvenile GM1 patients, late-infantile GM1 patients, and neurotypical controls (NC). In all box and whisker plots, the center line represents the median, box limits represent quartiles, and error bars extend to the 1st and 99th percentiles. Late-infantile GM1 patients had an average BSAGE = 38.10, juvenile GM1 patients had an average BSAGE = 21.87, and NC participants had an average BSAGE = 0.01. An analysis of variance (ANOVA) showed statistically significant ($F(2, 595) = 479.9$, $p = 7.864e-125$) differences in BSAGE between the cohorts. A post-hoc Tukey test showed the late-infantile cohort had a statistically significantly higher BSAGE compared to both the neurotypical controls ($p = 4.79e-10$) and juvenile GM1 patients ($p = 4.89e-10$). The juvenile GM1 cohort also had a significantly higher BSAGE compared to the neurotypical controls ($p = 4.79e-10$). There were no adjustments made for multiple comparisons of BSAGE based on the number of brain structures analyzed.

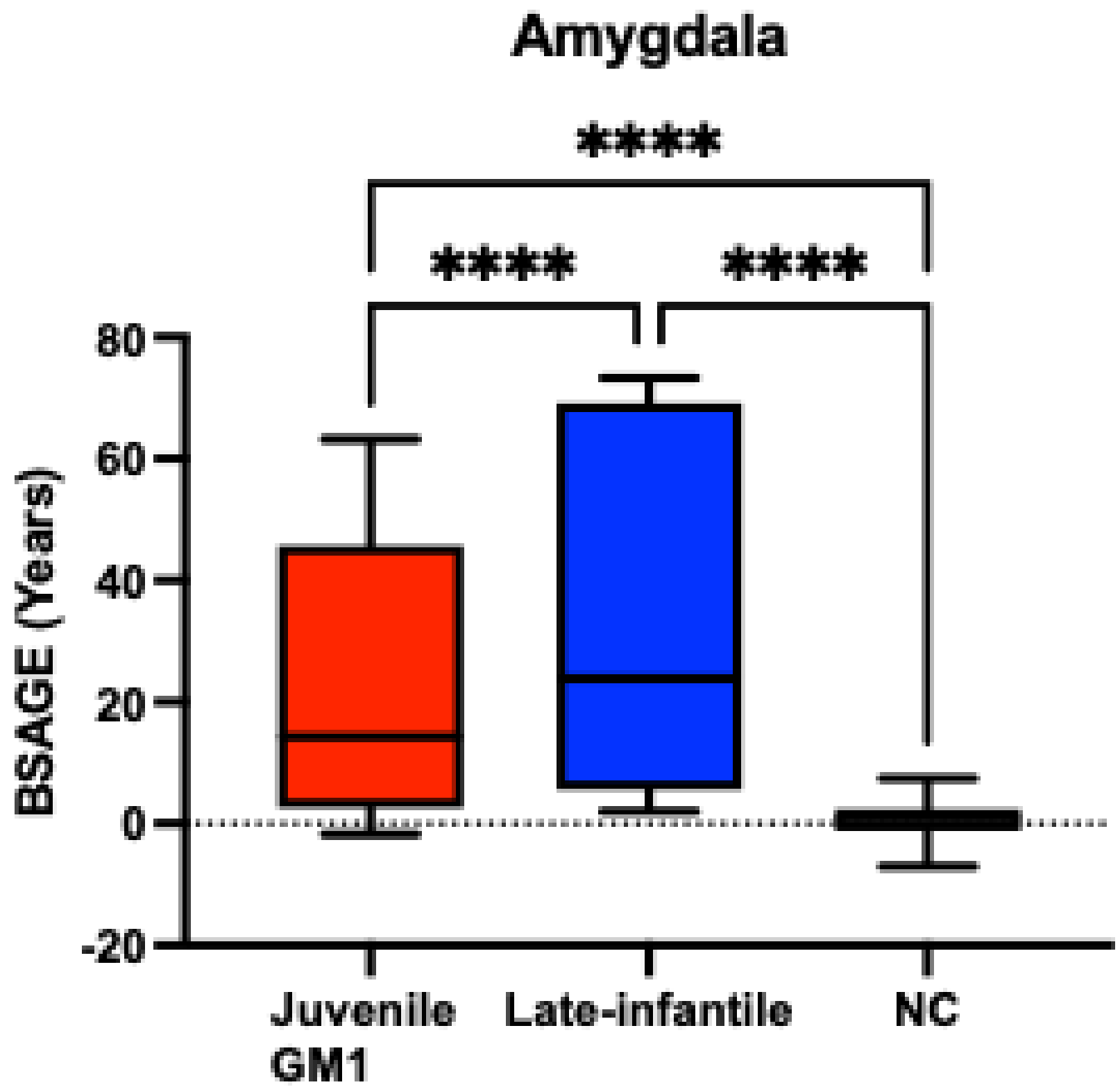

Supplement Figure 65. Comparison of the amygdala BSAGE between Juvenile GM1 patients, late-infantile GM1 patients, and neurotypical controls (NC). In all box and whisker plots, the center line represents the median, box limits represent quartiles, and error bars extend to the 1st and 99th percentiles. Late-infantile GM1 patients had an average BSAGE = 36.78, juvenile GM1 patients had an average BSAGE = 23.57, and NC participants had an average BSAGE = 0.55. An analysis of variance (ANOVA) showed statistically significant ($F(2, 595) = 514.7$, p = 1.728e-130) differences in BSAGE between the cohorts. A post-hoc Tukey test showed the late-infantile cohort had a statistically significantly higher BSAGE compared to both the neurotypical controls (p = 4.65e-10) and juvenile GM1 patients (p = 1.03e-7). The juvenile GM1 cohort also had a significantly higher BSAGE compared to the neurotypical controls (p = 4.65e-10). There were no adjustments made for multiple comparisons of BSAGE based on the number of brain structures analyzed.

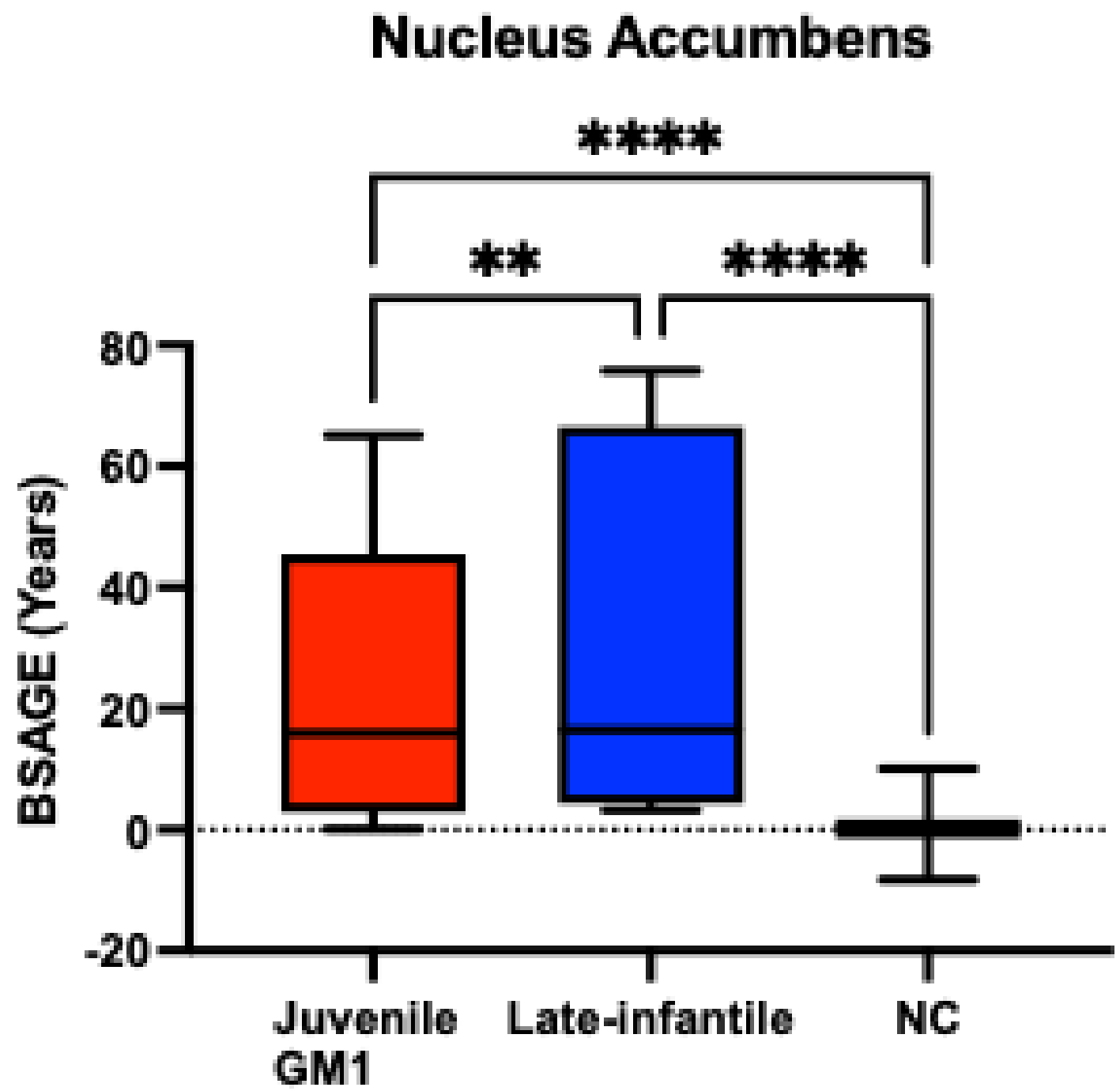

Supplement Figure 66. Comparison of the nucleus accumbens (NAC) BSAGE between Juvenile GM1 patients, late-infantile GM1 patients, and neurotypical controls (NC). In all box and whisker plots, the center line represents the median, box limits represent quartiles, and error bars extend to the 1st and 99th percentiles. Late-infantile GM1 patients had an average BSAGE = 30.56, juvenile GM1 patients had an average BSAGE = 23.46, and NC participants had an average BSAGE = 0.18. An analysis of variance (ANOVA) showed statistically significant ($F(2, 595) = 373.2$, $p = 9.337e-106$) differences in BSAGE between the cohorts. A post-hoc Tukey test showed the late-infantile cohort had a statistically significantly higher BSAGE compared to both the neurotypical controls ($p = 4.75e-10$) and juvenile GM1 patients ($p = 0.0055$). The juvenile GM1 cohort also had a significantly higher BSAGE compared to the neurotypical controls ($p = 4.75e-10$). There were no adjustments made for multiple comparisons of BSAGE based on the number of brain structures analyzed.

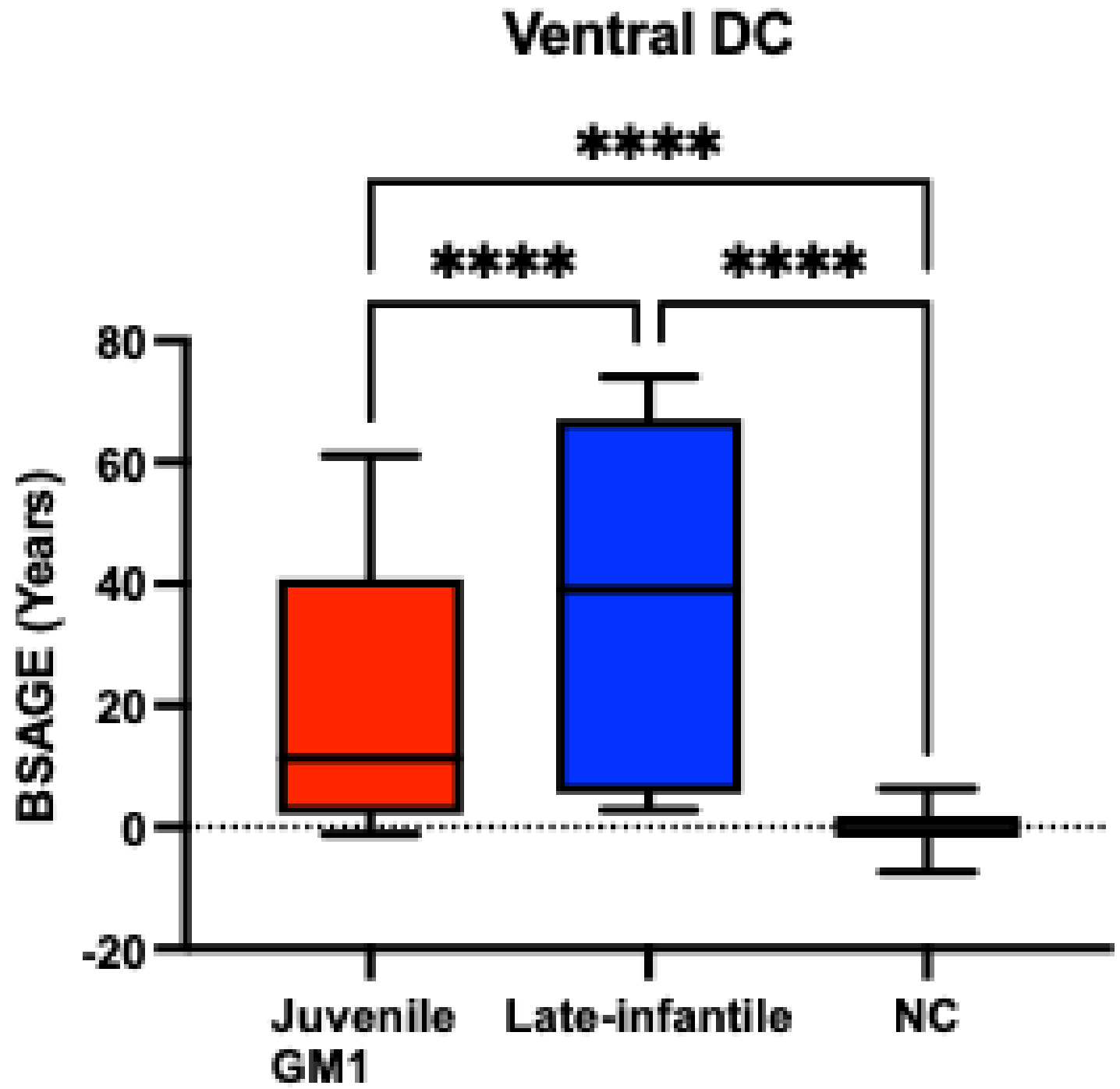

Supplement Figure 67. Comparison of the ventral diencephalon BSAGE between Juvenile GM1 patients, late-infantile GM1 patients, and neurotypical controls (NC). In all box and whisker plots, the center line represents the median, box limits represent quartiles, and error bars extend to the 1st and 99th percentiles. Late-infantile GM1 patients had an average BSAGE = 39.12, juvenile GM1 patients had an average BSAGE = 21.10, and NC participants had an average BSAGE = 0.08. An analysis of variance (ANOVA) showed statistically significant ($F(2, 595) = 532.3$, $p < 2.936e\text{-}133$) differences in BSAGE between the cohorts. A post-hoc Tukey test showed the late-infantile cohort had a statistically significantly higher BSAGE compared to both the neurotypical controls (p = 4.75e-10) and juvenile GM1 patients (p = 4.75e-10). The juvenile GM1 cohort also had a significantly higher BSAGE compared to the neurotypical controls (p = 4.75e-10). There were no adjustments made for multiple comparisons of BSAGE based on the number of brain structures analyzed.

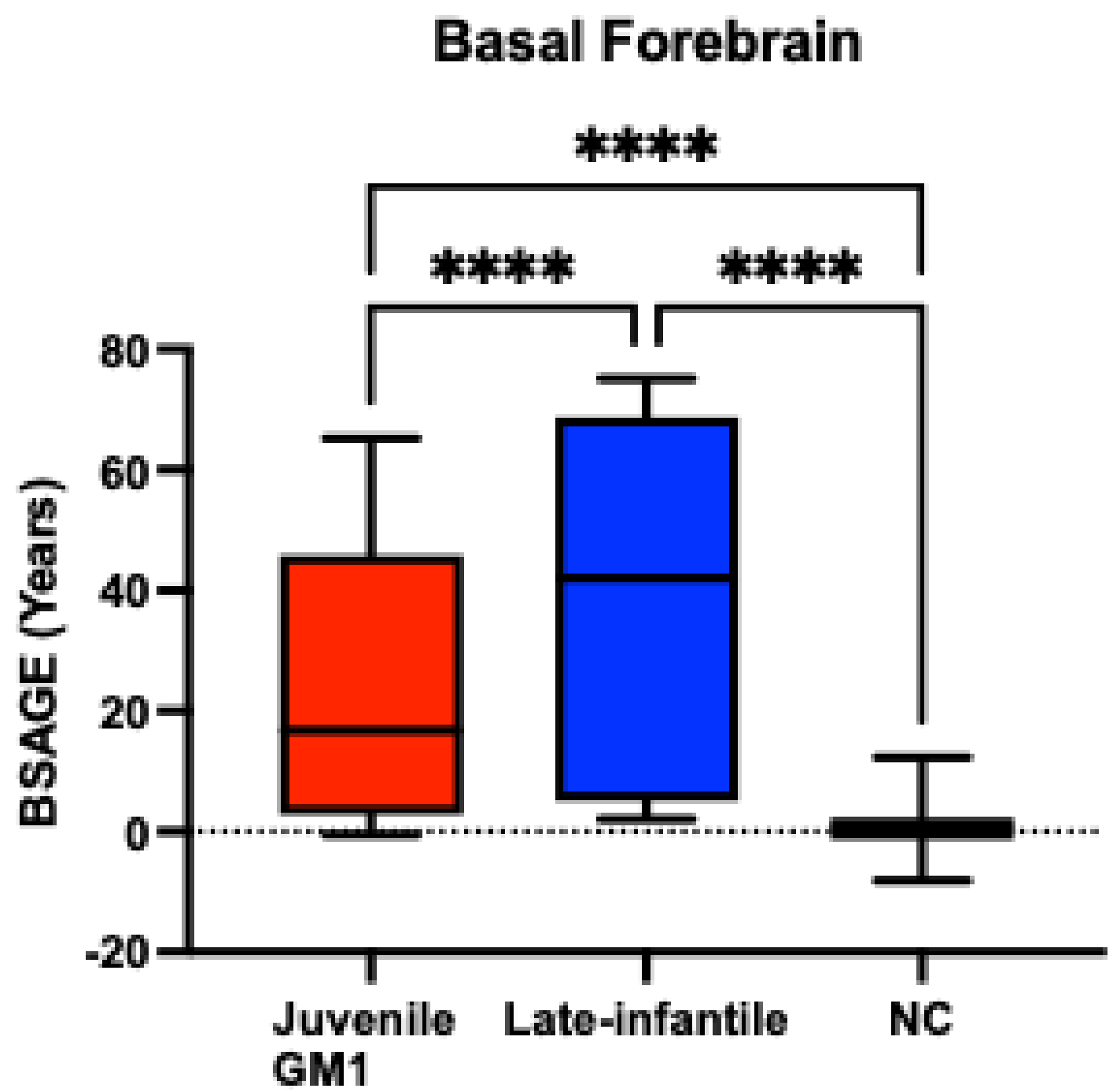

Supplement Figure 68. Comparison of the basal forebrain BSAGE between Juvenile GM1 patients, late-infantile GM1 patients, and neurotypical controls (NC). In all box and whisker plots, the center line represents the median, box limits represent quartiles, and error bars extend to the 1st and 99th percentiles. Late-infantile GM1 patients had an average BSAGE = 39.00, juvenile GM1 patients had an average BSAGE = 24.66, and NC participants had an average BSAGE = 0.45. An analysis of variance (ANOVA) showed statistically significant ($F(2, 595) = 717.8$, $p = 2.515e-159$) differences in BSAGE between the cohorts. A post-hoc Tukey test showed the late-infantile cohort had a statistically significantly higher BSAGE compared to both the neurotypical controls ($p = 4.75e-10$) and juvenile GM1 patients ($p = 4.75e-10$). The juvenile GM1 cohort also had a significantly higher BSAGE compared to the neurotypical controls ($p = 4.75e-10$). There were no adjustments made for multiple comparisons of BSAGE based on the number of brain structures analyzed.

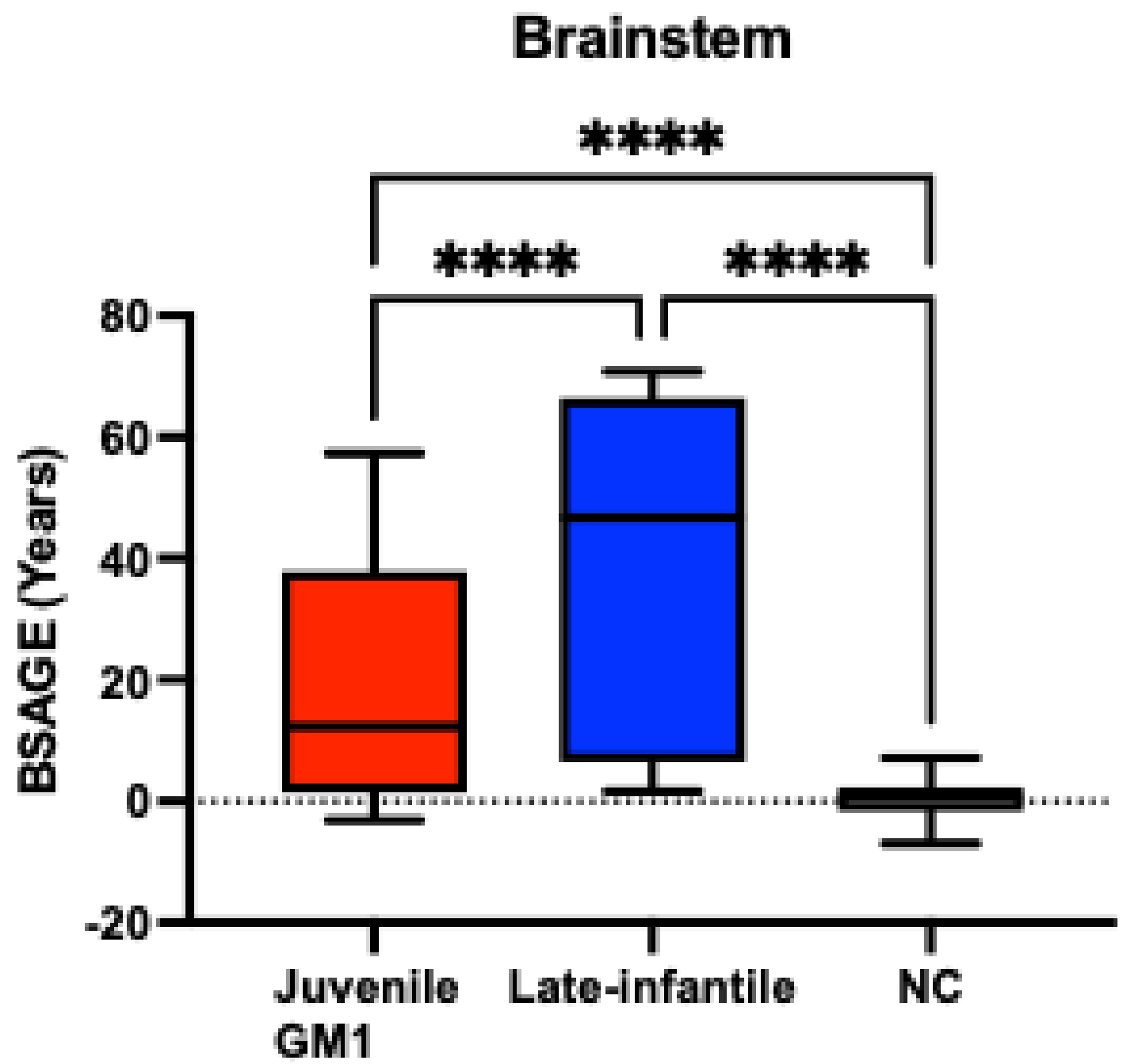

Supplement Figure 69. Comparison of the brainstem BSAGE between Juvenile GM1 patients, late-infantile GM1 patients, and neurotypical controls (NC). In all box and whisker plots, the center line represents the median, box limits represent quartiles, and error bars extend to the 1st and 99th percentiles. Late-infantile GM1 patients had an average BSAGE = 38.56, juvenile GM1 patients had an average BSAGE = 19.56, and NC participants had an average BSAGE = 0.40. An analysis of variance (ANOVA) showed statistically significant ($F(2, 595) = 429.3$, p = 3.903e-116) differences in BSAGE between the cohorts. A post-hoc Tukey test showed the late-infantile cohort had a statistically significantly higher BSAGE compared to both the neurotypical controls (p = 4.65e-10) and juvenile GM1 patients (p = 4.65e-10). The juvenile GM1 cohort also had a significantly higher BSAGE compared to the neurotypical controls (p = 4.65e-10). There were no adjustments made for multiple comparisons of BSAGE based on the number of brain structures analyzed.

Supplement J: Comparisons Between Predicted Brain Age and Brain Volumetrics

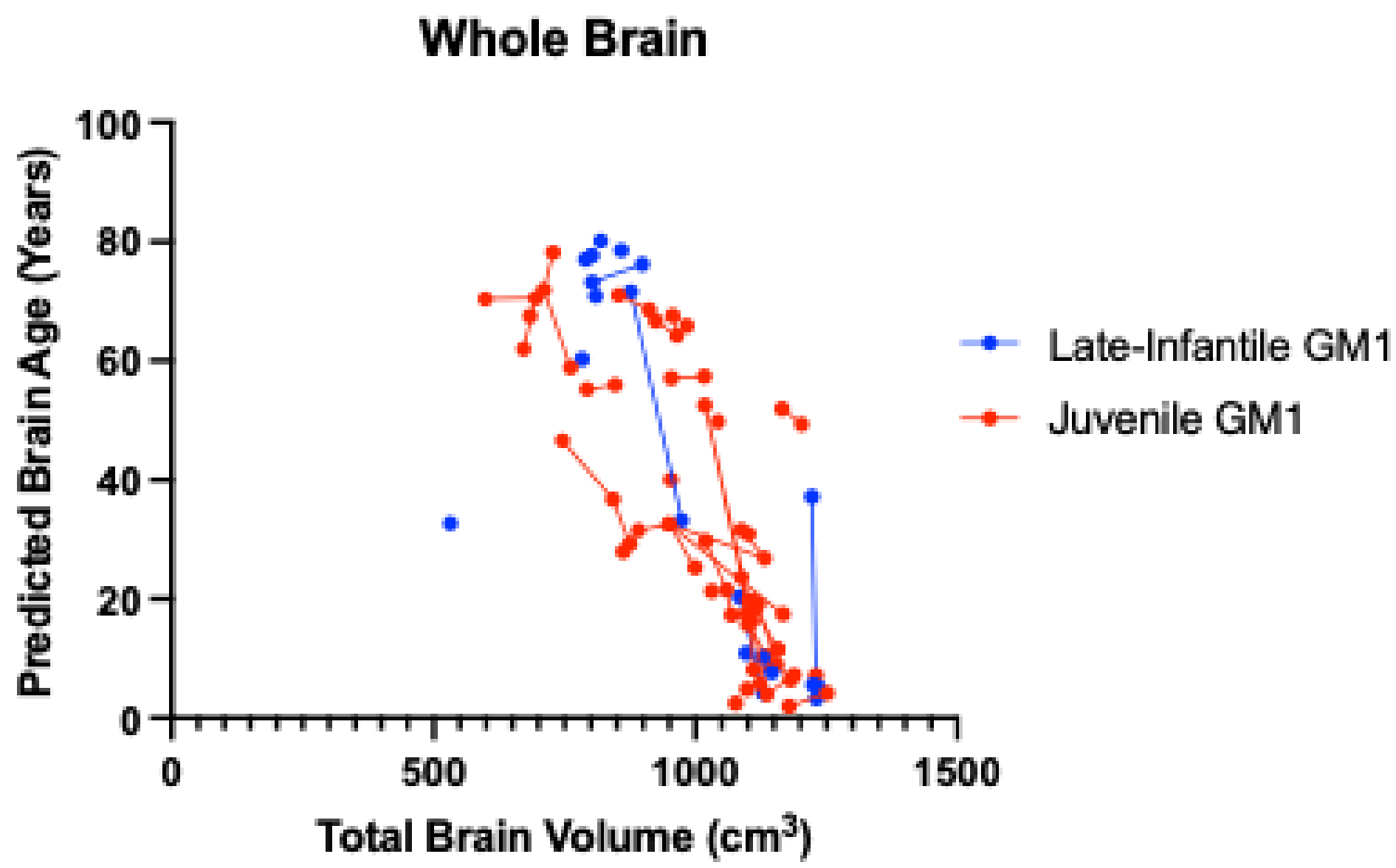

Supplement Figure 70. Correlation between whole brain volume (cm^3) and predicted whole brain age in juvenile and late-infantile GM1 patients. Total brain volume correlated with predicted whole brain age in both the juvenile ($R^2 = 0.57$) and late-infantile ($R^2 = 0.46$) patients.

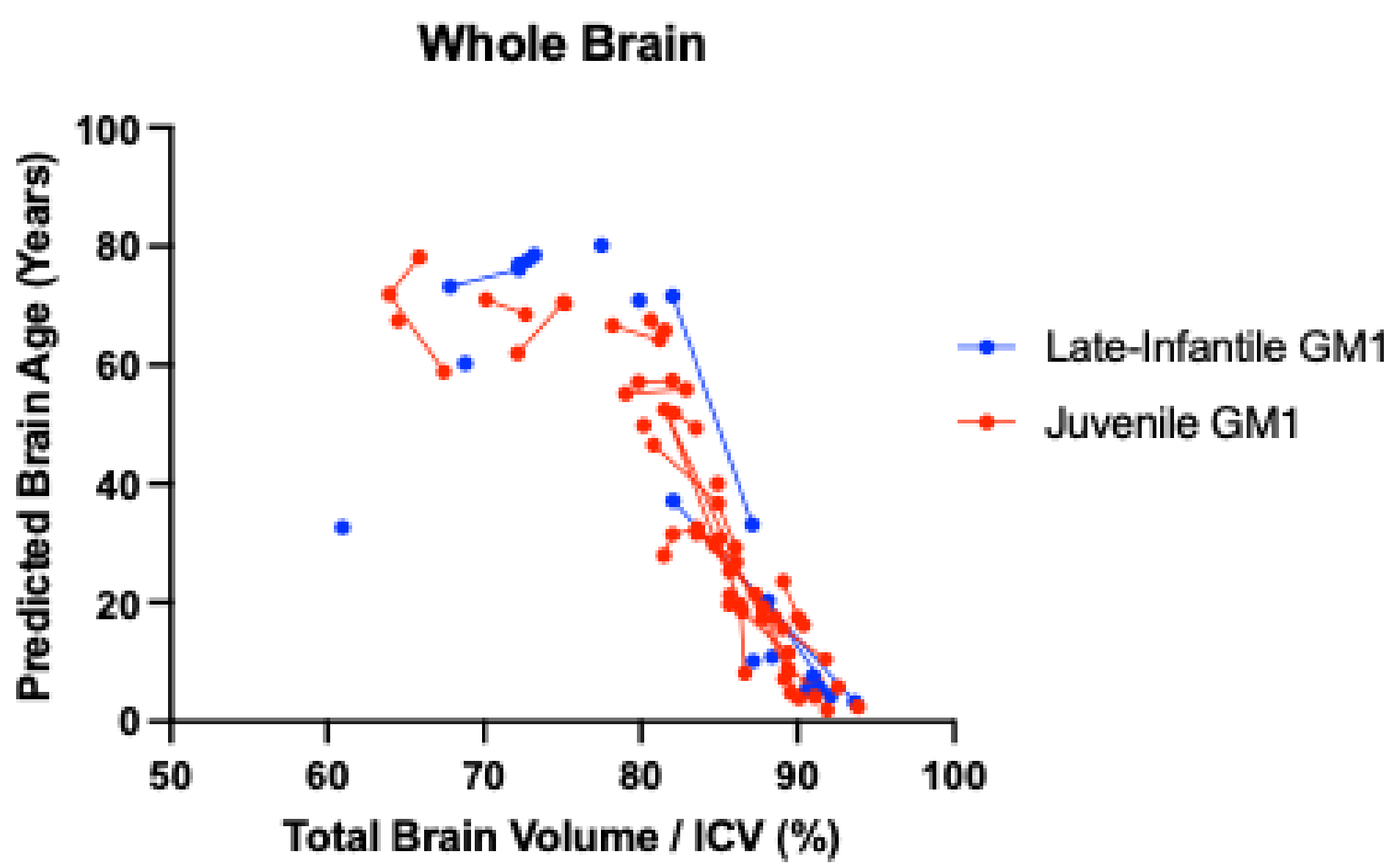

Supplement Figure 71. Correlation between whole brain volume controlled for total intracranial volume (ICV) and predicted whole brain age in juvenile and late-infantile GM1 patients. Total brain volume correlated with predicted whole brain age in both the juvenile ($R^2 = 0.77$) and late-infantile ($R^2 = 0.51$) patients.

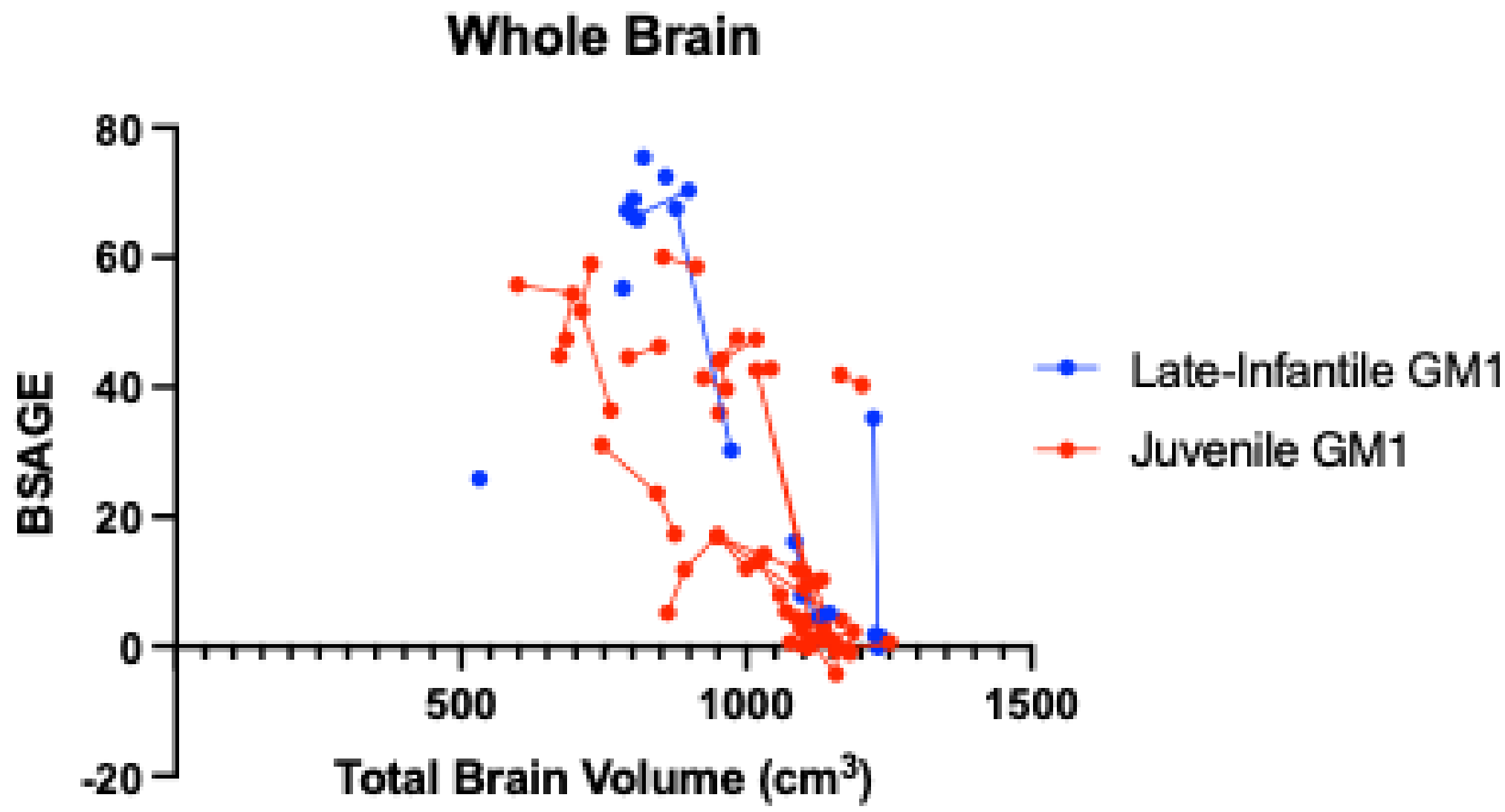

Supplement Figure 72. Correlation between whole brain volume (cm^3) and predicted whole brain, brain structures age gap estimation (BSAGE) in juvenile and late-infantile GM1 patients. Total brain volume correlated with predicted whole brain age in both the juvenile ($R^2 = 0.49$) and late-infantile ($R^2 = 0.43$) patients.

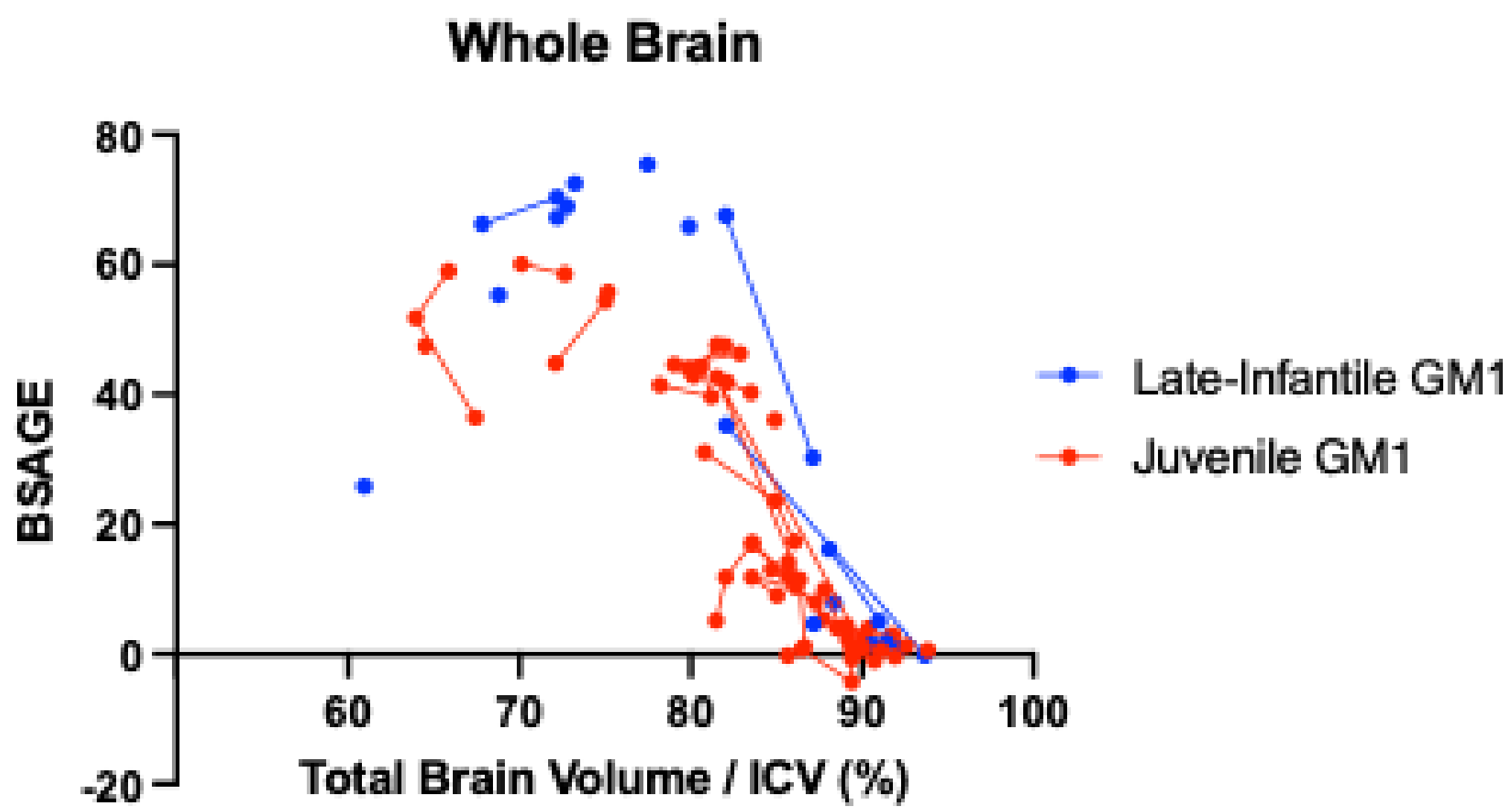

Supplement Figure 73. Correlation between whole brain volume controlled for total intracranial volume (ICV) and predicted whole brain, brain structures age gap estimation (BSAGE) in juvenile and late-infantile GM1 patients. Total brain volume correlated with predicted whole brain age in both the juvenile ($R^2 = 0.69$) and late-infantile ($R^2 = 0.47$) patients.

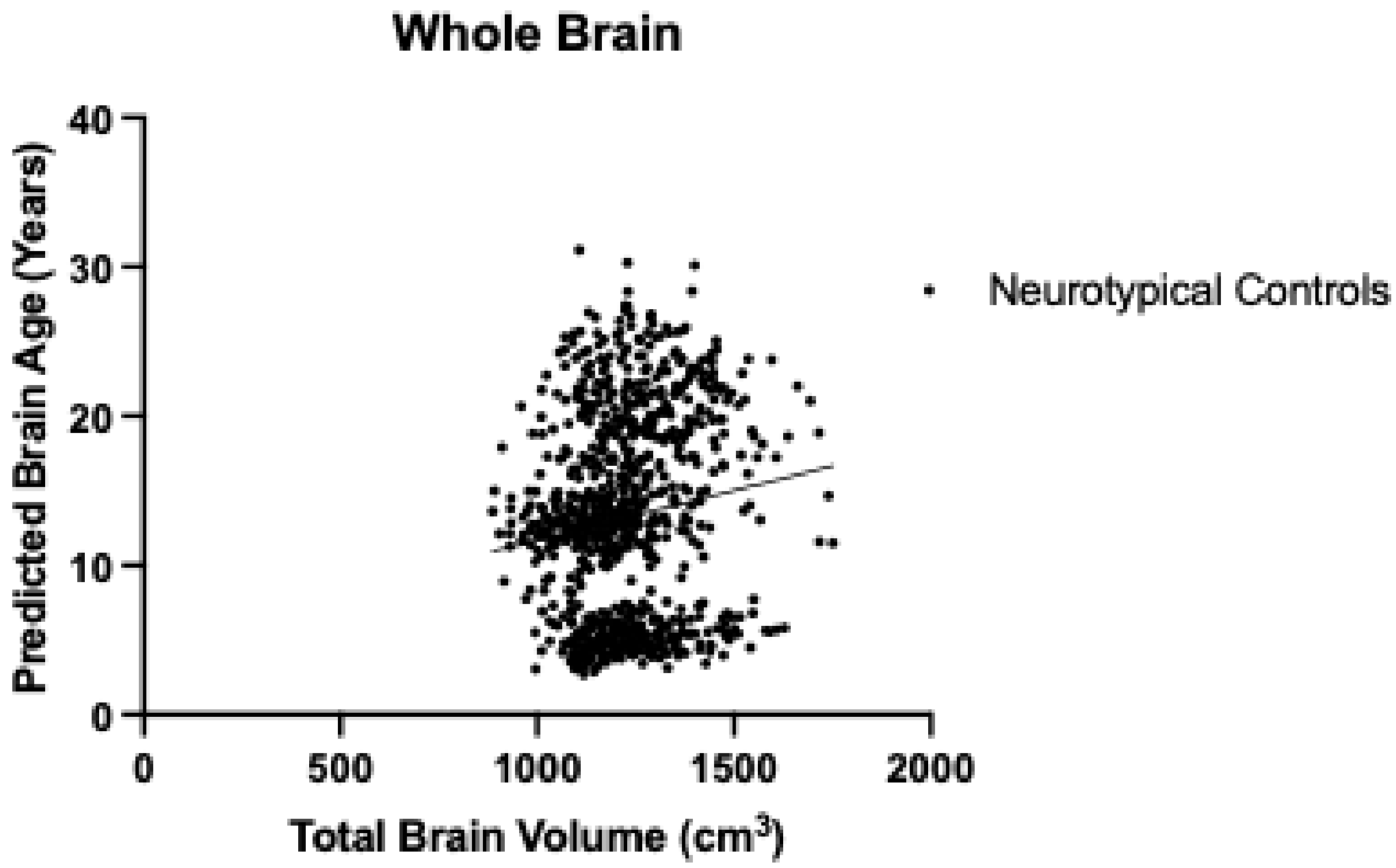

Supplement Figure 74. Correlation between whole brain volume (cm^3) and predicted whole brain age in neurotypical controls. Total brain volume correlated with predicted whole brain age in the neurotypical controls ($R^2 = 0.02$).

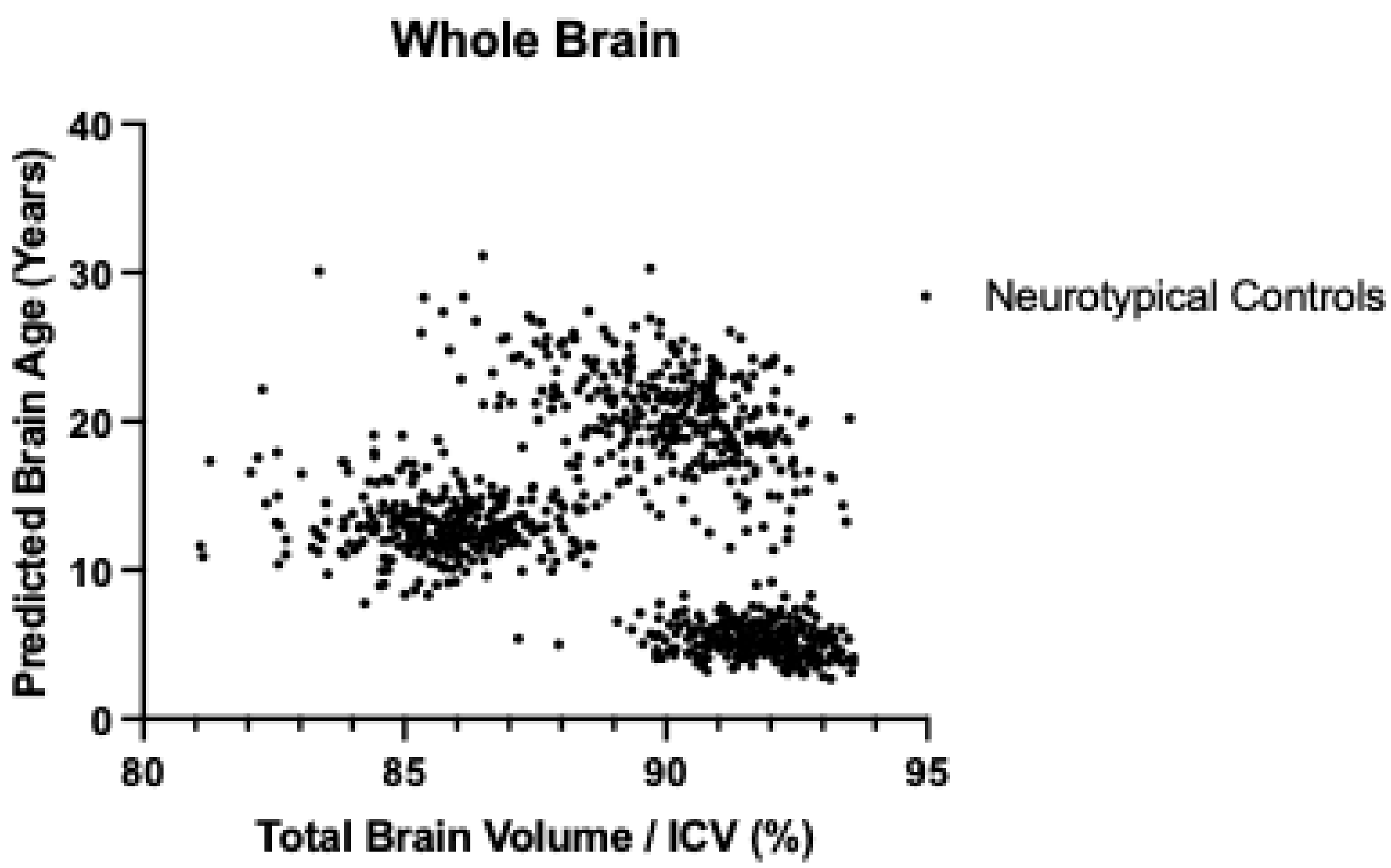

Supplement Figure 75. Correlation between whole brain volume controlled for total intracranial volume (ICV) and predicted whole brain age in neurotypical controls. Total brain volume correlated with predicted whole brain age in the neurotypical controls ($R^2 = 0.07$).

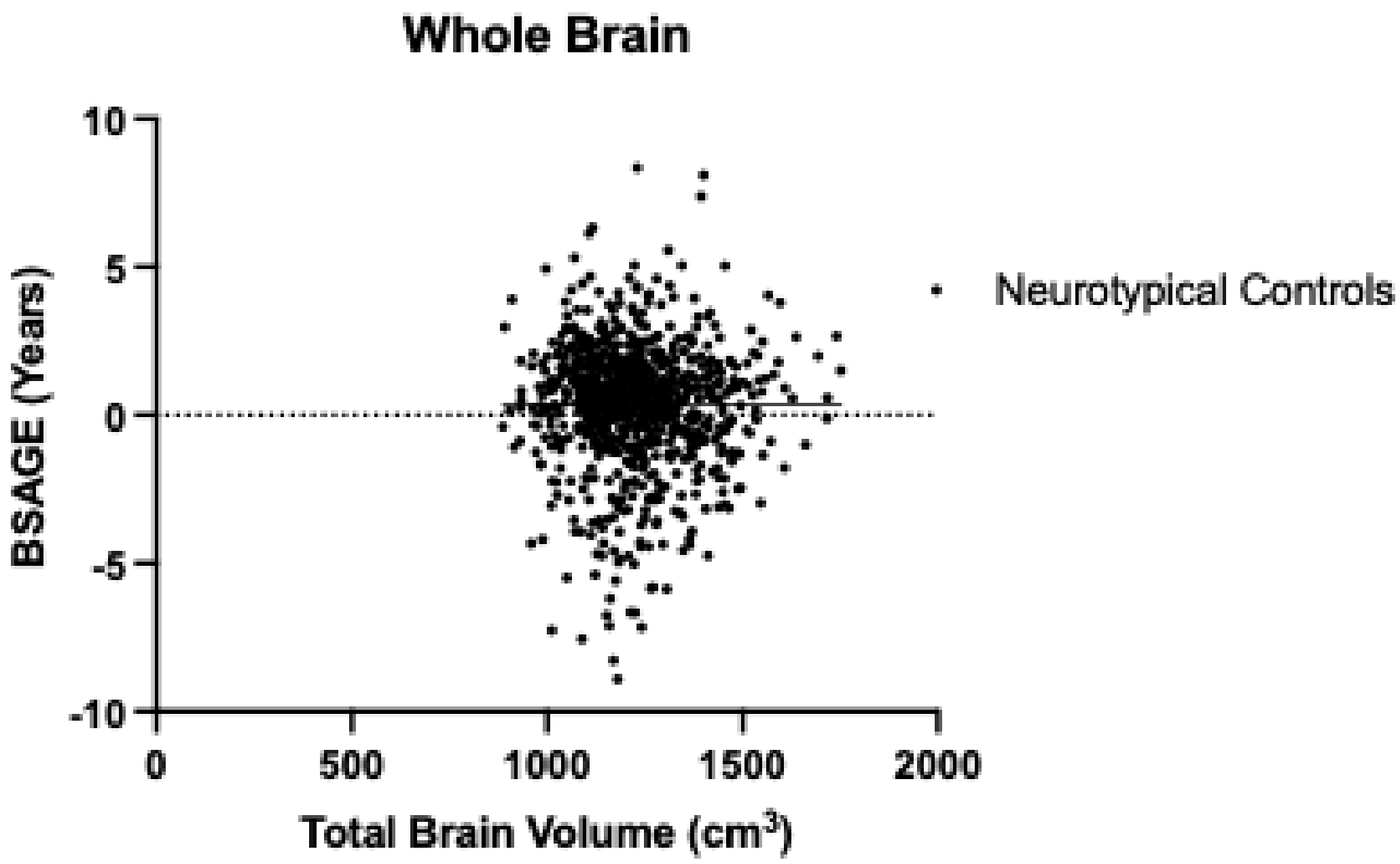

Supplement Figure 76. Correlation between whole brain volume (cm^3) and predicted whole brain, brain structures age gap estimation (BSAGE) in neurotypical controls. Total brain volume did not correlate with predicted whole brain age in the neurotypical controls ($R^2 < 0.01$).

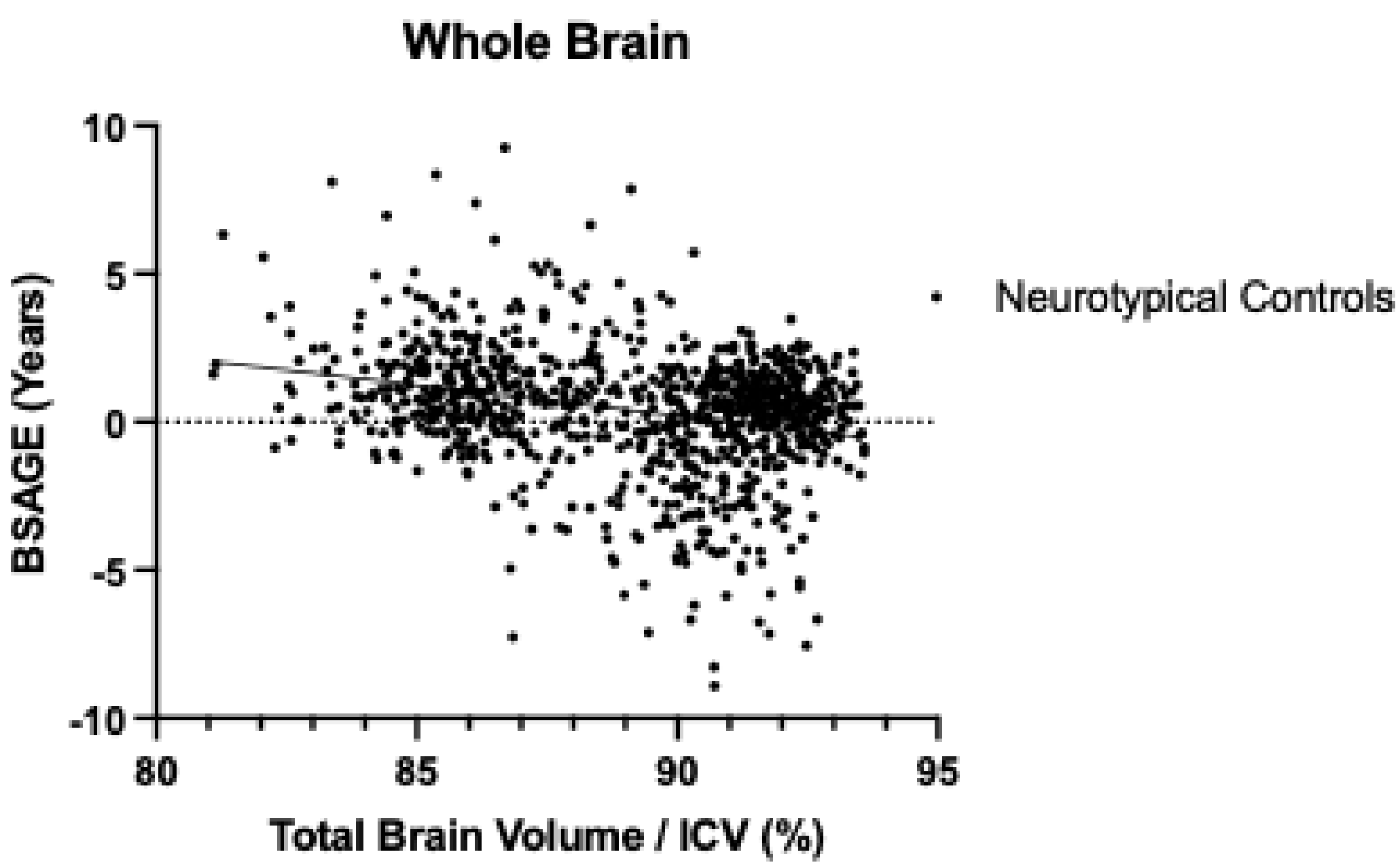

Supplement Figure 77. Correlation between whole brain volume controlled for total intracranial volume (ICV) and predicted whole brain, brain structures age gap estimation (BSAGE) in neurotypical controls. Total brain volume correlated with predicted whole brain age in the neurotypical controls ($R^2 = 0.08$).

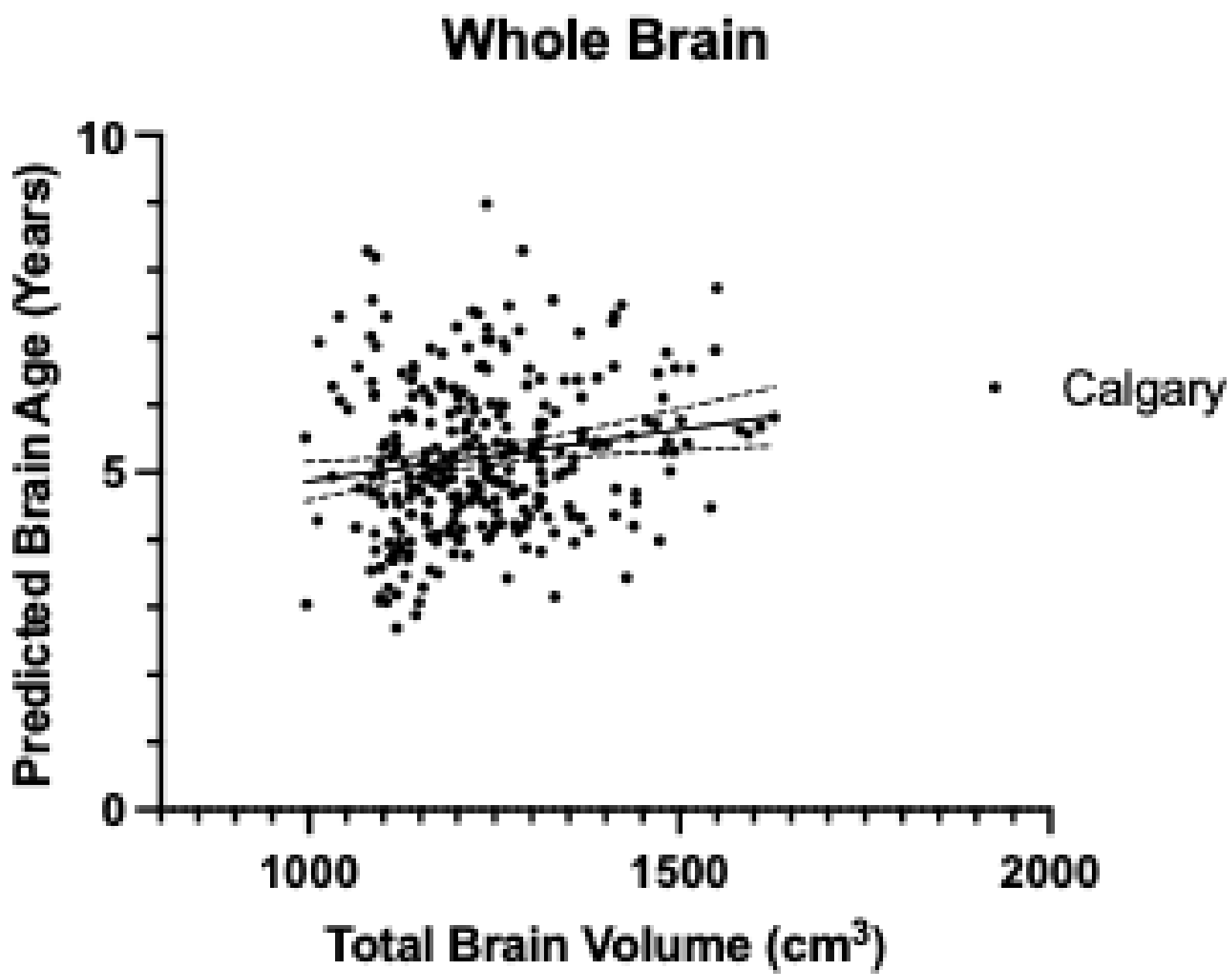

Supplement Figure 78. Correlation between whole brain volume (cm^3) and predicted whole brain age in Calgary neurotypical controls. Total brain volume did correlated with predicted whole brain age in Calgary neurotypical controls ($R^2 = 0.03$).

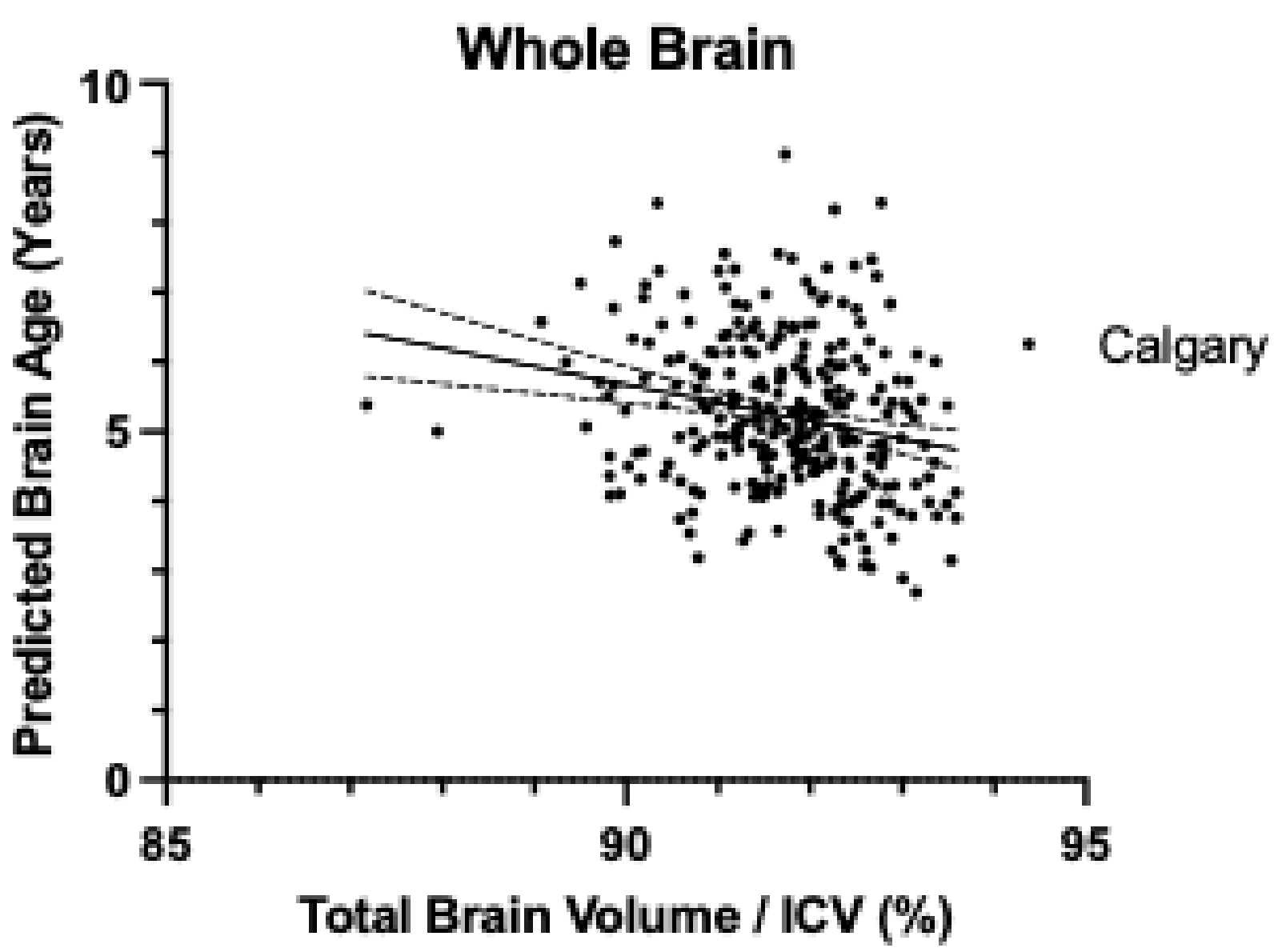

Supplement Figure 79. Correlation between whole brain volume controlled for total intracranial volume (ICV) and predicted whole brain, brain structures age gap estimation (BSAGE) in Calgary neurotypical controls. Total brain volume correlated with predicted whole brain age in the Calgary neurotypical controls ($R^2 = 0.05$).

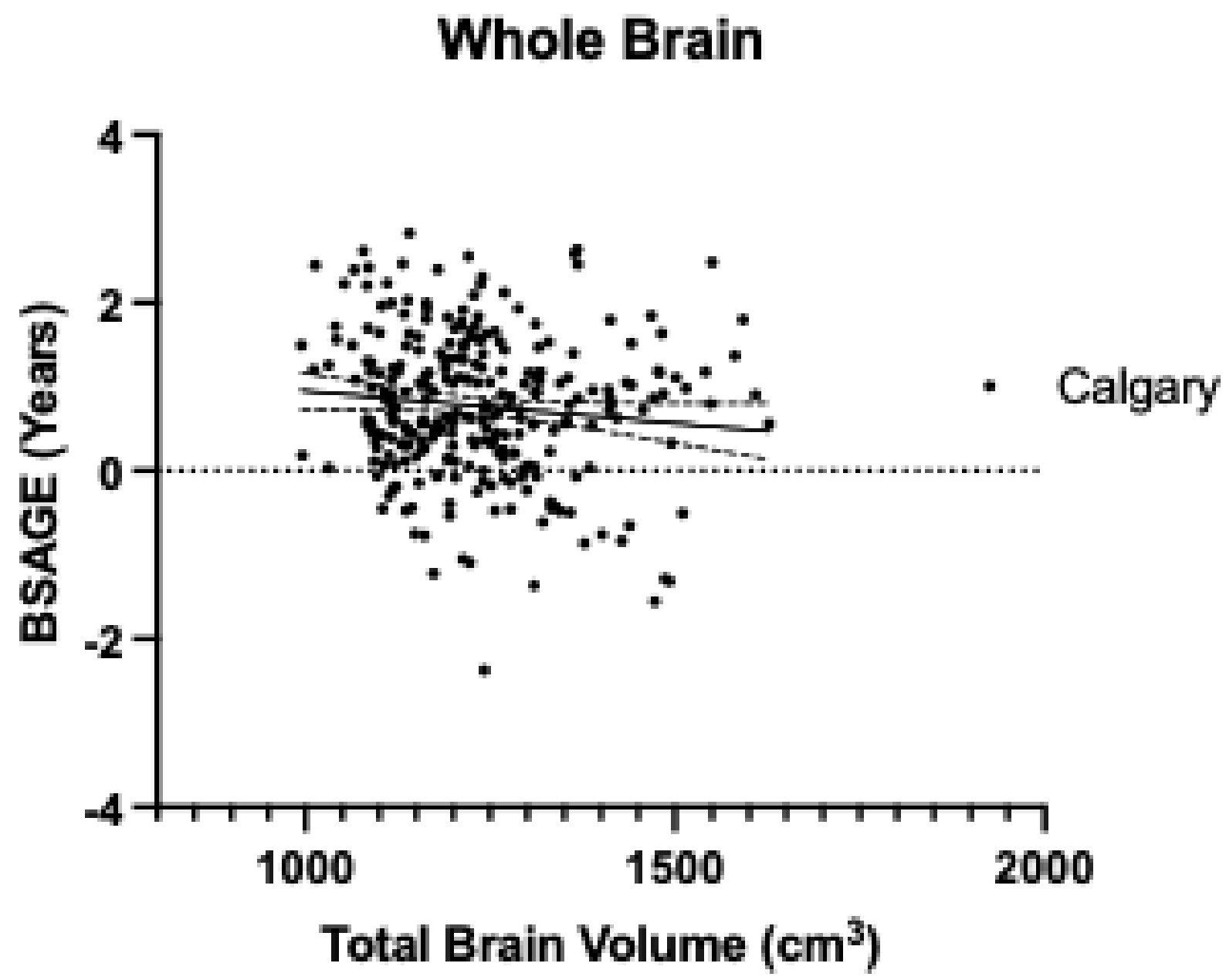

Supplement Figure 80. Correlation between whole brain volume (cm^3) and predicted whole brain, brain structures age gap estimation (BSAGE) in neurotypical controls. Total brain volume did not correlate with predicted whole brain age in the neurotypical controls ($R^2 = 0.01$).

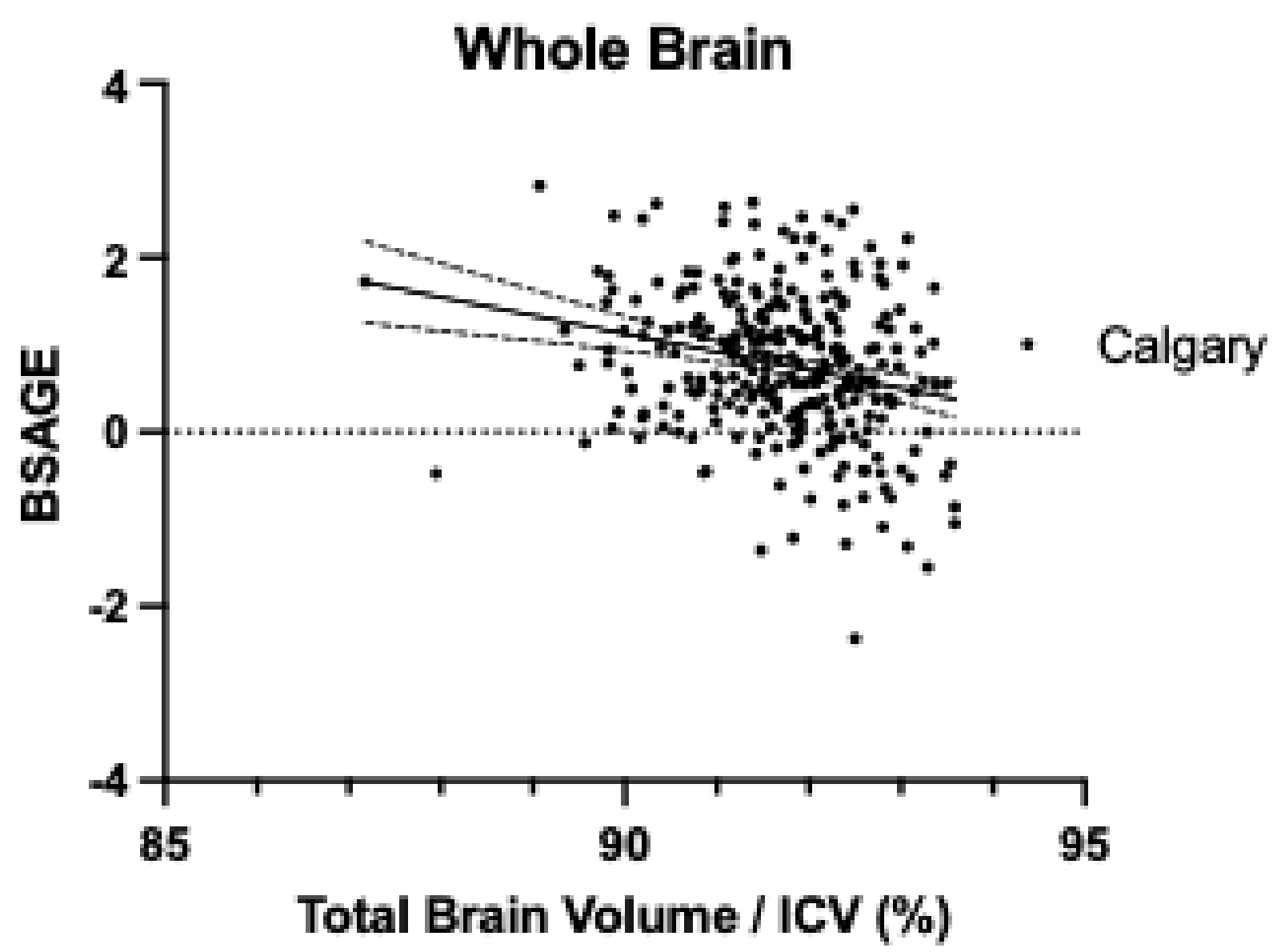

Supplement Figure 81. Correlation between whole brain volume controlled for ICV and predicted whole brain, brain structures age gap estimation (BSAGE) in neurotypical controls. Total brain volume did not correlate with predicted whole brain age in the neurotypical controls ($R^2 = 0.06$).

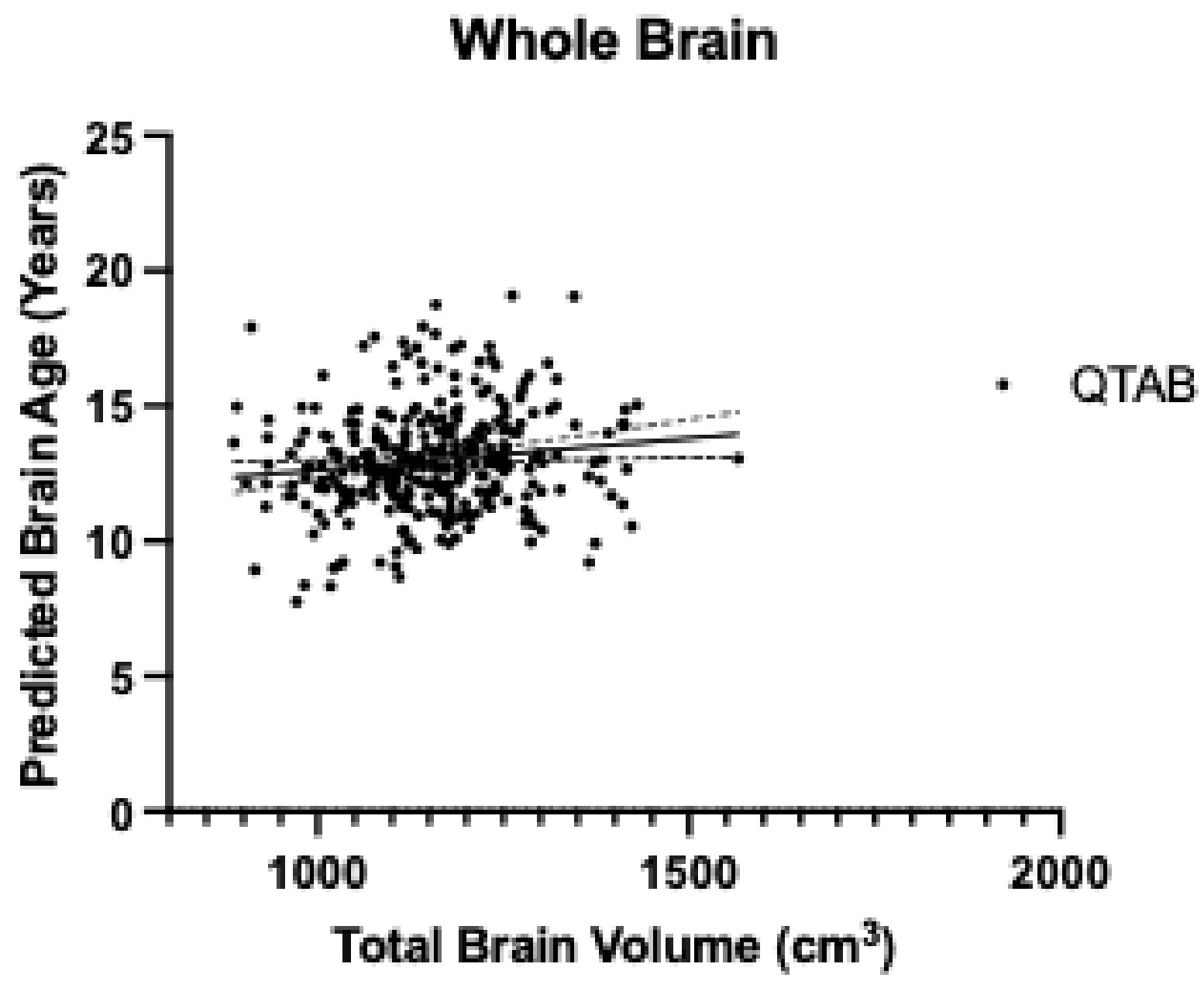

Supplement Figure 82. Correlation between whole brain volume and predicted whole brain age gap estimation in QTAB neurotypical controls. Total brain volume correlated with predicted whole brain age in the QTAB neurotypical controls ($R^2 = 0.02$).

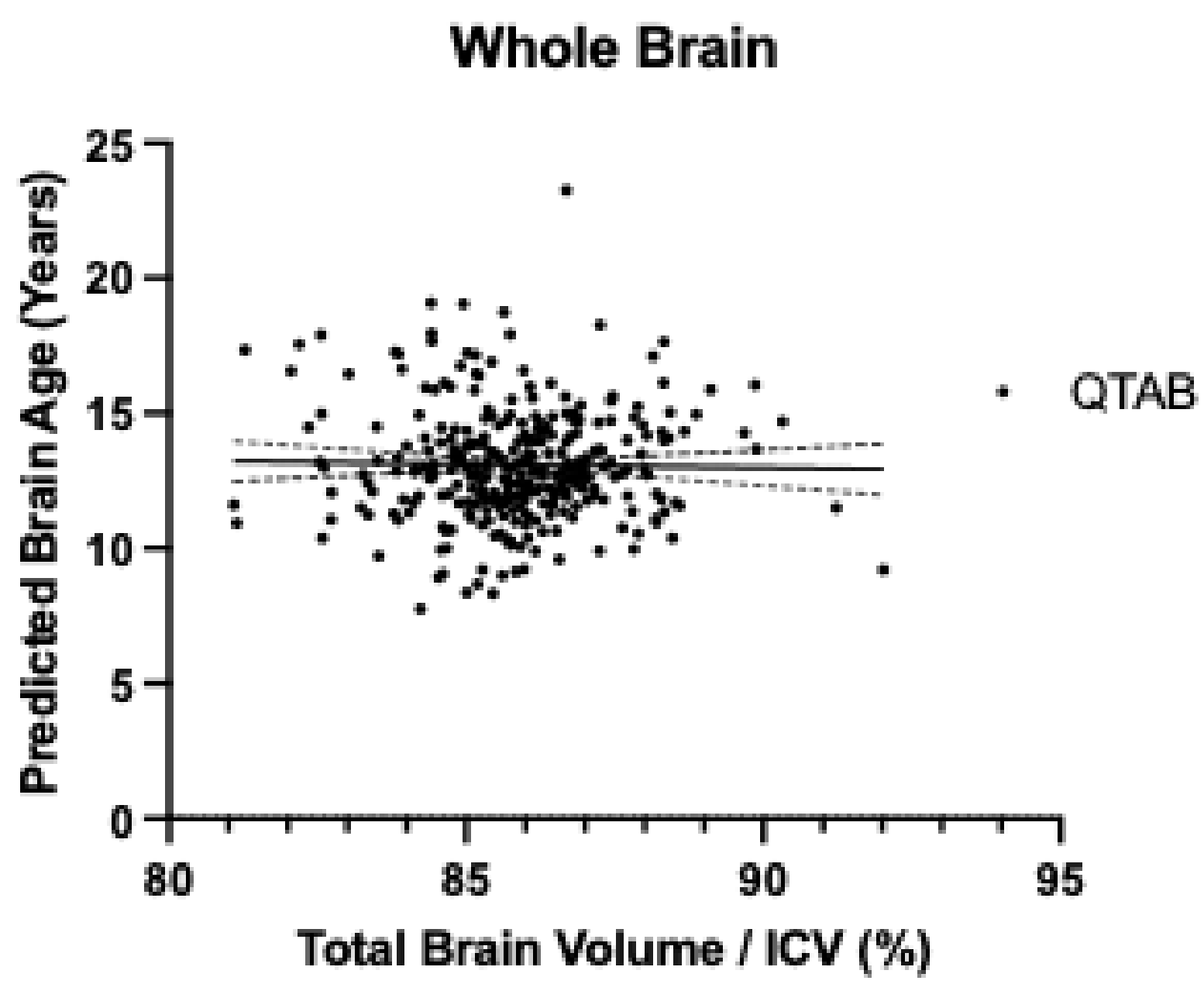

Supplement Figure 83. Correlation between whole brain volume controlled for total intracranial volume (ICV) and predicted whole brain age gap estimation in QTAB neurotypical controls. Total brain volume correlated with predicted whole brain age in the QTAB neurotypical controls ($R^2 < 0.01$).

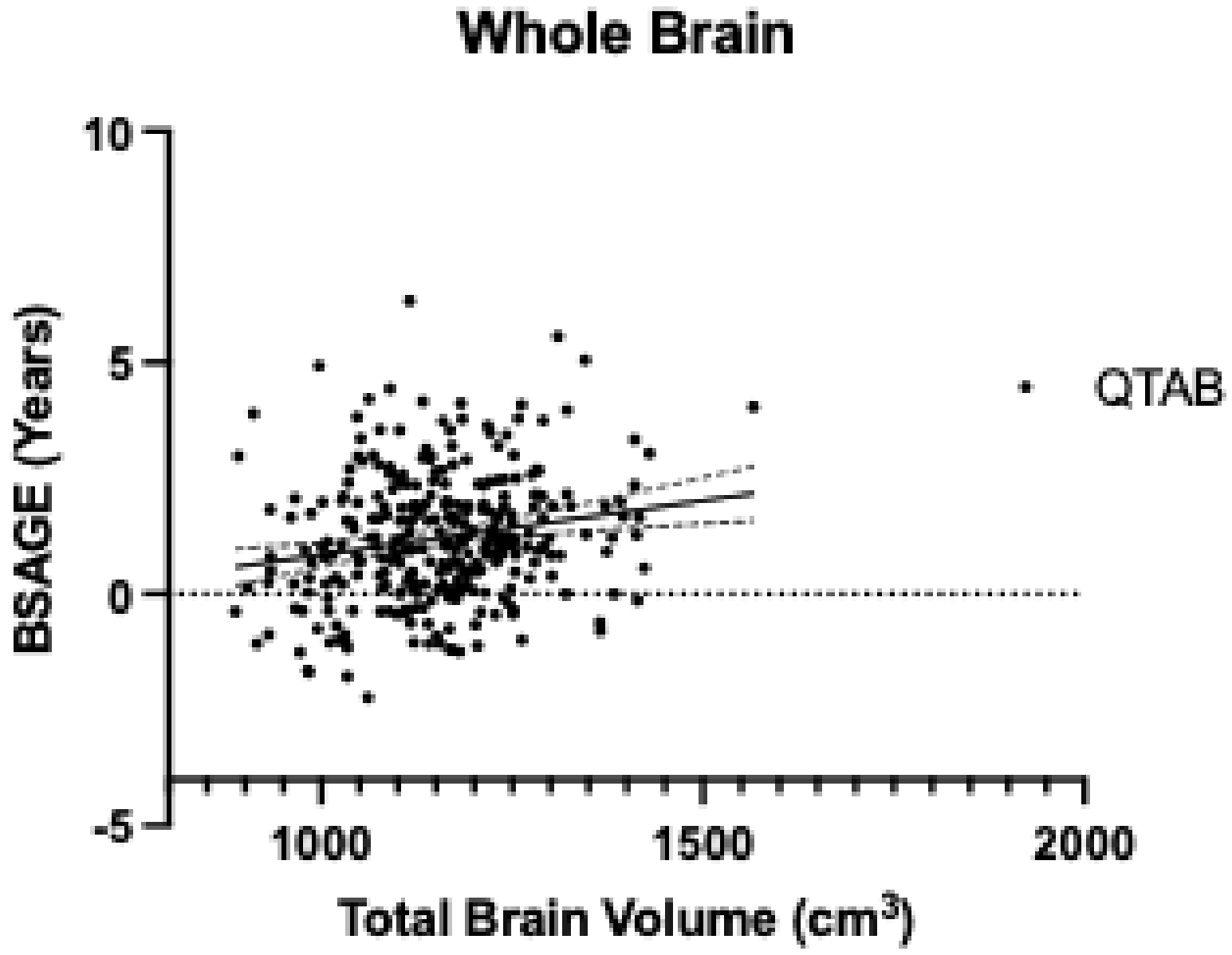

Supplement Figure 84. Correlation between whole brain volume (cm^3) and predicted whole brain, brain structures age gap estimation (BSAGE) in the QTAB neurotypical controls. Total brain volume correlated with predicted whole brain age in the QTAB neurotypical controls (R^2 = 0.04).

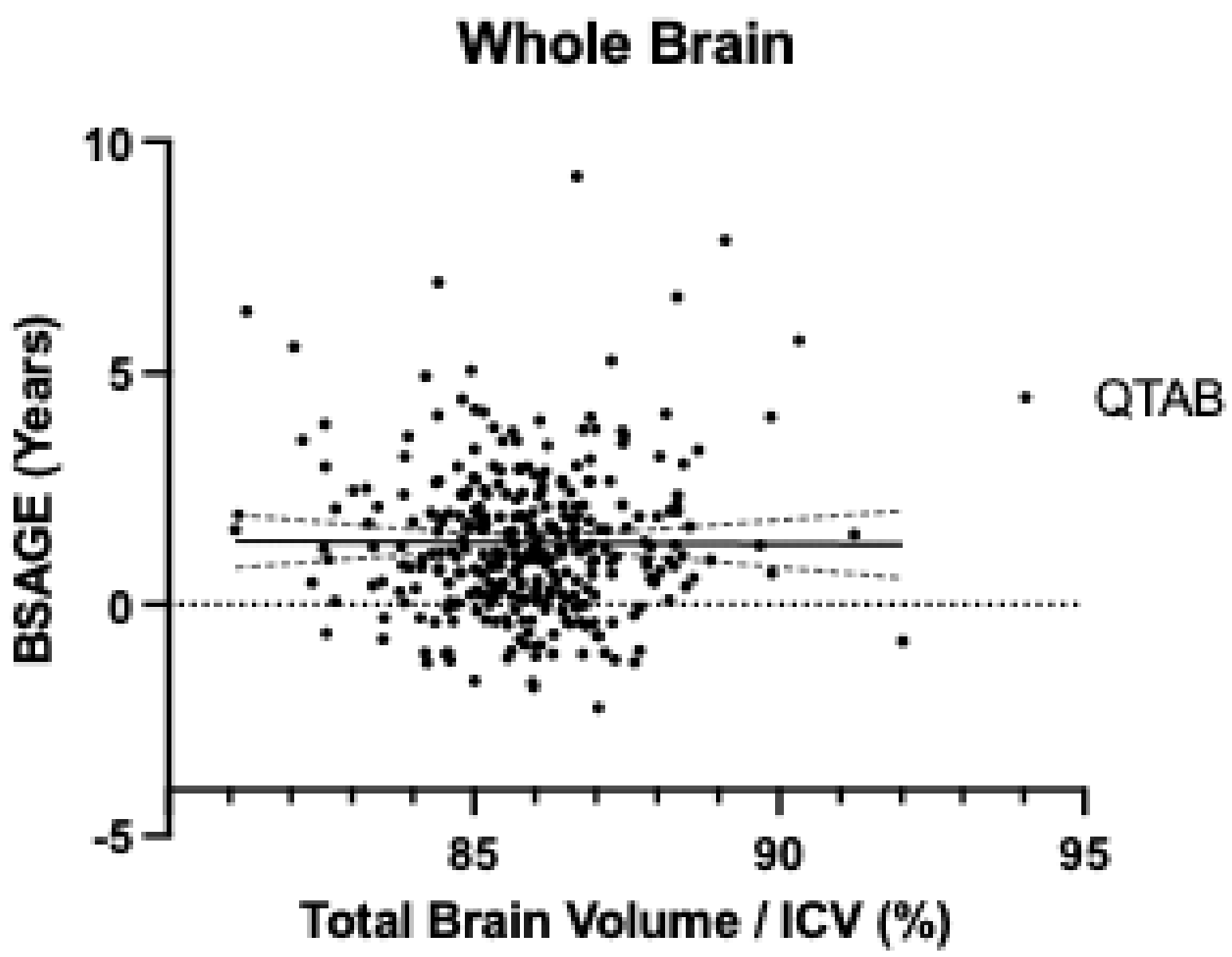

Supplement Figure 85. Correlation between whole brain volume controlled for total intracranial volume (ICV) and predicted whole brain, brain structures age gap estimation (BSAGE) in the QTAB neurotypical controls. Total brain volume did not correlate with predicted whole brain age in the QTAB neurotypical controls ($R^2 < 0.01$).

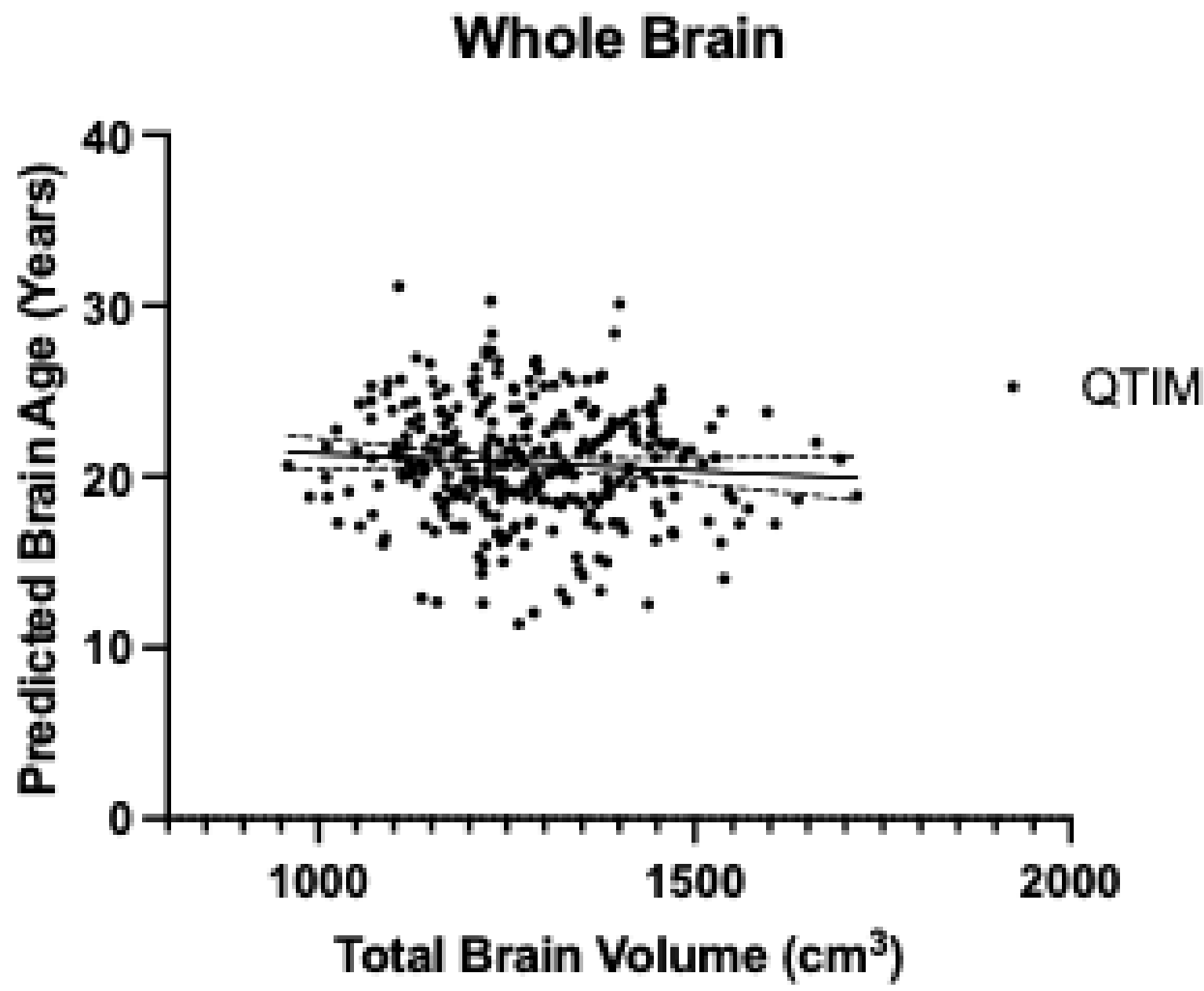

Supplement Figure 86. Correlation between whole brain volume and predicted whole brain age gap estimation in QTIM neurotypical controls. Total brain volume correlated with predicted whole brain age in the QTIM neurotypical controls ($R^2 = 0.01$).

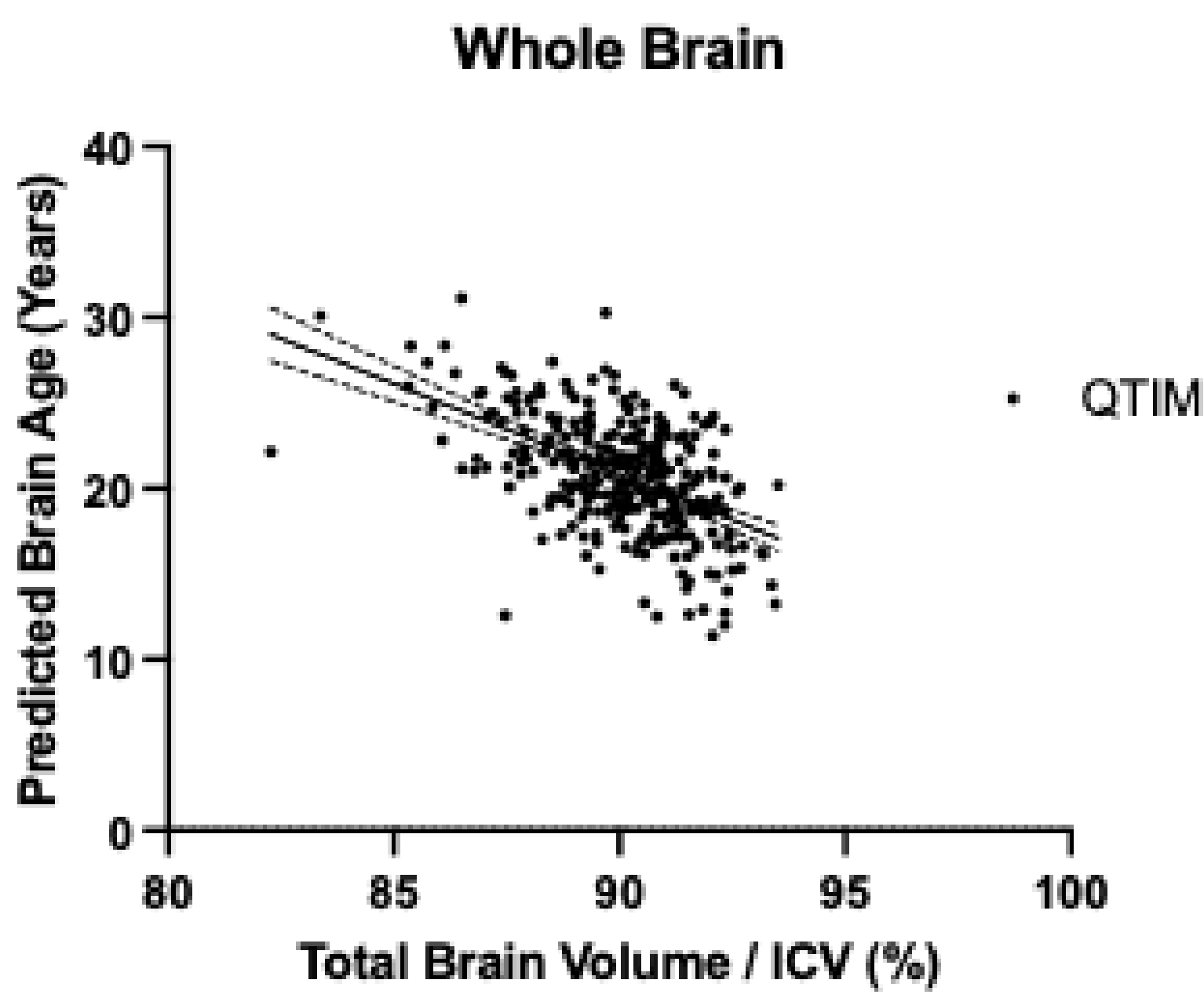

Supplement Figure 87. Correlation between whole brain volume controlled for ICV and predicted whole brain age gap estimation in QTIM neurotypical controls. Total brain volume correlated with predicted whole brain age in the QTIM neurotypical controls ($R^2 = 0.28$).

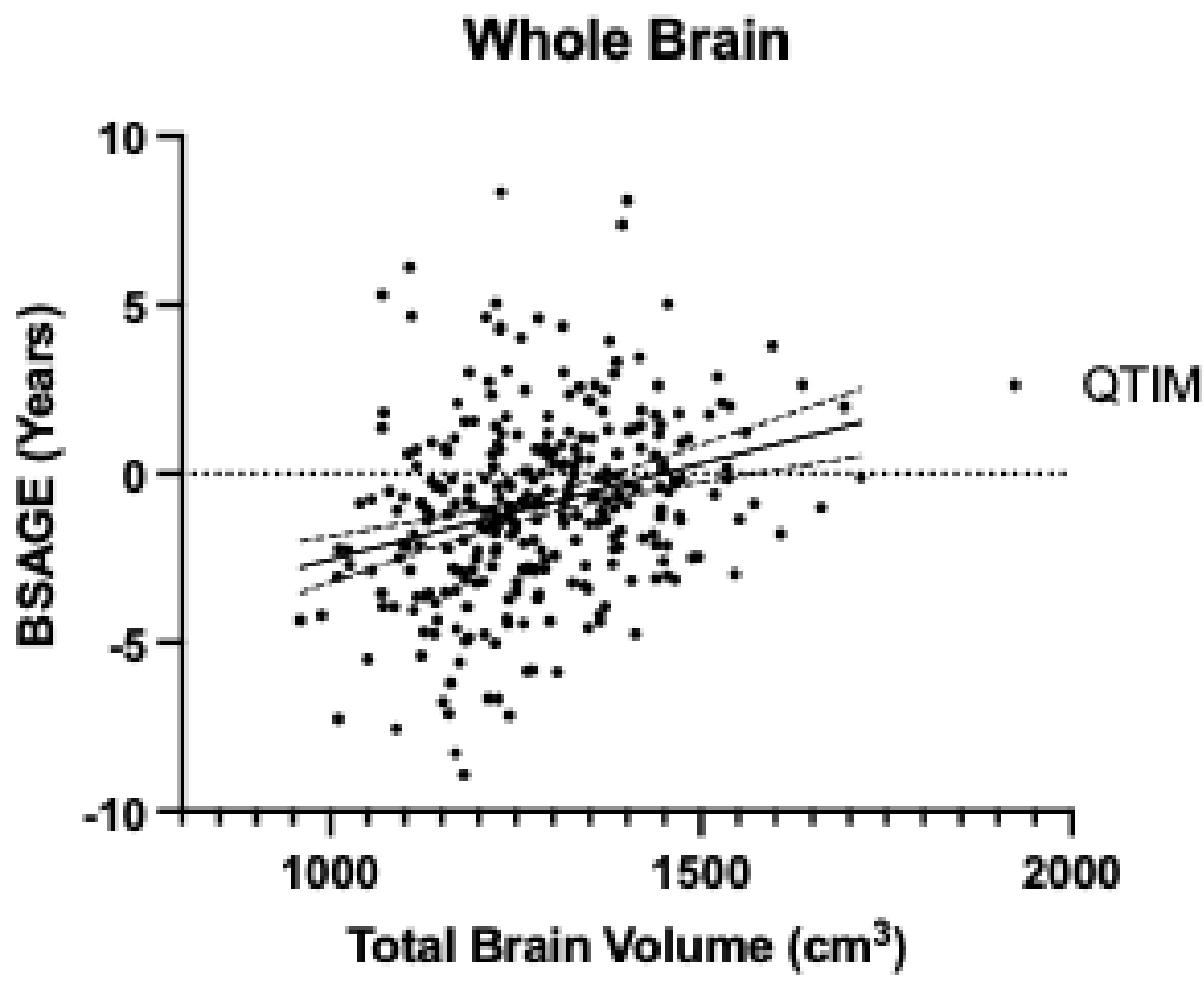

Supplement Figure 88. Correlation between whole brain volume (cm^3) and predicted whole brain, brain structures age gap estimation (BSAGE) in the QTIM neurotypical controls. Total brain volume correlated with predicted whole brain age in the QTIM neurotypical controls (R^2 = 0.08).

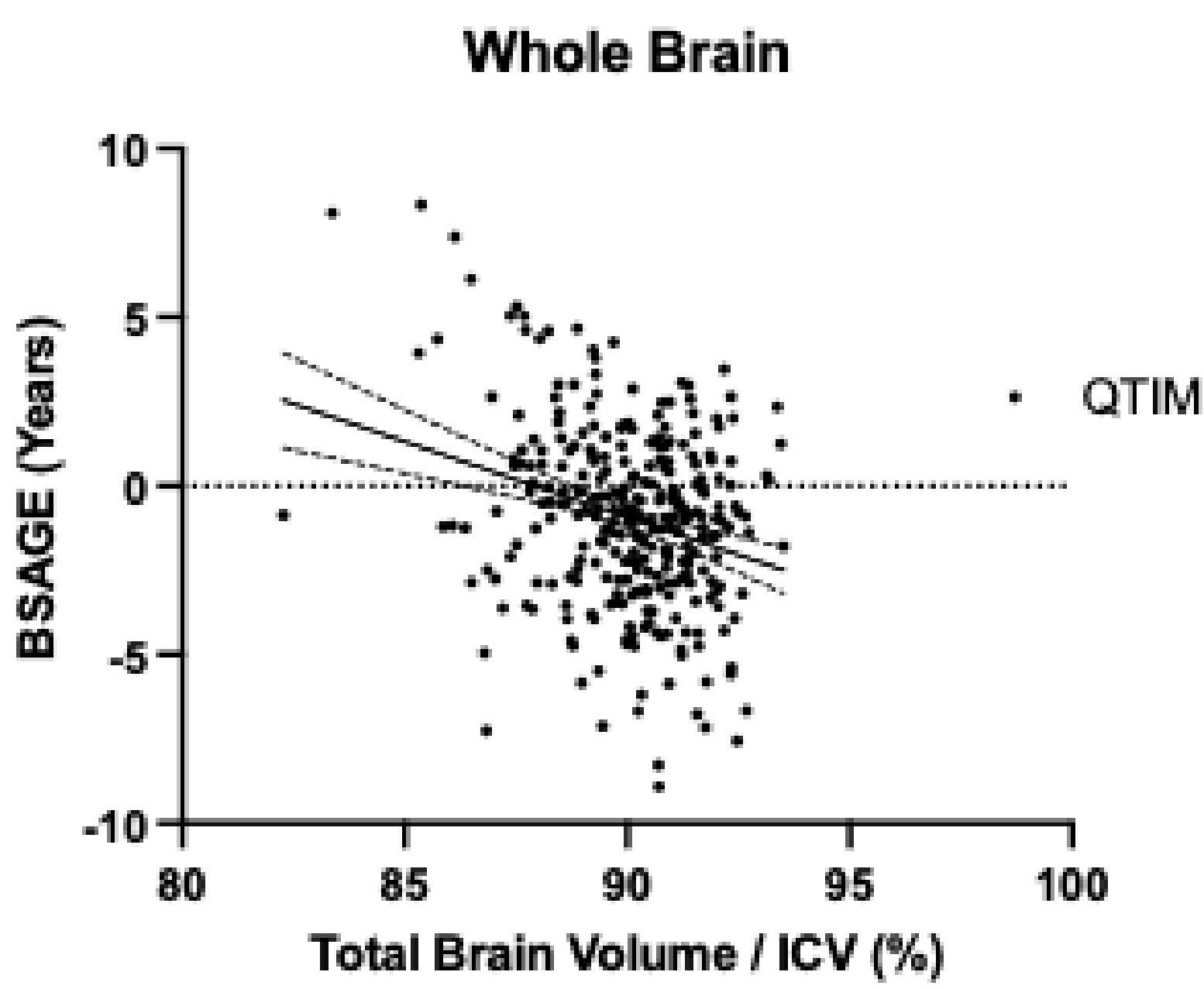

Supplement Figure 89. Correlation between whole brain volume controlled for total intracranial volume (ICV) and predicted whole brain, brain structures age gap estimation (BSAGE) in the QTIM neurotypical controls. Total brain volume correlated with predicted whole brain BSAGE in the QTIM neurotypical controls (R^2 = 0.08).

Supplement K: Descriptive evaluation of accelerated brain aging in GM1 cohort

Method

A regression-based percentile estimate was calculated from the NC data to calculate the normative predicted brain age at one, two, and three standard deviations above the mean at the age of each GM1 patient. The predicted brain age from Vol2brain was compared to the three values and the predicted values from the normative data as shown in Supplement Tables 5 and 6.

Supplement Table 5. Proportion of GM1 patients' baseline scan above the neurotypical control cohort.

***n* (%)**	**> 1 SD Above**	**> 2 SD Above**	**> 3 SD Above**
**Whole GM1 Cohort (*n* = 41)**	**30 (74%)**	**30 (74%)**	**30 (74%)**
**Juvenile GM1 (*n* = 26)**	**18 (69%)**	**18 (69%)**	**18 (69%)**
**Late-infantile GM1 (*n* = 15)**	**12 (80%)**	**12 (80%)**	**12 (80%)**

Supplement Table 6. Proportion of all GM1 patient scans above the neurotypical control cohort.

***Count* (%)**	**> 1 SD Above**	**> 2 SD Above**	**> 3 SD Above**
**Whole GM1 Cohort (with repeated scans out of 81)**	**66 (81%)**	**66 (81%)**	**66 (81%)**
**Juvenile Cohort (with repeated scans out of 61)**	**49 (80%)**	**49 (80%)**	**49 (80%)**
**Late-infantile Cohort (with repeated scans out of 20)**	**17 (85%)**	**17 (85%)**	**17 (85%)**

Supplementary References

1. National Human Genome Research Institute. Natural History of Glycosphingolipid Storage Disorders and Glycoprotein Disorders ClinicalTrials.gov identifier: NCT00029965. Updated August 7, 2024. Accessed August 12, 2024. https://clinicaltrials.gov/study/NCT00029965.
2. Reynolds JE, Long X, Paniukov D, Bagshawe M, Lebel C. Calgary Preschool magnetic resonance imaging (MRI) dataset. Data Brief. 2020;29:105224. Published 2020 Jan 31. doi:10.1016/j.dib.2020.105224
3. Reynolds, J., Long, X., Paniukov, D., Bagshawe, M., Dewey, D., & Lebel, C. (2023, June 13). Calgary Preschool MRI Dataset. https://doi.org/10.17605/OSF.IO/AXZ5R.
4. Strike, Lachlan T. and Hansell, Narelle K. and Miller, Jessica L. and Chuang, Kai-Hsiang and Thompson, Paul M. and de Zubicaray, Greig I. and McMahon, Katie L. and Wright, Margaret J. (2022). Queensland Twin Adolescent Brain (QTAB). OpenNeuro. [Dataset] doi: doi:10.18112/openneuro.ds004146.v1.0.3.
5. Strike, L.T., Hansell, N.K., Chuang, KH. *et al.* The Queensland Twin Adolescent Brain Project, a longitudinal study of adolescent brain development. *Sci Data* **10**, 195 (2023). https://doi.org/10.1038/s41597-023-02038-w.
6. Strike L. T., Hansell N. K., Couvy-Duchesne B., Thompson P. M., de Zubicaray G. I., McMahon K. L., Wright M. J. Genetic Complexity of Cortical Structure: Differences in Genetic and Environmental Factors Influencing Cortical Surface Area and Thickness. Cereb Cortex. 2019 Mar 1;29(3):952-962. https://doi.org/10.1093/cercor/bhy002.
7. Strike, Lachlan T. and Blokland, Gabriella A.M. and Hansell, Narelle K. and Martin, Nicholas G. and Toga, Arthur W. and Thompson, Paul M. and de Zubicaray, Greig I. and McMahon, Katie L. and Wright, Margaret J. (2022). Queensland Twin IMaging (QTIM). OpenNeuro. [Dataset] doi: doi:10.18112/openneuro.ds004169.v1.0.6.
8. Dessau RB, Pipper CB. R--en programpakke til statistisk databehandling og grafik ["R"--project for statistical computing]. *Ugeskr Laeger*. 2008;170(5):328-330.
9. Winter B, Linear models and linear mixed effects models in R with linguistic applications", 2013. 10.48550/arXiv.1308.5499.
10. Mittal N, Thakkar B, Hodges CB, et al. Effect of neuroanatomy on corticomotor excitability during and after transcranial magnetic stimulation and intermittent theta burst stimulation. Hum Brain Mapp. 2022;43(14):4492-4507. doi:10.1002/hbm.25968.
11. Lewis CJ, Johnston JM, Zaragoza Domingo S, et al. Retrospective assessment of clinical global impression of severity and change in GM1 gangliosidosis: a tool to score natural history data in rare disease cohorts. *Orphanet J Rare Dis*. 2025;20(1):125. Published 2025 Mar 14. doi:10.1186/s13023-025-03614-6
