# Supplementary material for: Brain aging in Type II GM1 gangliosidosis
Source: Commun Med (Lond). 2026 Jul 6;6:568. doi: 10.1038/s43856-026-01745-w (PMC13720018; doi:10.1038/s43856-026-01745-w)
Supplement: Supplementary file 3 [file 43856_2026_1745_MOESM3_ESM.docx]

**Description of Additional Supplementary Files**

File name: Supplementary Data 1

Description: The source data for Figures 1, 3, 4, 5, and 6

File name: Supplementary Data 2

Description: Brain Age Predictions Results from Linear Mixed Effects Modeling. Estimates and Standard Errors from the LMEM for the interaction between participant age and the cohort comparison can be found in Supplement Data 3.

File name: Supplementary Data 3

Description: Estimates and Standard Errors from the LMEM for comparisons of the whole GM1 cohort, Late-Infantile GM1 patients, Juvenile GM1 patients, and Neurotypical controls (NC) for each of the structures analyzed. The estimates for the intercept, followed by the interaction between the cohort and biological age, biological age alone, the cohort alone, and biological sex are defined in each cell with the standard errors in parenthesis.
