# Supplementary material for: Brain aging in Type II GM1 gangliosidosis
Source: Commun Med (Lond). 2026 Jul 6;6:568. doi: 10.1038/s43856-026-01745-w (PMC13720018; doi:10.1038/s43856-026-01745-w)
Supplement: Supplementary file 4 [file 43856_2026_1745_MOESM5_ESM.docx]

Supplement Data 2. Brain Age Predictions Results from Linear Mixed Effects Modeling.	Estimates and Standard Errors from the LMEM for the interaction between participant age and the cohort comparison can be found in Supplement Data 3.

| Structure | *GM1 v NC* | *Juvenile v NC* | *Late-Infantile v NC* | *Juvenile v Late-Infantile* |
| --- | --- | --- | --- | --- |
| ^2^(1)  *p-value*  *p-value_corrected_* |  |  |  |  |
| Whole Brain | 27.78  **1.4e-7**  **1.2e-5** | 67.32  **2.3e-16**  **2.0e-14** | 383.80  **1.8e-85**  **1.6e-83** | 15.91  **6.6e-5**  **0.0058** |
| White Matter | 40.18  **2.3e-10**  **2.0e-8** | 73.69  **9.1e-18**  **8.0e-16** | 322.4  **4.4e-72**  **3.9e-70** | 12.14  **0.00049**  **0.043** |
| 3^rd^ Ventricle | 22.29  **2.3e-6**  **0.00020** | 55.83  **7.9e-14**  **7.0e-12** | 236.90  **1.9e-53**  **1.7e-51** | 10.58  0.0011  0.1005 |
| 4^th^ Ventricle | 13.70  **0.00021**  **0.018** | 33.56  **6.9e-9**  **6.1e-7** | 424.0  **2.7e-94**  **2.4e-92** | 27.84  **6.5e-6**  **5.7e-4** |
| Inferior Lateral Ventricles | 13.38  **0.00026**  **0.023** | 25.42  **4.6e-7**  **4.0e-5** | 311.1  **1.3e-69**  **1.1e-67** | 13.06  **0.00030**  **0.026** |
| Lateral Ventricles | 41.46  **1.2e-10**  **1.1e-8** | 85.71  **2.1e-20**  **1.8e-18** | 307.0  **1.0e-68**  **8.8e-67** | 11.25  0.00080  0.070 |
| Ventral Diencephalon | 19.85  **8.38e-6**  **0.00074** | 47.44  **5.7e-12**  **5.0e-10** | 414.1  **4.6e-92**  **4.0e-90** | 20.33  **6.5e-6**  **5.7e-4** |
| External CSF | 53.83  **2.2e-13**  **1.9e-11** | 88.25  **6.0e-21**  **5.3e-19** | 294.2  **6.1e-66**  **5.4e-64** | 10.98  **0.00092**  **0.081** |
| Thalamus | 22.03  **2.7e-06**  **0.00024** | 52.80  **3.7e-13**  **3.3e-11** | 335.5  **6.1e-75**  **5.4e-73** | 14.02  **0.00018**  **0.016** |
| Caudate Nucleus | 47.75  **4.8e-12**  **4.2e-10** | 79.53  **4.7e-19**  **4.1e-17** | 211.6  **6.1e-48**  **< 0.0001** | 8.15  0.0043  0.38 |
| Nucleus Accumbens | 35.30  **2.8e-9**  **2.5e-7** | 49.73  **1.8e-12**  **1.6e-10** | 139.6  **3.3e-32**  **2.9e-30** | 4.90  0.027  1.000 |
| Putamen | 32.56  **1.2e-8**  **1.1e-6** | 64.28  **1.1e-15**  **9.7e-14** | 352.7  **1.1e-78**  **9.7e-77** | 13.81  **0.00020**  **0.018** |
| Globus Pallidus | 29.01  **7.2e-8**  **6.3e-6** | 60.59  **6.7e-15**  **5.9e-13** | 330.2  **8.6e-74**  **7.6e-72** | 13.13  **0.0003**  **0.026** |
| Hippocampus | 17.31  **3.2e-5**  **0.0028** | 31.71  **1.8e-8**  **1.6e-6** | 392.3  **2.6e-87**  **2.3e-85** | 18.88  **1.4e-5**  **0.0012** |
| Basal Forebrain | 31.50  **2.0e-8**  **1.8e-6** | 62.40  **2.8e-15**  **2.5e-13** | 378.7  **2.4e-84**  **2.1e-82** | 16.55  **4.7e-5**  **0.0041** |
| Amygdala | 27.36  **1.7e-7**  **1.5e-5** | 49.26  **2.2e-12**  **1.9e-10** | 346.5  **2.5e-77**  **< 0.0001** | 15.28  **9.3e-5**  **0.0082** |
| Cerebellar Gray Matter | 11.17  0.00083  0.073 | 27.67  **1.4e-7**  **1.2e-5** | 375.8  **1.0e-83**  **8.8e-82** | 25.14  **5.3e-7**  **4.7e-5** |
| Cerebellar White Matter | 13.25  **0.00027**  **0.024** | 31.81  **1.7e-8**  **1.5e-6** | 395.8  **4.5e-88**  **4.0e-86** | 25.79  **3.8e-7**  **3.3e-5** |
| Cerebellar Lobule I-V | 19.80  **8.6e-6**  **0.00076** | 40.37  **2.1e-10**  **1.8e-8** | 412.6  **1.7e-90**  **1.5e-88** | 19.28  **1.1e-5**  **0.00088** |
| Cerebellar Lobule VI-VII | 15.58  **7.9e-5**  **0.0070** | 40.80  **1.7e-10**  **1.5e-8** | 358.9  **4.8e-80**  **4.2e-78** | 22.93  **1.7e-6**  **0.00015** |
| Cerebellar Lobule VIII-X | 12.25  **0.00046**  **0.041** | 29.7  **5.0e-8**  **4.4e-6** | 340.3  **5.4e-76**  **4.8e-74** | 20.30  **6.6e-6**  **0.00058** |
| Brainstem | 16.53  **4.8e-5**  **0.0042** | 41.48  **1.2e-10**  **1.1e-8** | 422.1  **8.4e-94**  **7.4e-92** | 26.04  **3.4e-7**  **3.0e-5** |

*p-*values < 0.05 after a Bonferroni correction (*p-value_corrected_*) for multiple comparisons was considered significant and bolded.
