# Supplementary material for: Brain aging in Type II GM1 gangliosidosis
Source: Commun Med (Lond). 2026 Jul 6;6:568. doi: 10.1038/s43856-026-01745-w (PMC13720018; doi:10.1038/s43856-026-01745-w)
Supplement: Supplementary file 5 [file 43856_2026_1745_MOESM6_ESM.docx]

Supplement Data 3. Estimates and Standard Errors from the LMEM for comparisons of the

whole GM1 cohort, Late-Infantile GM1 patients, Juvenile GM1 patients, and Neurotypical controls (NC) for each of the structures analyzed. The estimates for the intercept, followed by the interaction between the cohort and biological age, biological age alone, the cohort alone, and biological sex are defined in each cell with the standard errors in parenthesis.

| **Structure** | | ***GM1 v NC*** | ***Juvenile v NC*** | ***Late-Infantile v NC*** | ***Juvenile v Late-Infantile*** |
| --- | --- | --- | --- | --- | --- |
| Intercept: Estimate (Standard Error)  Interaction: Estimate (Standard Error)  Age: Estimate (Standard Error)  Cohort: Estimate (Standard Error)  Sex: Estimate (Standard Error) | |  |  |  |  |
| **Whole Brain** | | 3.06 (0.75)  0.74 (0.14)  0.83 (0.04)  18.77 (1.73)  -0.74 (0.57) | 2.90 (0.53)  0.96 (0.11)  0.83 (0.03)  10.31 (1.61)  -0.51 (0.40) | 2.79 (0.47)  11.37 (0.52)  0.83 (0.02)  -21.29 (2.76)  -0.24 (0.36) | 20.06 (6.93)  10.70 (2.63)  1.65 (0.42)  -34.12 (15.04)  -13.06 (6.77) |
| **White Matter** | | 3.76 (0.76)  0.92 (0.14)  0.77 (0.04)  18.94 (1.77)  -0.76 (0.57) | 3.59 (0.57)  1.09 (0.12)  0.78 (0.03)  12.02 (1.74)  -0.47 (0.43) | 3.45 (0.49)  10.57 (0.54)  0.78 (0.03)  -18.32 (2.87)  -0.23 (0.38) | 22.81 (7.27)  9.67 (2.76)  1.73 (0.45)  -32.39 (15.78)  -13.92 (7.03) |
| **3^rd^ Ventricle** | | 3.74 (0.77)  0.62 (0.13)  0.79 (0.04)  20.38 (1.70)  -0.79 (0.58) | 3.50 (0.53)  0.83 (0.11)  0.80 (0.03)  12.35 (1.57)  -0.40 (0.41) | 3.41 (0.49)  8.98 (0.53)  0.80 (0.02)  -9.51 (2.84)  -0.22 (0.38) | 23.85 (6.81)  8.48 (2.58)  1.49 (0.40)  -24.57 (14.82)  -15.87 (6.87) |
| **4^th^ Ventricle** | | 5.04 (0.74)  0.42 (0.11)  0.74 (0.04)  17.31 (1.58)  -0.73 (0.57) | 4.88 (0.51)  0.47 (0.08)  0.74 (0.03)  9.65 (1.33)  -0.46 (0.39) | 4.76 (0.45)  11.30 (0.49)  0.74 (0.02)  -22.22 (2.61)  -0.23 (0.35) | 20.68 (5.69)    -35.25 (12.03)  -13.43 (6.42) |
| **Inferior Lateral Ventricles** | | 3.30 (0.76)  0.42 (0.11)  0.81 (0.04)  22.58 (1.61)  -0.85 (0.58) | 3.10 (0.56)  0.50 (0.10)  0.82 (0.03)  16.69 (1.53)  -0.54 (0.43) | 2.99 (0.48)  8.60 (0.45)  0.82 (0.02)  -7.93 (2.50)  -0.25 (0.38) | 27.48 (6.47)  8.45 (2.32)  1.19 (0.32)  -27.43 (13.76)  -15.32 (7.22) |
| **Lateral Ventricles** | | 5.04 (0.76)  0.94 (0.15)  0.70 (0.04)  17.72 (1.78)  -0.70 (0.57) | 4.87 (0.55)  1.16 (0.12)  0.71 (0.03)  9.81 (1.71)  -0.40 (0.42) | 4.69 (0.50)  10.40 (0.54)  0.71 (0.03)  -17.86 (2.91)  -0.16 (0.38) | 21.95 (7.35)  9.36 (2.78)  1.74 (0.46)  -29.34 (15.90)  -14.18 (2.78) |
| **Ventral Diencephalon** | | 4.19 (0.75)  0.58 (0.13) 0.77 (0.04)  19.89 (1.68)  -0.75 (0.57) | 4.19 (0.75)  0.70 (0.10) 0.77 (0.04)  19.89 (1.68)  -0.75 (0.57) | 3.87 (0.46)  11.54 (0.50)  0.77 (0.02)  -21.35 (2.69)  -0.21 (0.36) | 23.46 (6.46)  11.36 (2.43)  1.36 (0.37)  -37.08 (14.04)  -14.62 (6.73) |
| **External CSF** | 5.14 (0.74)  1.04 (0.14)  0.73 (0.04)  16.45 (1.73)  -0.80 (0.56) | | 5.14 (0.74)  1.20 (0.12)  0.73 (0.04)  16.45 (1.73)  -0.80 (0.56) | 4.85 (0.47)  9.76 (0.53)  0.74 (0.02)  -16.91 (2.80)  -0.30 (0.36) | 21.58 (7.03)  8.83 (2.67)  1.83 (0.44)  -29.15 (15.23)  -13.29 (6.74) |
| **Thalamus** | 3.34 (0.76)  0.63 (0.13)  0.80 (0.04)  20.65 (1.72)  -0.75 (0.58) | | 3.34 (0.76)  0.81 (0.11)  0.80 (0.04)  20.65 (1.72)  -0.75 (0.58) | 3.03 (0.50)  10.57 (0.52)  0.80 (0.03)  -16.54 (2.82)  -0.19 (0.39) | 23.50 (6.94)  9.94 (2.62)  1.47 (0.40)  -31.39 (15.09)  -14.55 (7.09) |
| **Caudate Nucleus** | 3.44 (0.78)  1.05 (0.15)  0.79 (0.04)  15.88 (1.84)  -0.64 (0.59) | | 3.44 (0.78)  1.16 (0.13)  0.79 (0.04)  15.88 (1.84)  -0.64 (0.59) | 3.13 (0.53)  9.30 (0.60)  0.80 (0.03)  -17.63 (3.18)  -0.07 (0.41) | 21.17 (8.01)  8.57 (3.03)  1.80 (0.51)  -32.17 (17.30)  -11.83 (7.58) |
| **Nucleus Accumbens** | 3.82 (0.73)  0.93 (0.15)  0.79 (0.04)  14.63 (1.82)  -0.42 (0.56) | | 3.82 (0.73)  0.98 (0.14)  0.79 (0.04)  14.63 (1.82)  -0.42 (0.56) | 3.50 (0.49)  7.37 (0.59)  0.79 (0.03)  -12.17 (3.11)  0.14 (0.38) | 21.01 (8.35)  6.68 (3.10)  1.65 (0.56)  -26.48 (17.68)  -9.81 (7.29) |
| **Putamen** | 4.01 (0.78)  0.82 (0.14)  0.76 (0.04)  20.21 (1.78)  -0.71 (0.59) | | 3.86 (0.55)  0.97 (0.12)  0.76 (0.03)  13.46 (1.68)  -0.47 (0.42) | 3.69 (0.50)  11.30 (0.54)  0.76 (0.03)  -21.02 (2.90)  -0.12 (0.39) | 25.19 (7.38)    -36.99 (16.06)  -14.31 (7.39) |
| **Globus Pallidus** | 3.82 (0.78) 0.77 (0.14)  0.78 (0.04)  19.97 (1.78)  -0.70 (0.59) | | 3.66 (0.55) 0.93 (0.42)  0.78 (0.03)  13.04 (1.66)  -0.47 (0.42) | 3.52 (0.51)  11.11 (0.55)  0.78 (0.03)  -20.39 (2.96)  -0.12 (0.40) | 24.30 (7.42)  10.33 (2.81)  1.54 (0.44)  -35.49 (16.14)  -13.98 (7.43) |
| **Hippocampus** | 4.54 (0.74)  0.51 (0.12)  0.73 (0.04)  21.92 (1.66)  -0.52 (0.57) | | 4.41 (0.53)  0.56 (0.10)  0.74 (0.03)  16.08 (1.56)  -0.28 (0.41) | 4.29 (0.47)  10.85 (0.49)  0.74 (0.02)  -19.08 (2,64)  -0.12 (0.37) | 26.36 (6.61)    -37.20 (14.07)  -13.55 (7.45) |
| **Basal Forebrain** | 4.63 (0.78)  0.84 (0.15)  0.76 (0.04)  19.11 (1.82)  -0.62 (0.59) | | 4.49 (0.56)  0.98 (0.12)  0.76 (0.03)  12.09 (1.71)  -0.39 (0.43) | 4.30 (0.49)  12.04 (0.56)  0.76 (0.03)  -24.73 (2.95)  -0.05 (0.38) | 23.93 (7.36)  11.63 (2.79  1.60 (0.45)  -40.74 (15.97)  -14.00 (7.17) |
| **Amygdala** | 4.70 (0.78)  0.68 (0.13)  0.76 (0.04)  19.48 (1.70)  -0.66 (0.59) | | 4.70 (0.78)  0.77 (0.11)  0.76 (0.04)  19.48 (1.70)  -0.66 (0.59) | 4.34 (0.49)  10.50 (0.51)  0.76 (0.03)  -18.77 (2.77)  -0.06 (0.38) | 25.70 (6.83)    -35.18 (14.77)  -14.47 (7.32) |
| **Cerebellar Gray Matter** | 6.36 (0.71)  0.38 (0.11)  0.65 (0.04)  16.61 (1.55)  -0.72 (0.54) | | 6.36 (0.71)  0.44 (0.08)  0.65 (0.04)  16.61 (1.55)  -0.72 (0.54) | 6.09 (0.45)  10.61 (0.49)  0.65 (0.02)  -20.90 (2.63)  -0.20 (0.35) | 21.80 (5.60)  10.89 (2.05)  1.04 (0.29)  -34.45 (12.02)  -13.46 (6.11) |
| **Cerebellar White Matter** | 5.55 (0.73)  0.43 (0.12)  0.70 (0.04)  17.33 (1.59)  -0.77 (0.56) | | 5.55 (0.73)  0.46 (0.08)  0.70 (0.04)  17.33 (1.59)  -0.77 (0.56) | 5.26 (0.45)  11.04 (0.50)  0.70 (0.02)  -21.60 (2.65)  -0.23 (0.35) | 21.66 (5.80)  11.32 (2.10)  1.12 (0.29)  -35.91 (12.38)  -13.77 (6.43) |
| **Cerebellar Lobule I-V** | 4.42 (0.76)  0.56 (0.13)  0.76 (0.04)  20.54 (1.67)  -0.80 (0.58) | | 4.42 (0.76)  0.64 (0.10)  0.76 (0.04)  20.54 (1.67)  -0.80 (0.58) | 4.09 (0.47)  11.22 (0.49)  0.76 (0.02)  -20.07 (2.67)  -0.23 (0.37) | 25.40 (6.49)  10.93 (2.41)  1.30 (0.35)  -36.45 (14.02)  -15.26 (6.94) |
| **Cerebellar Lobule VI-VII** | 5.13 (0.71)  0.45 (0.11)  0.72 (0.04)  17.44 (1.55)  -0.70 (0.54) | | 5.13 (0.71)  0.52 (0.08)  0.72 (0.04)  17.44 (1.55)  -0.70 (0.54) | 4.86 (0.46)  10.46 (0.50)  0.73 (0.02)  -18.56 (2.67)  -0.23 (0.35) | 20.95 (5.68)    -31.90 (12.23)  -13.56 (6.15) |
| **Cerebellar Lobule VIII-X** | 5.24 (0.70)  0.38 (0.11)  0.72 (0.04)  17.00 (1.50)  -0.73 (0.54) | | 5.24 (0.70)  0.44 (0.08)  0.72 (0.04)  17.00 (1.50)  -0.73 (0.54) | 4.97 (0.45)  9.57 (0.47)  0.72 (0.02)  -15.69 (2.54)  -0.23 (0.35) | 20.94 (5.58)  9.42 (2.02)  1.12 (0.28)  -27.54 (11.92)  -13.40 (6.18) |
| **Brainstem** | 4.80 (0.75)  0.51 (0.13)  0.75 (0.04)  17.99 (1.65)  -0.73 (0.57) | | 4.63 (0.52)  0.58 (0.09)  0.75 (0.03)  10.25 (1.40)  -0.45 (0.40) | 4.50 (0.46)  11.83 (0.51)  0.75 (0.02)  -23.93 (2.72)  -0.20 (0.35) | 21.36 (6.07)    -39.36 (13.09)  -13.89 (6.56) |
